# Supplementary figures and images for: Mg2+-dependent mechanism of environmental versatility in a multidrug efflux pump
Source: bioRxiv. 2024 Jun 10:2024.06.10.597921. Preprint. [Version 1] doi: 10.1101/2024.06.10.597921 (PMC11195059; doi:10.1101/2024.06.10.597921)

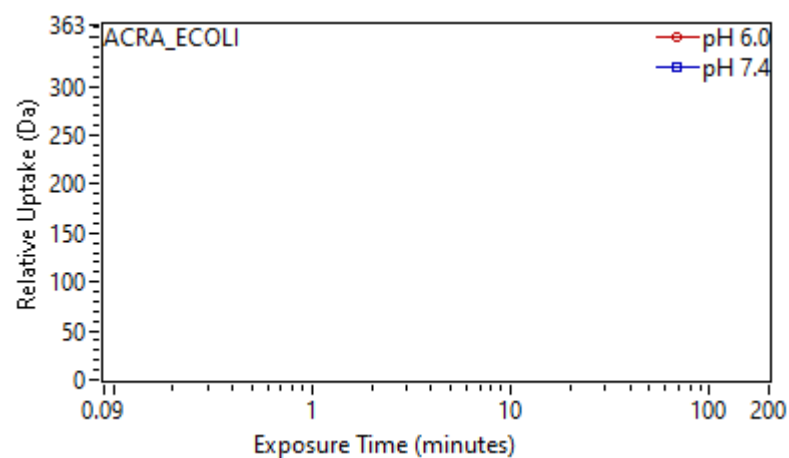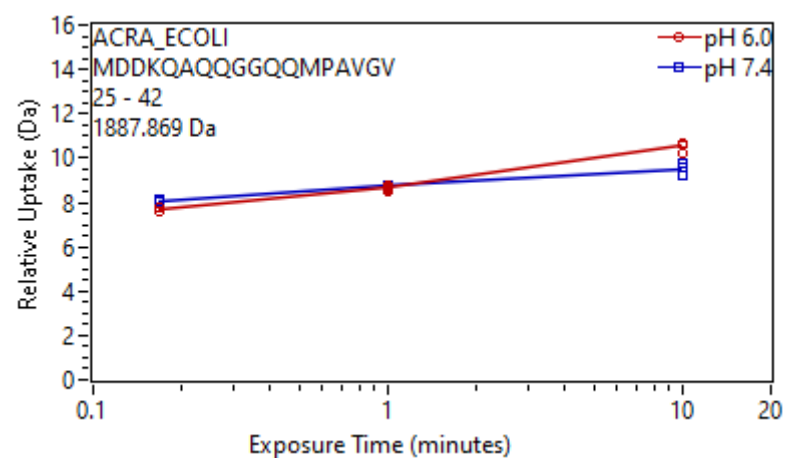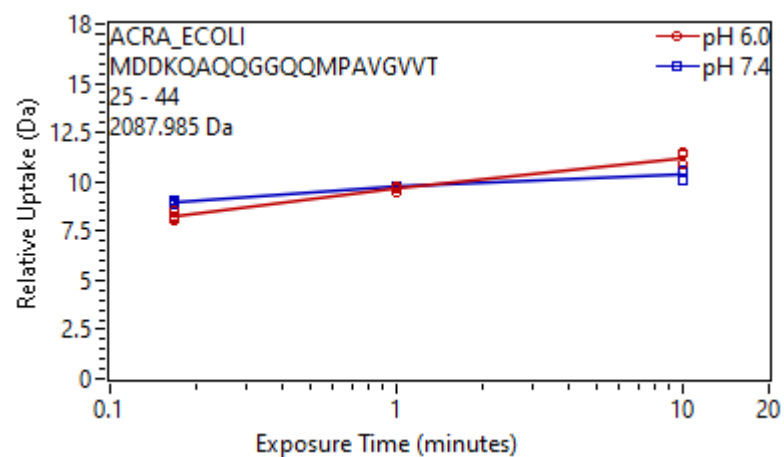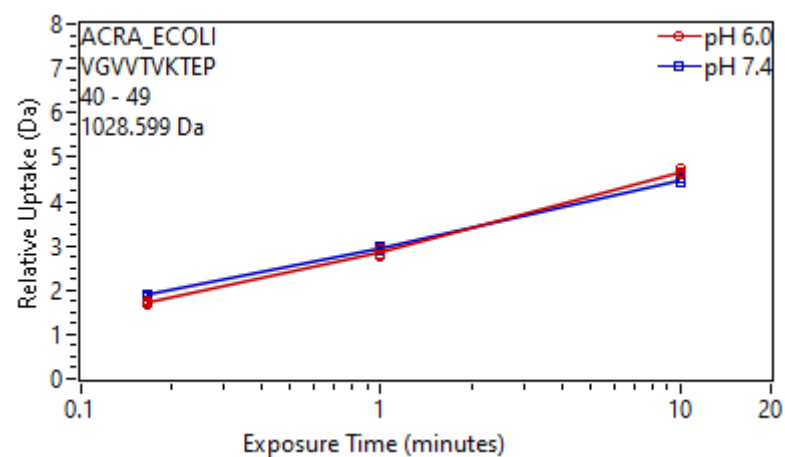

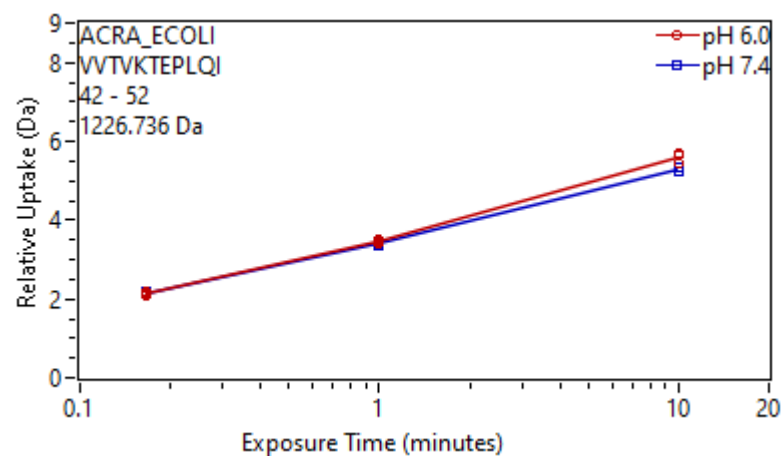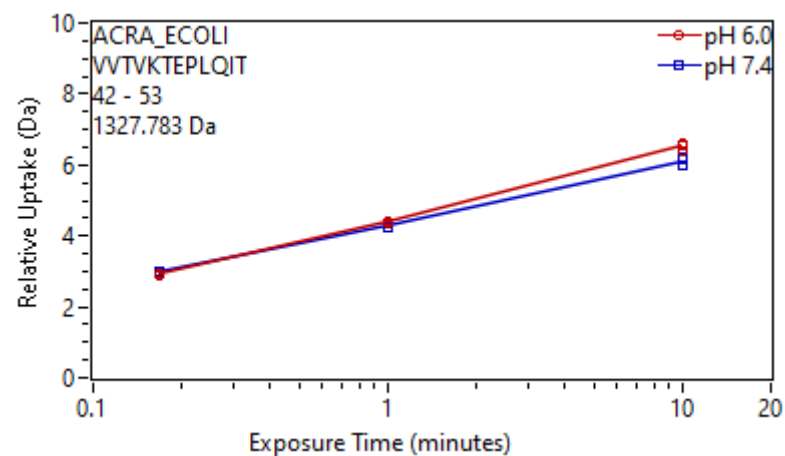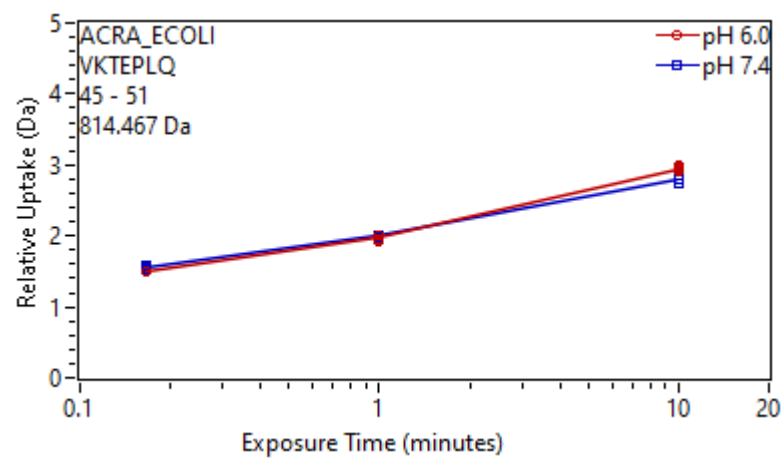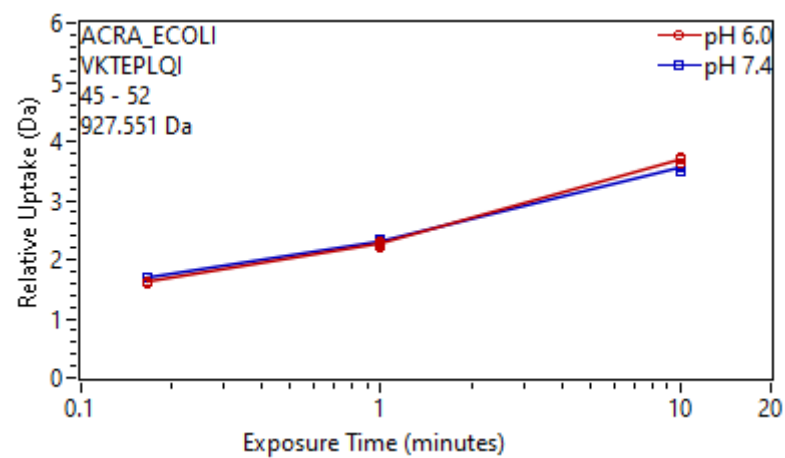

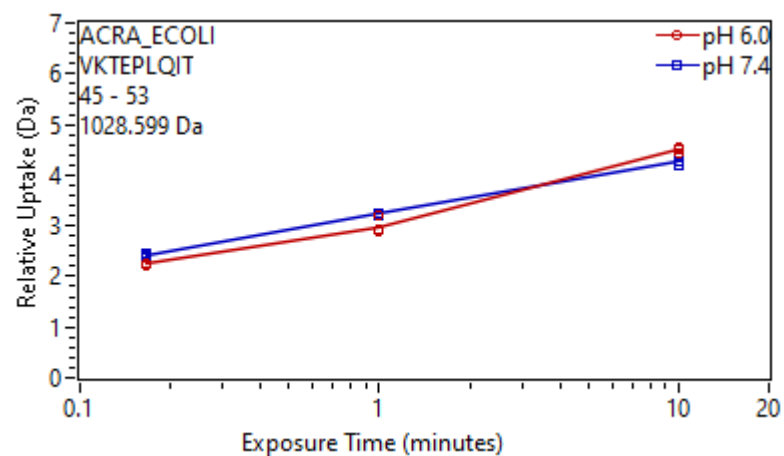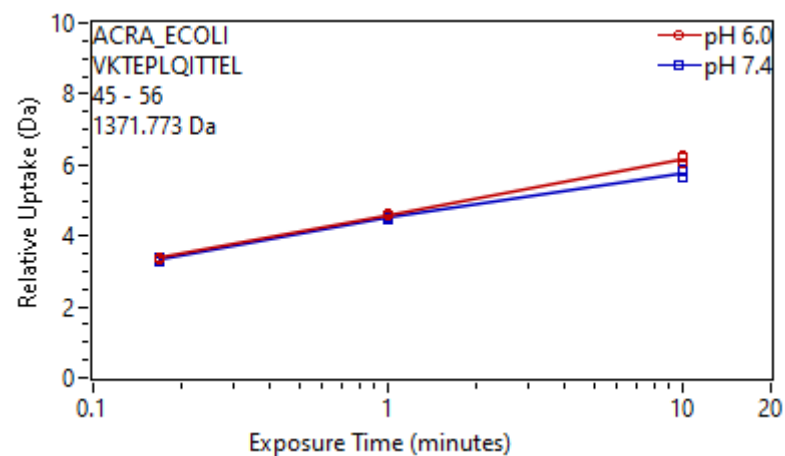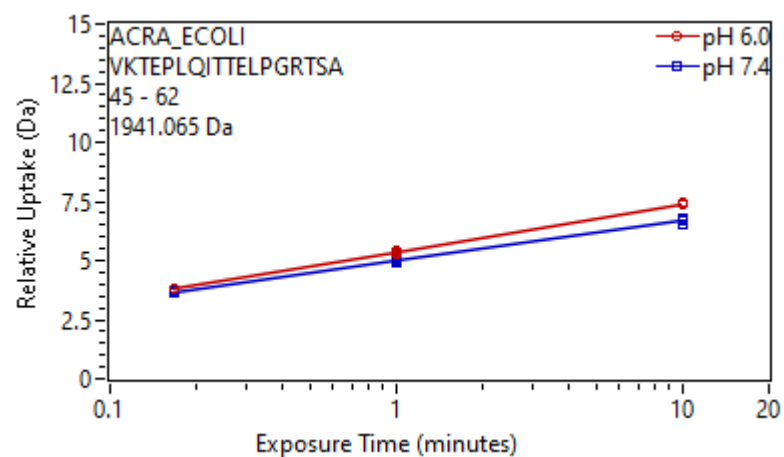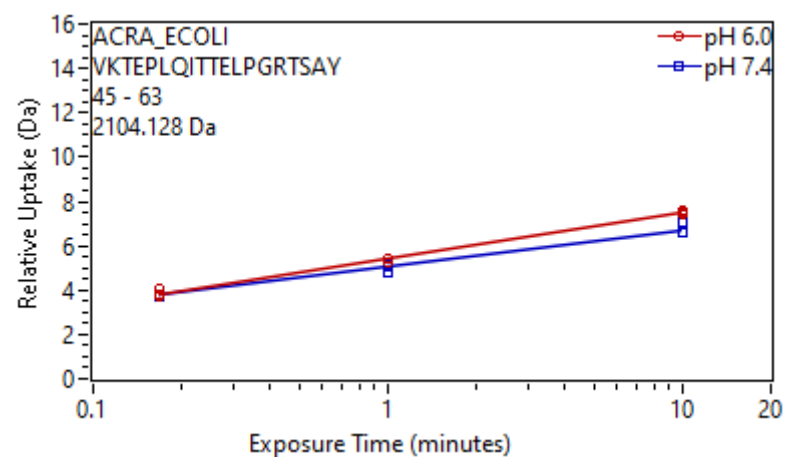

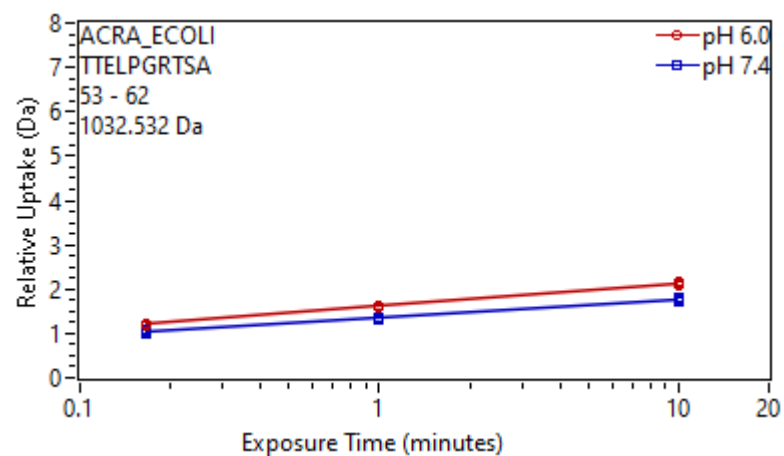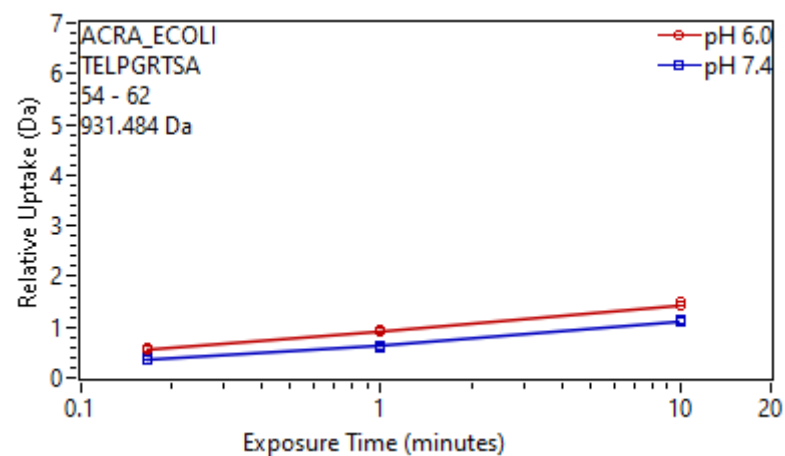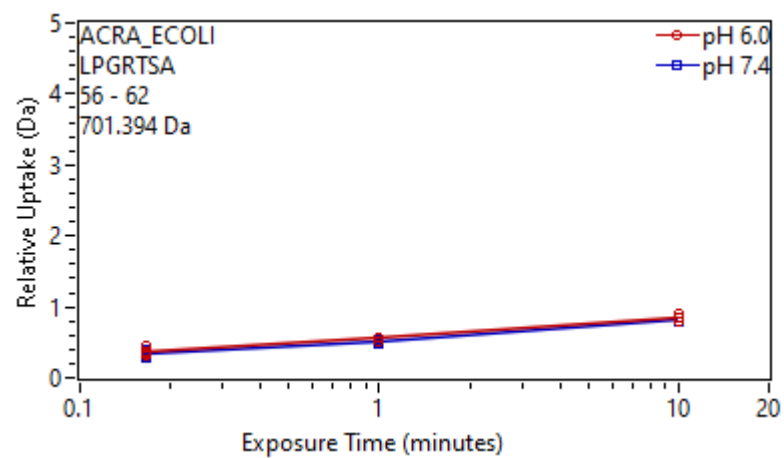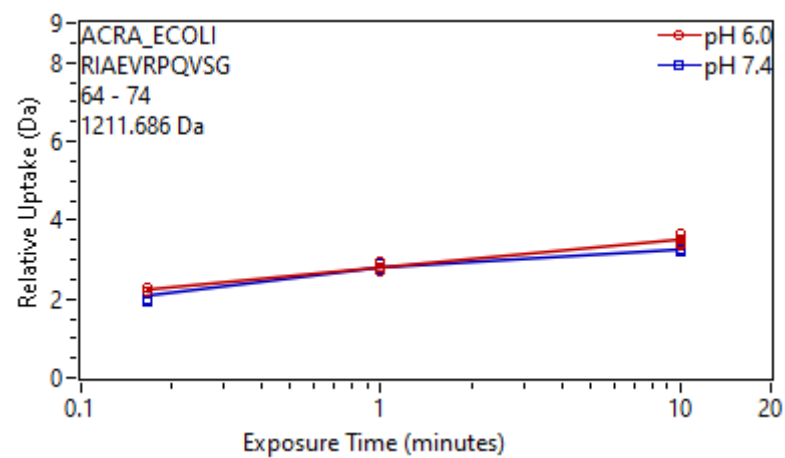

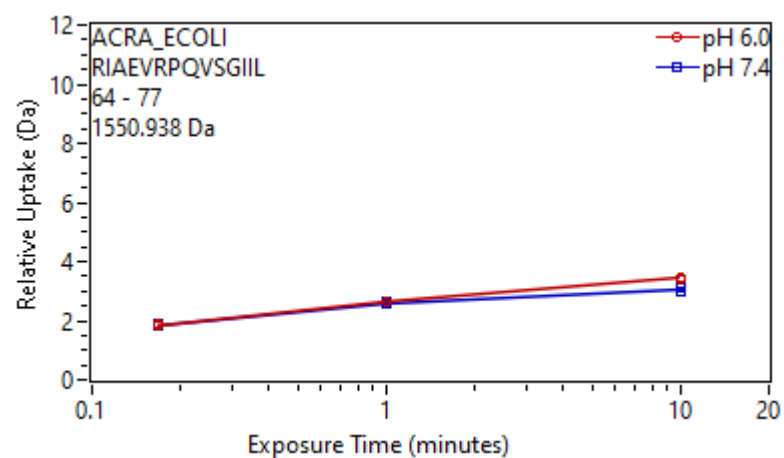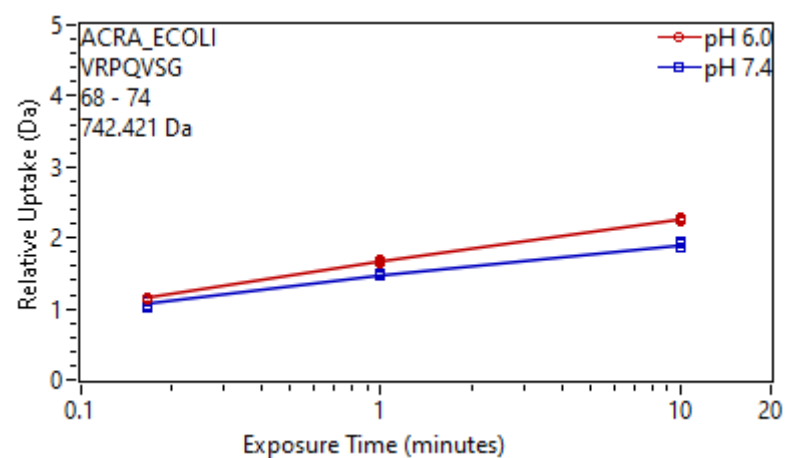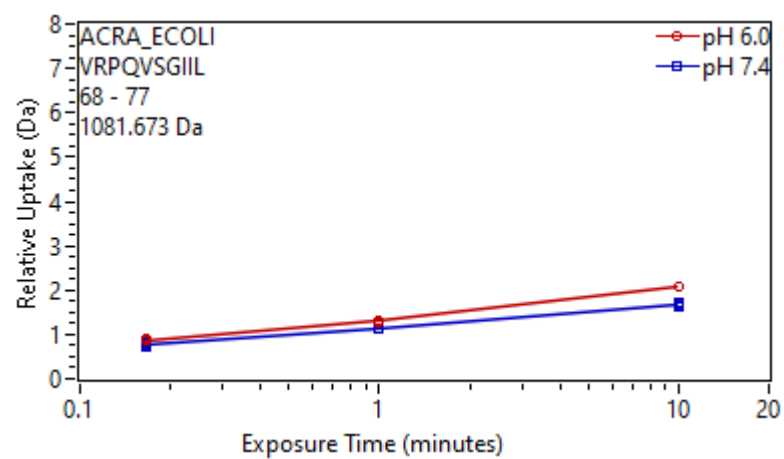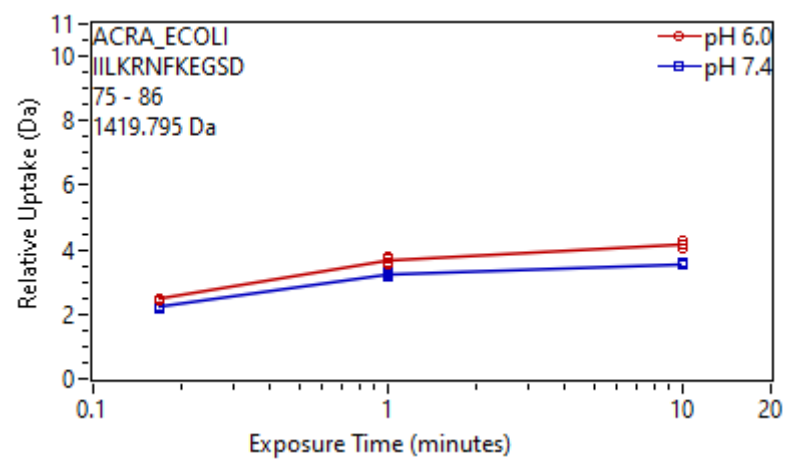

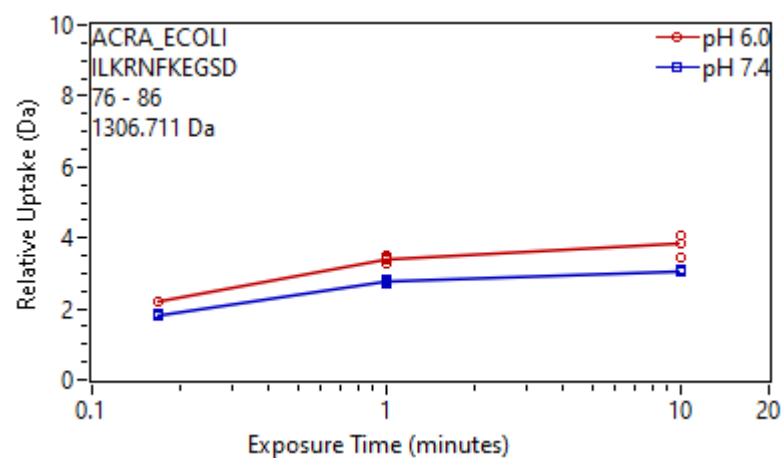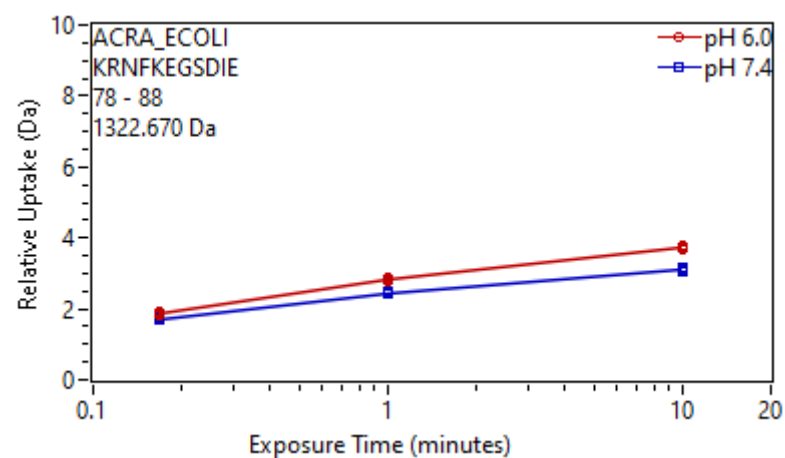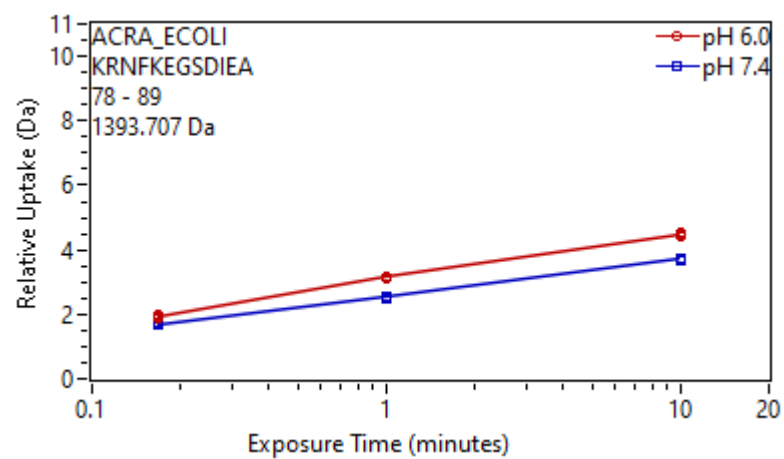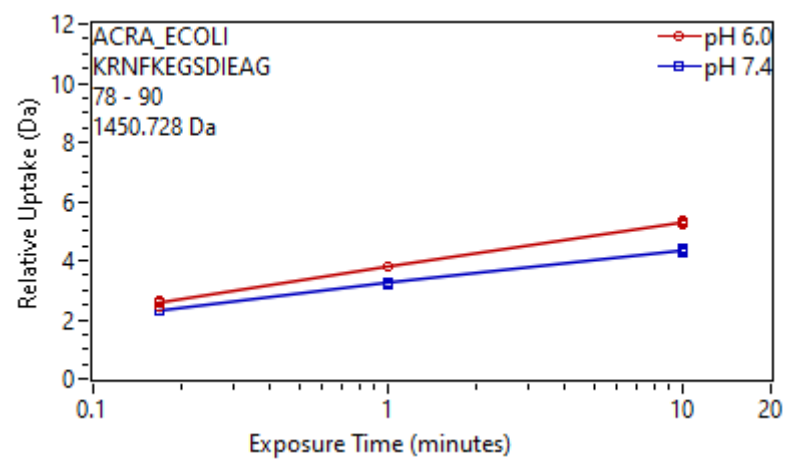

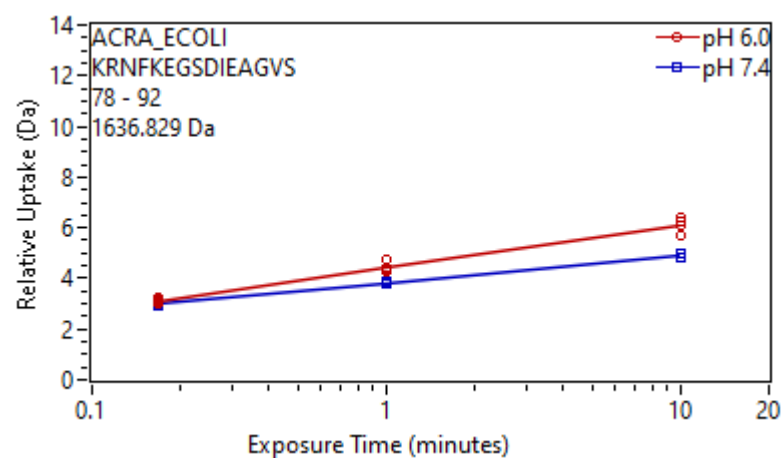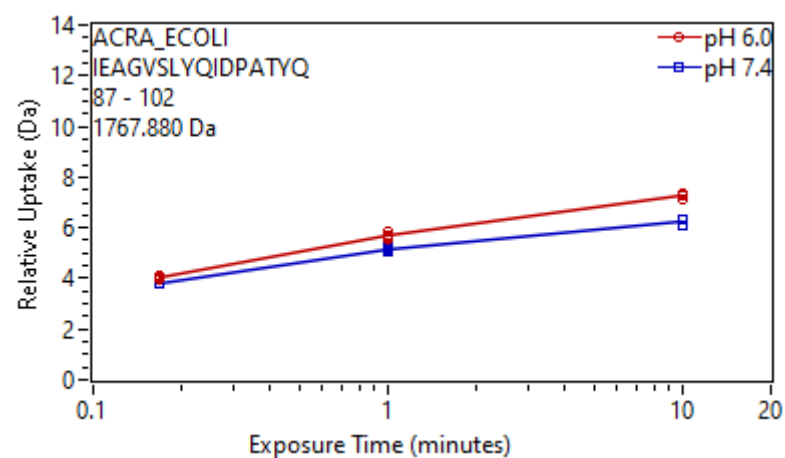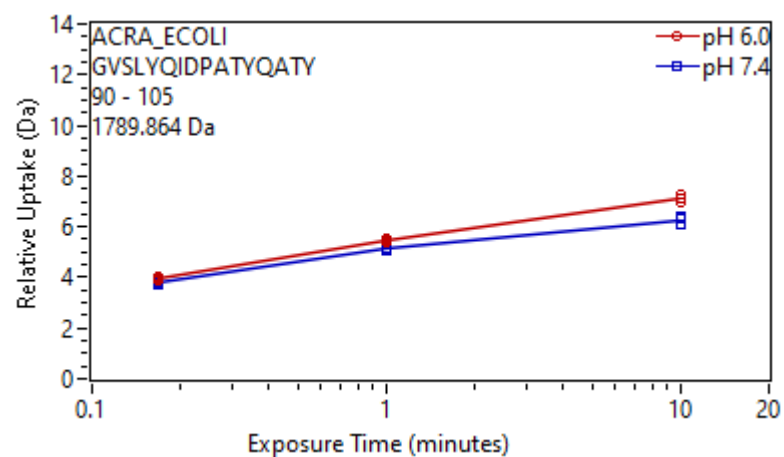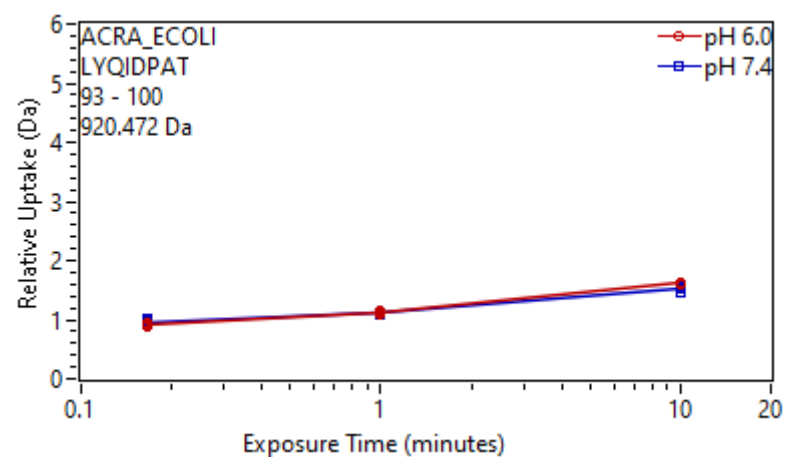

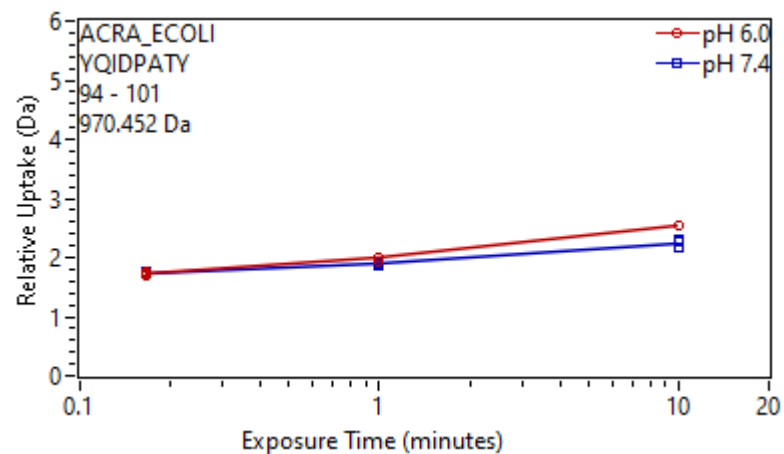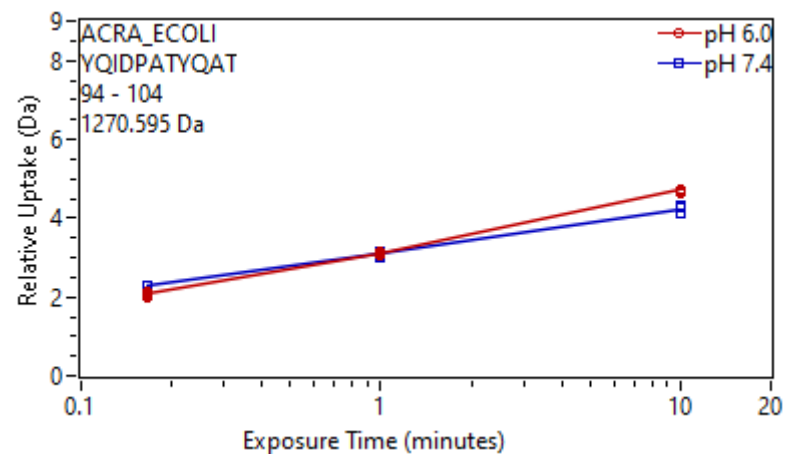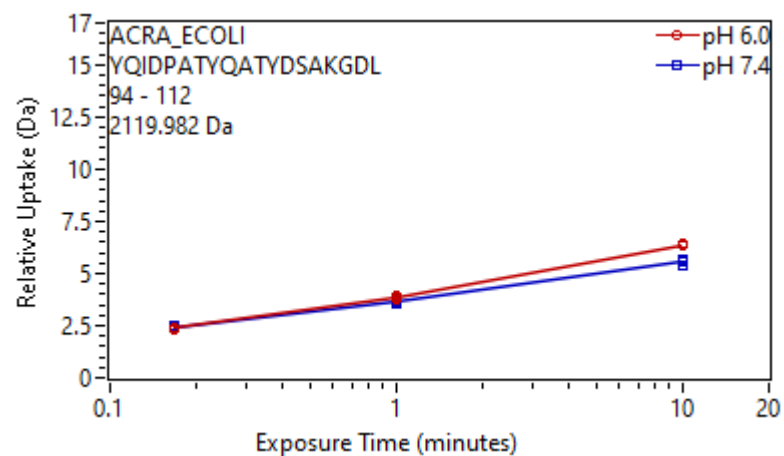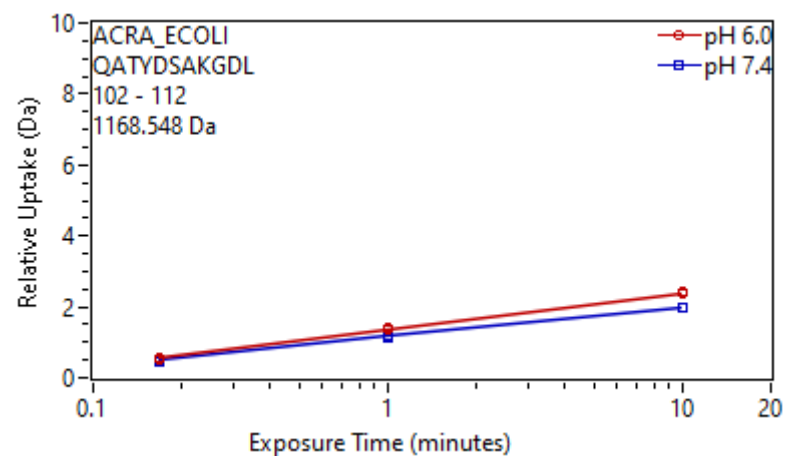

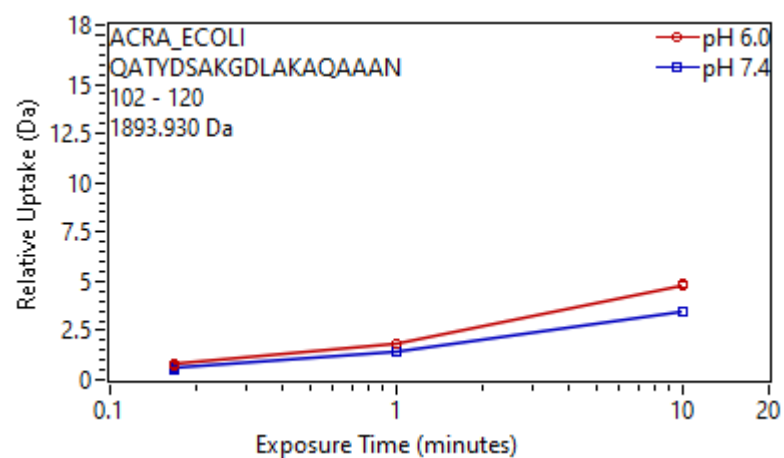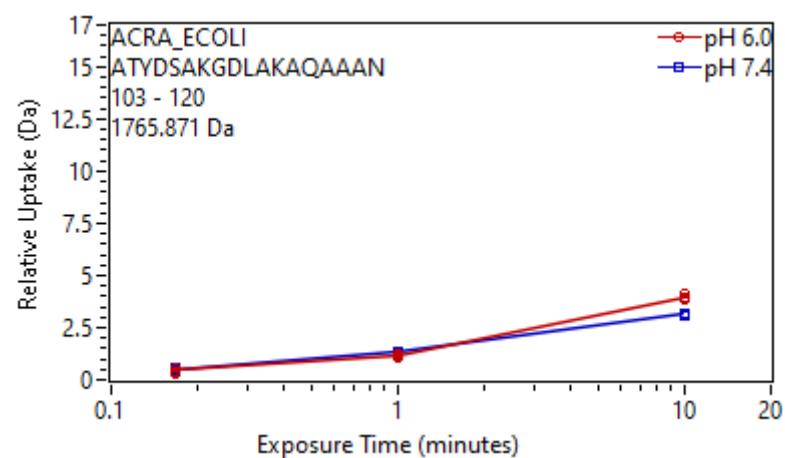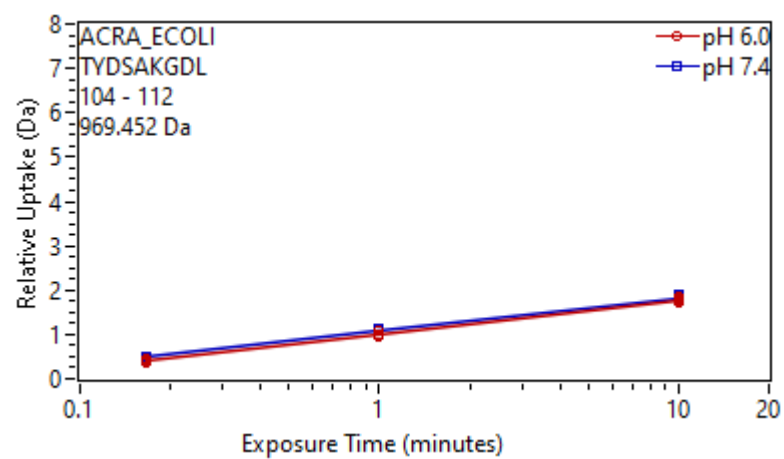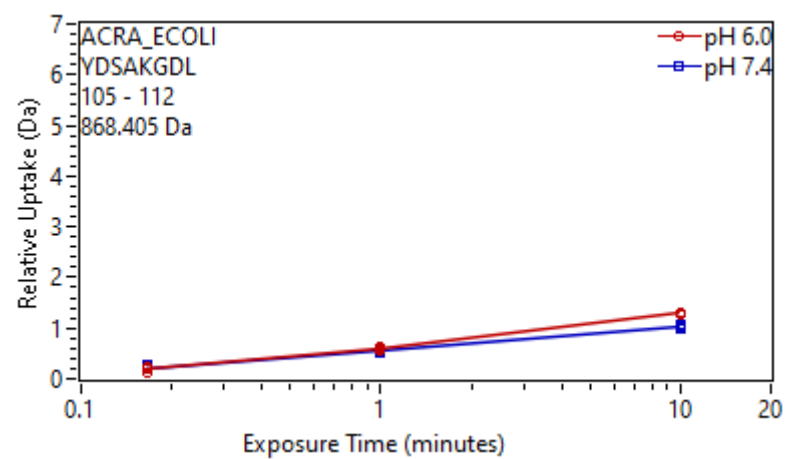

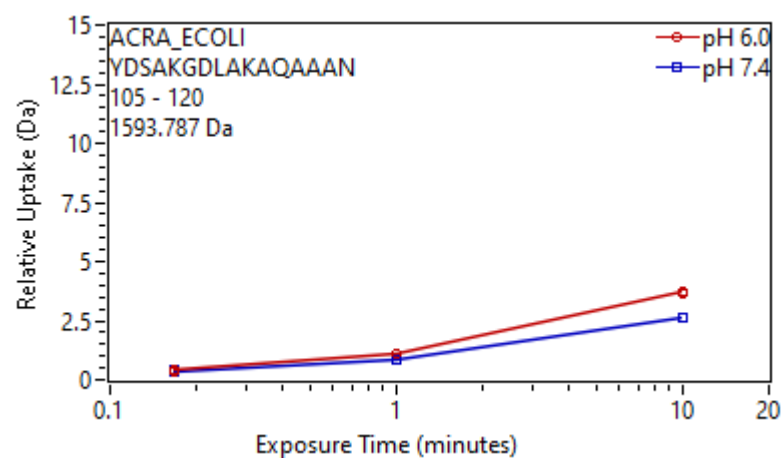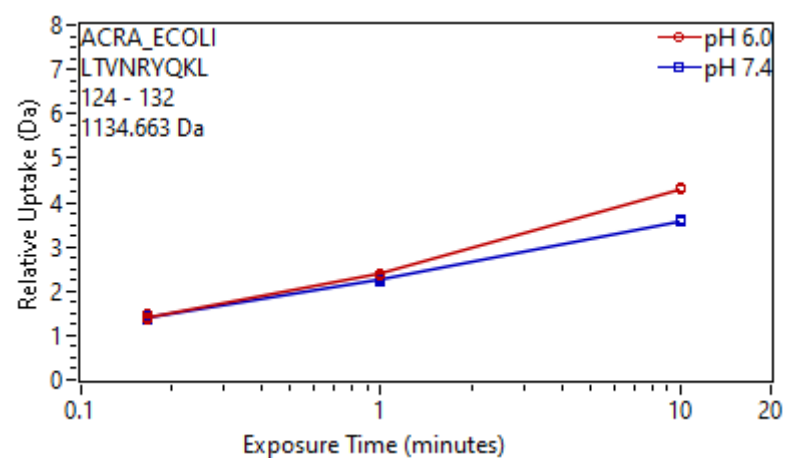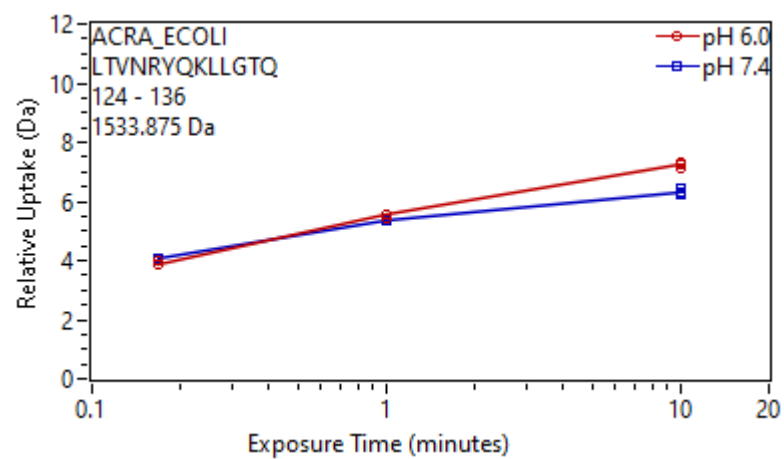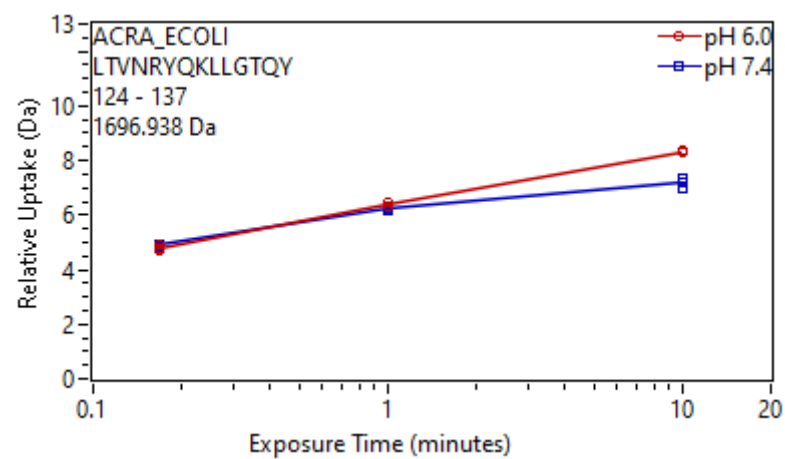

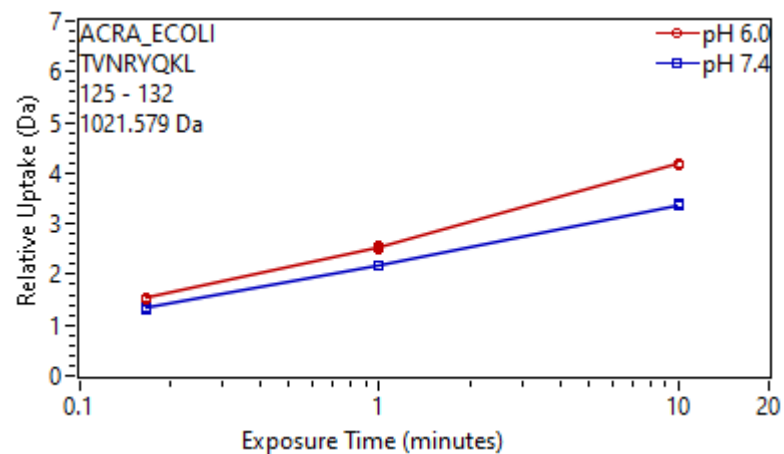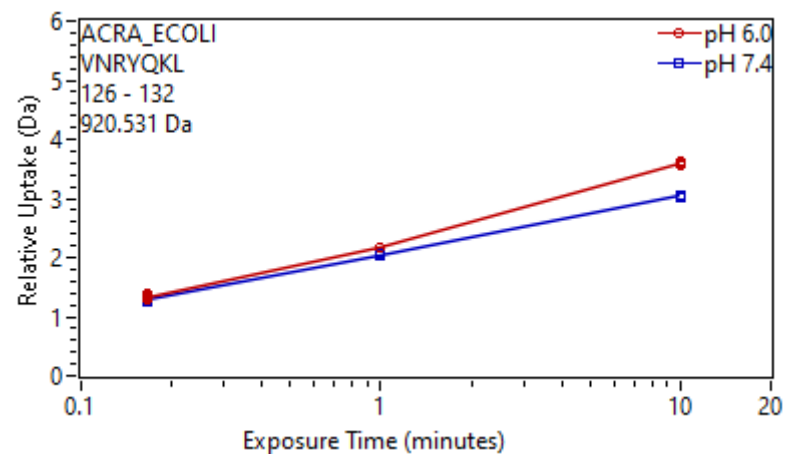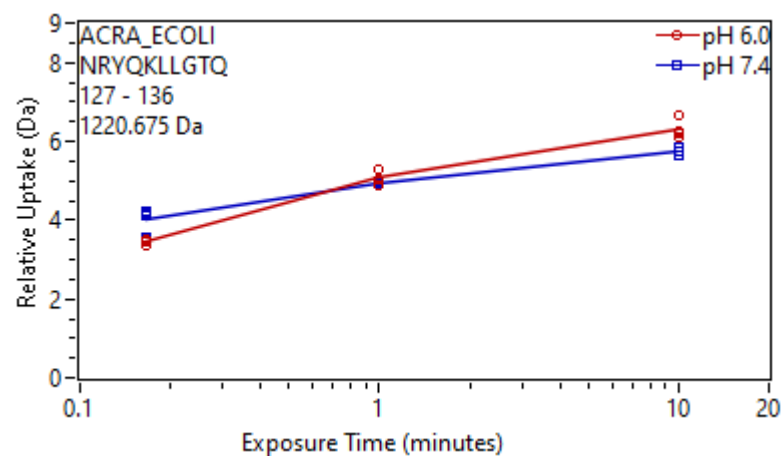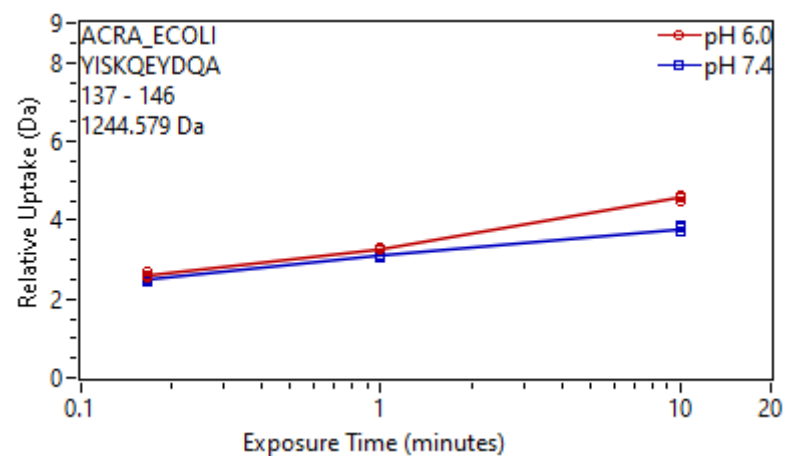

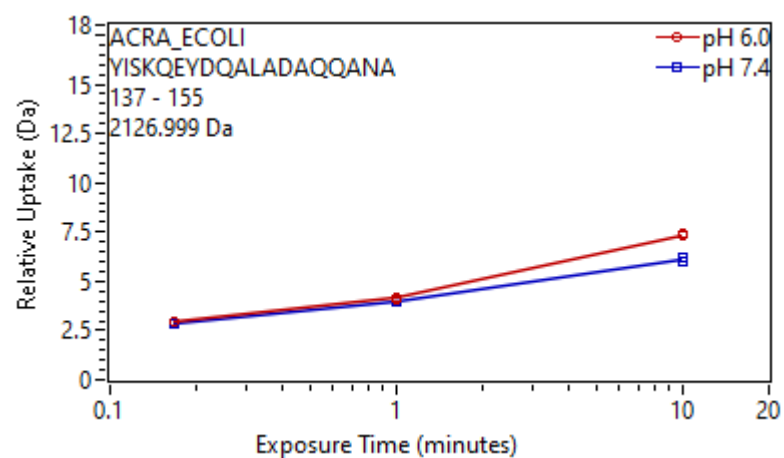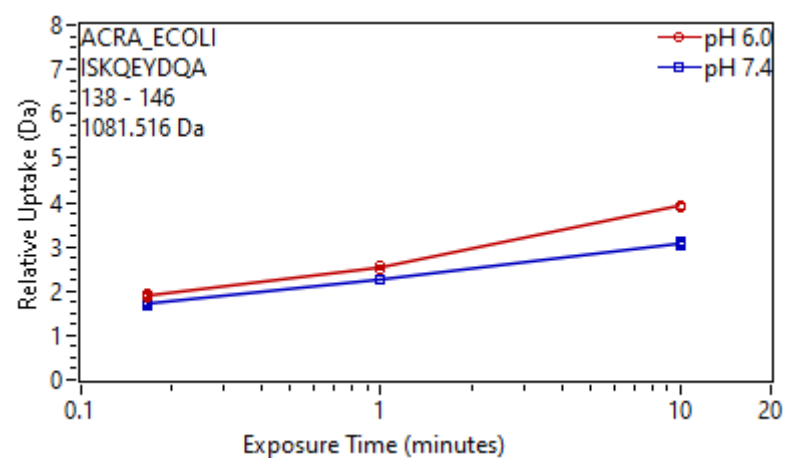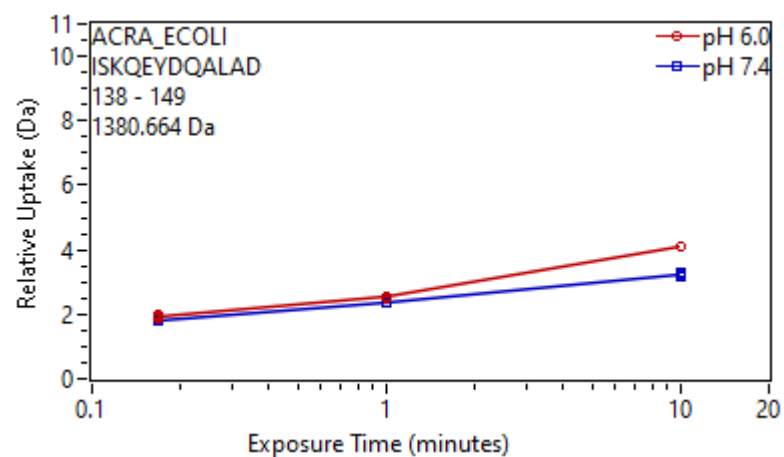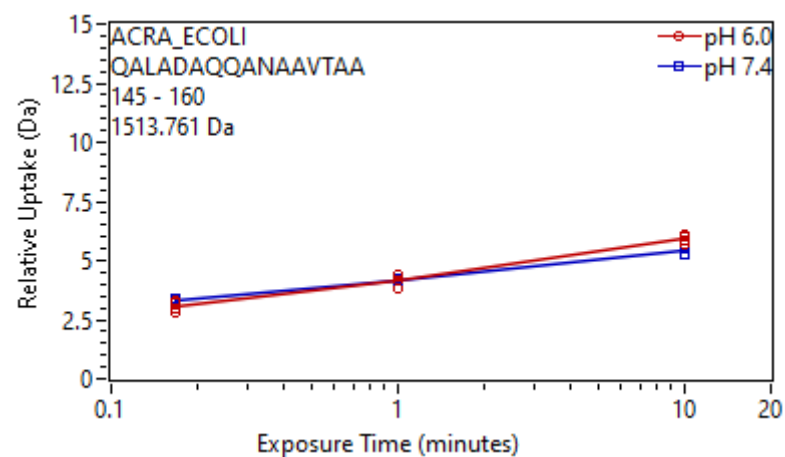

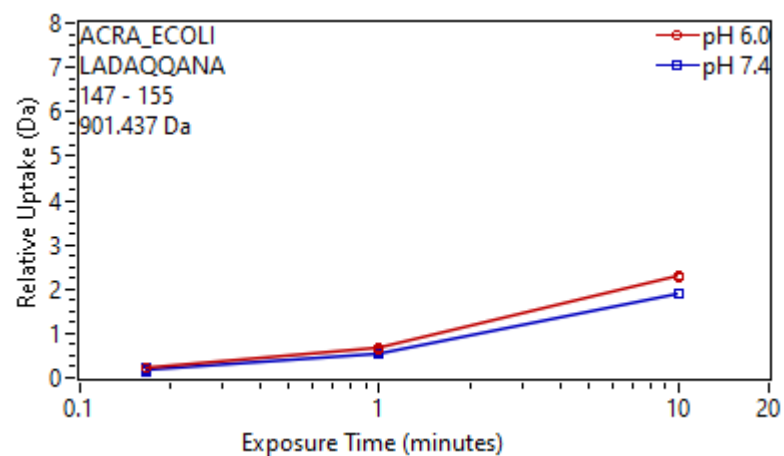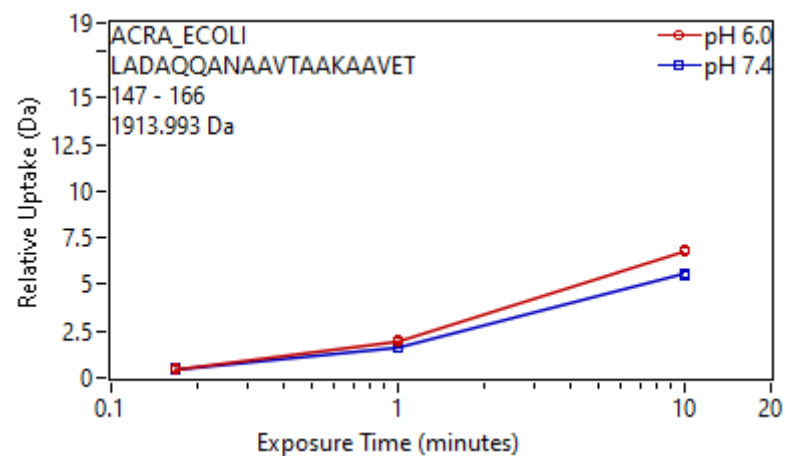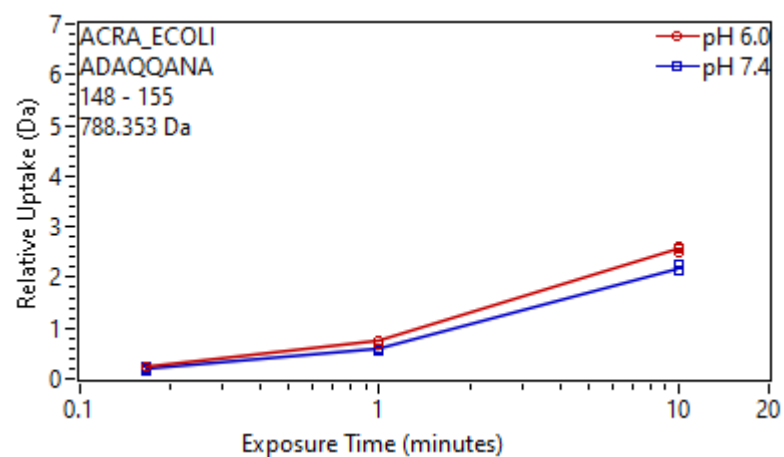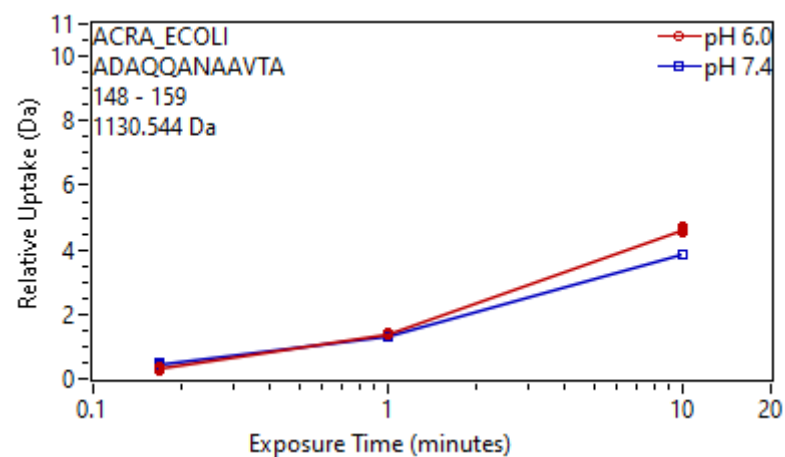

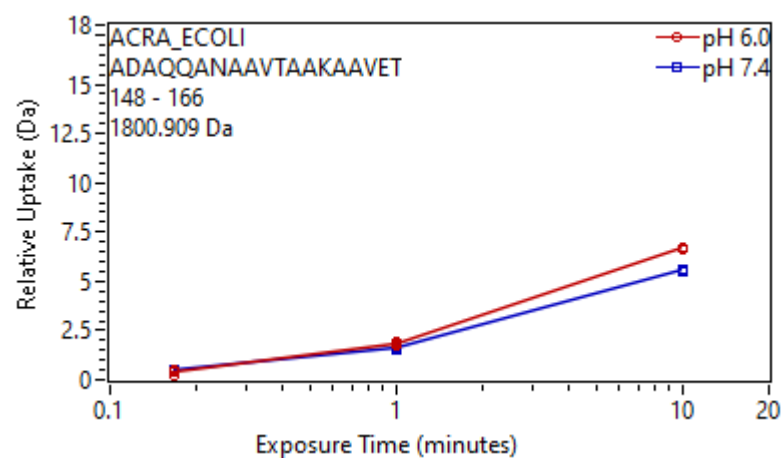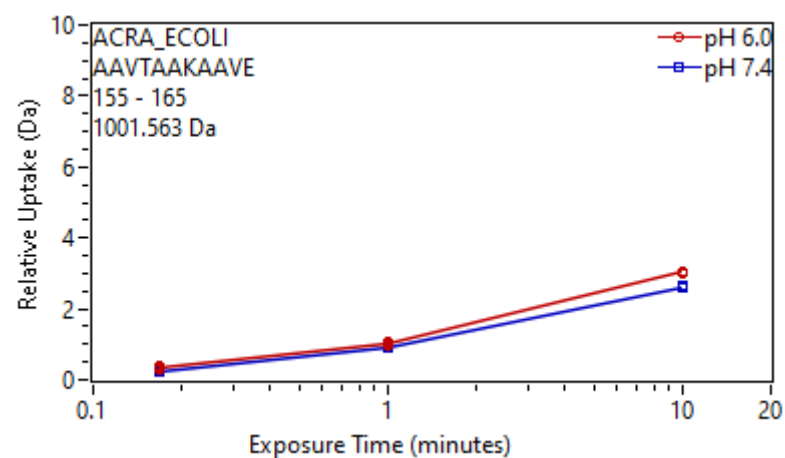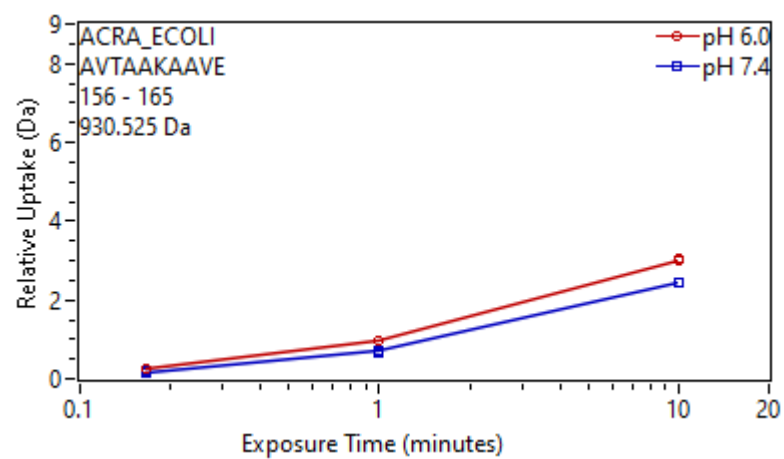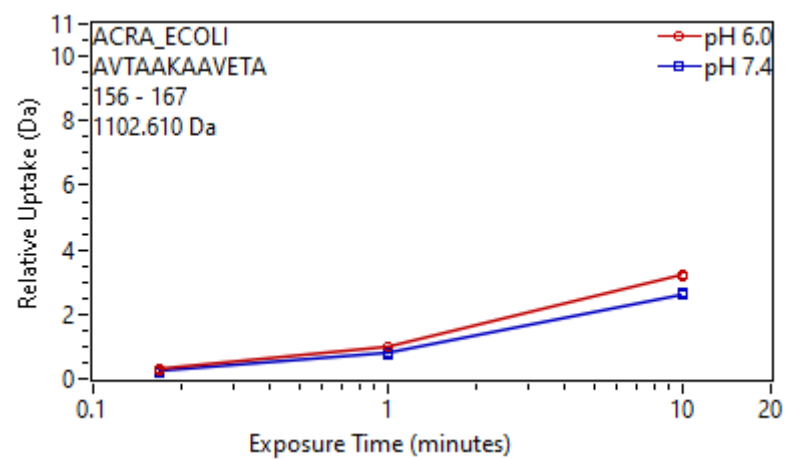

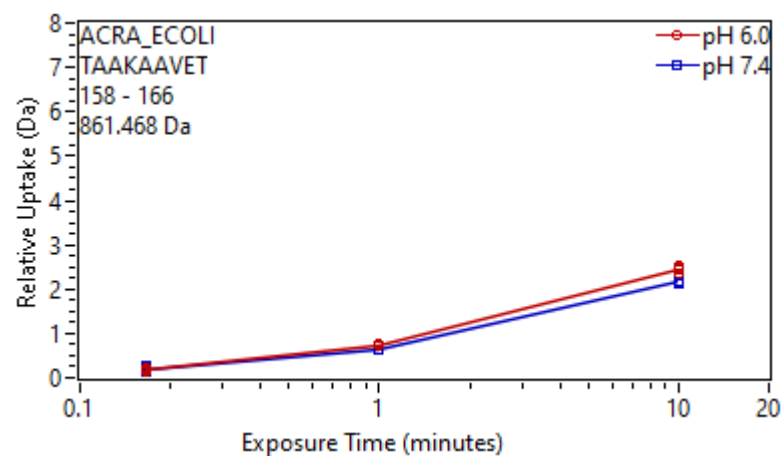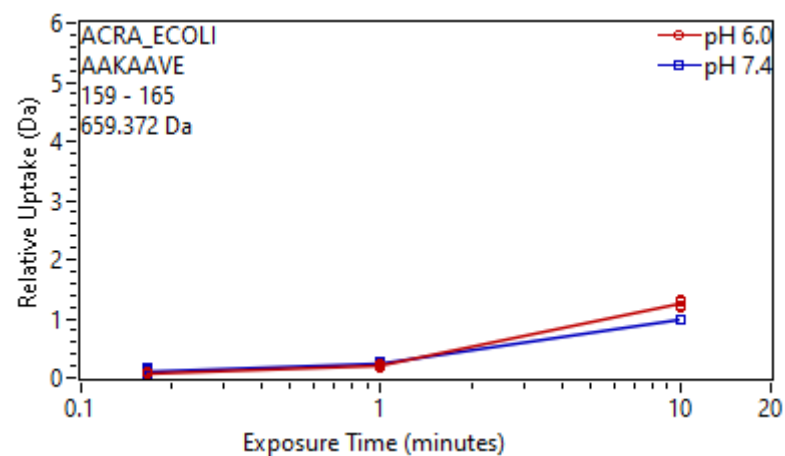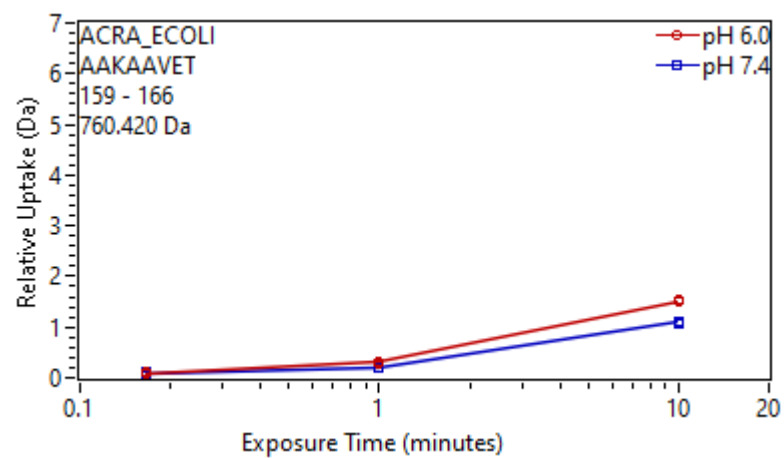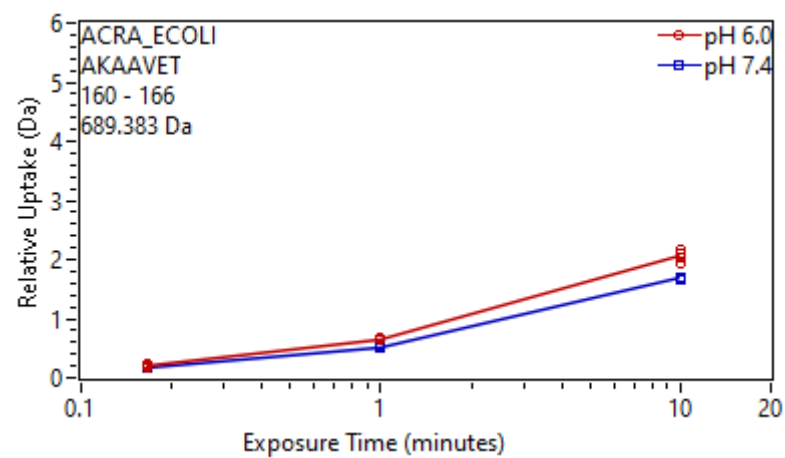

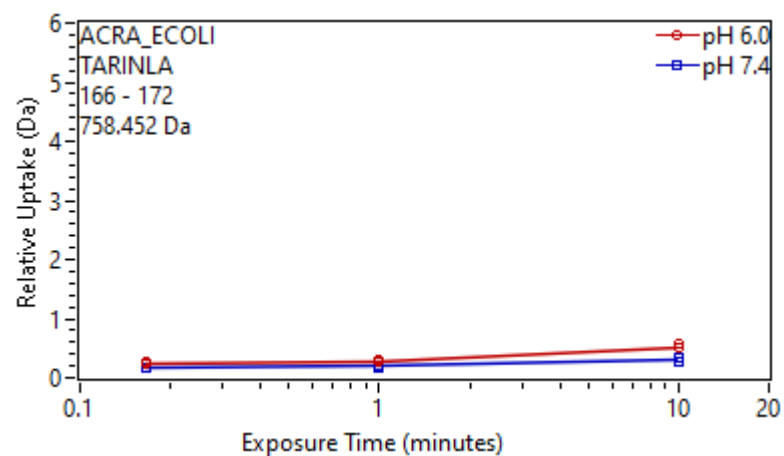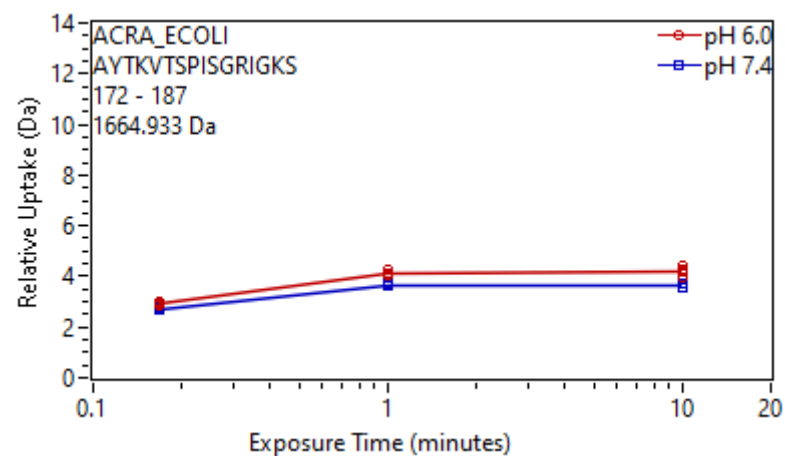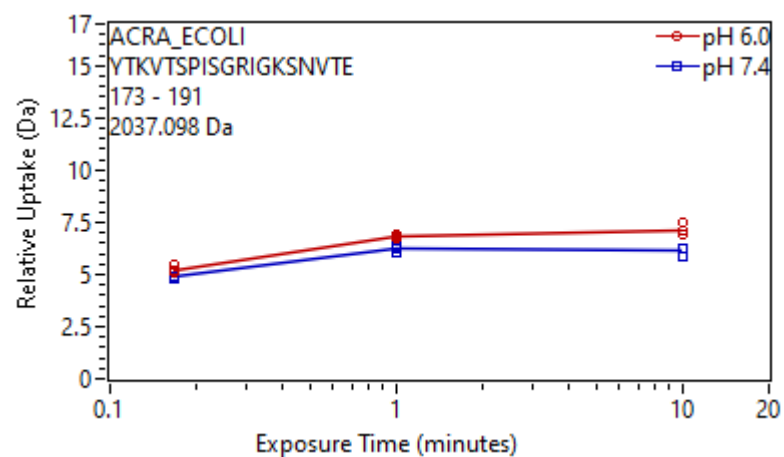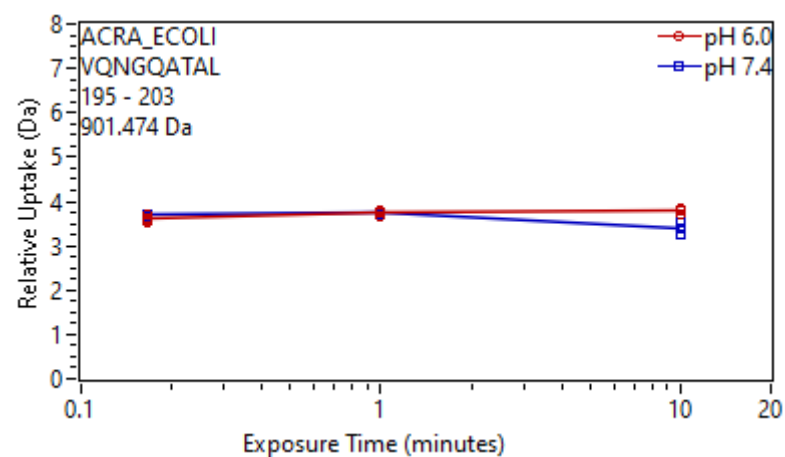

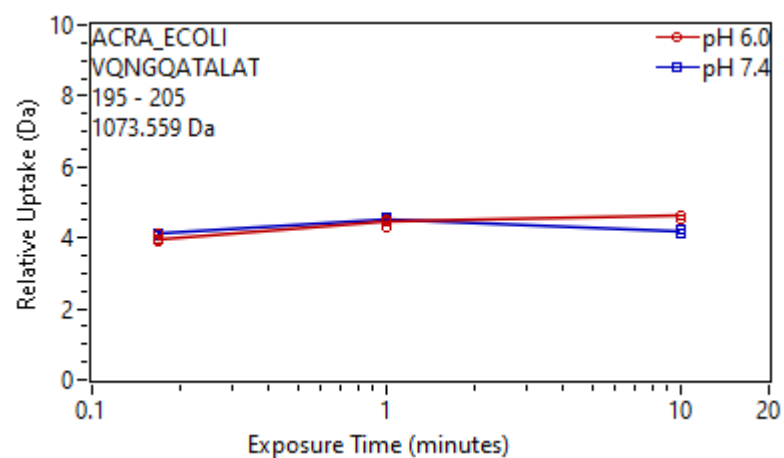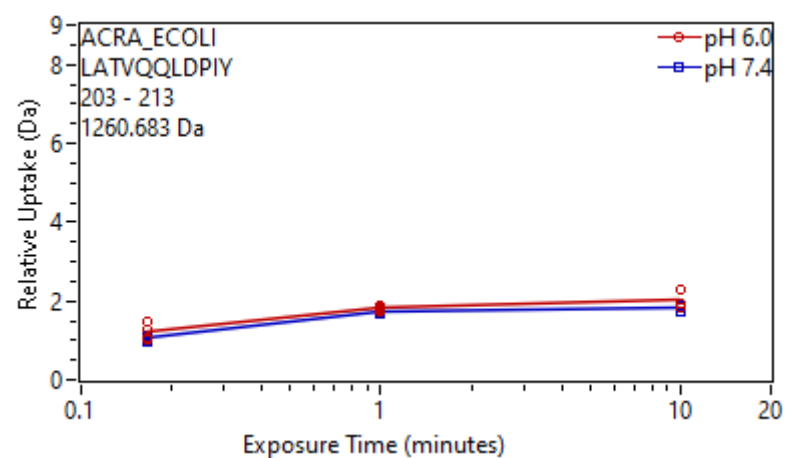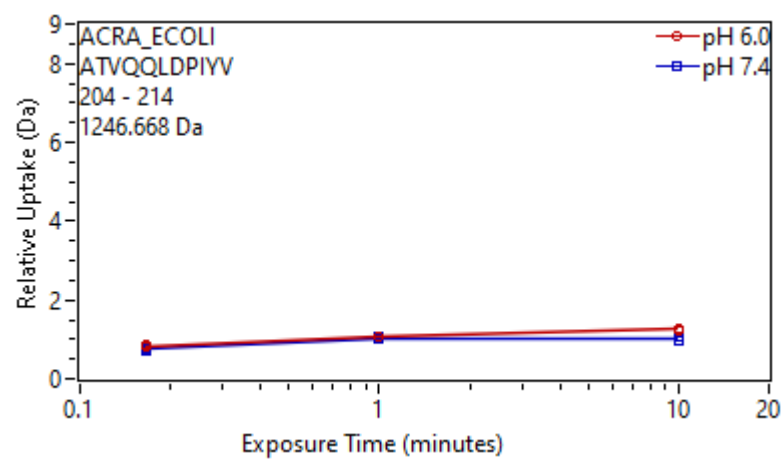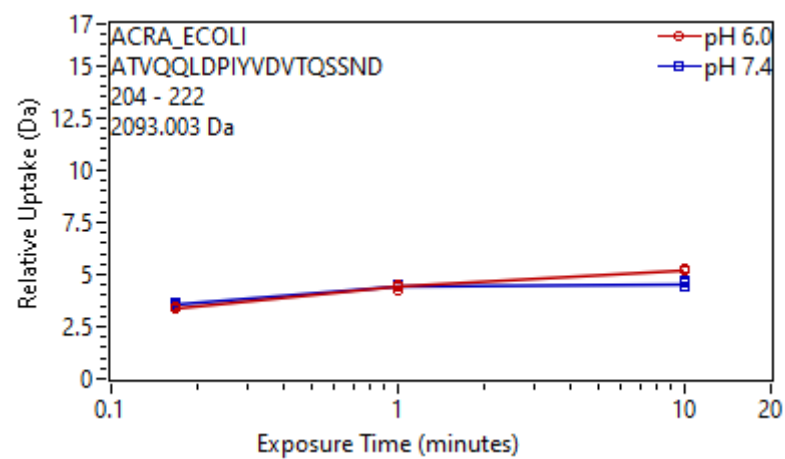

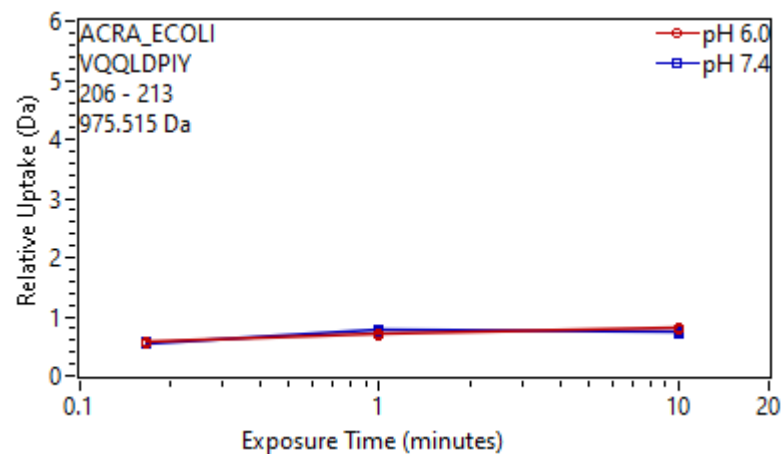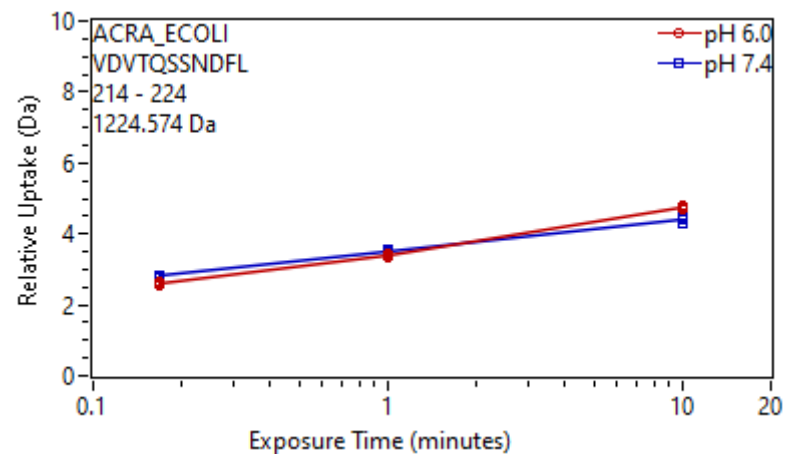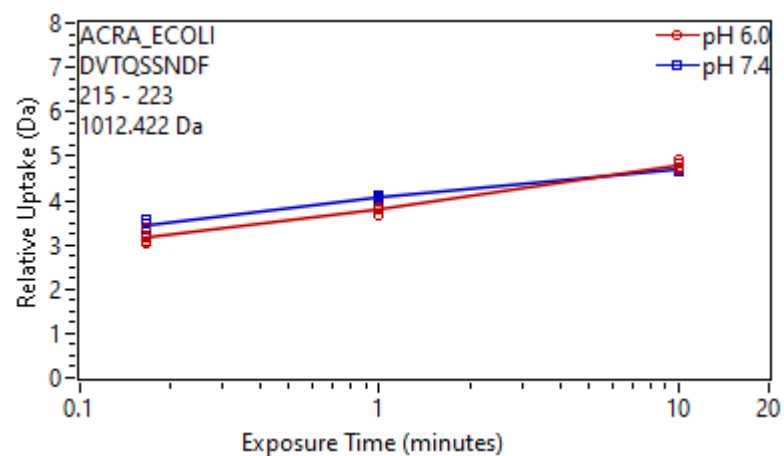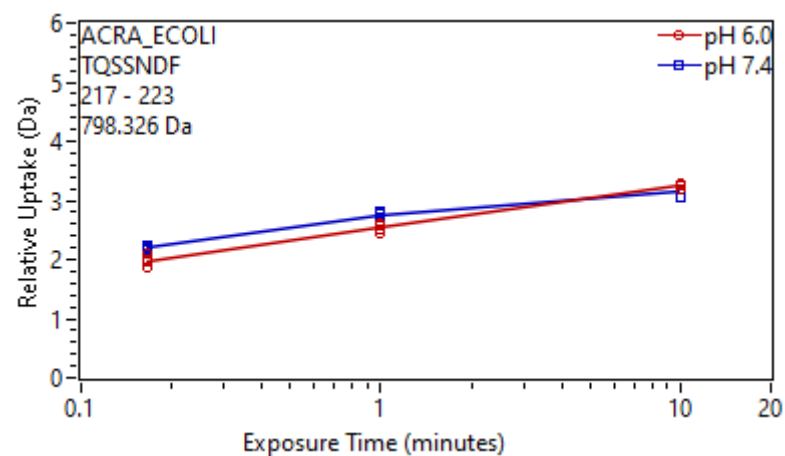

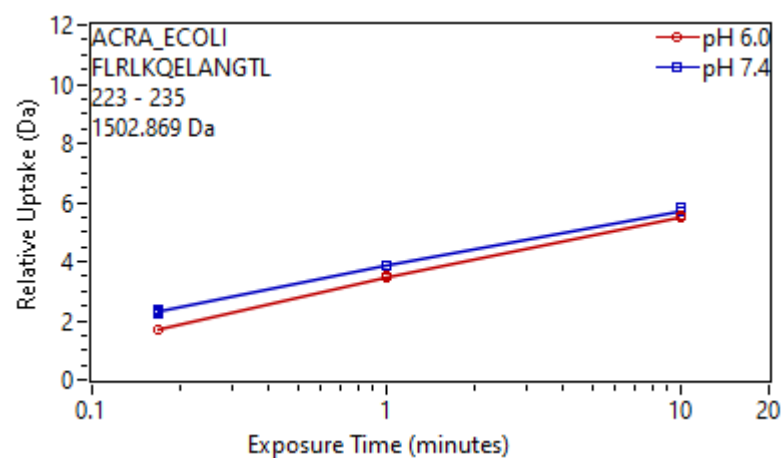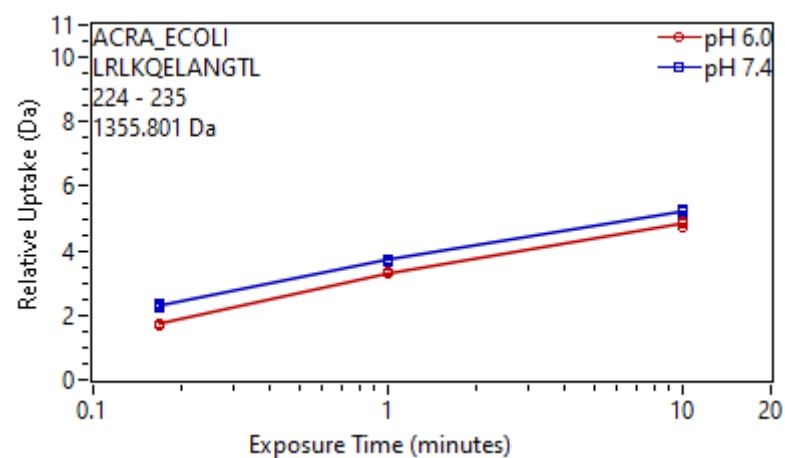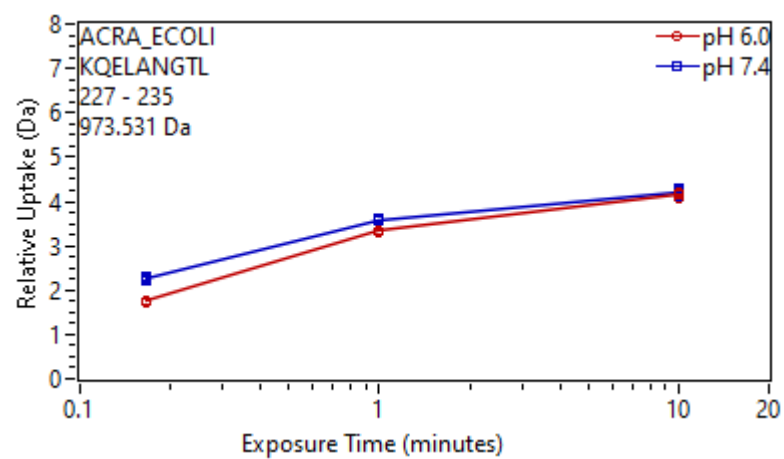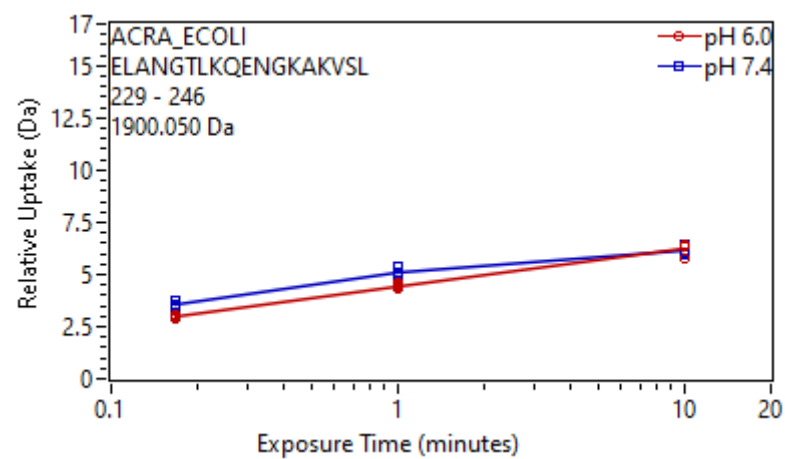

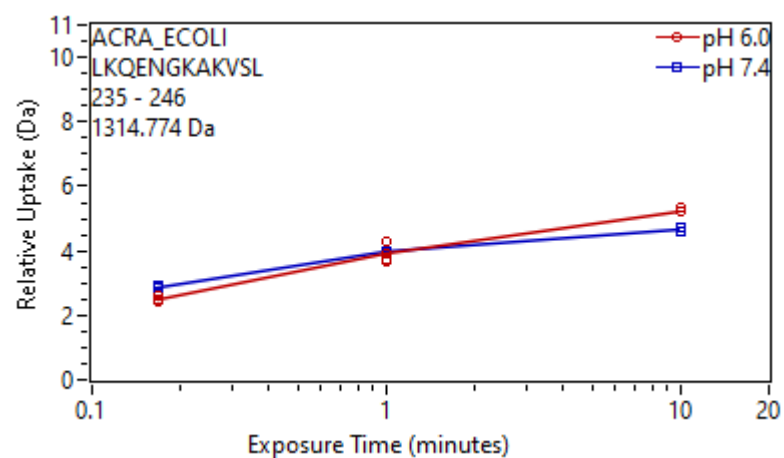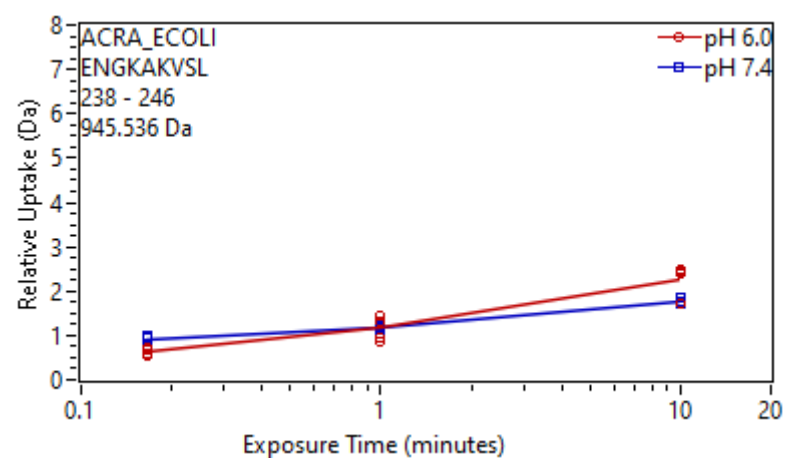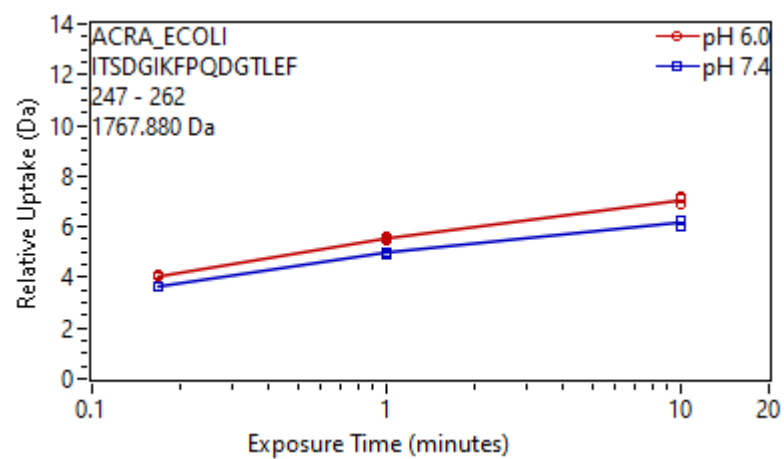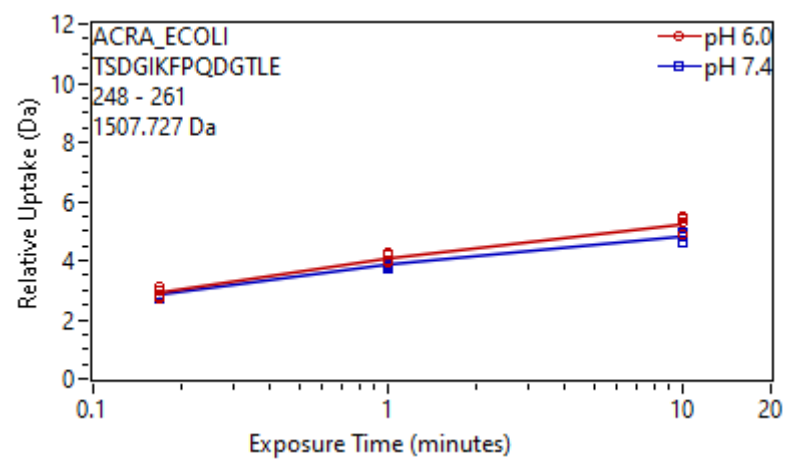

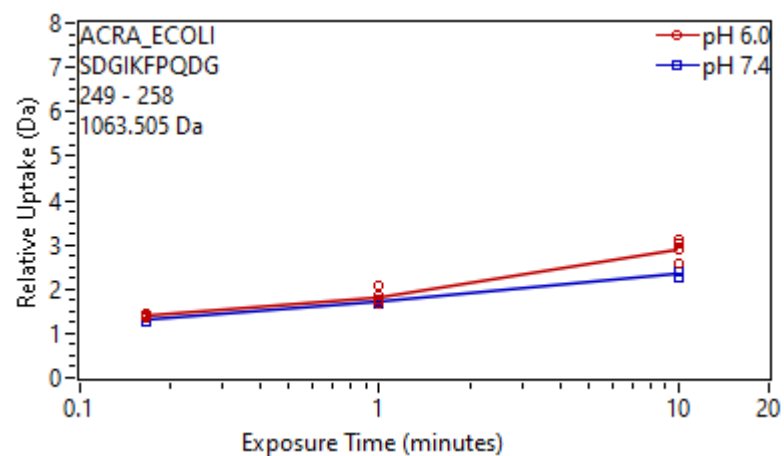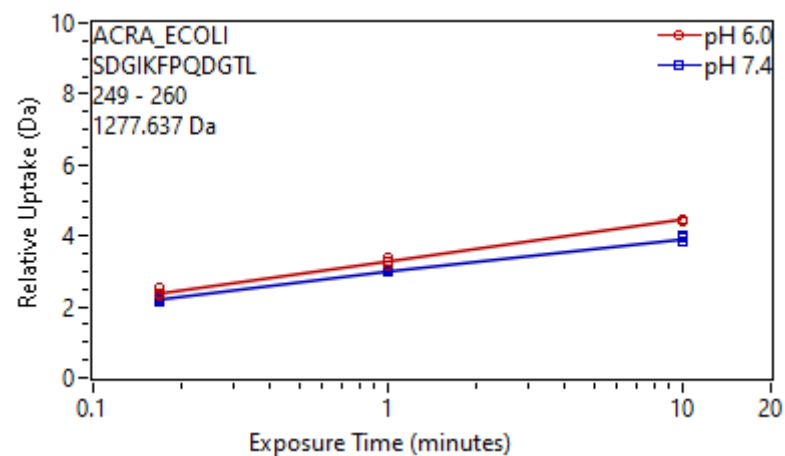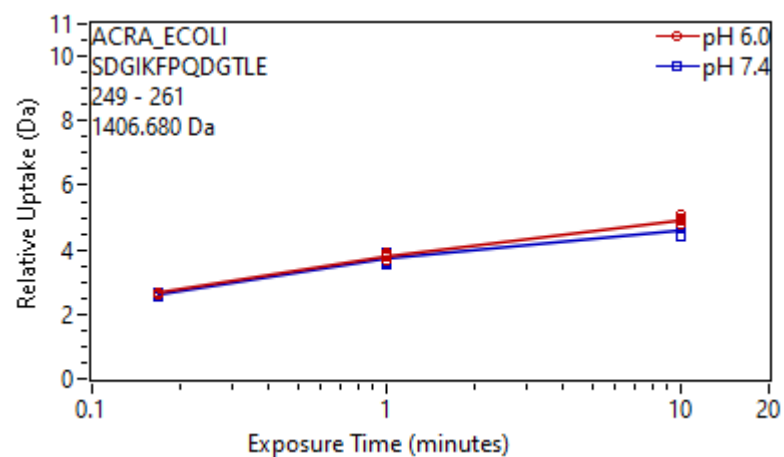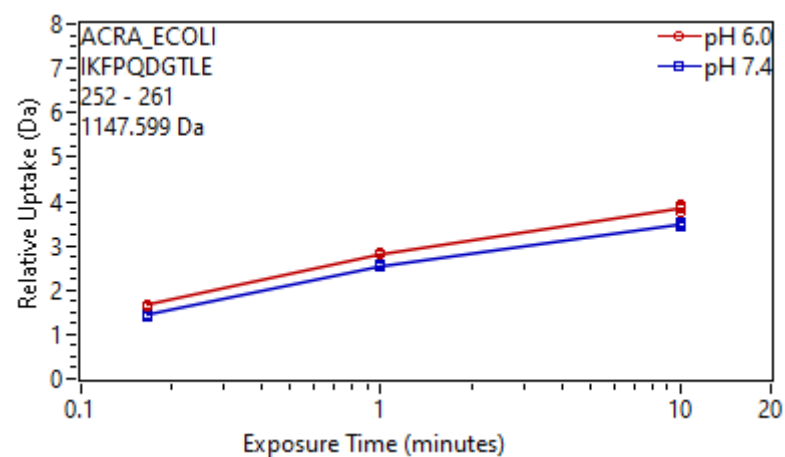

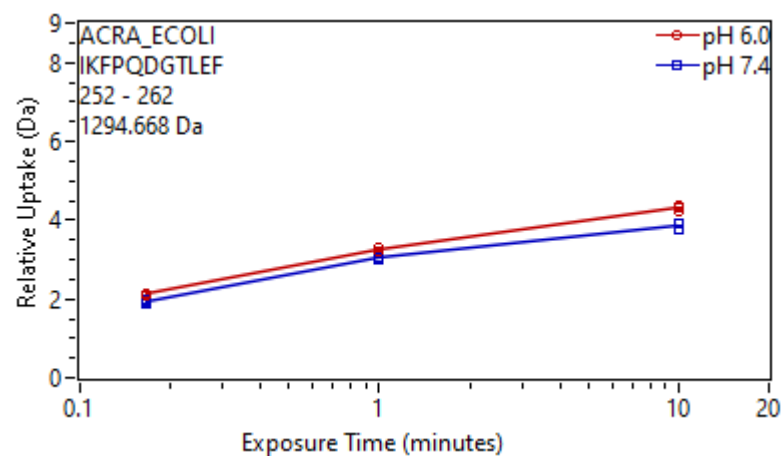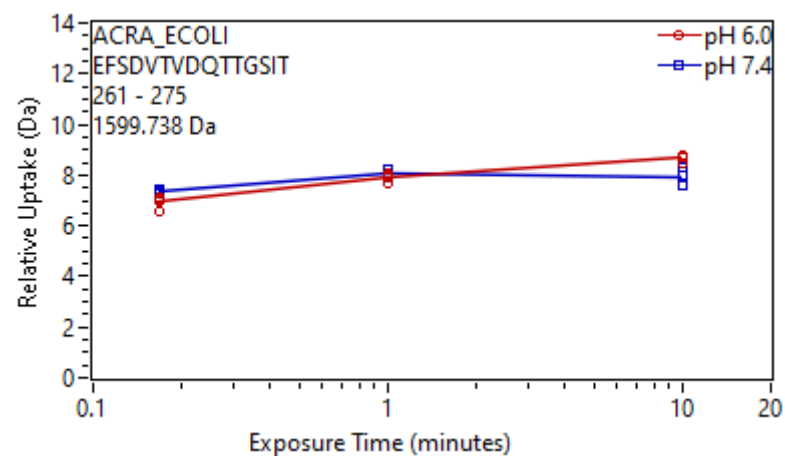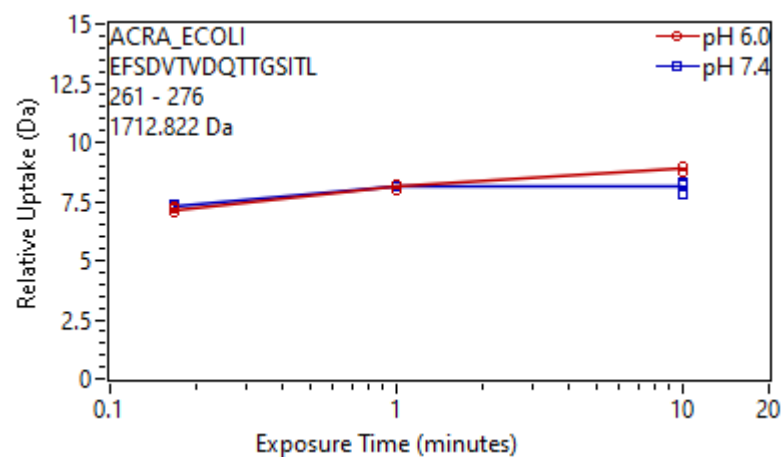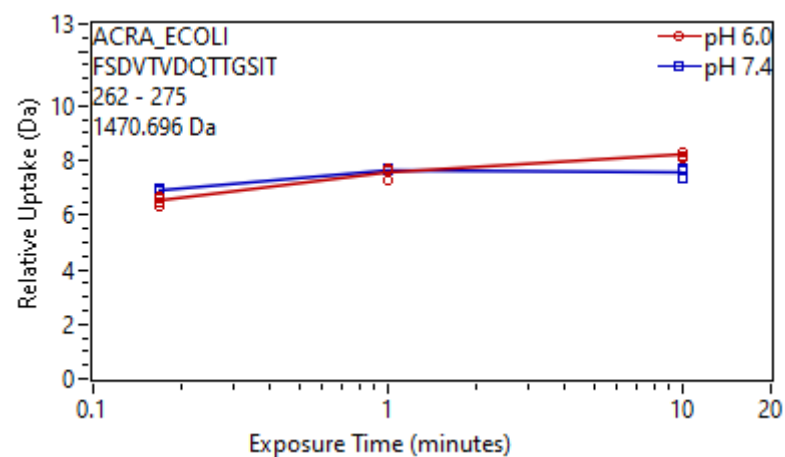

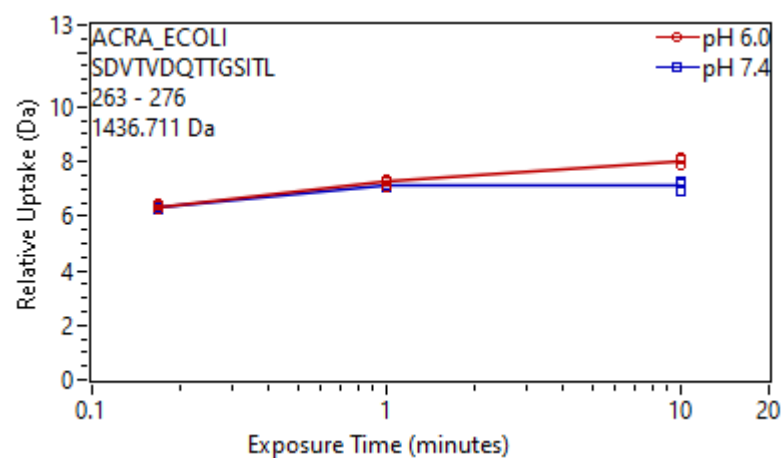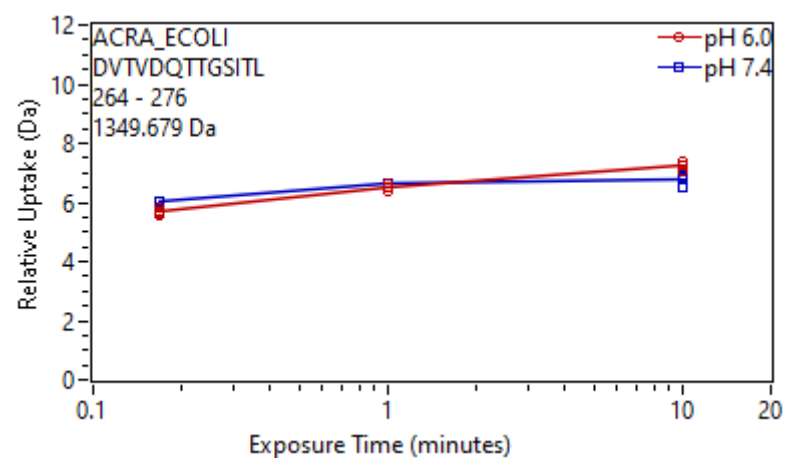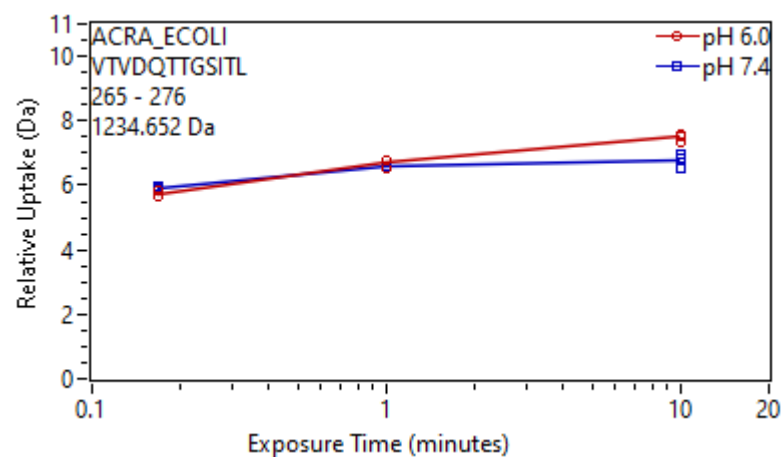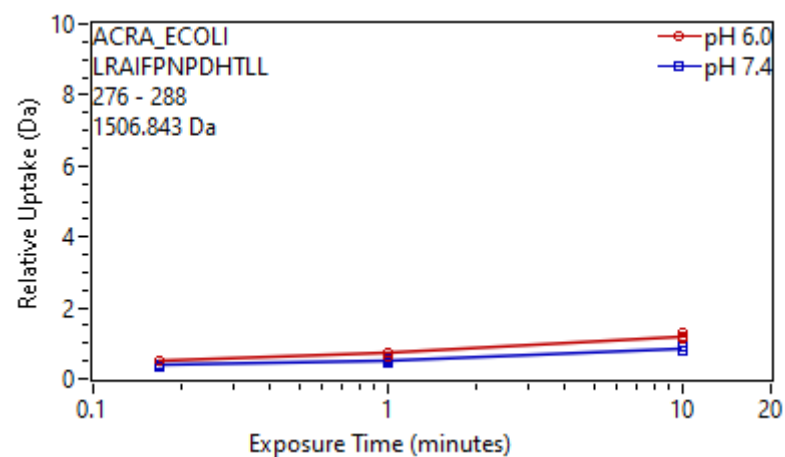

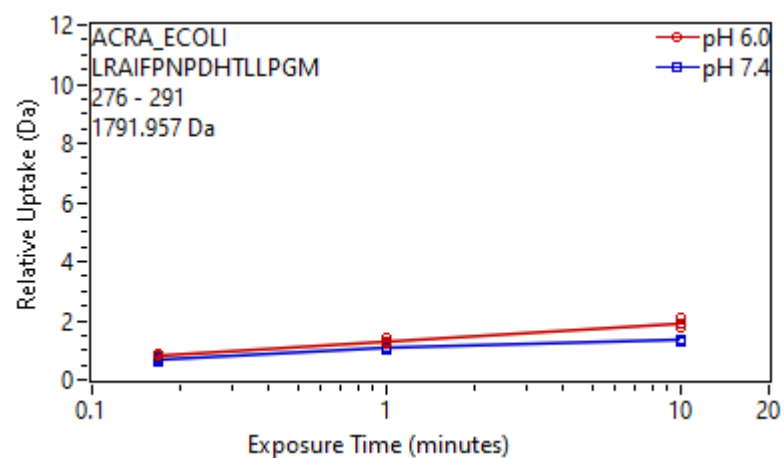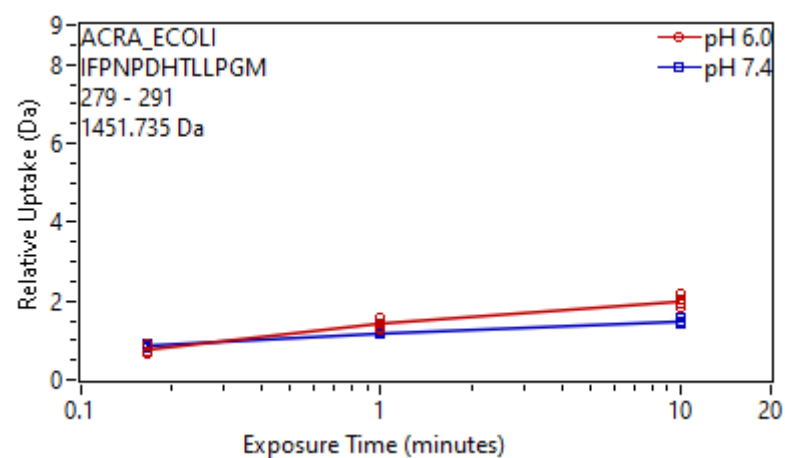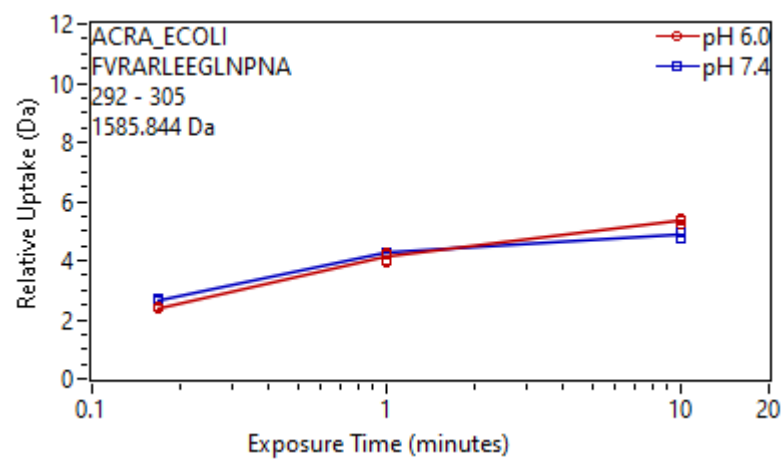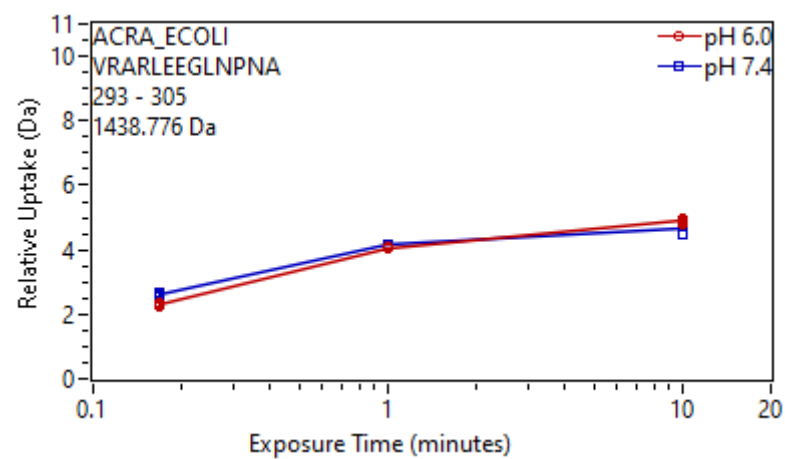

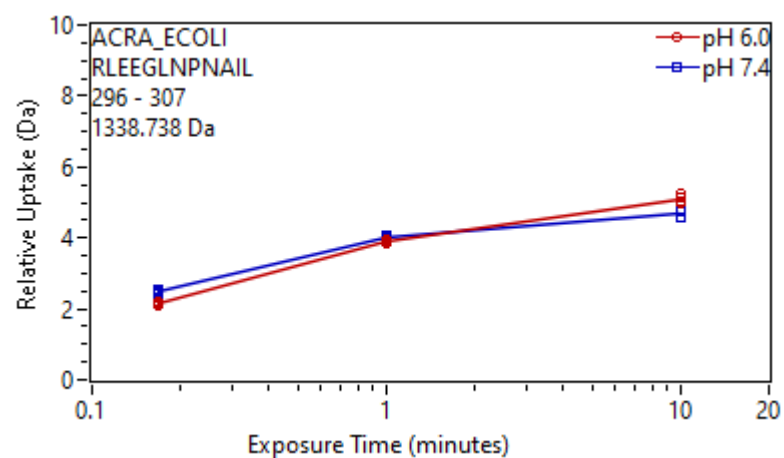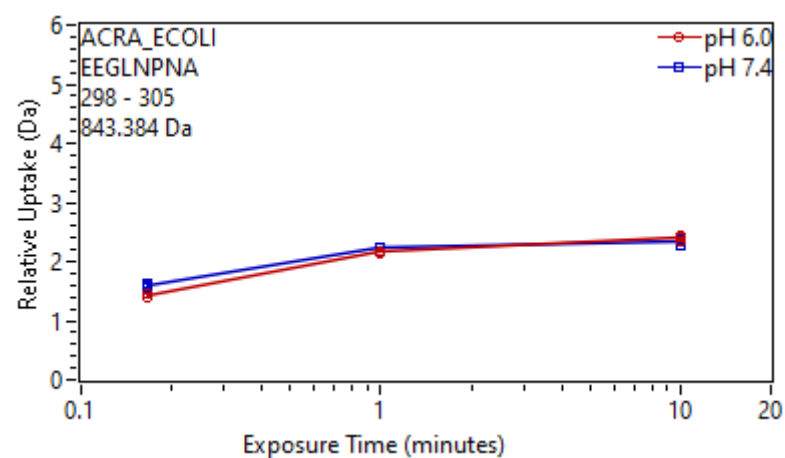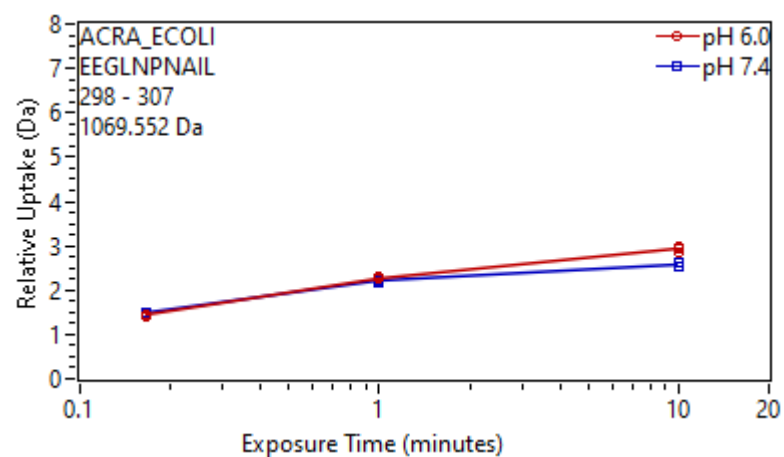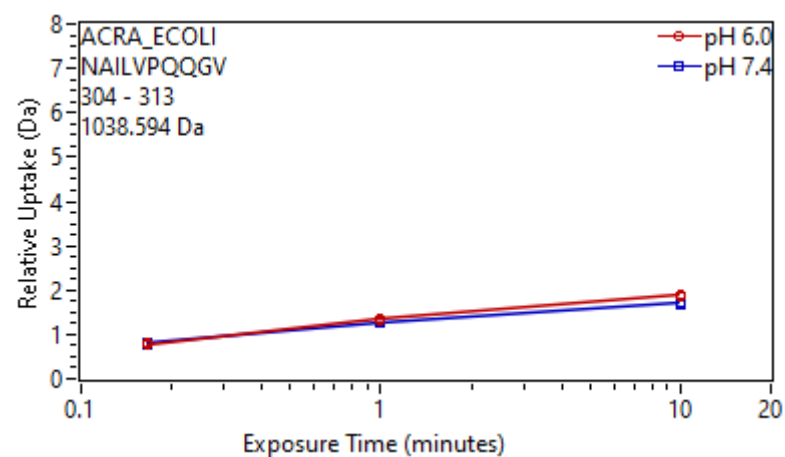

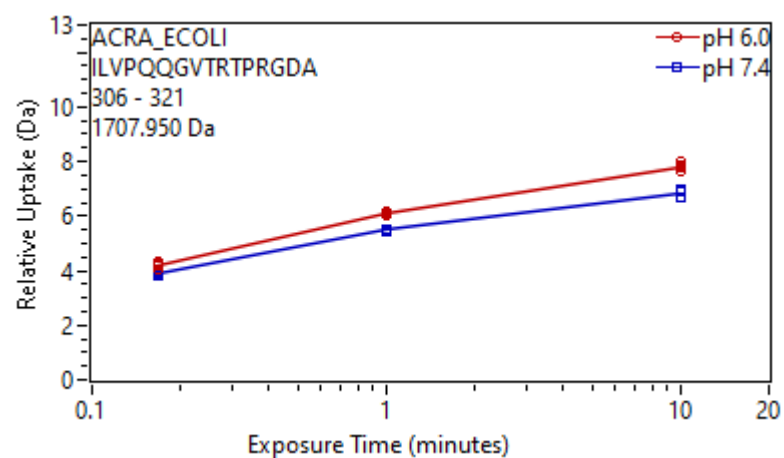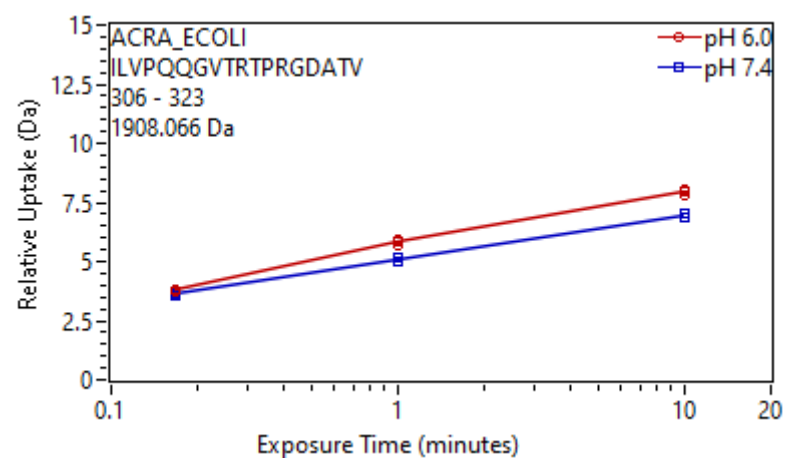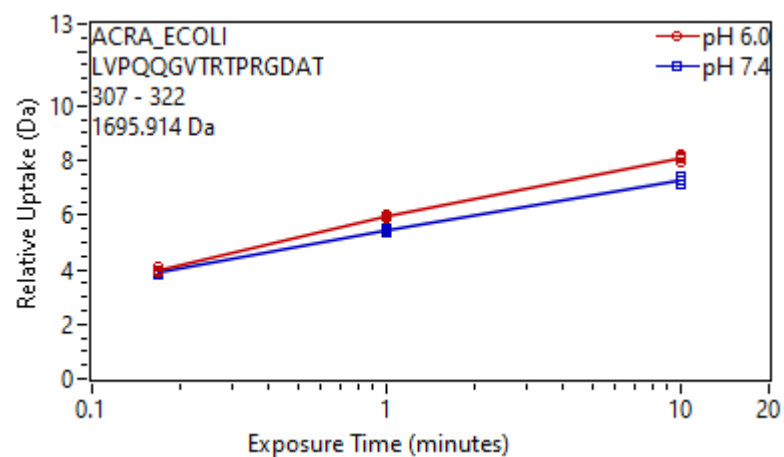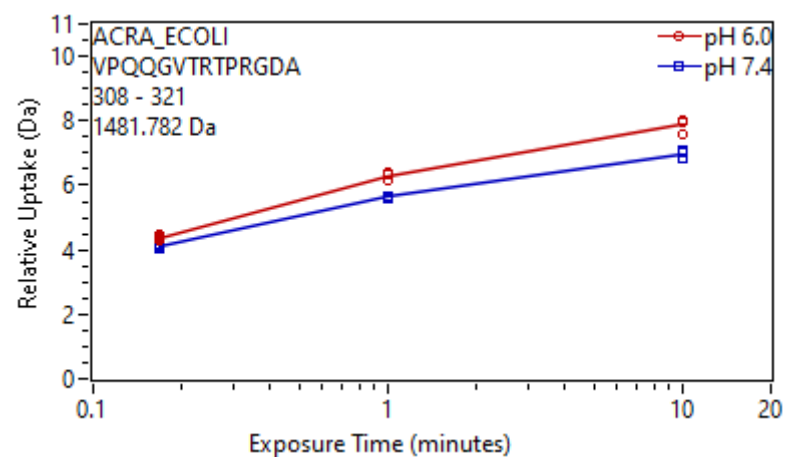

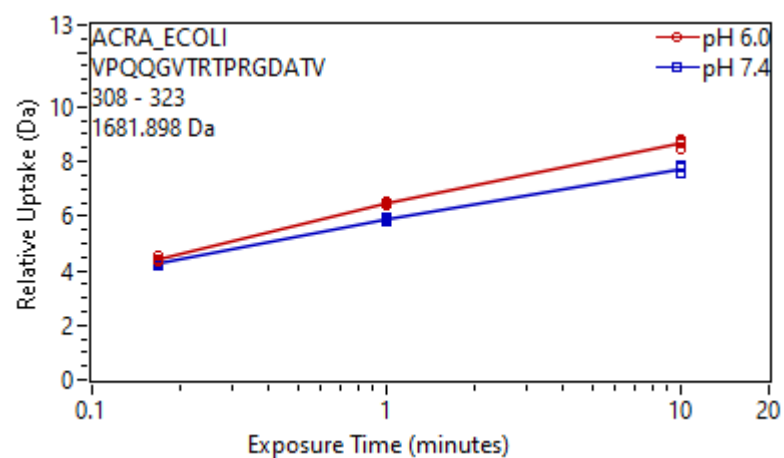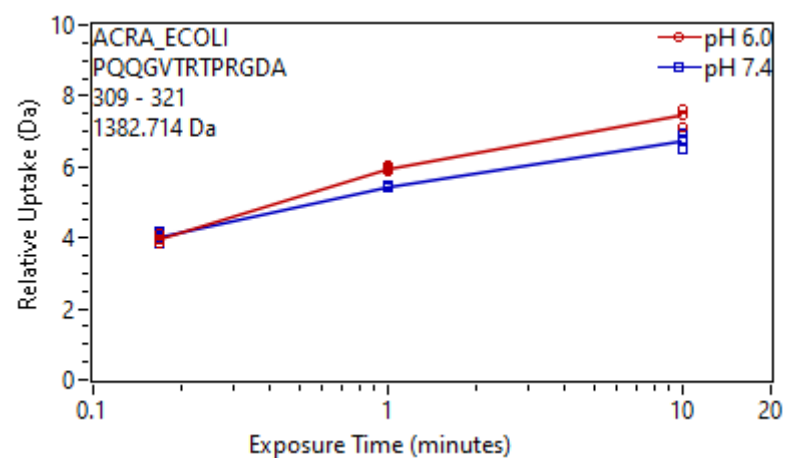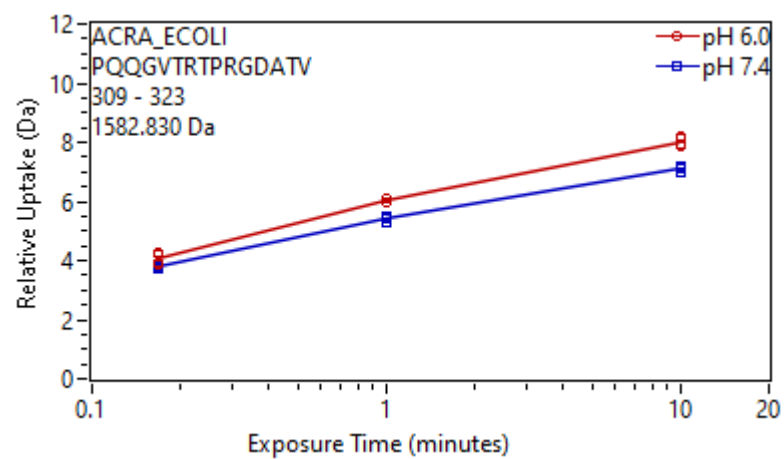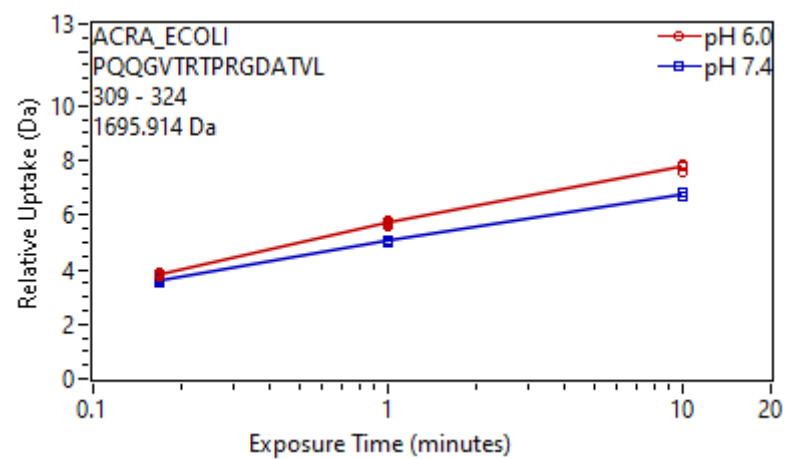

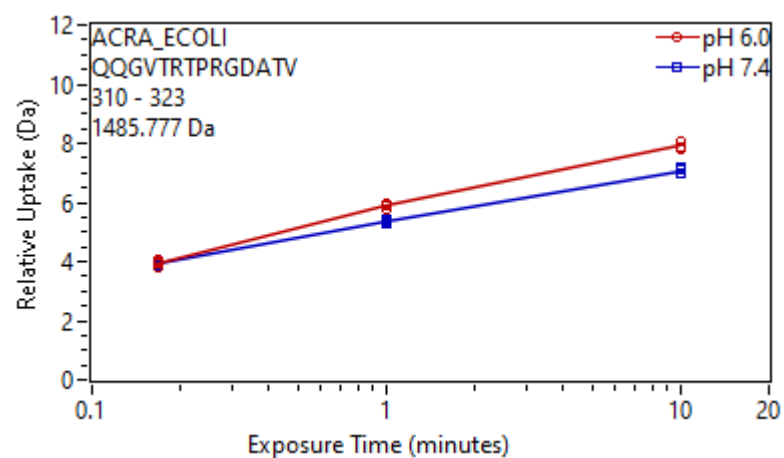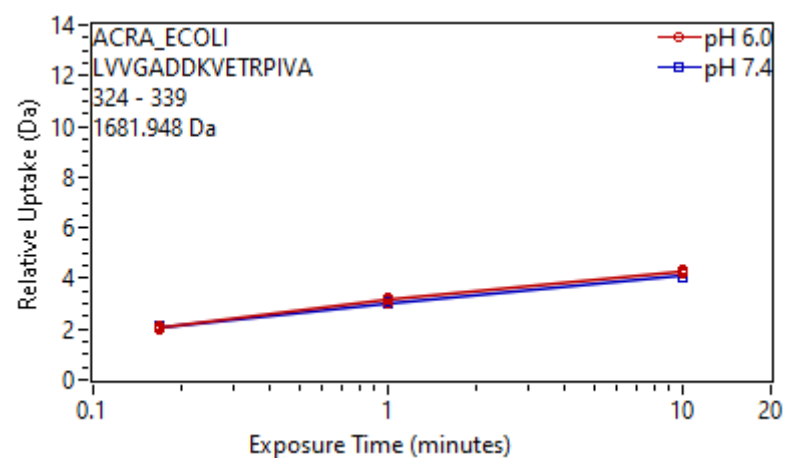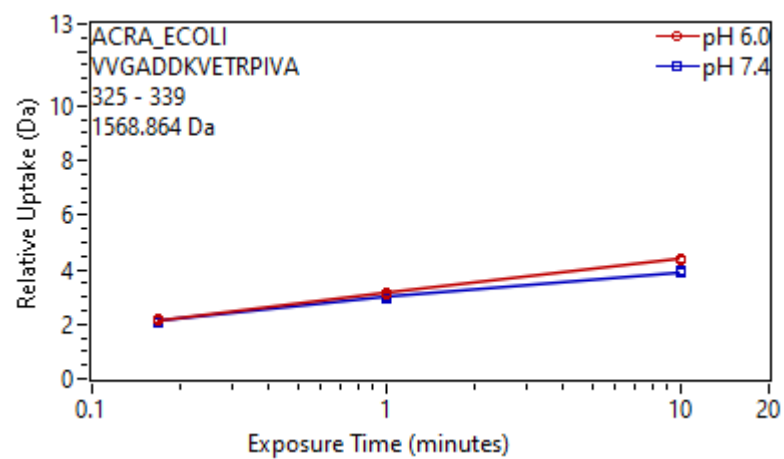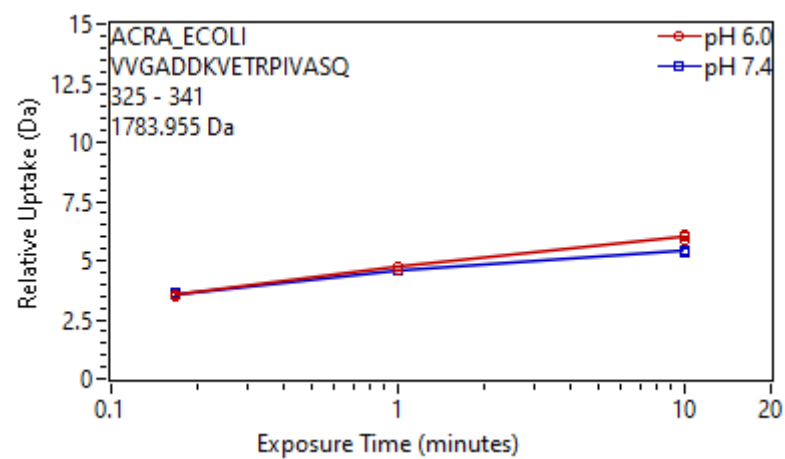

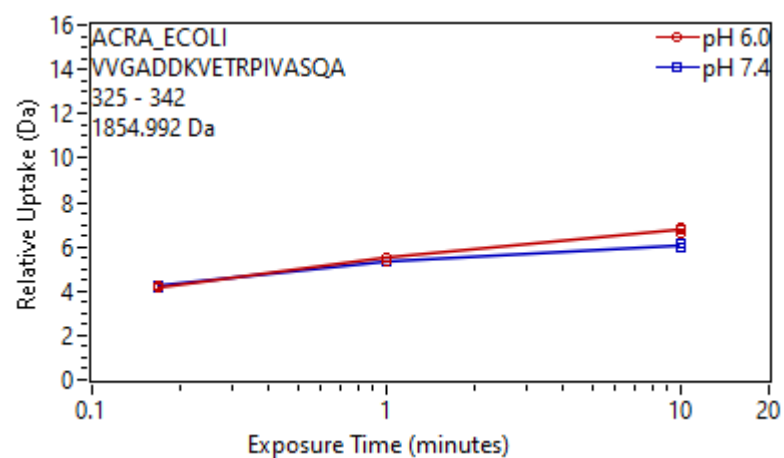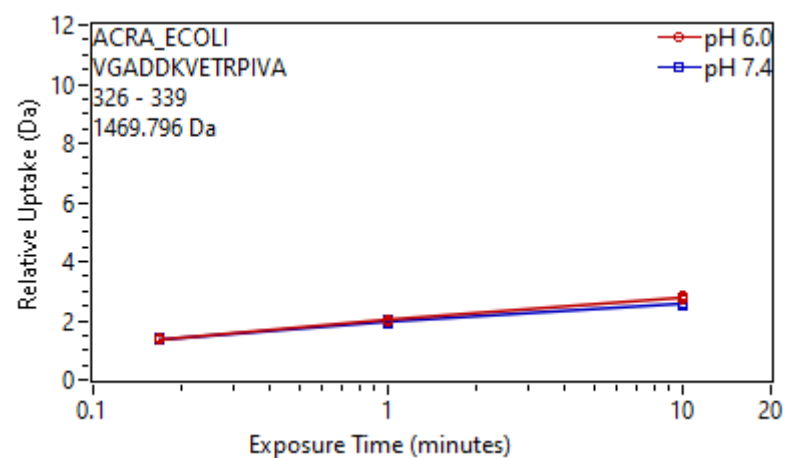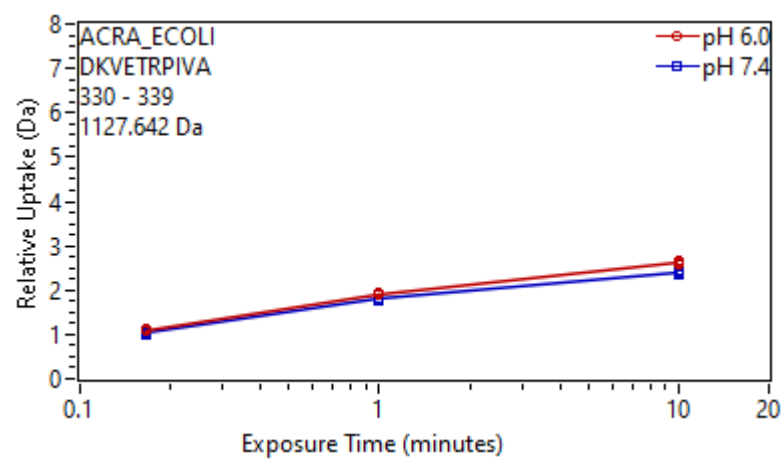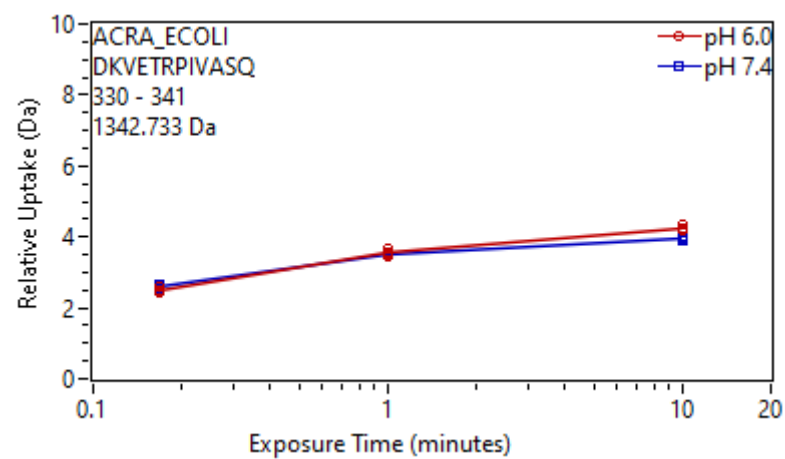

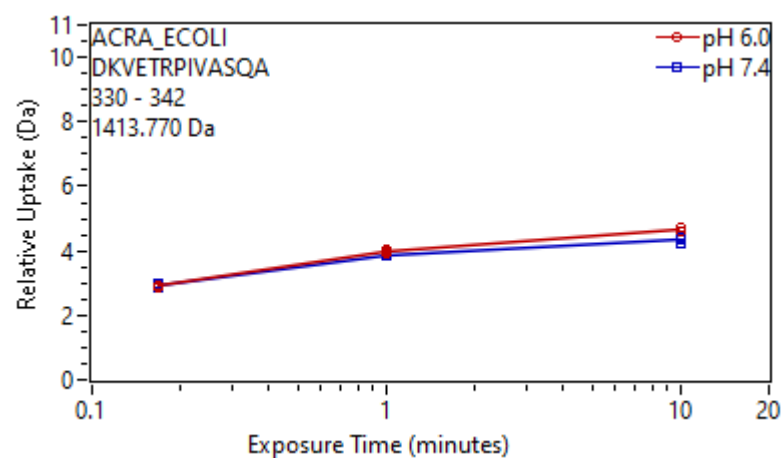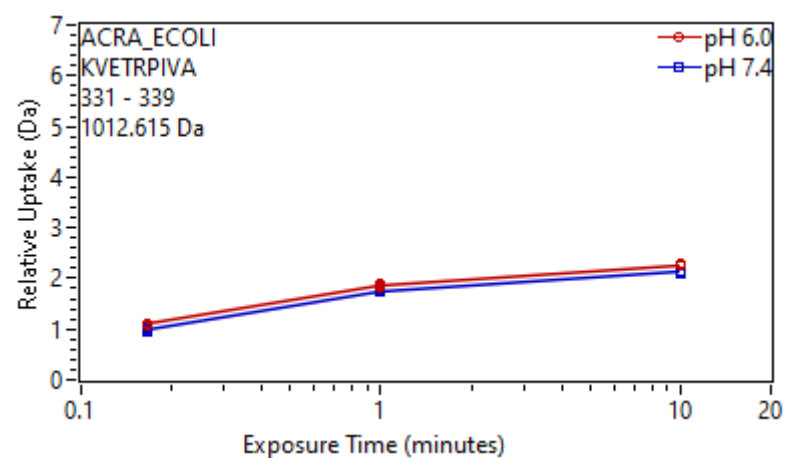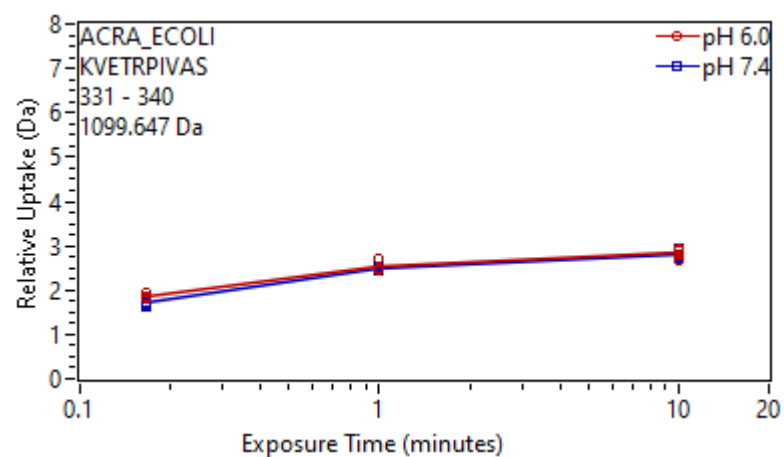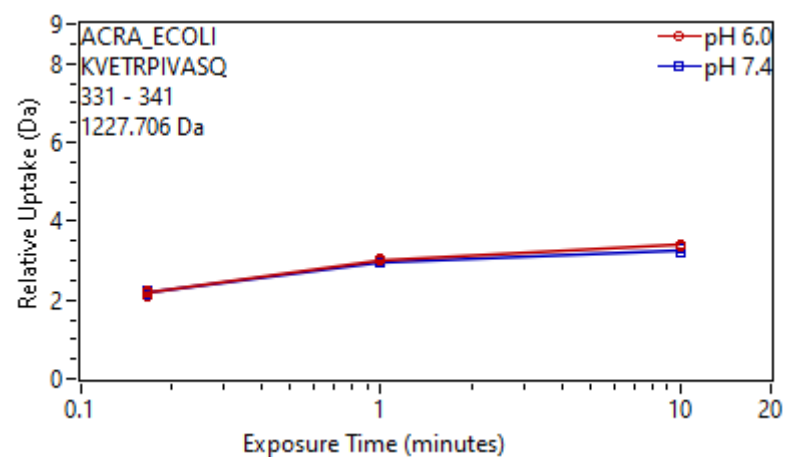

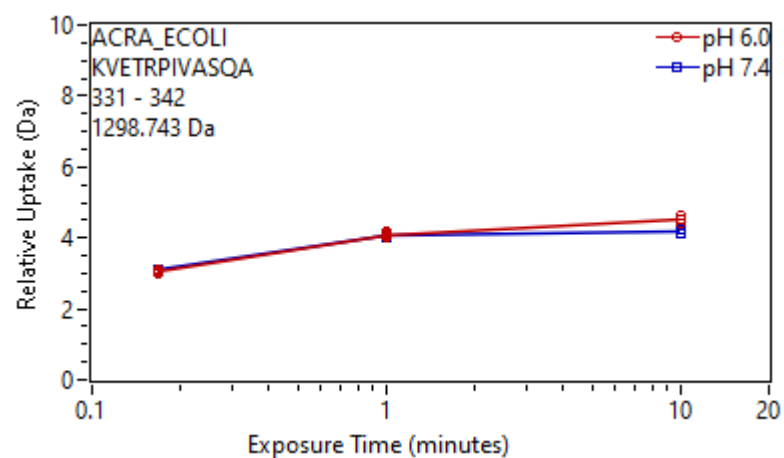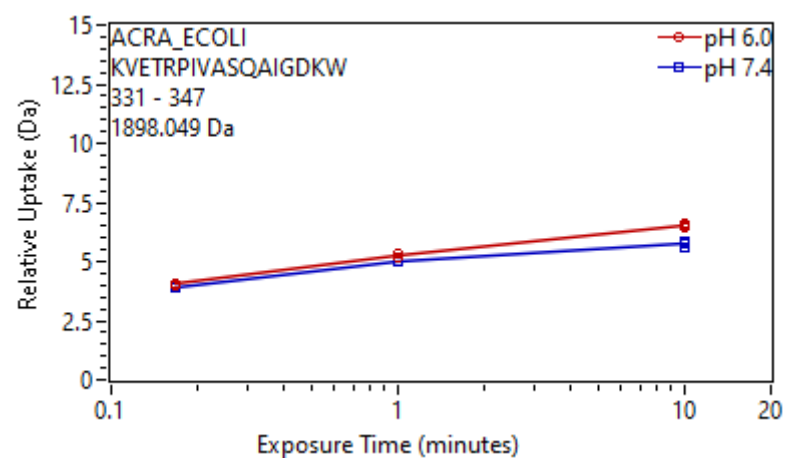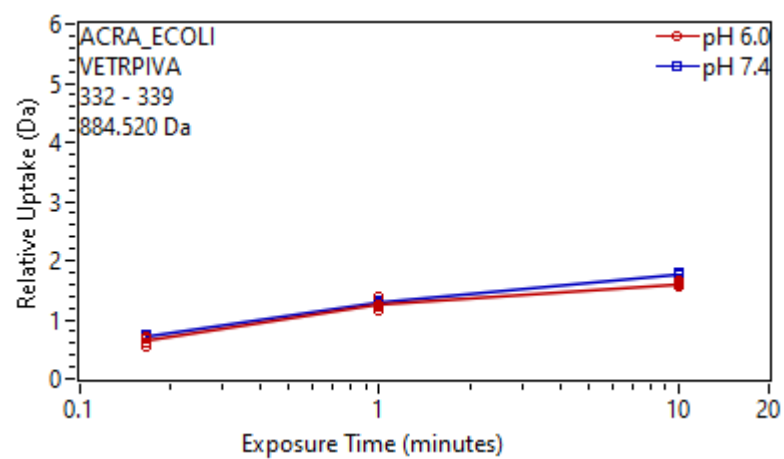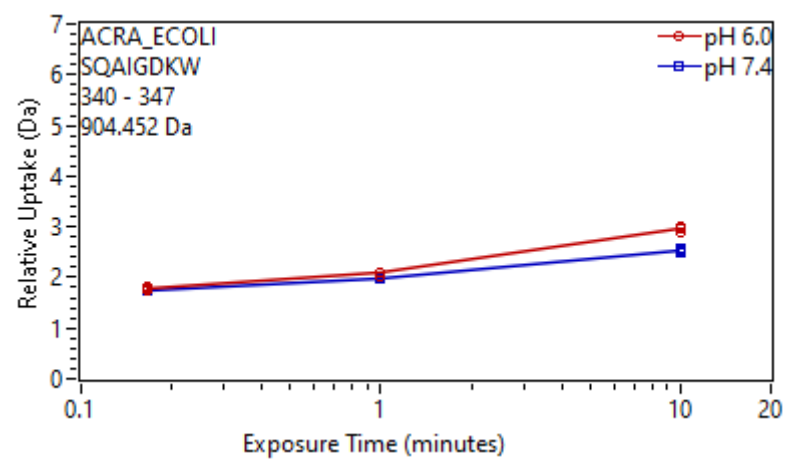

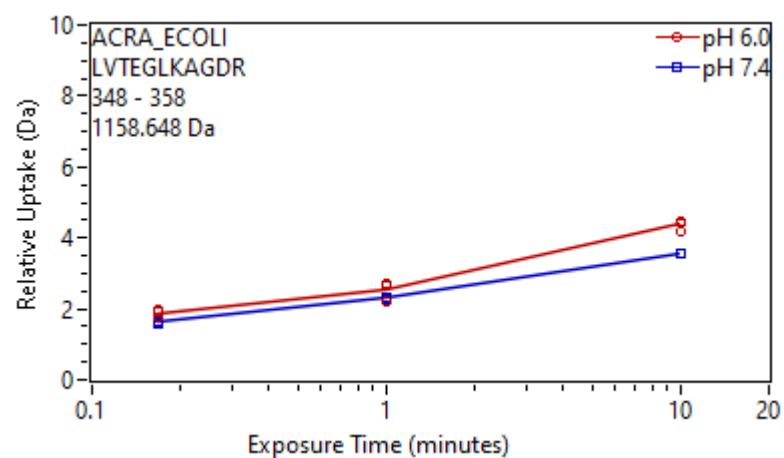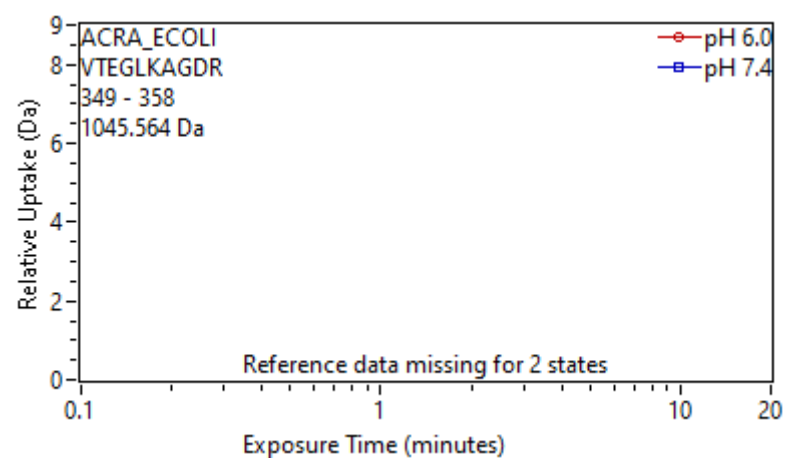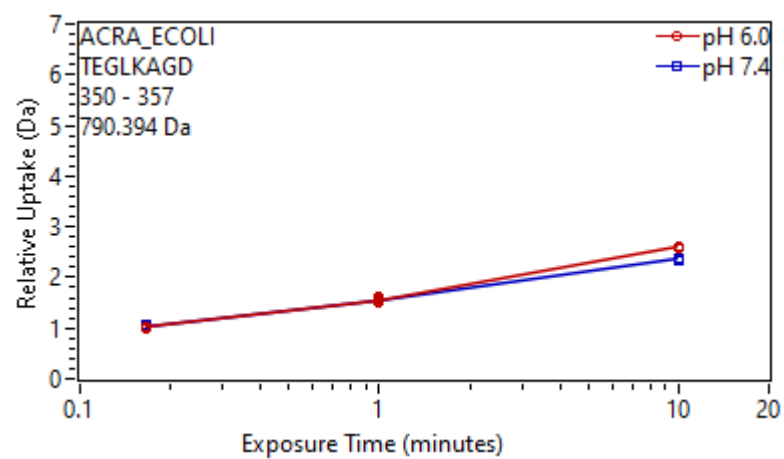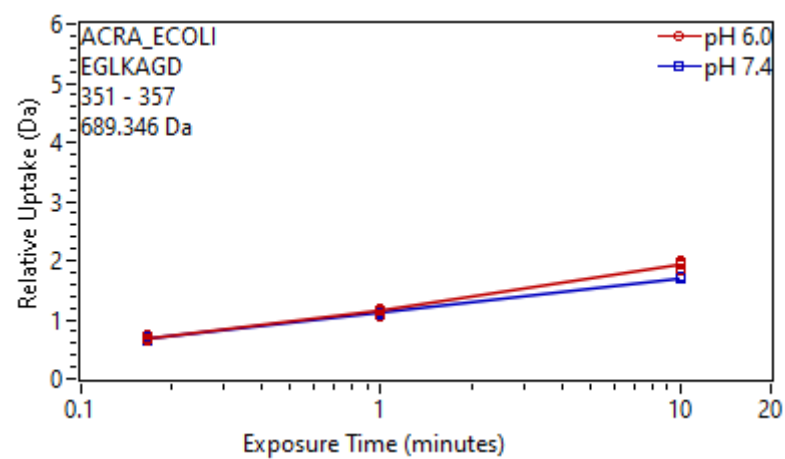

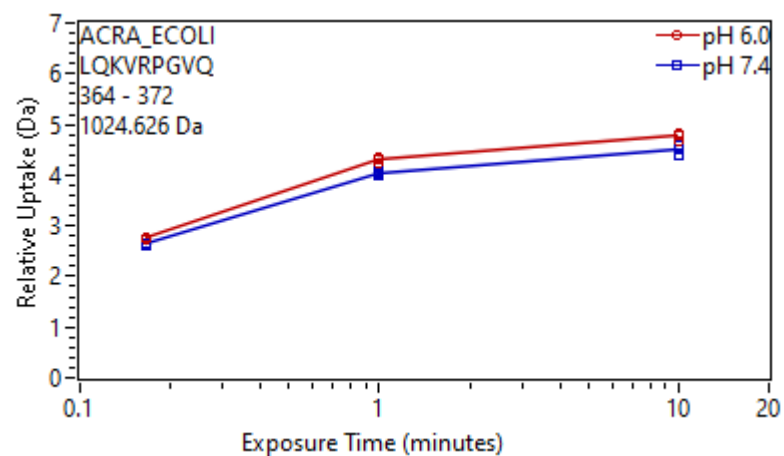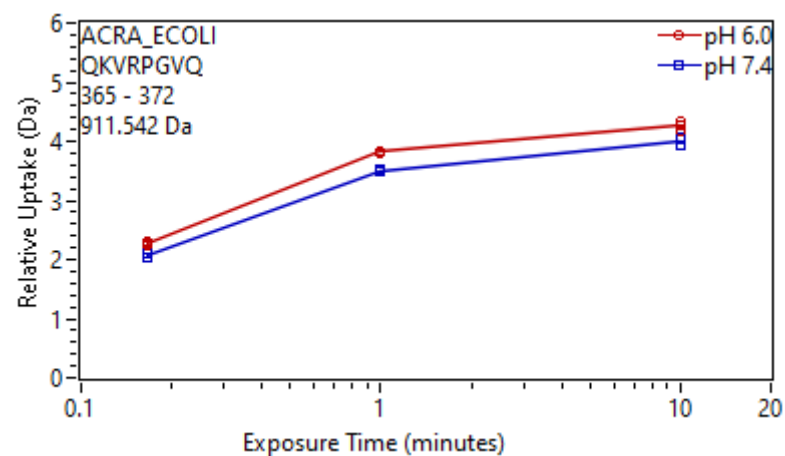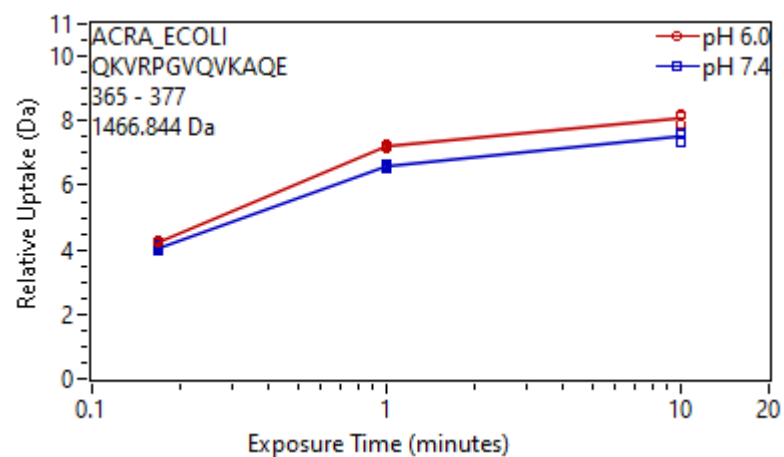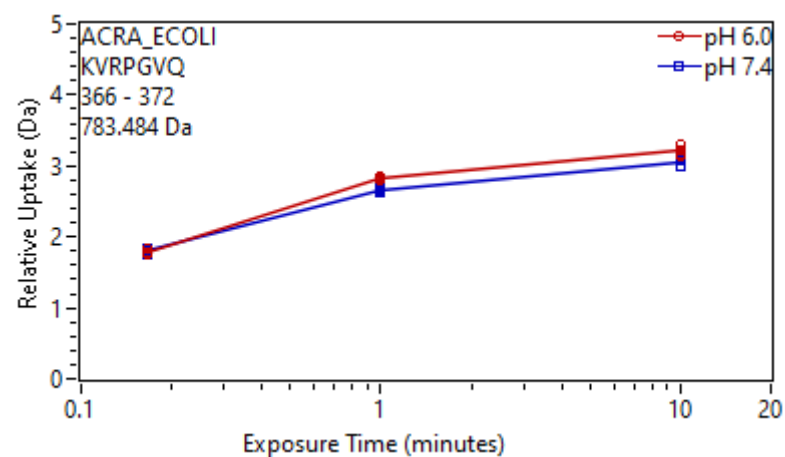

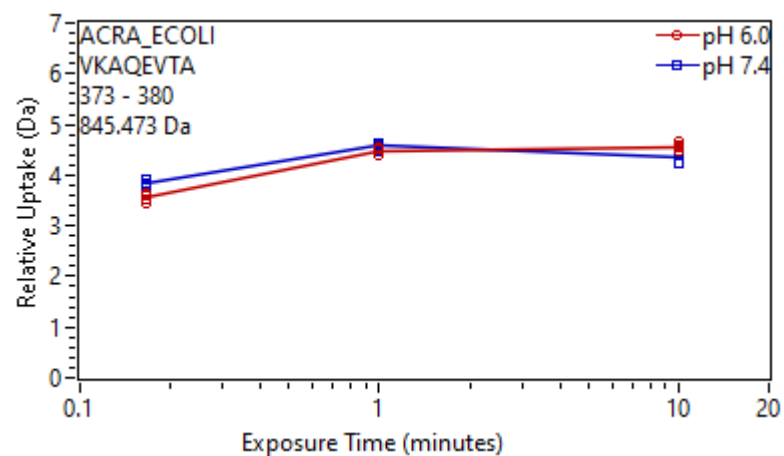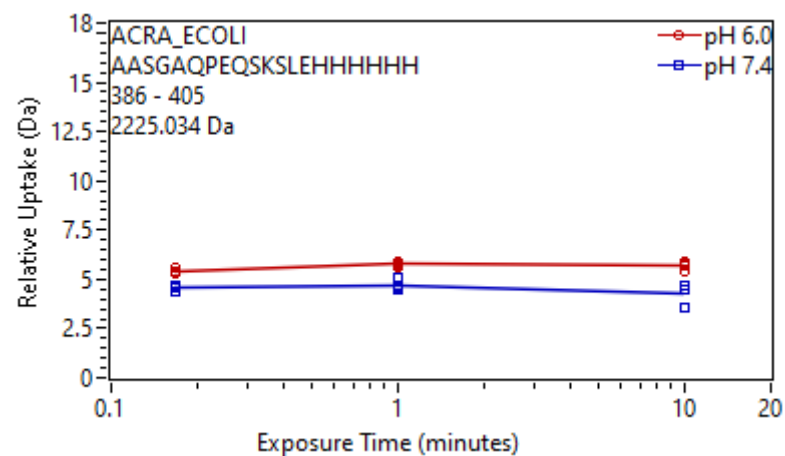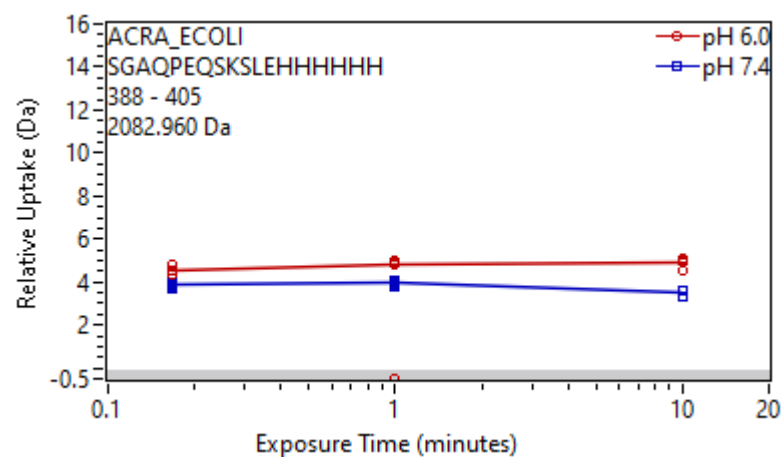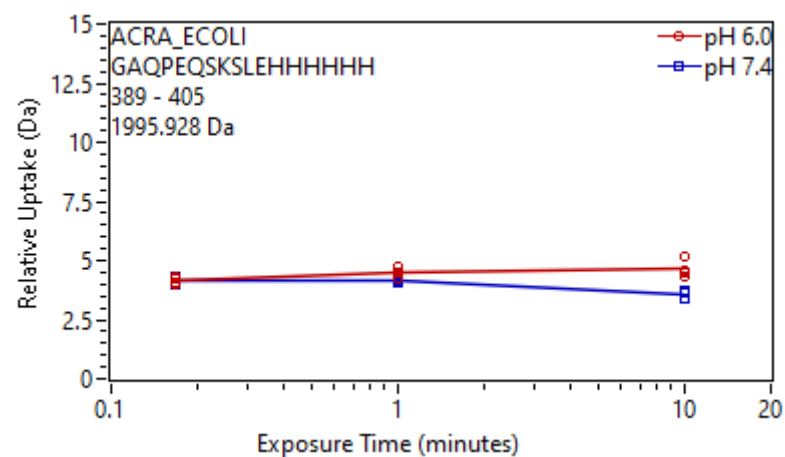

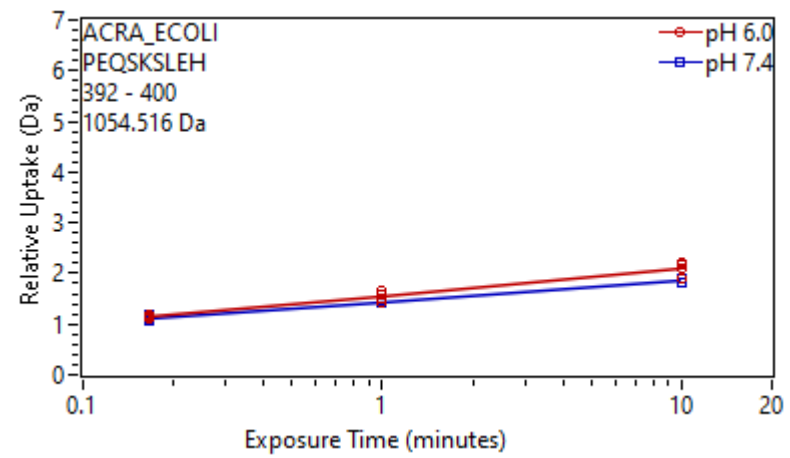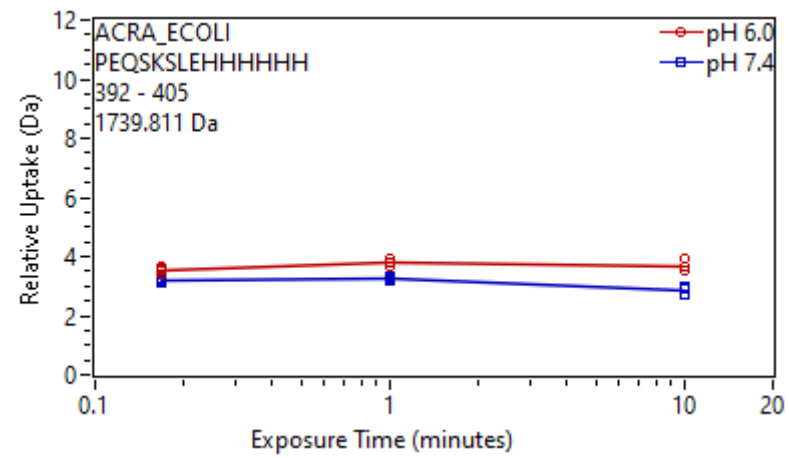

Supplement: Supplement 4 [file media-4.zip › Supplementary Data 3/Uptake plots 1.pdf]

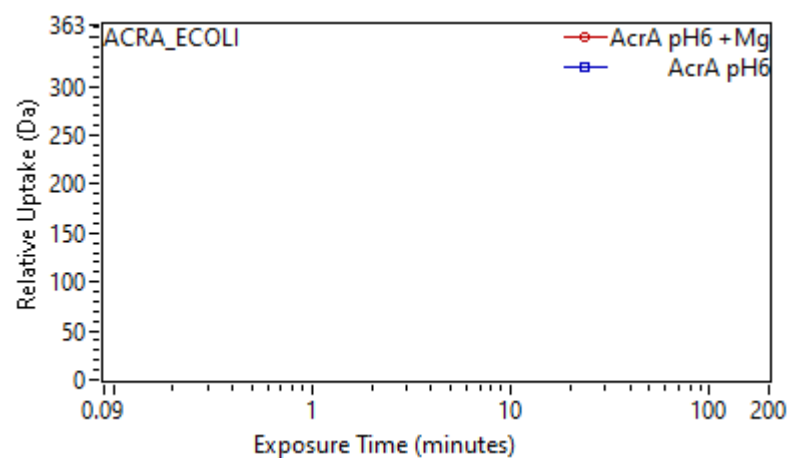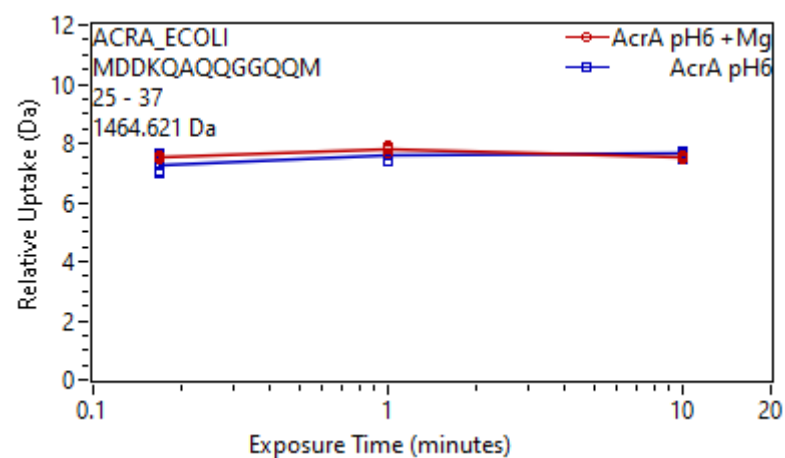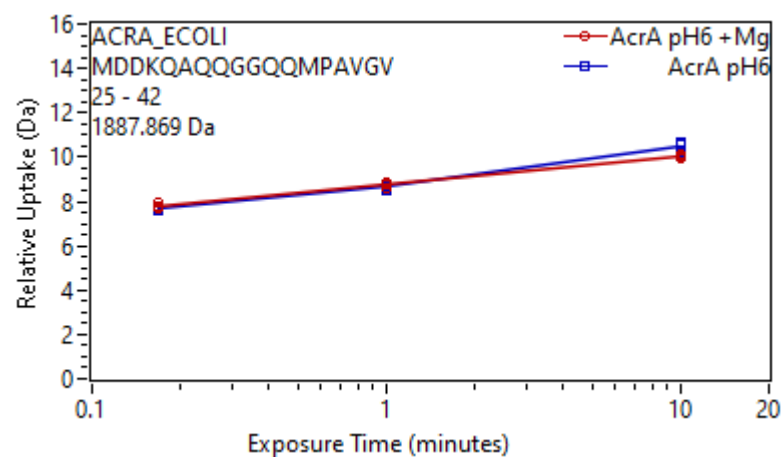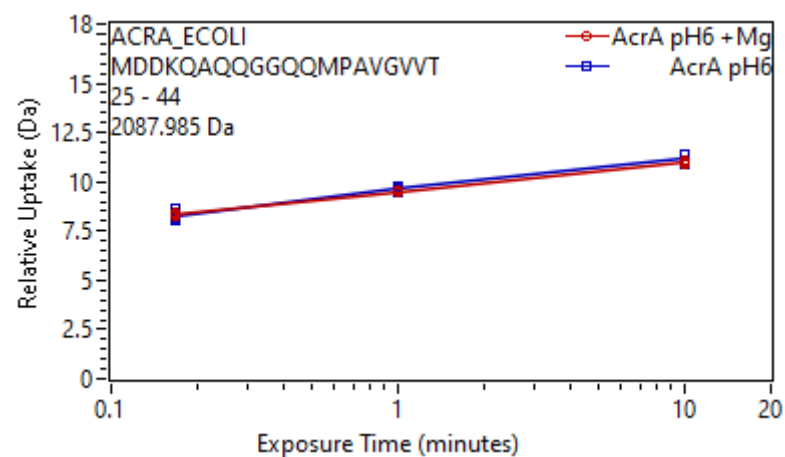

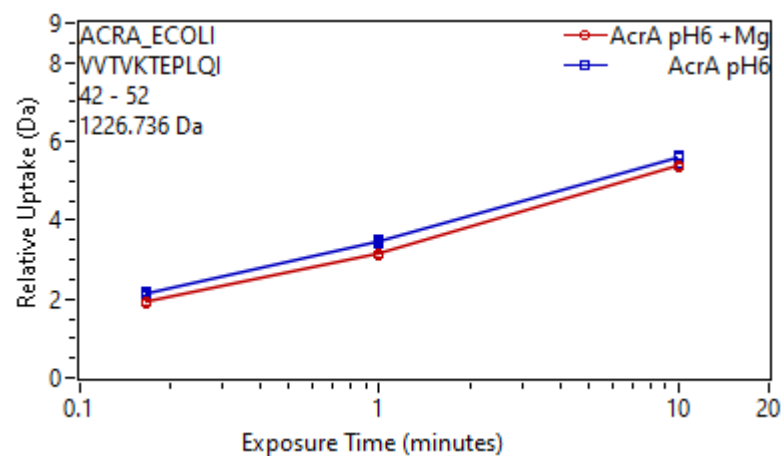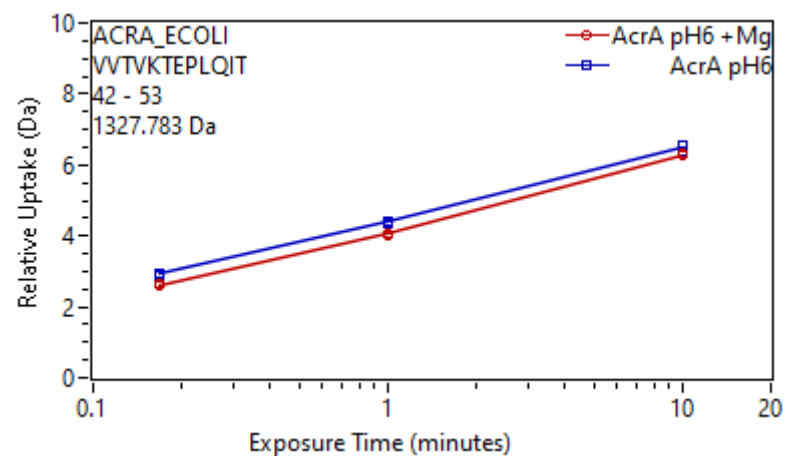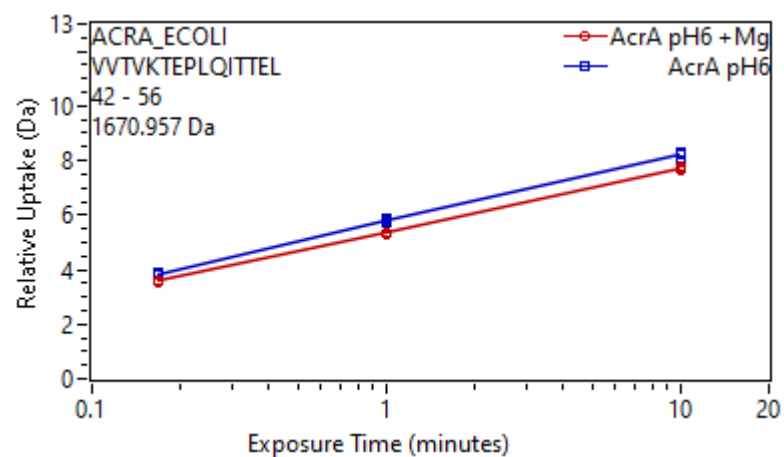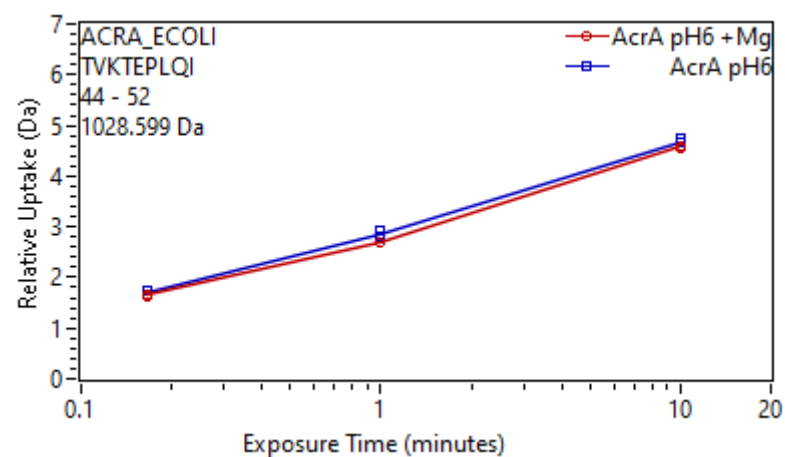

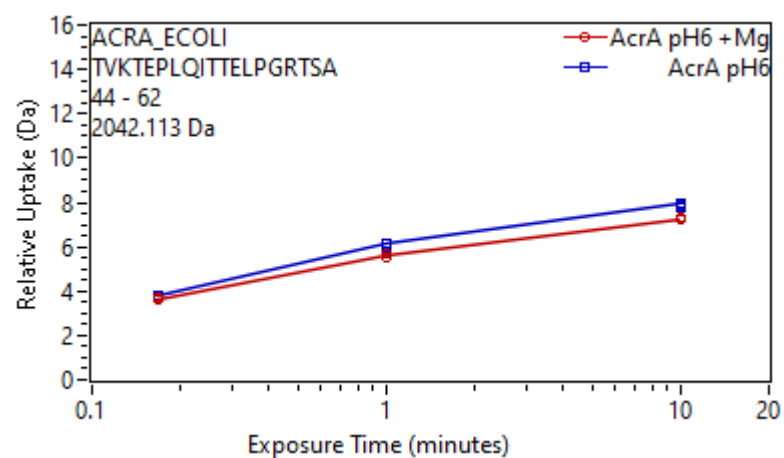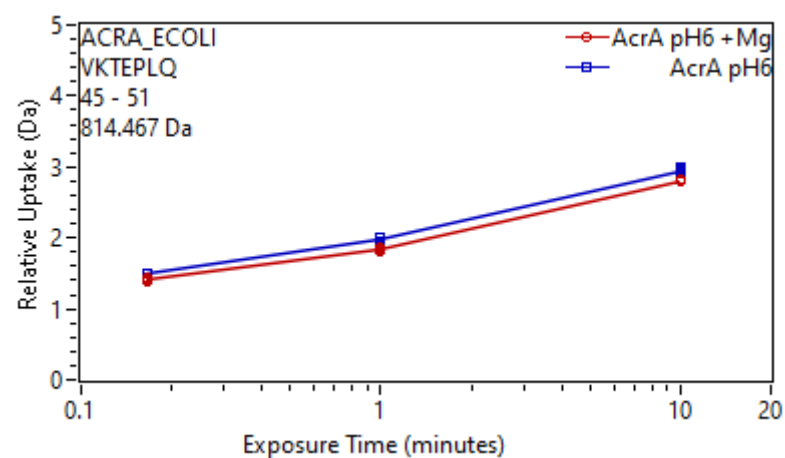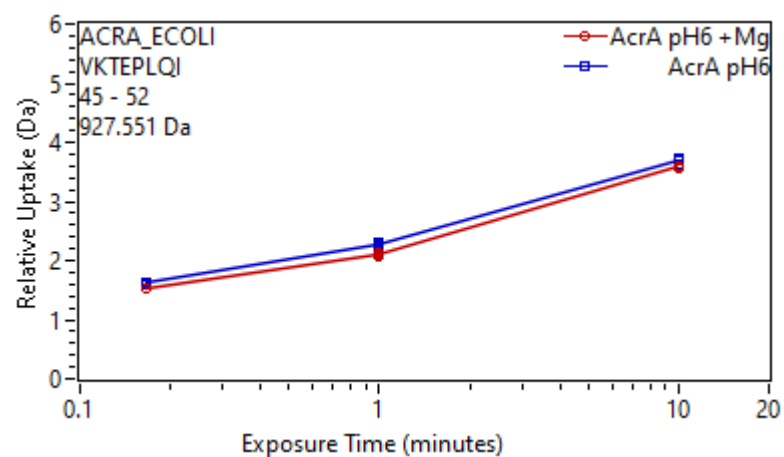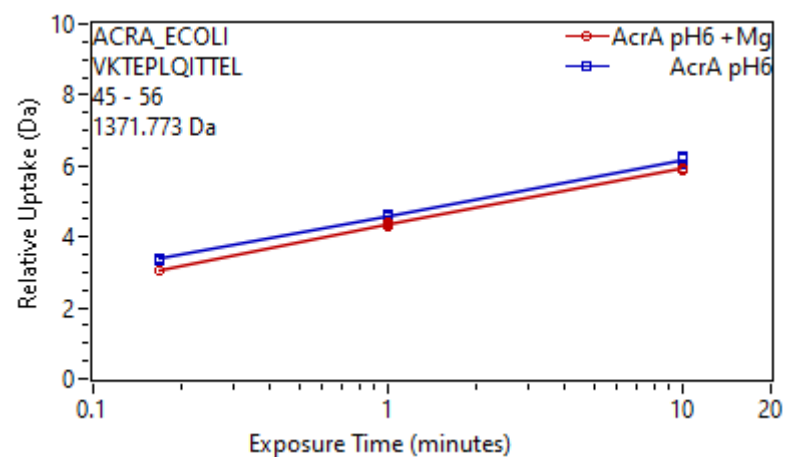

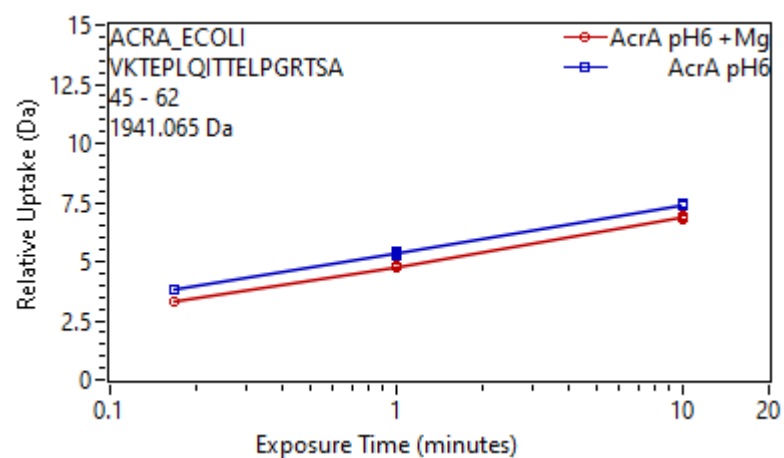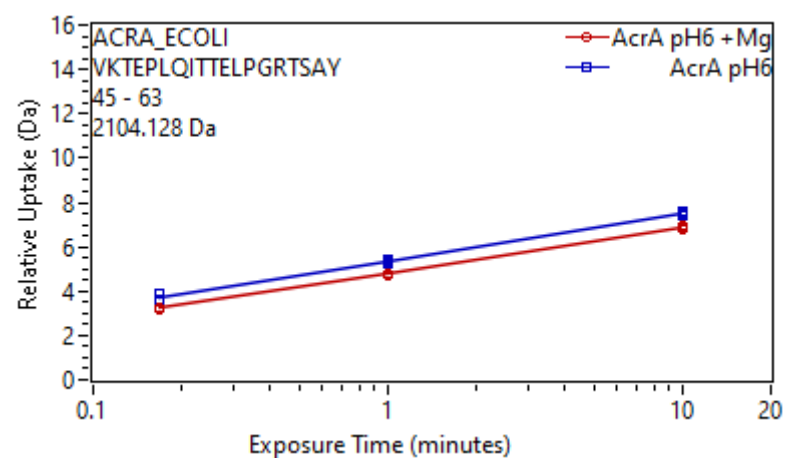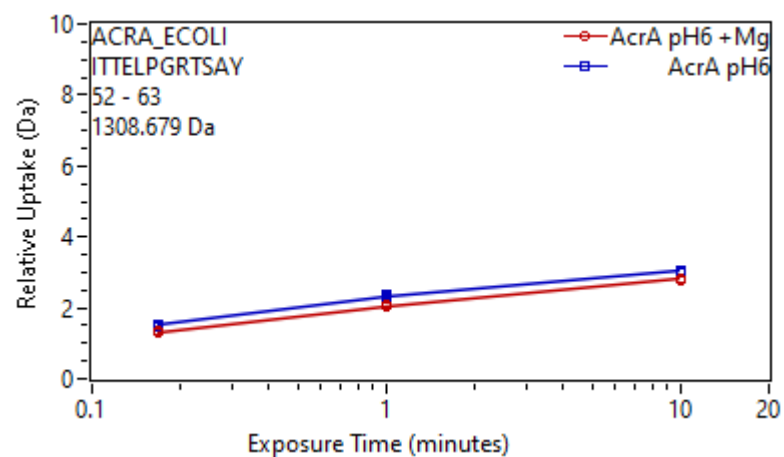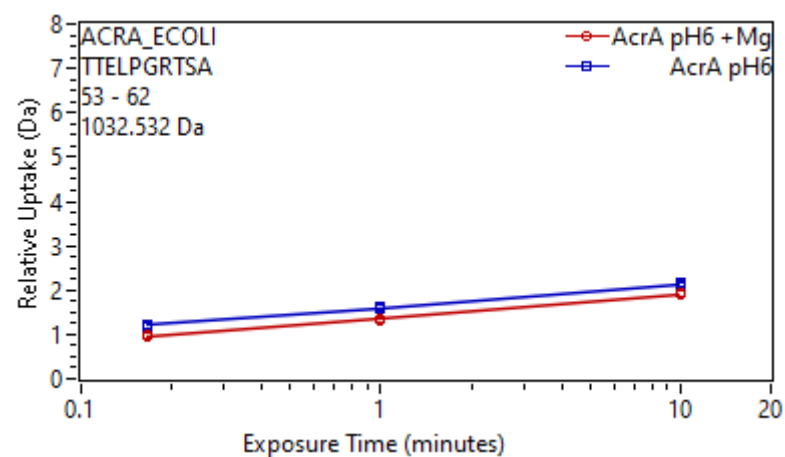

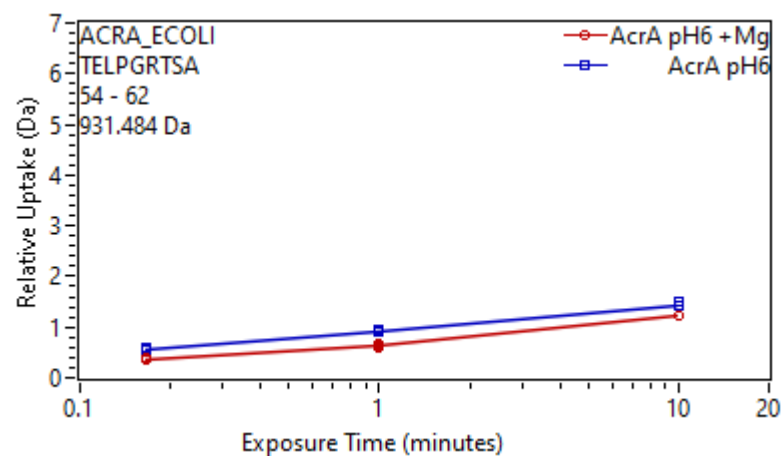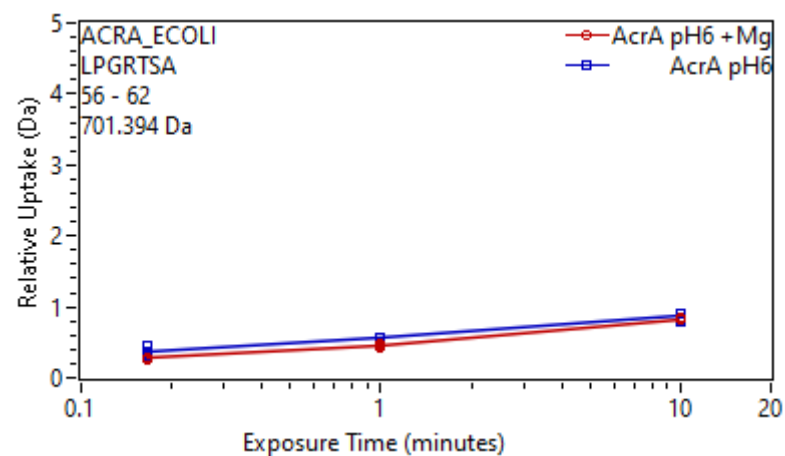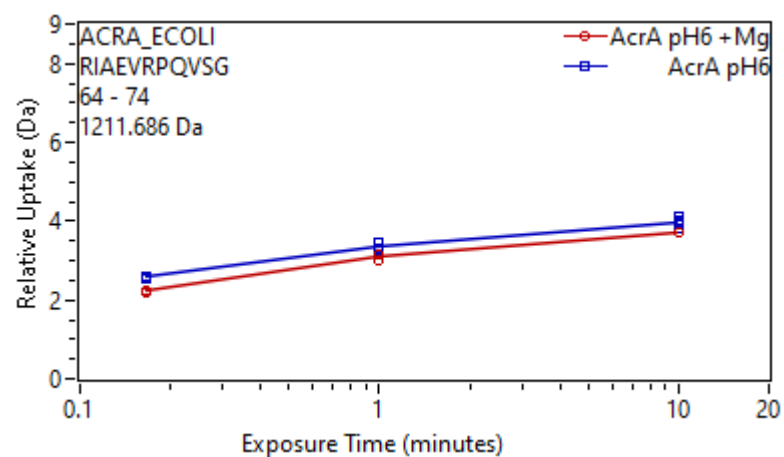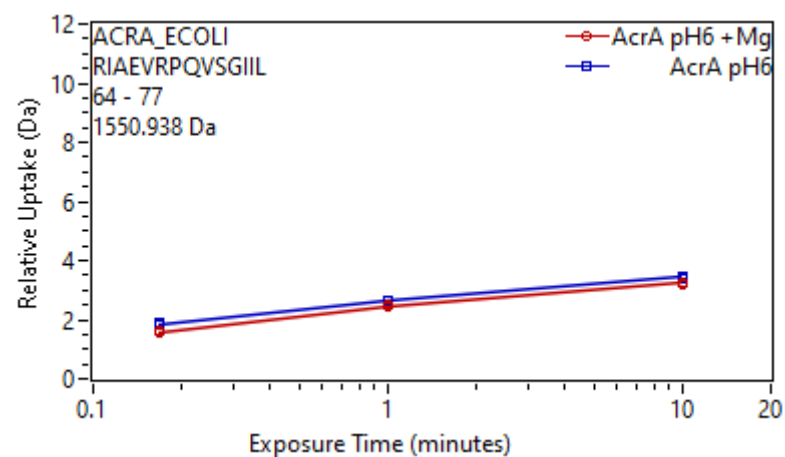

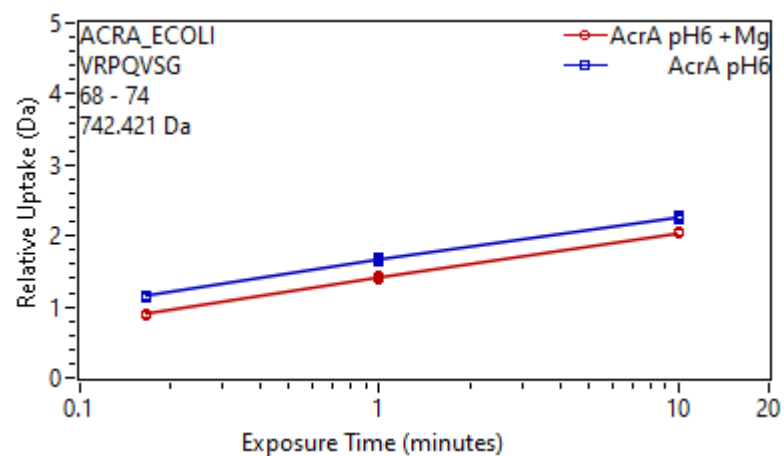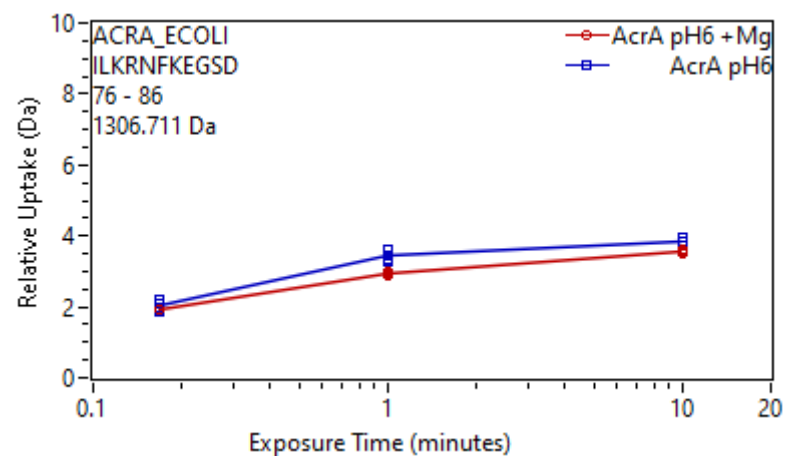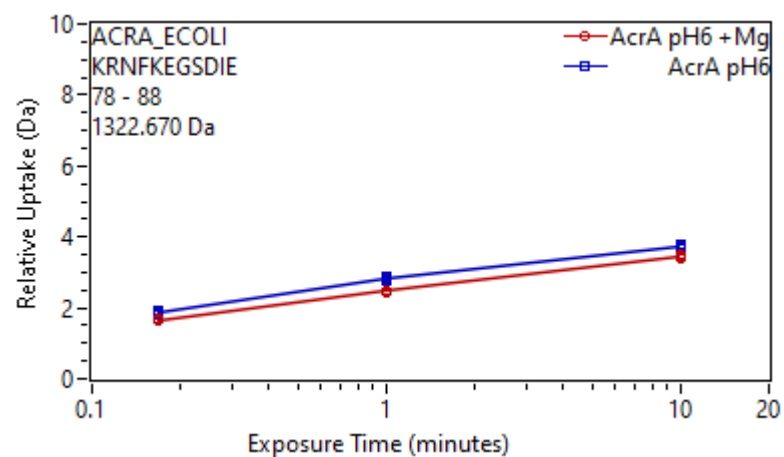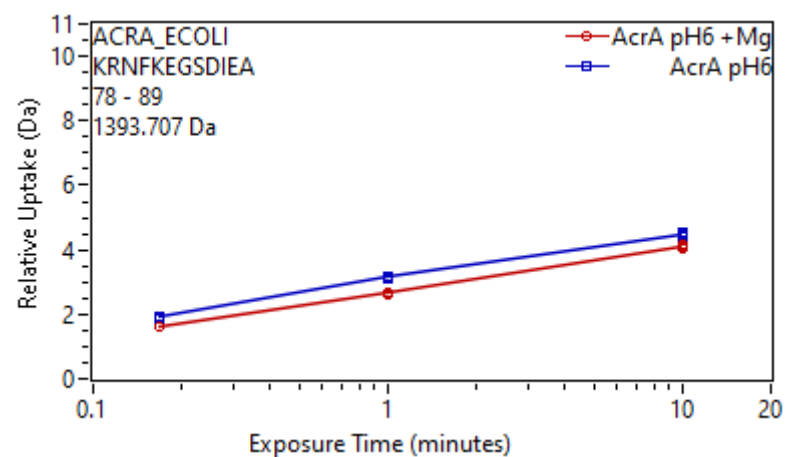

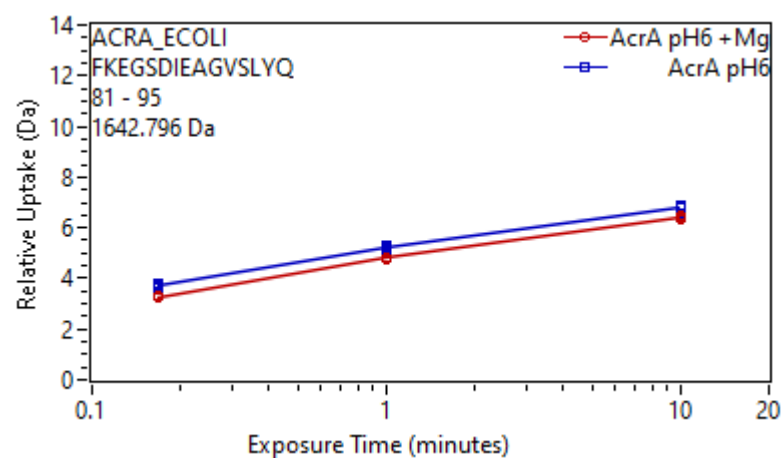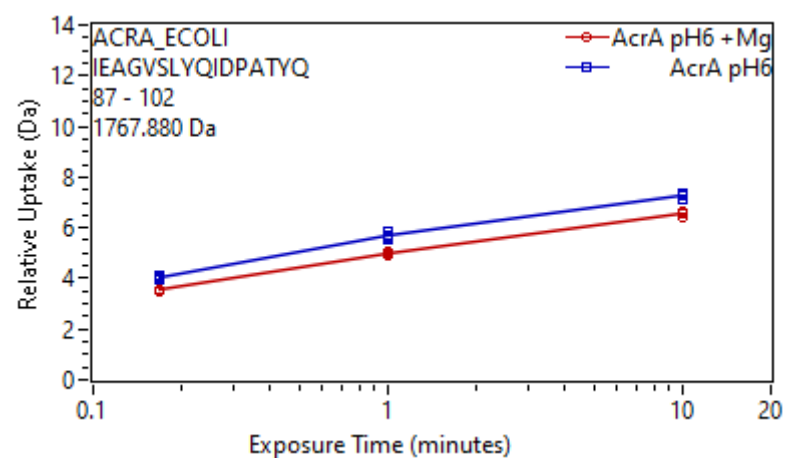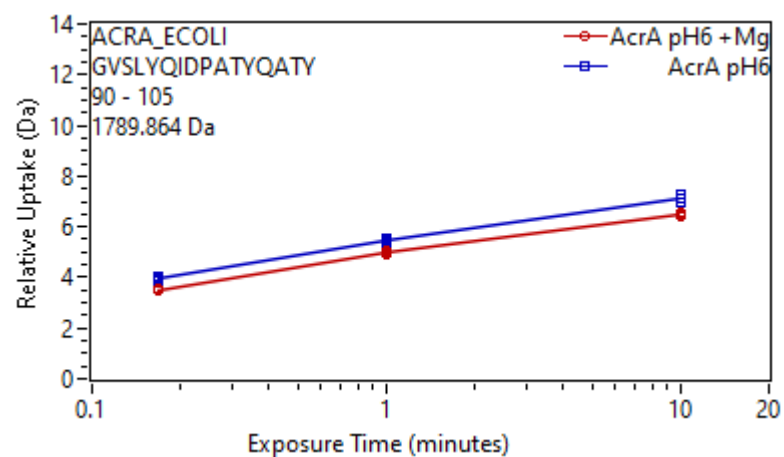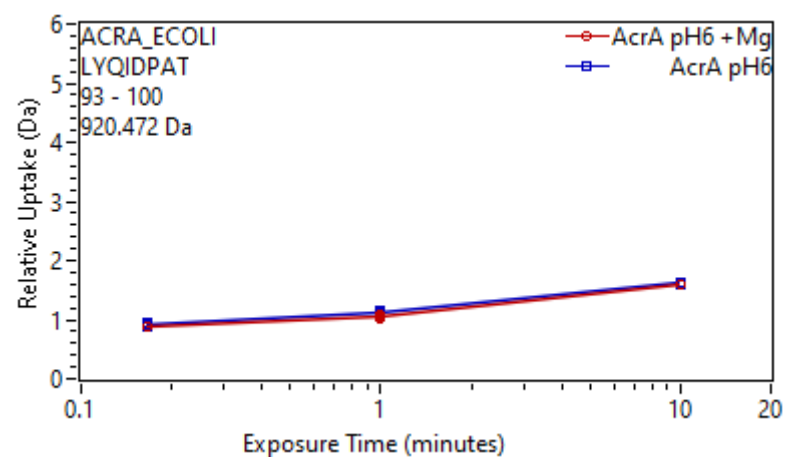

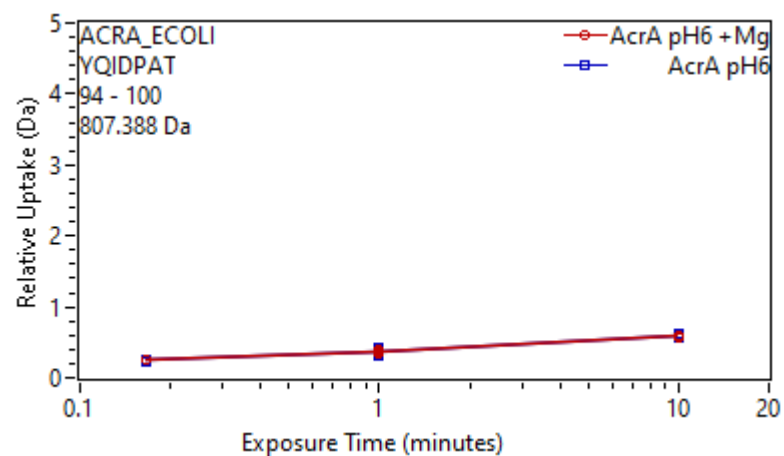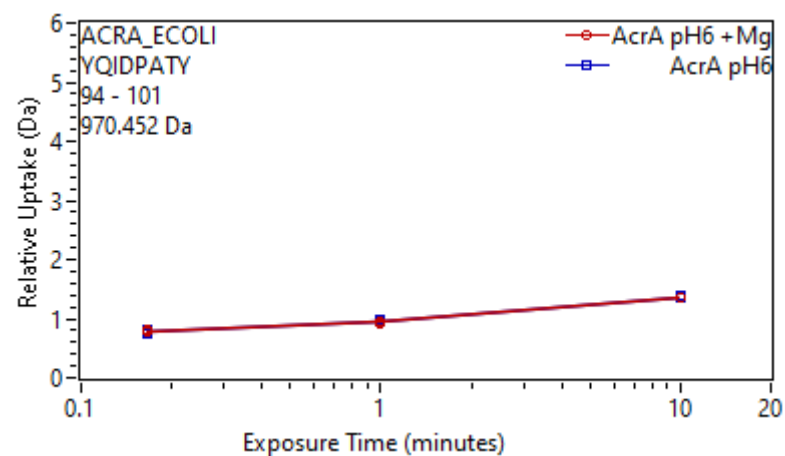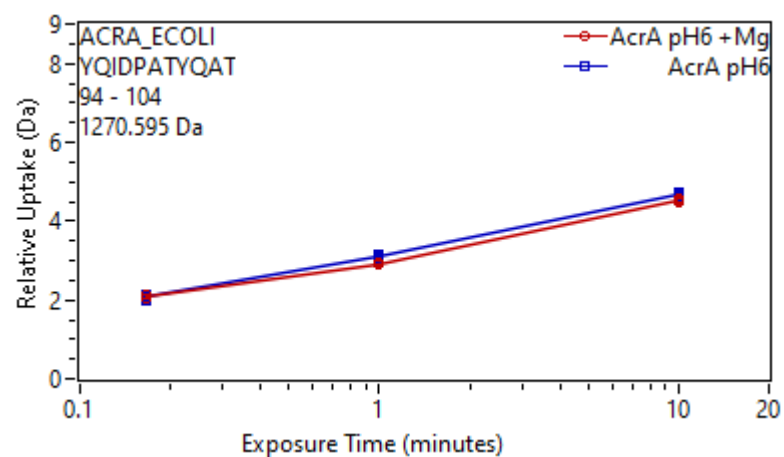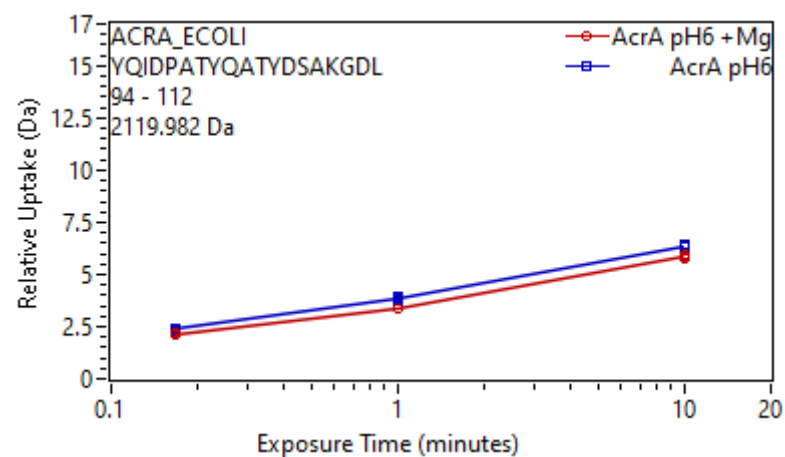

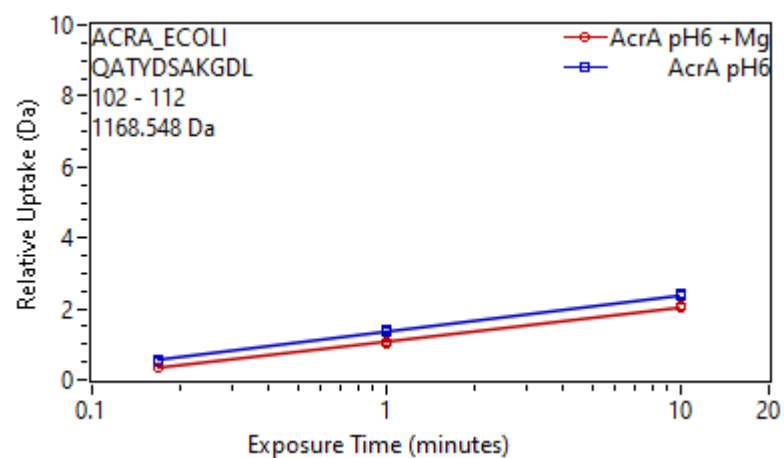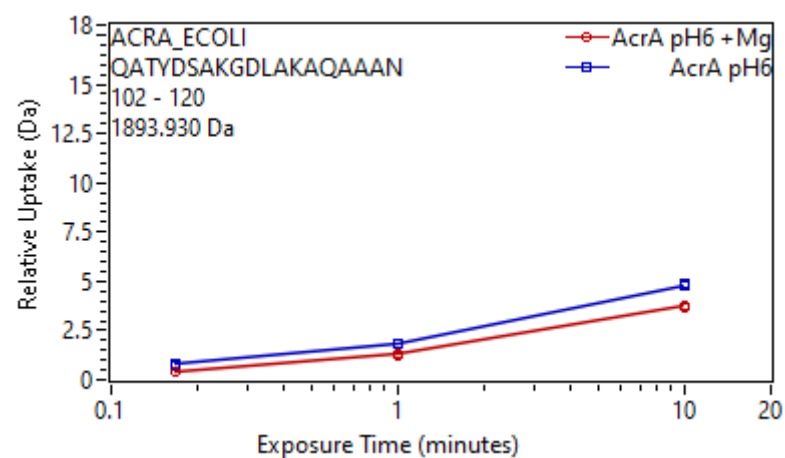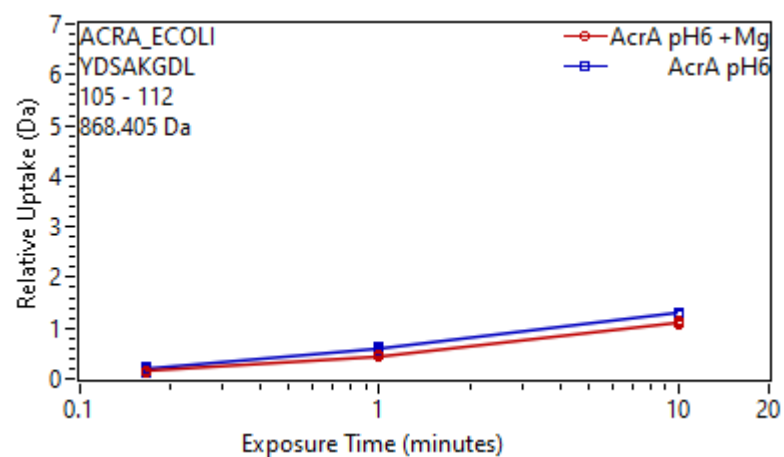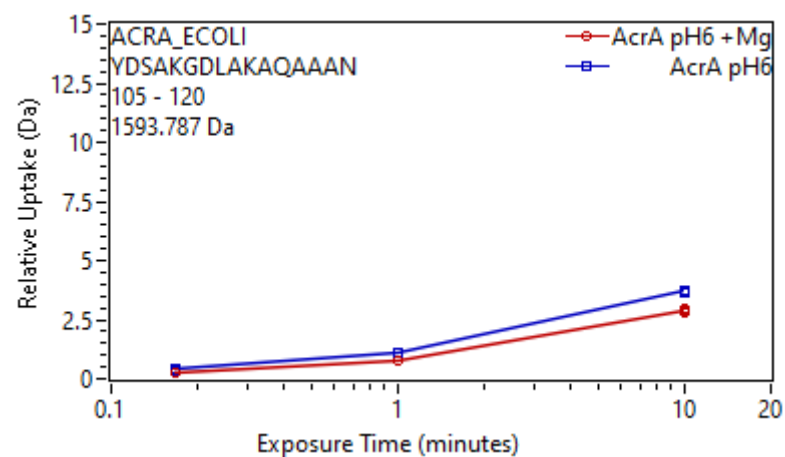

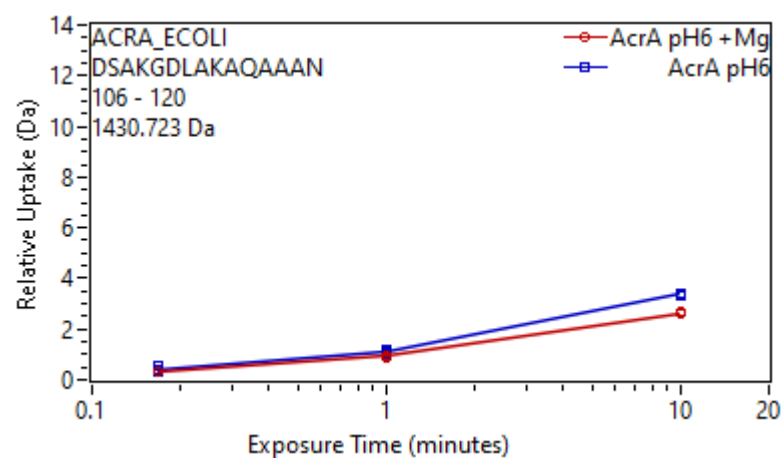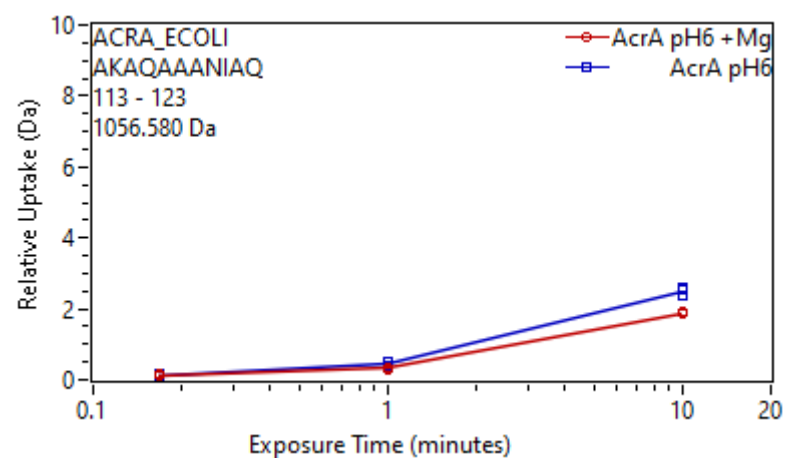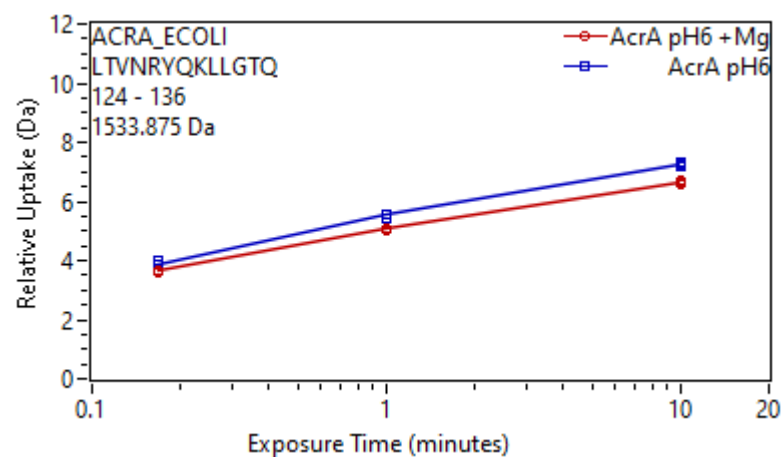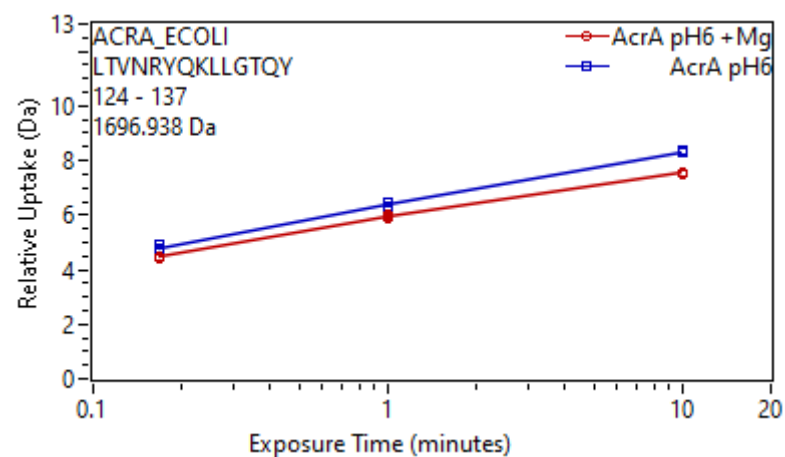

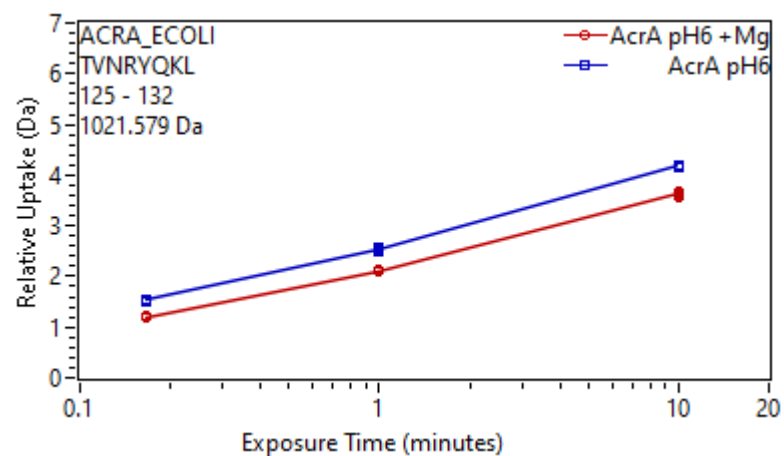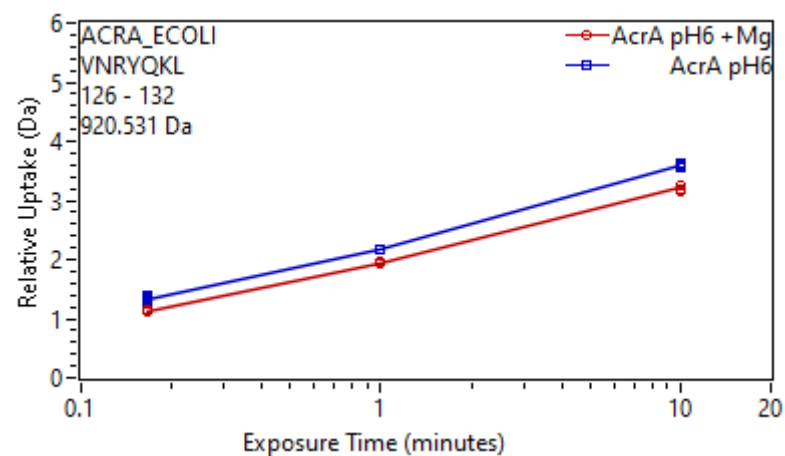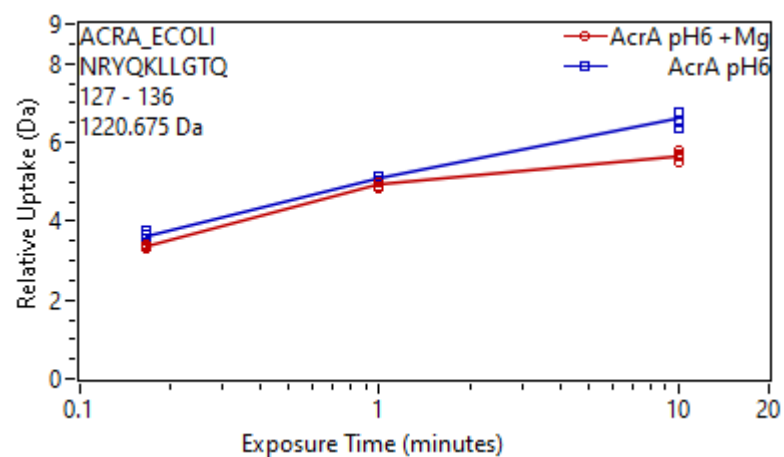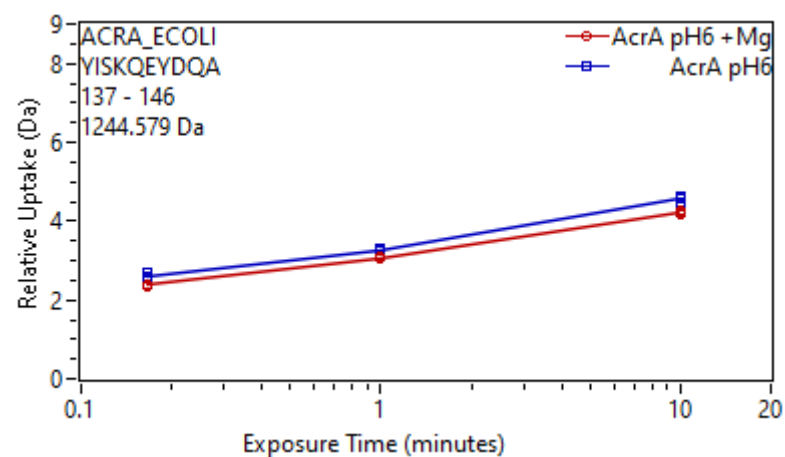

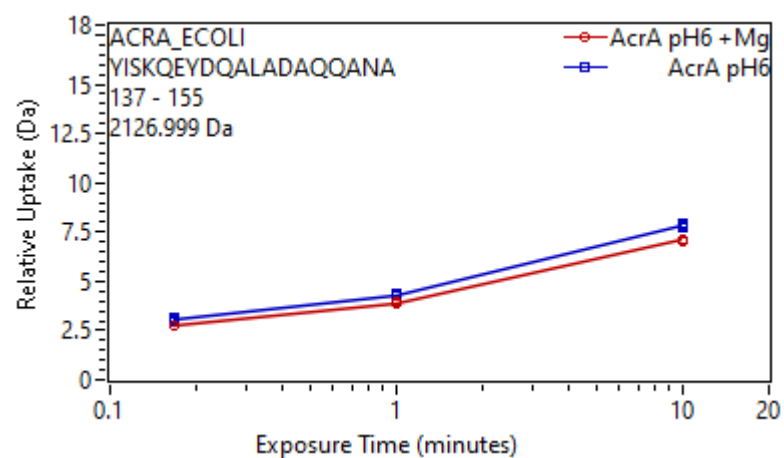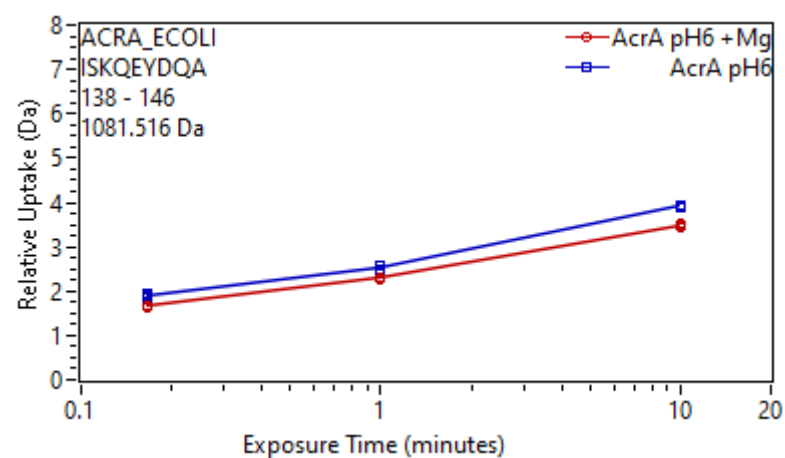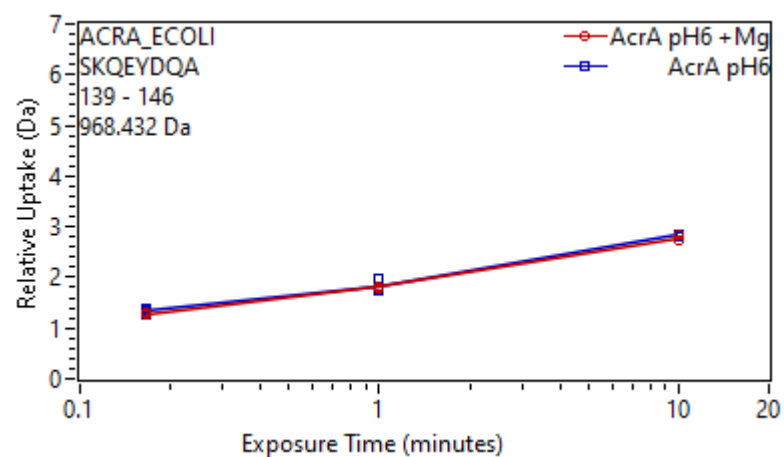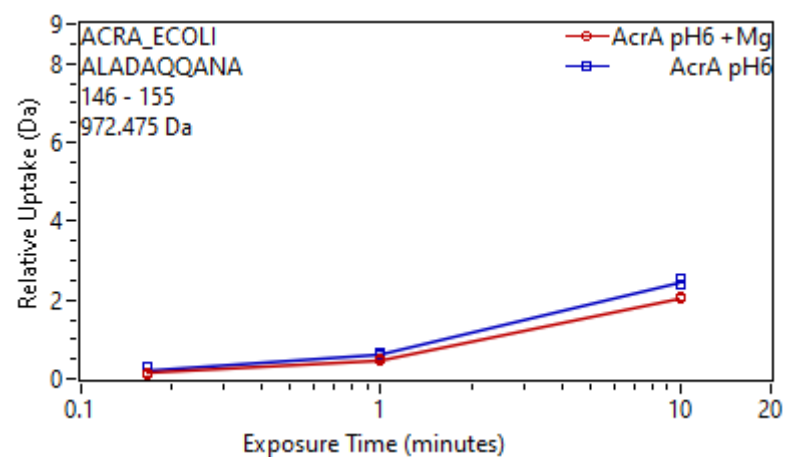

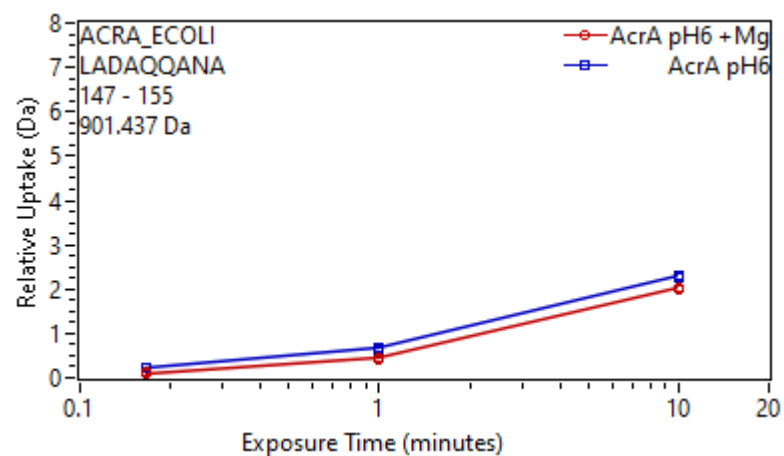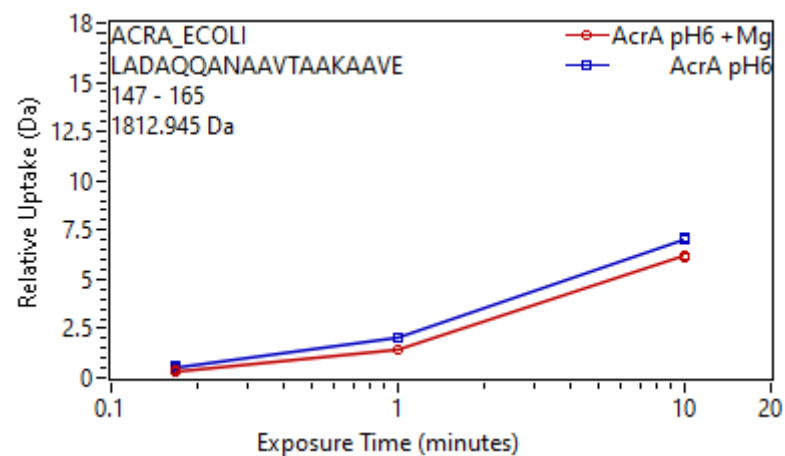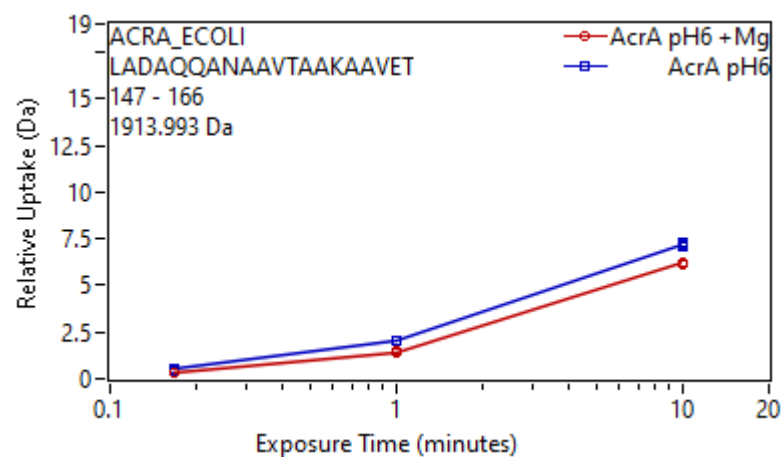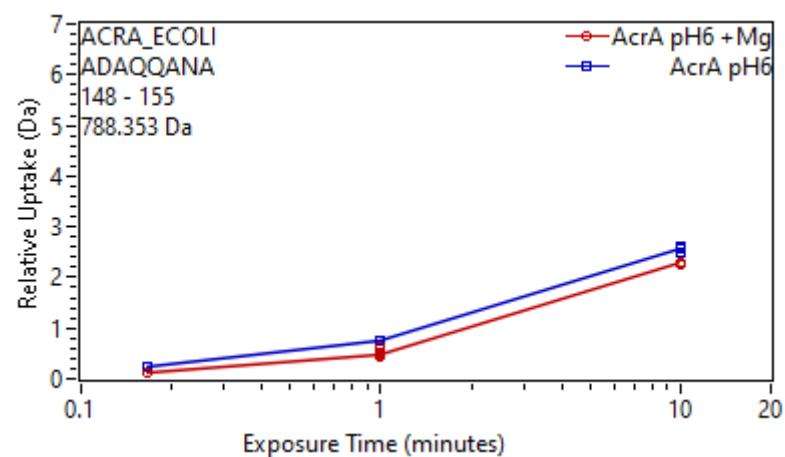

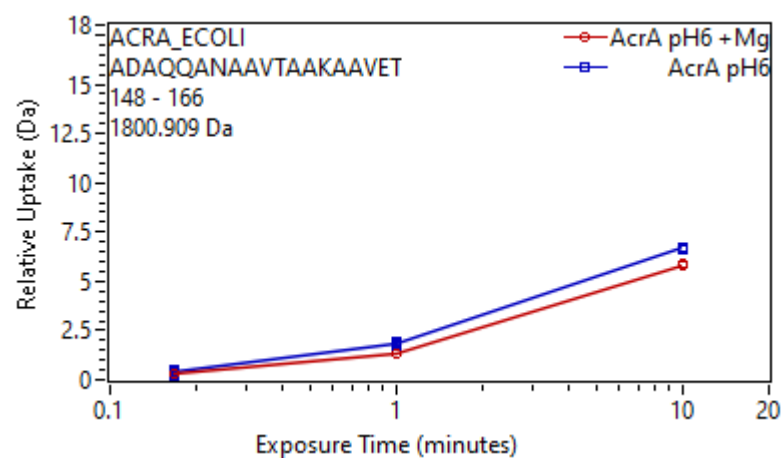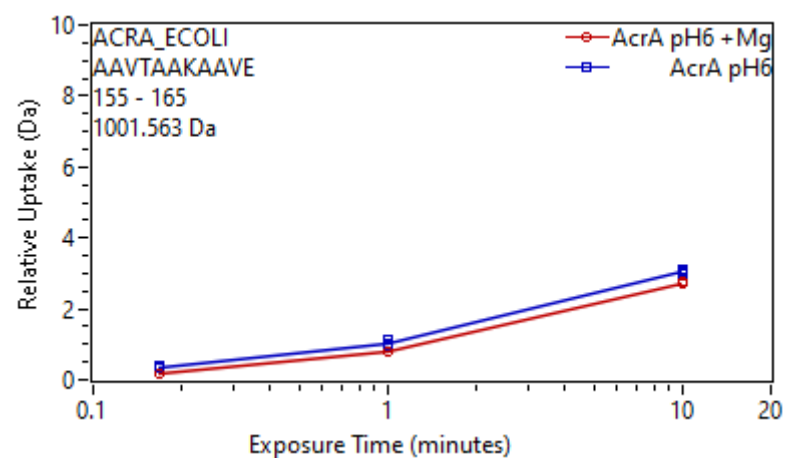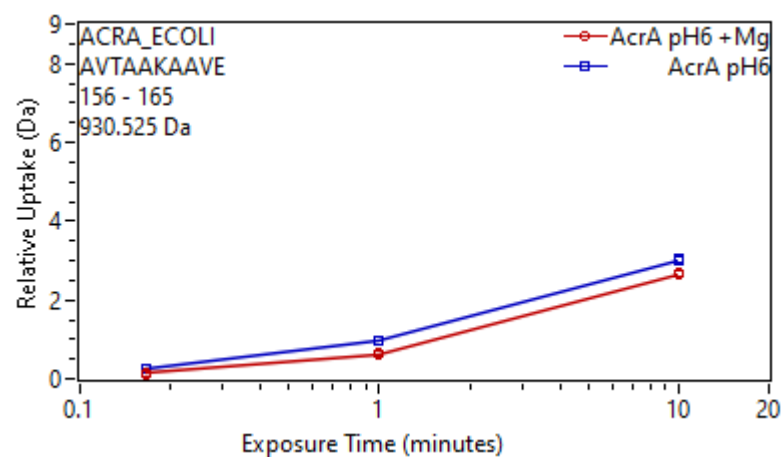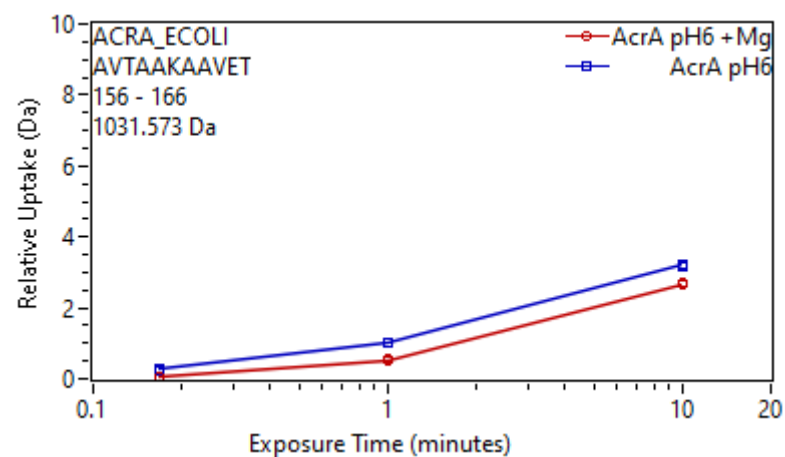

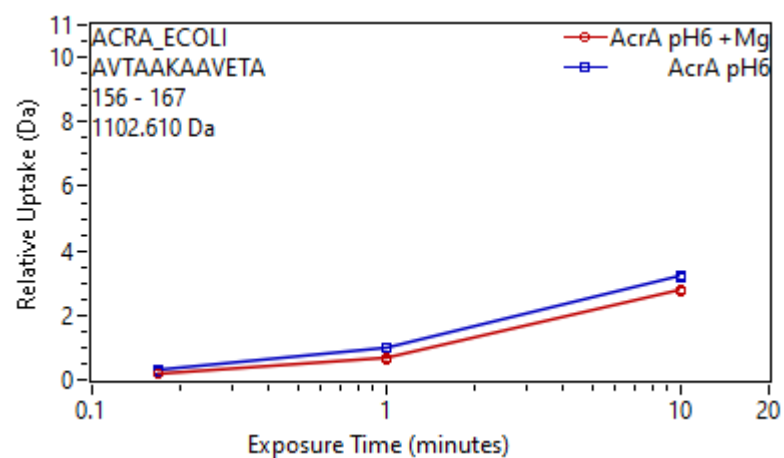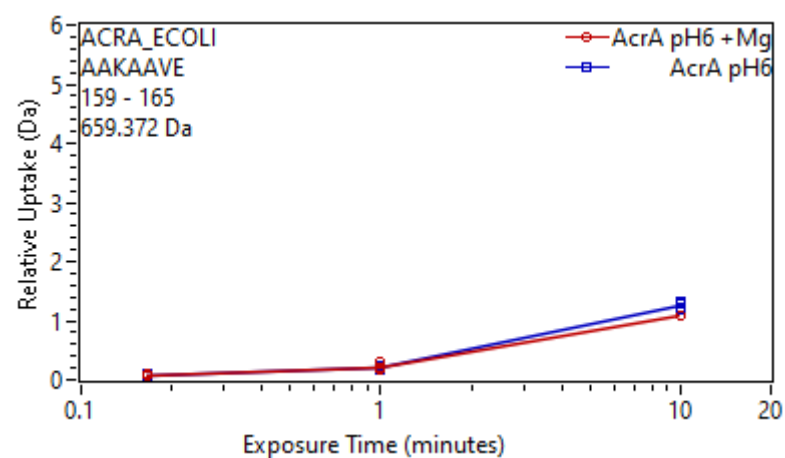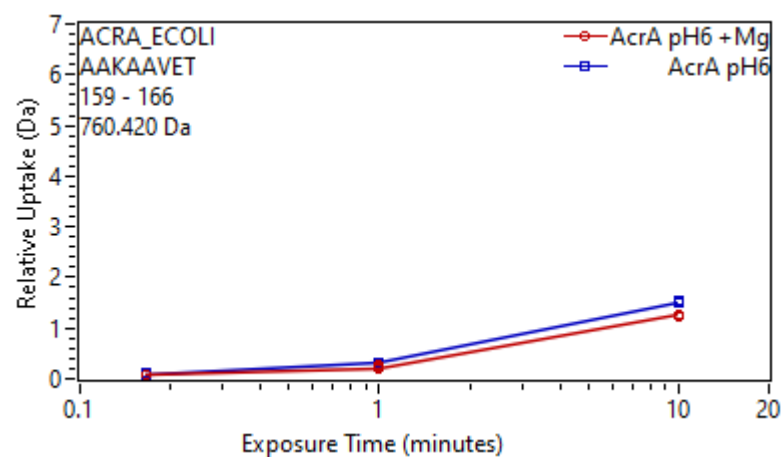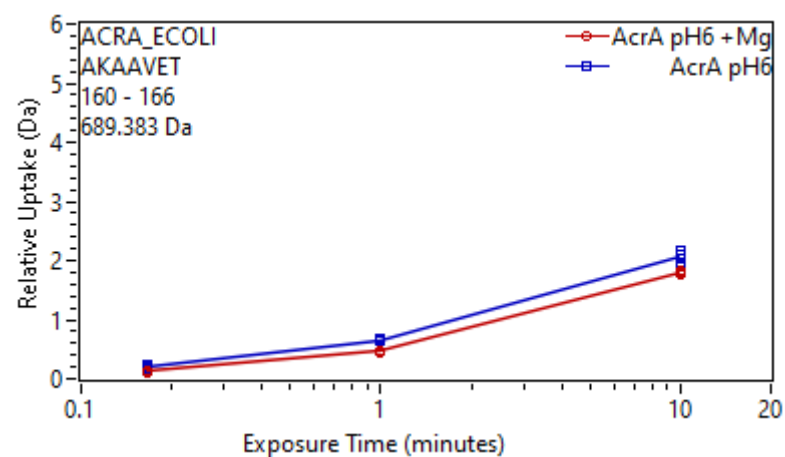

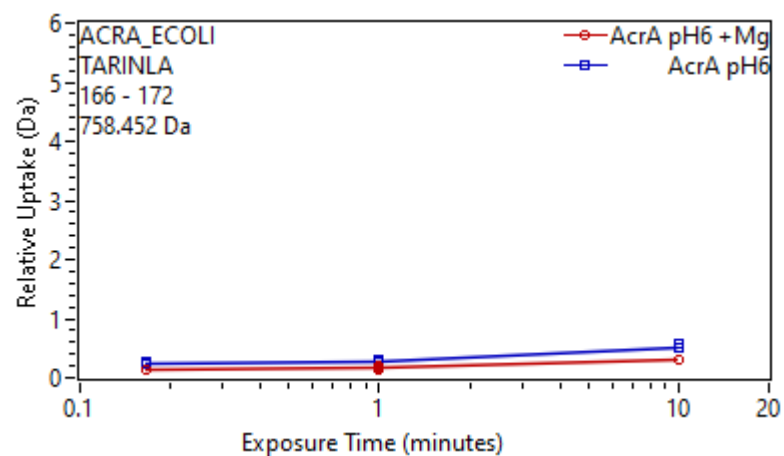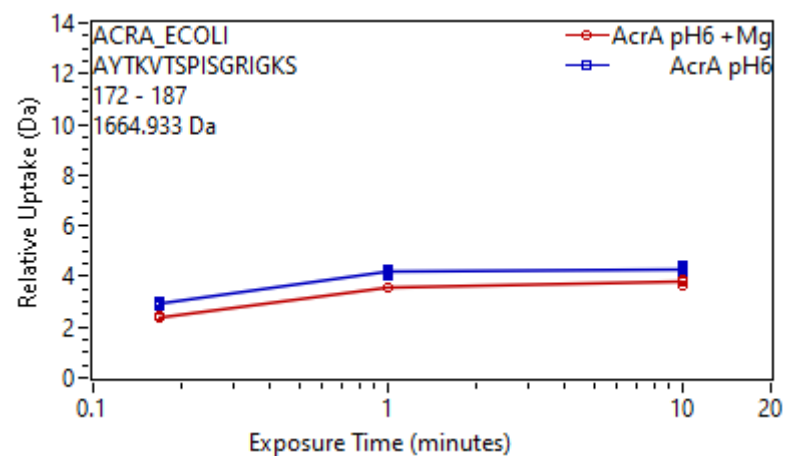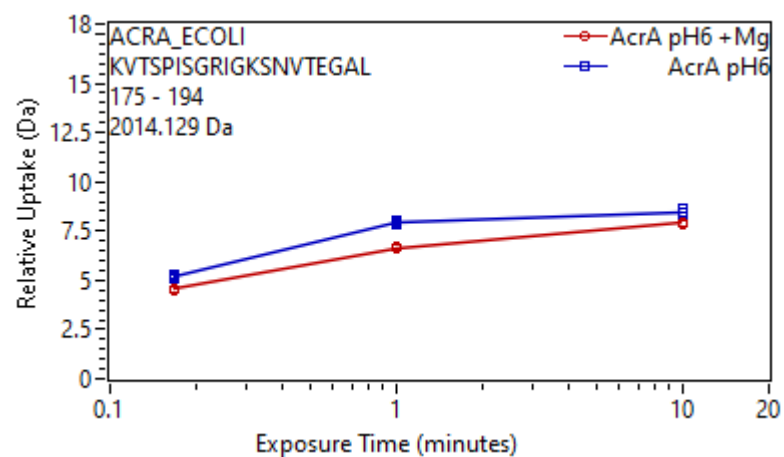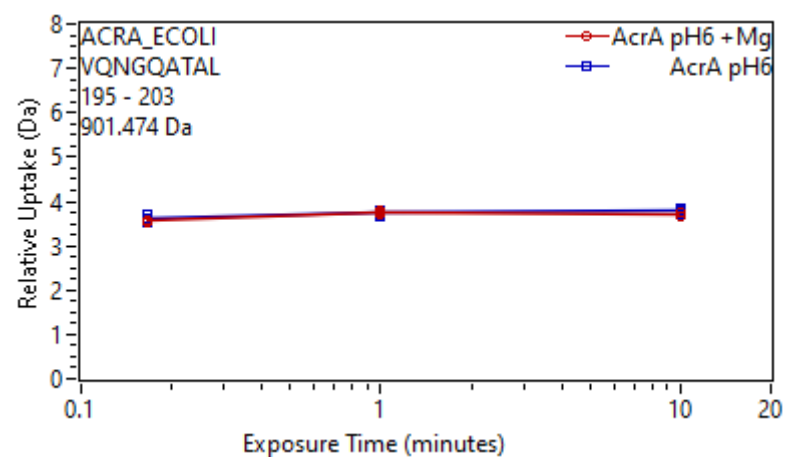

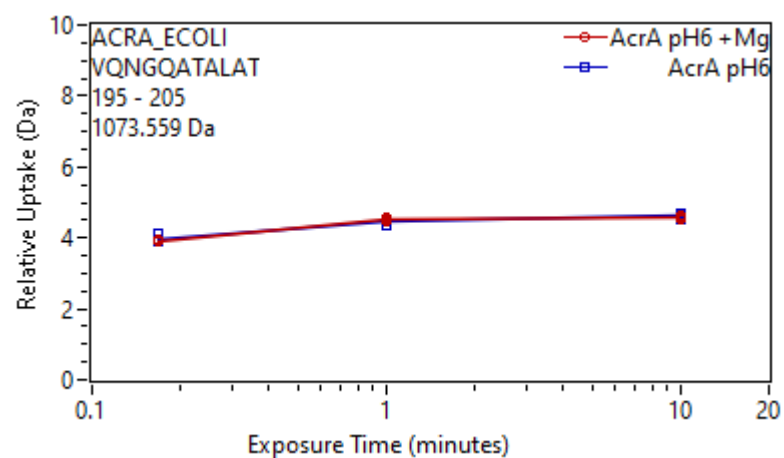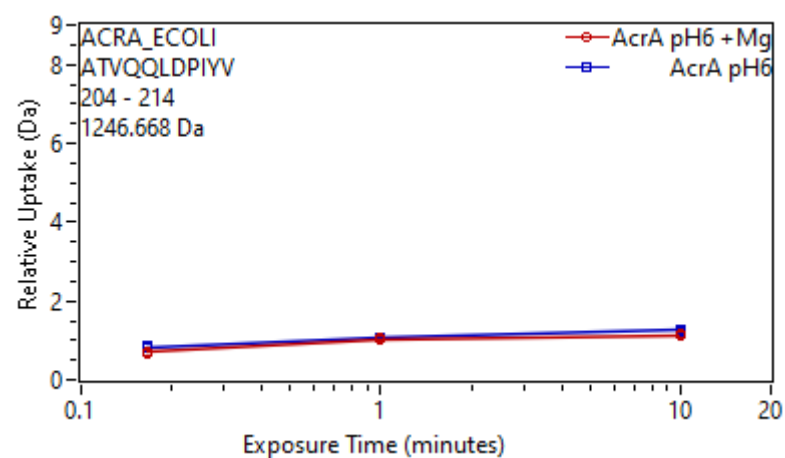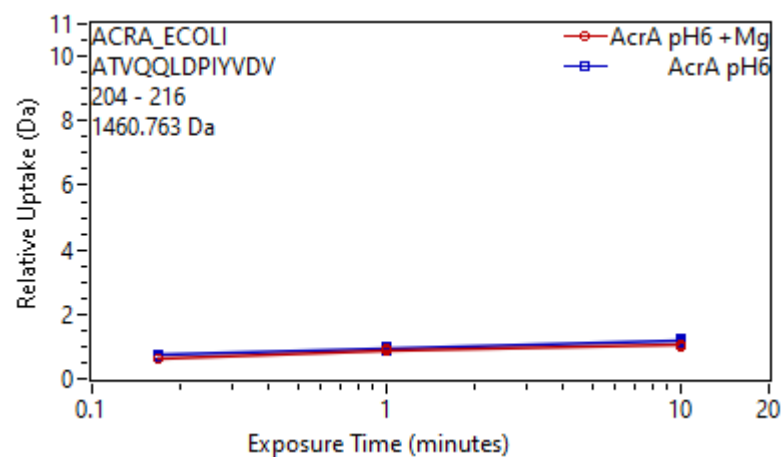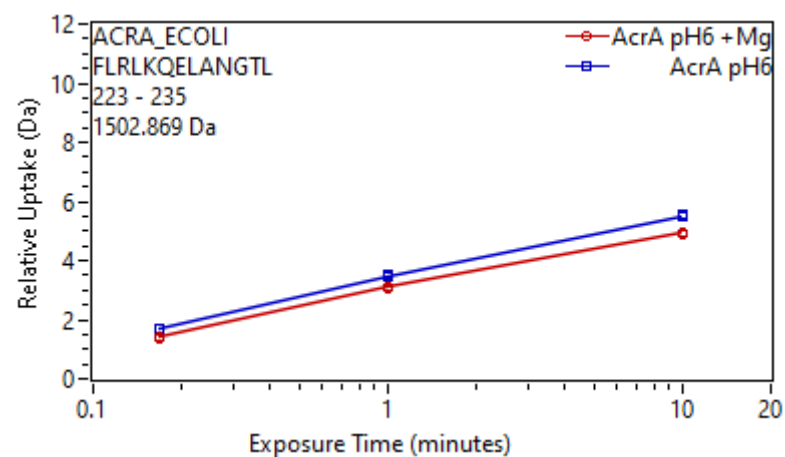

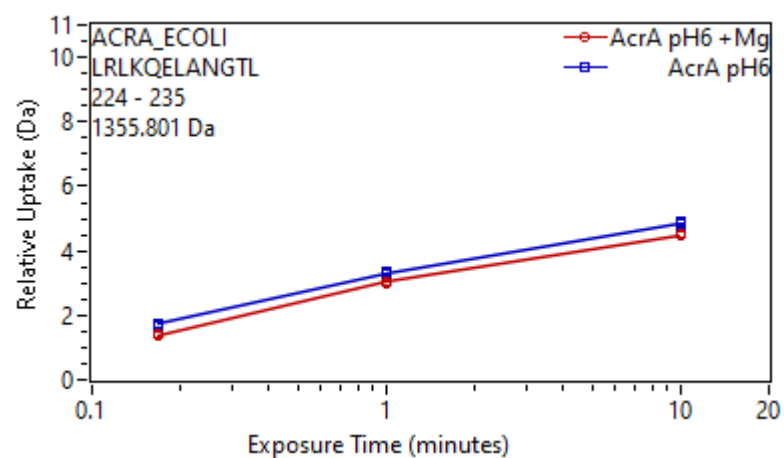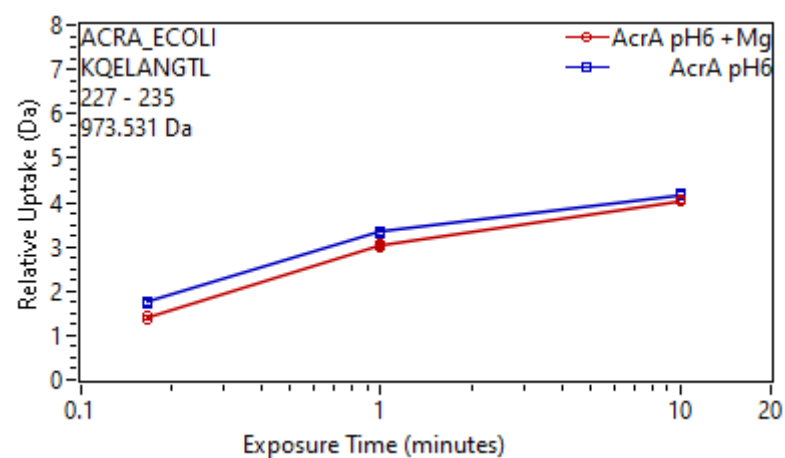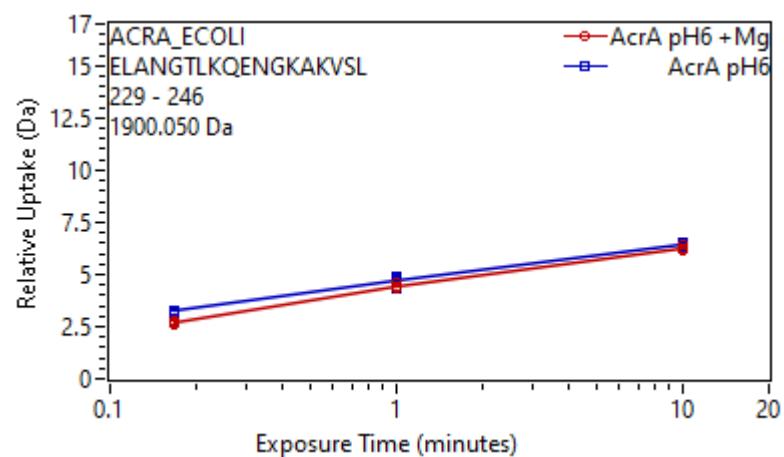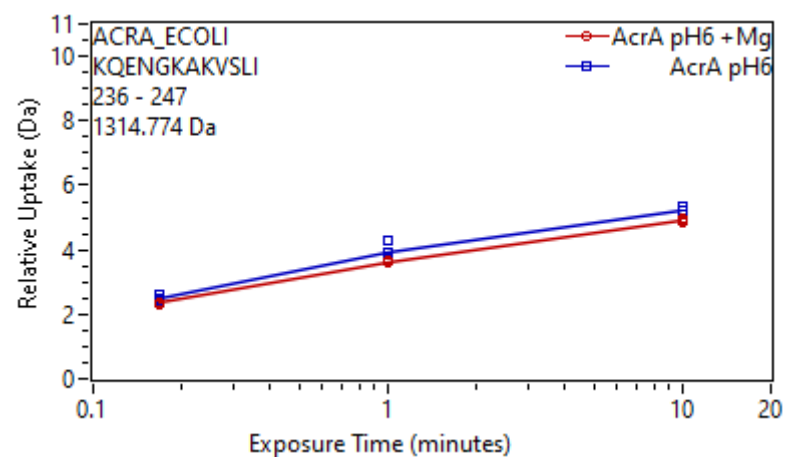

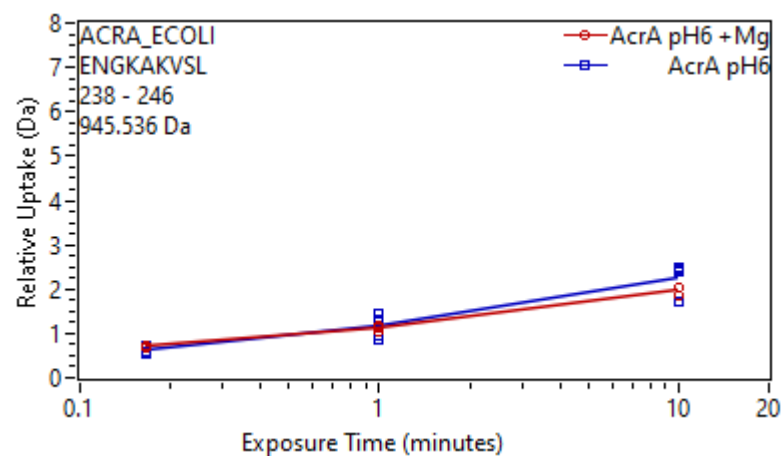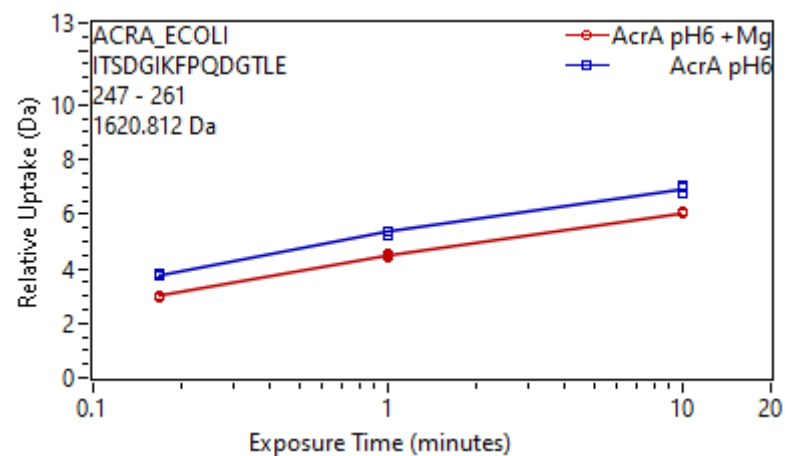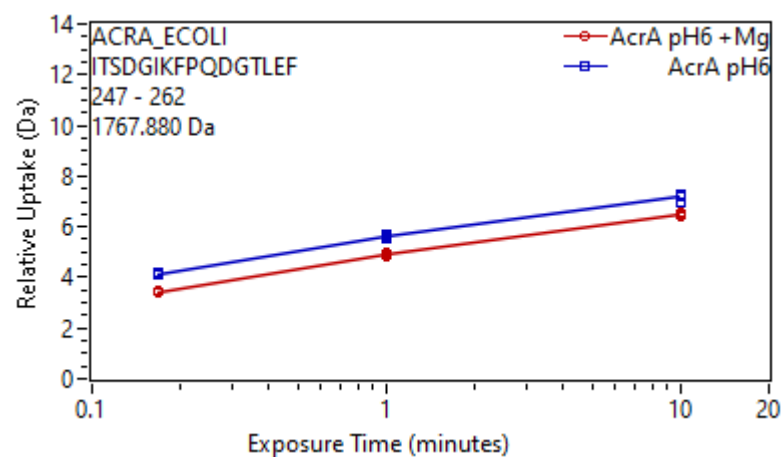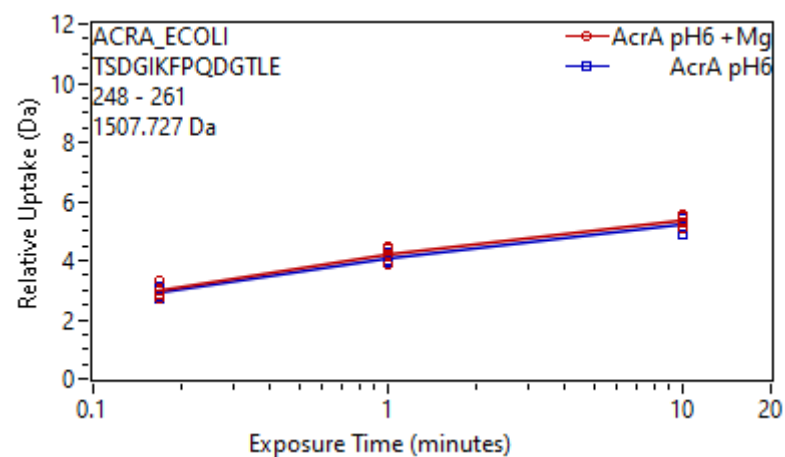

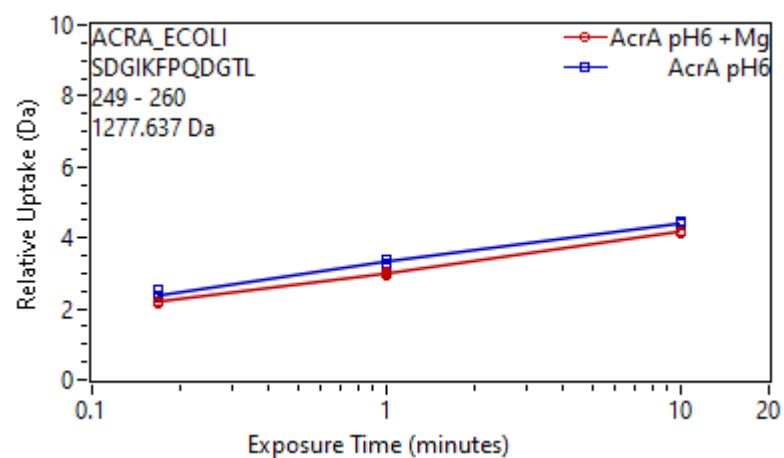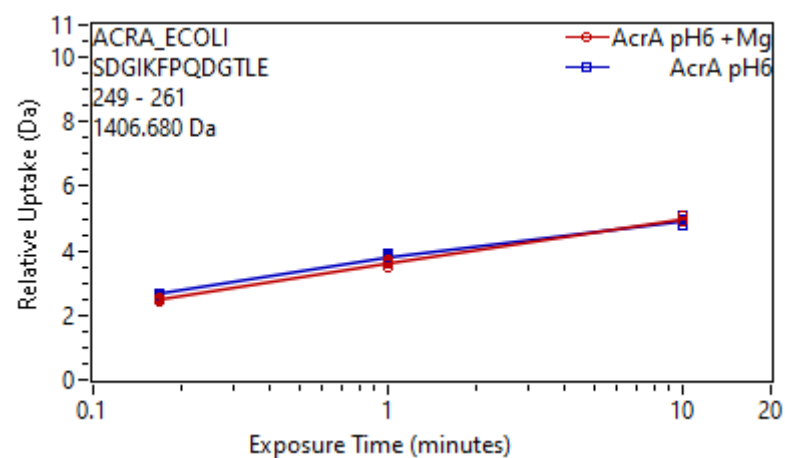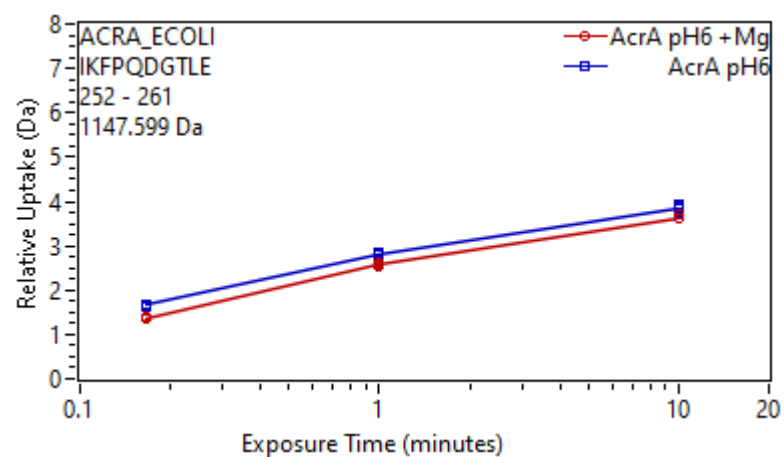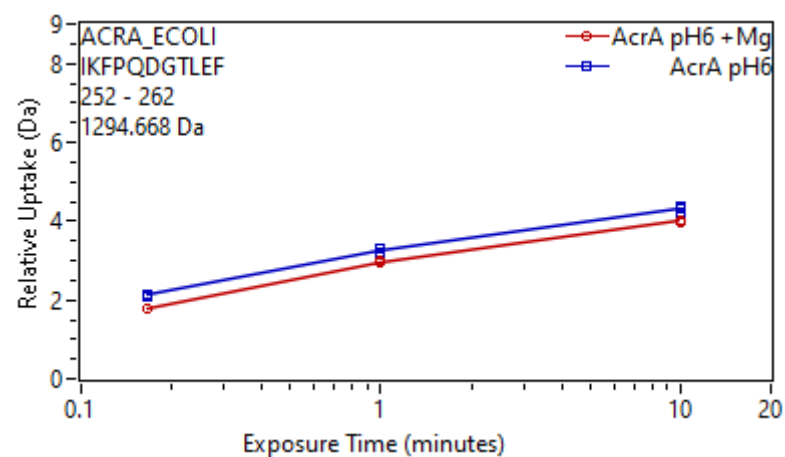

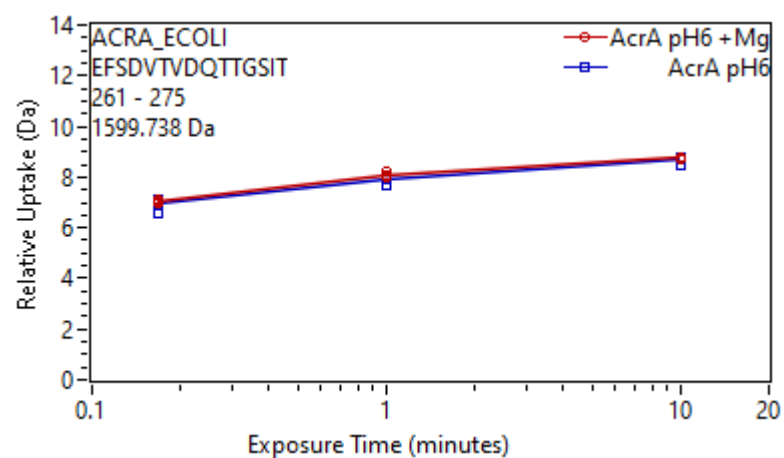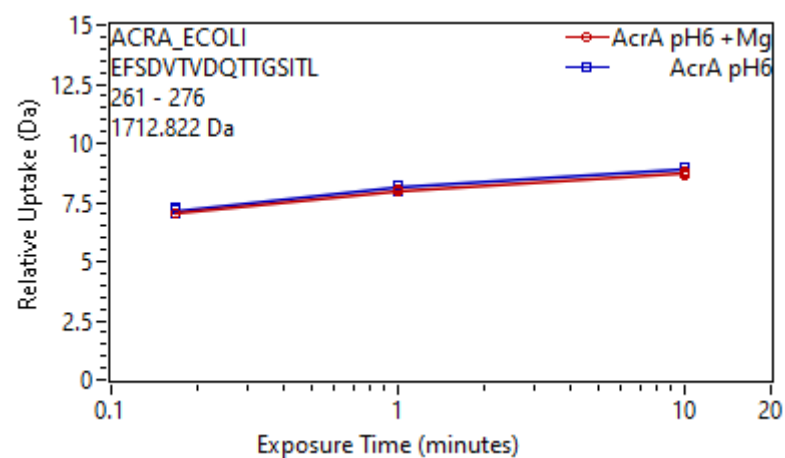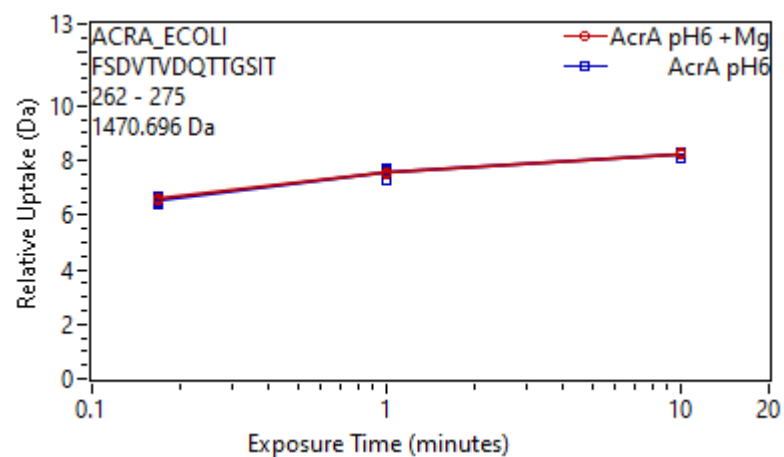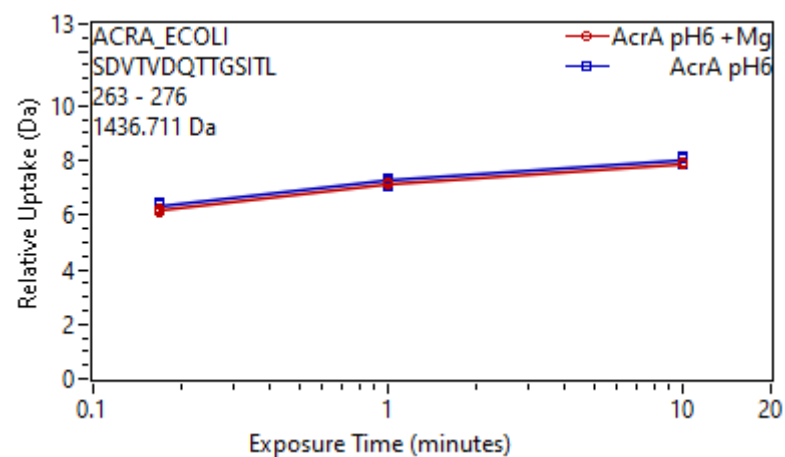

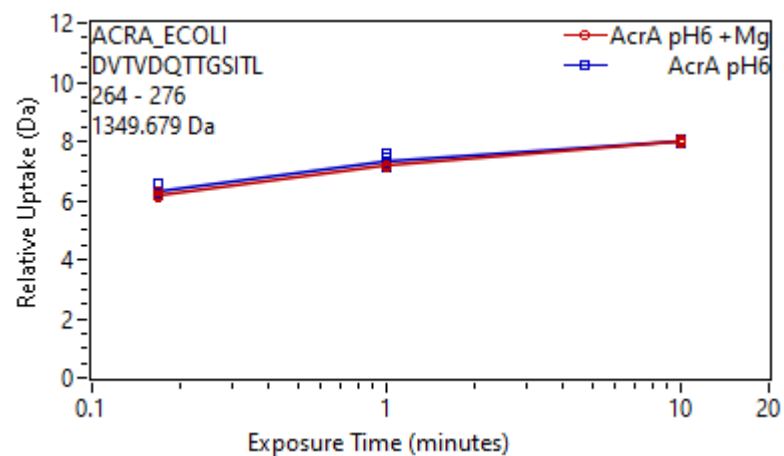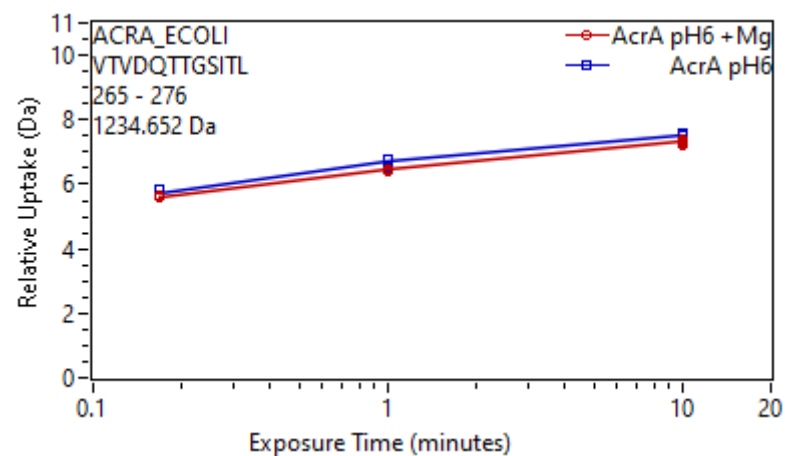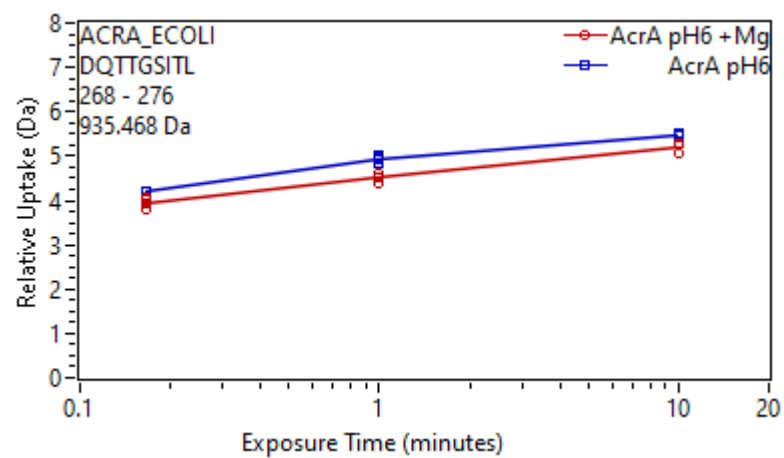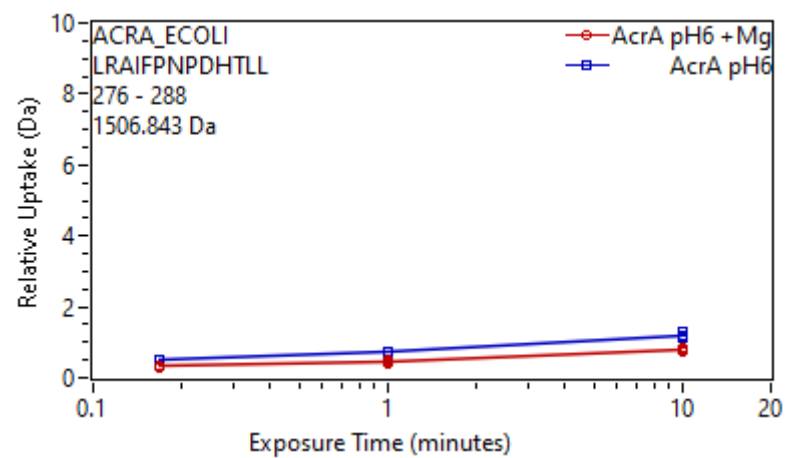

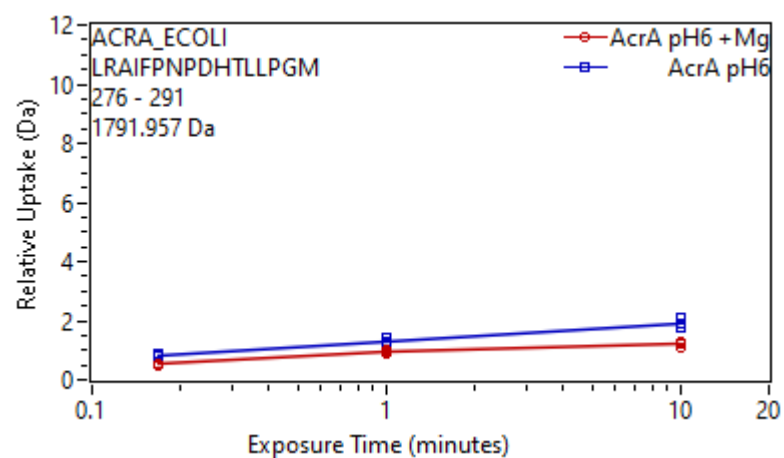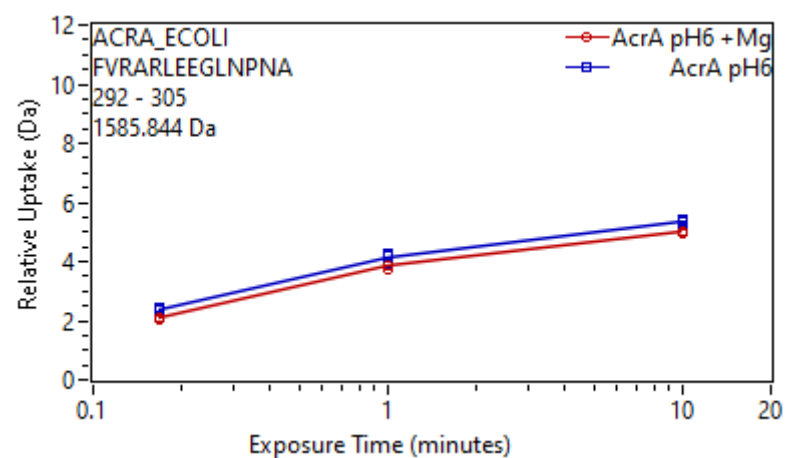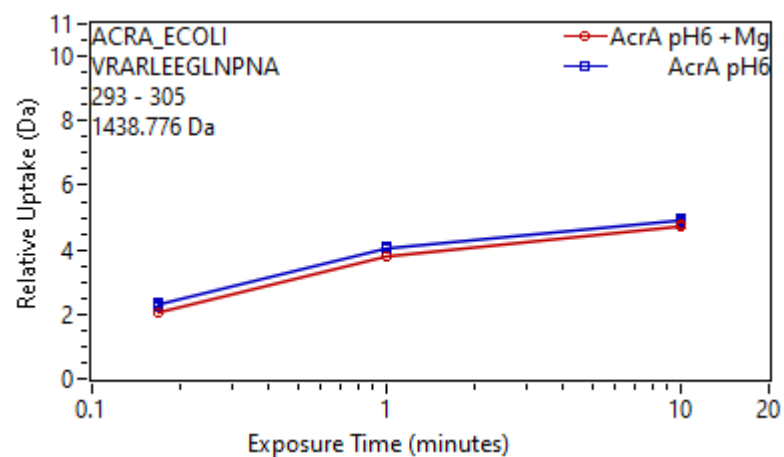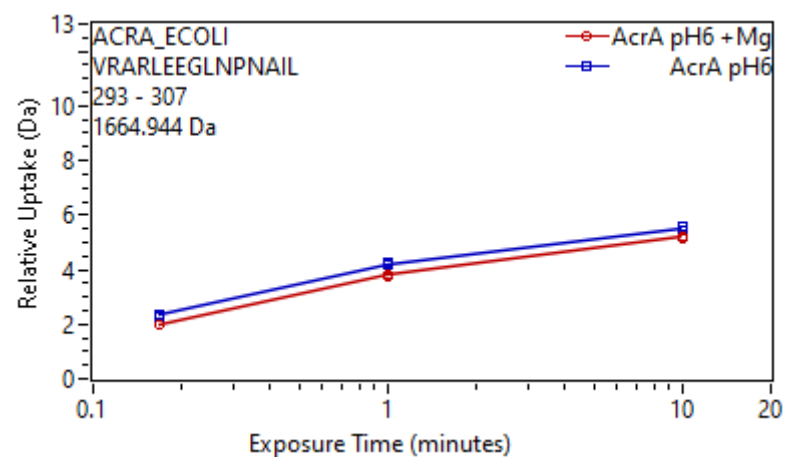

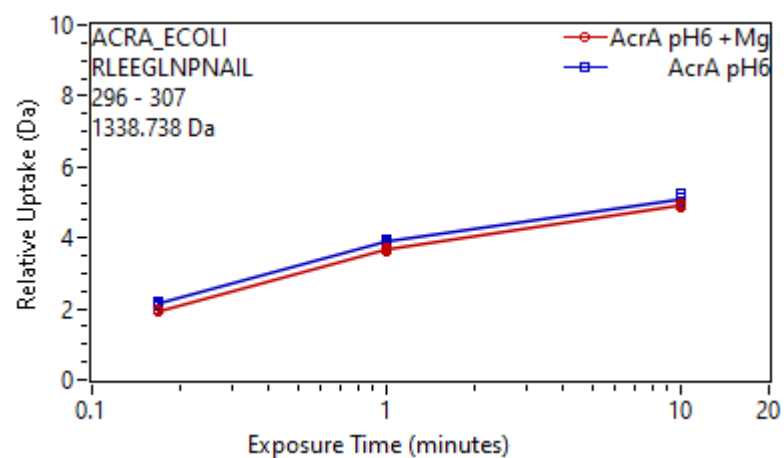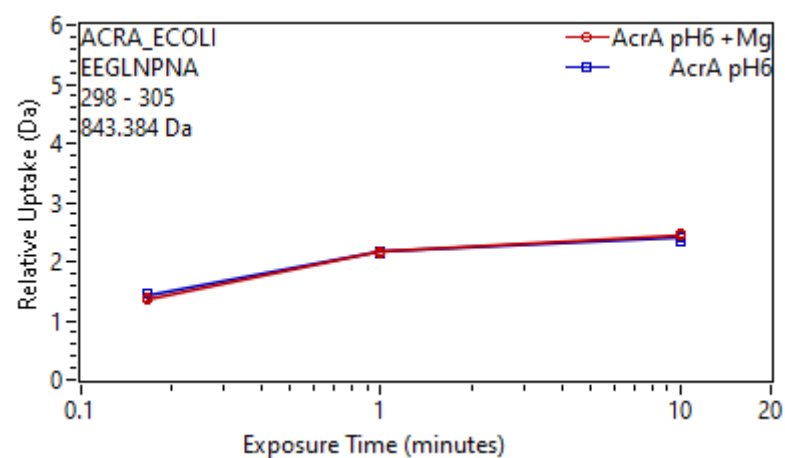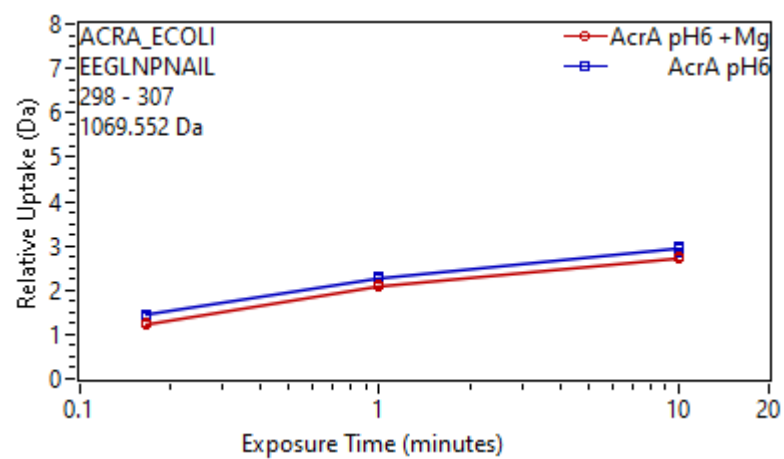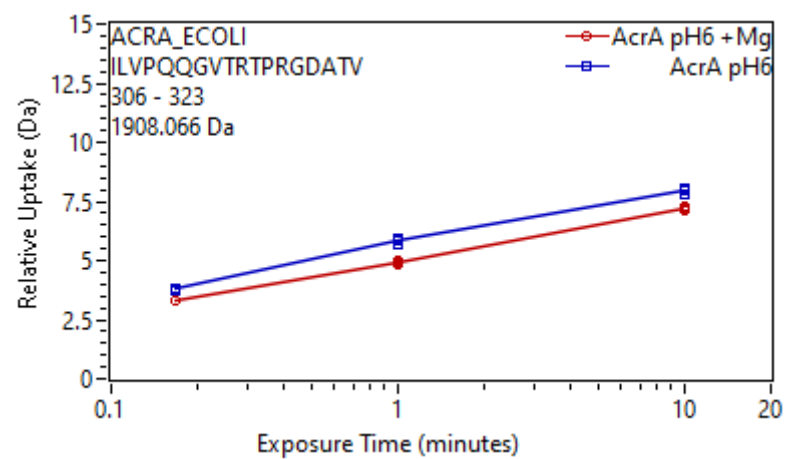

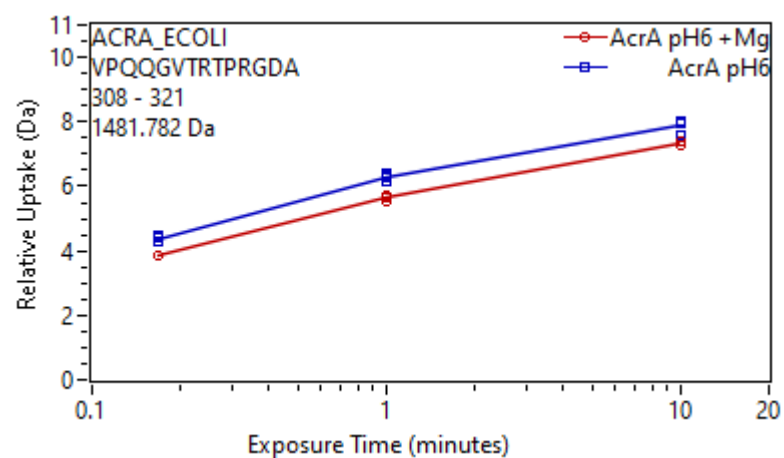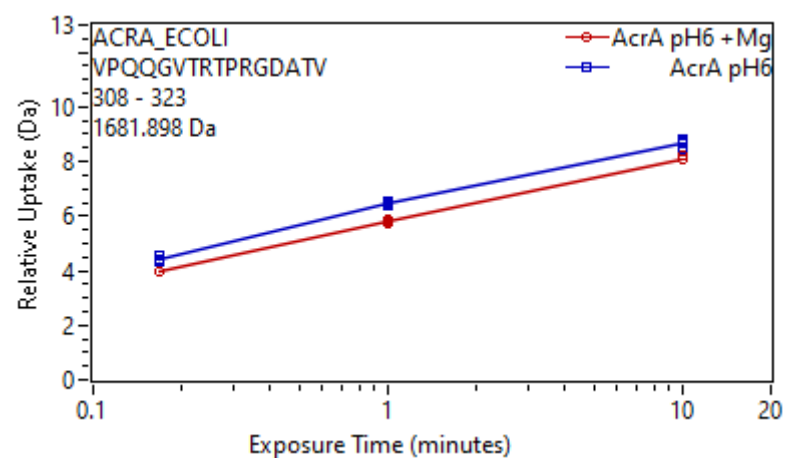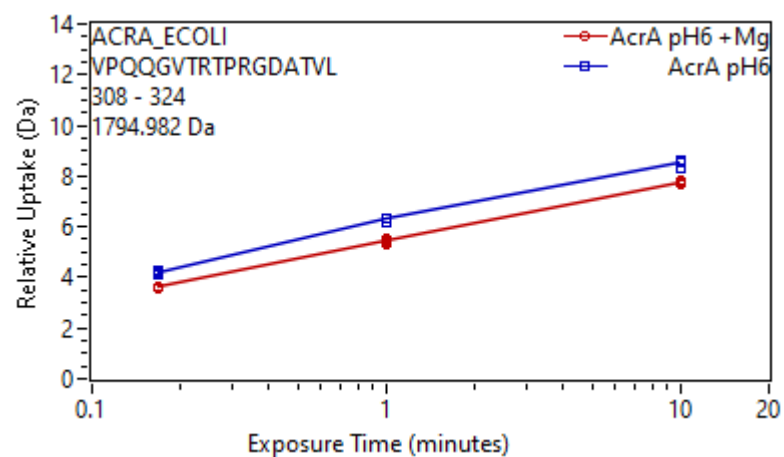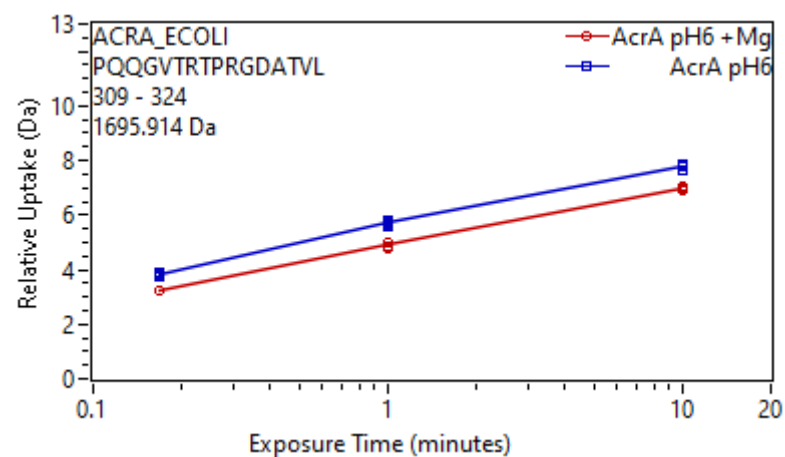

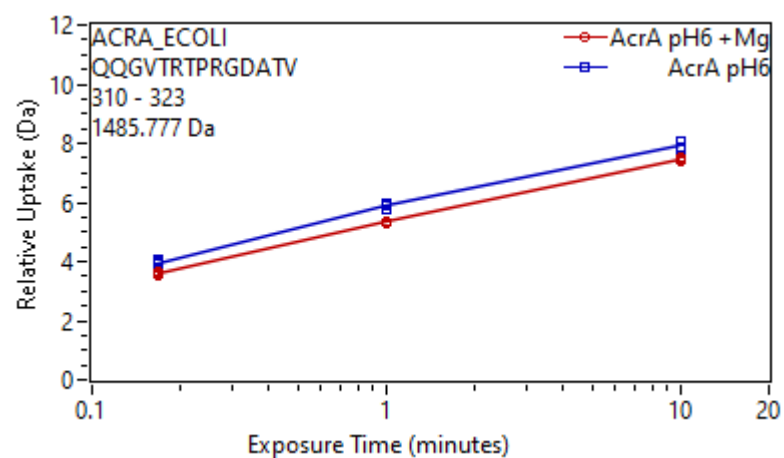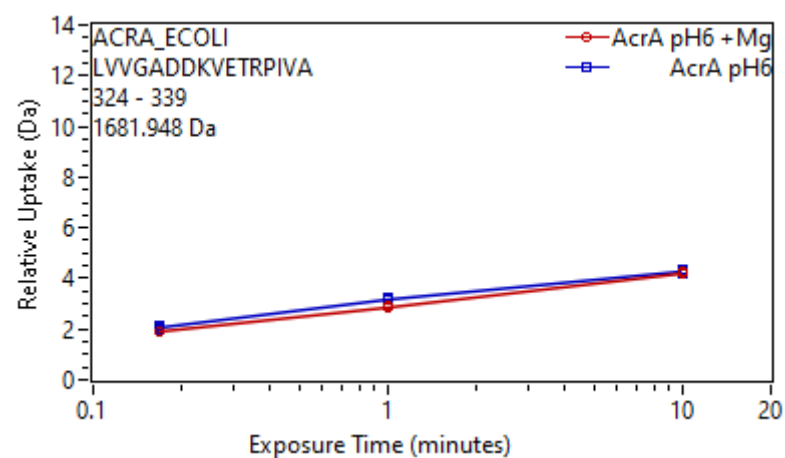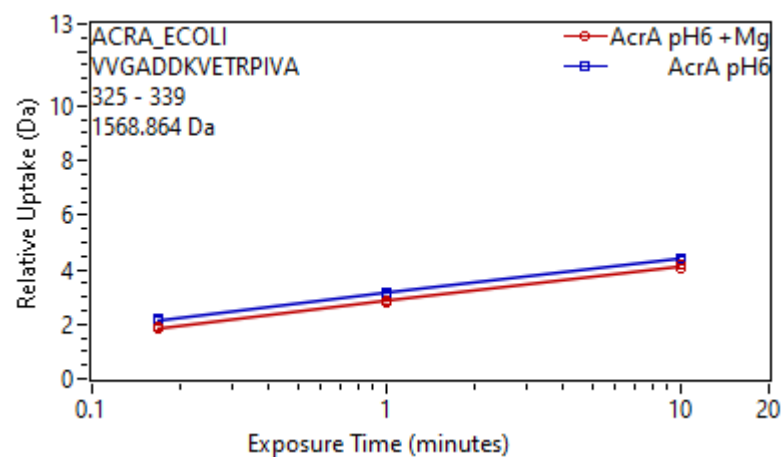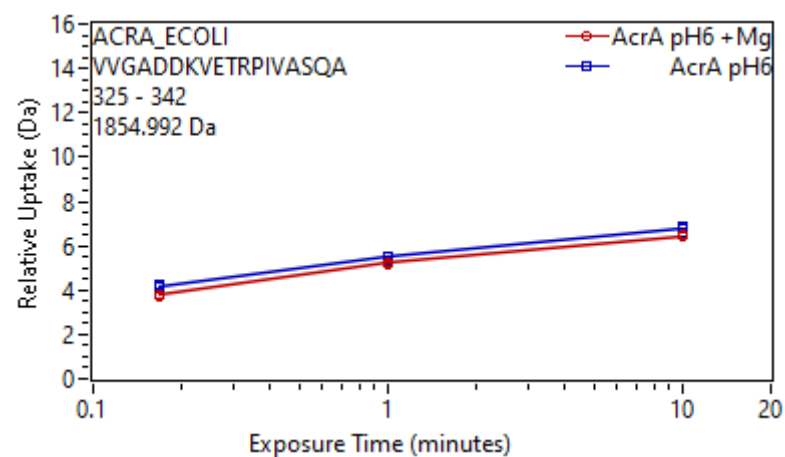

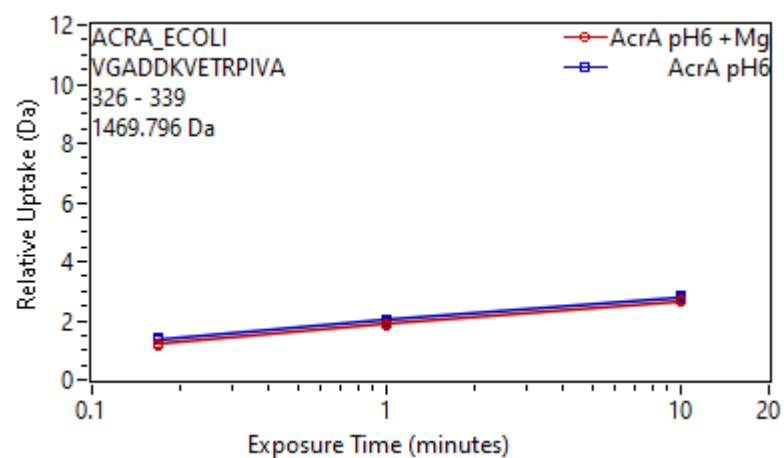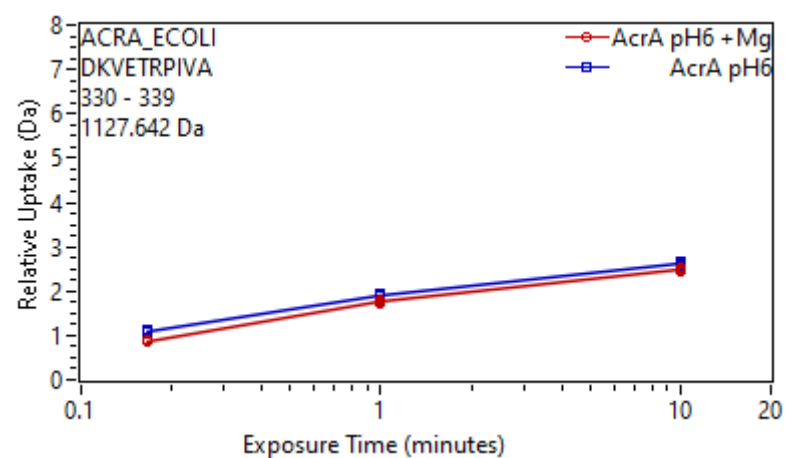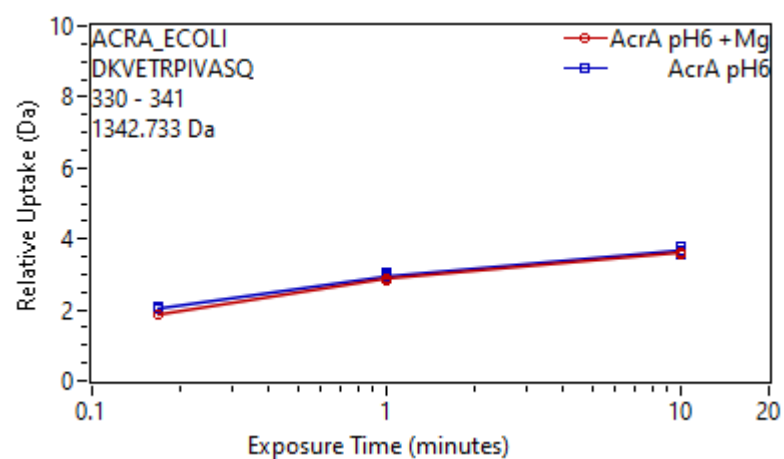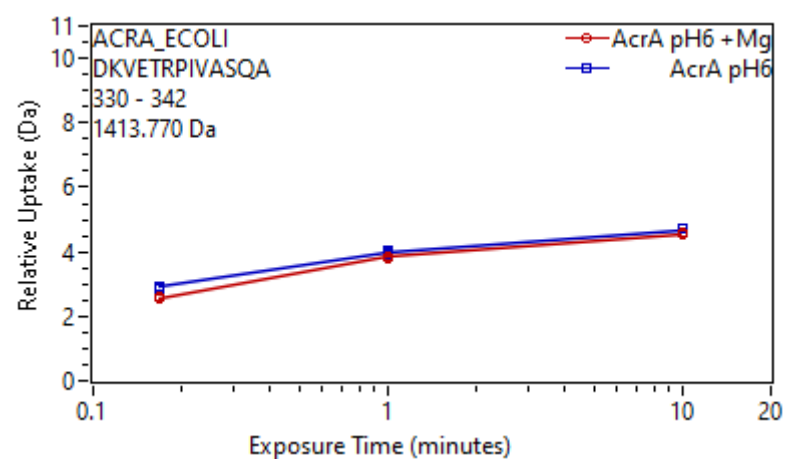

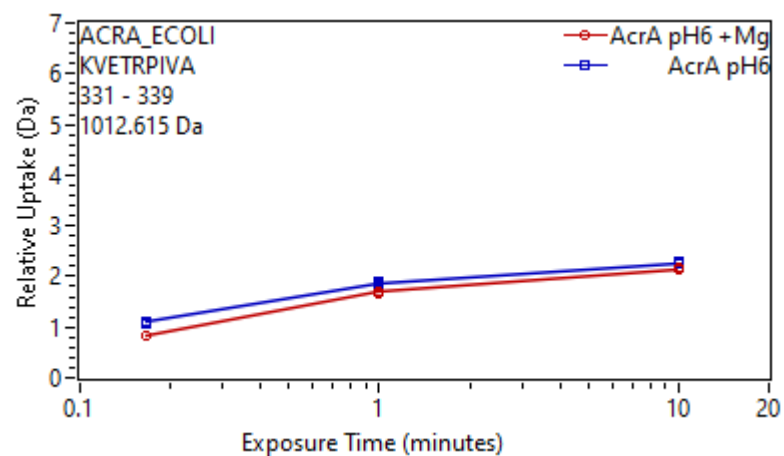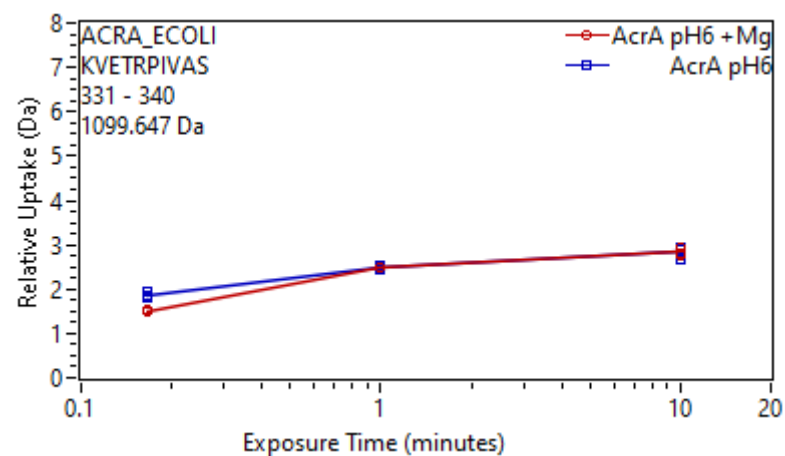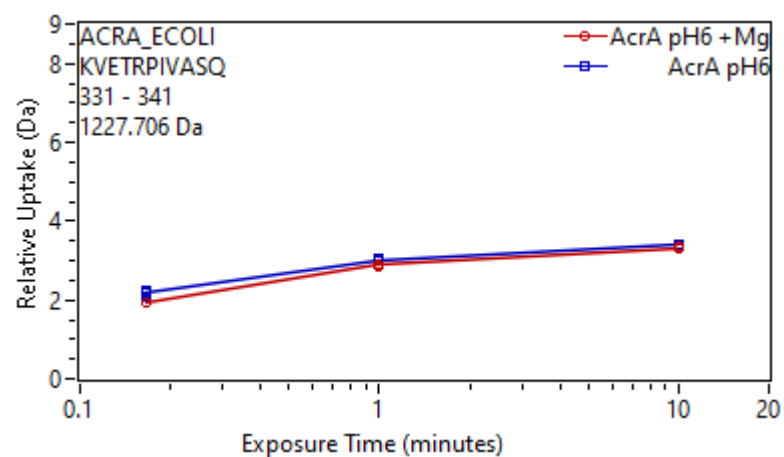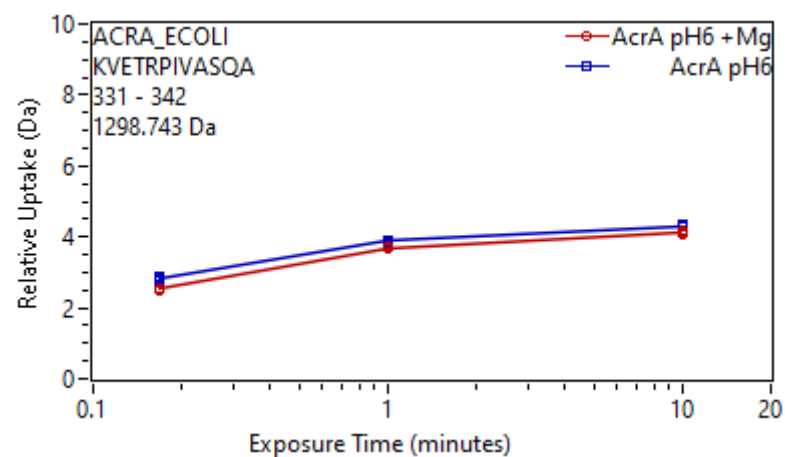

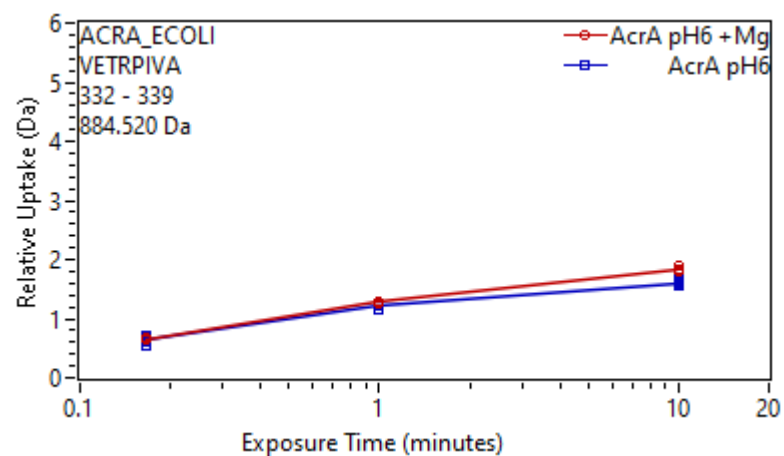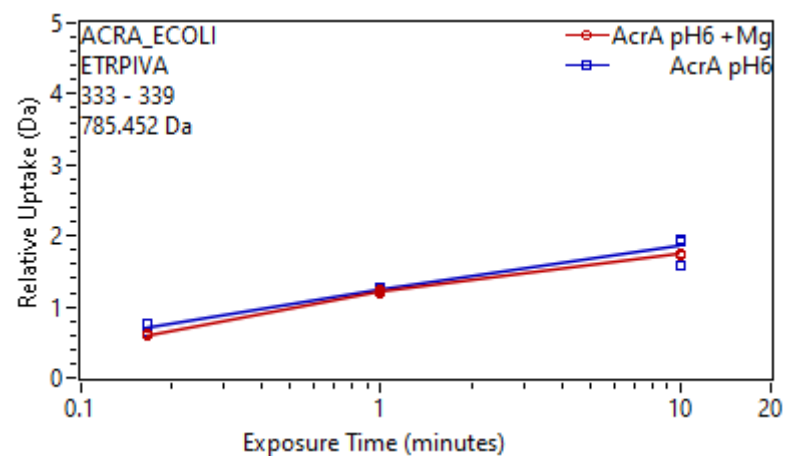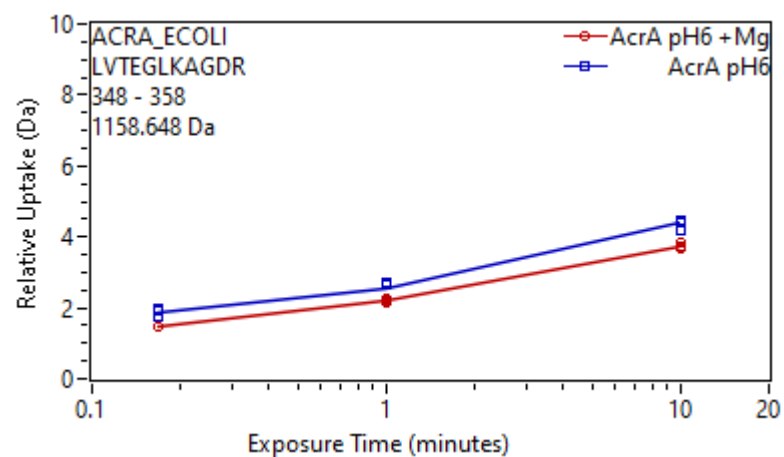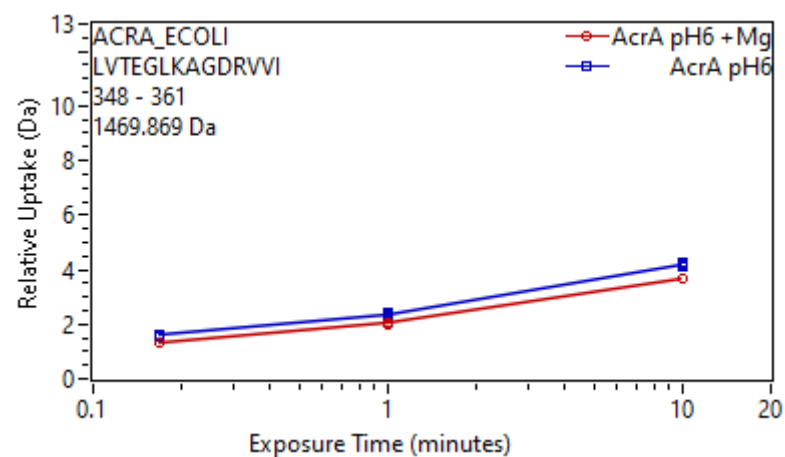

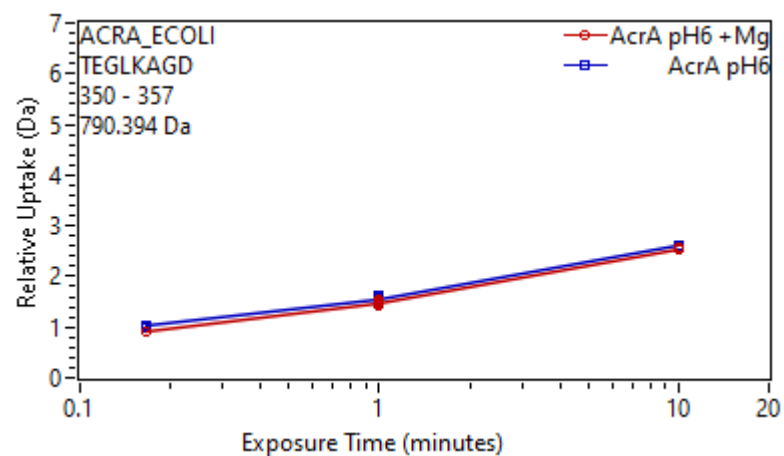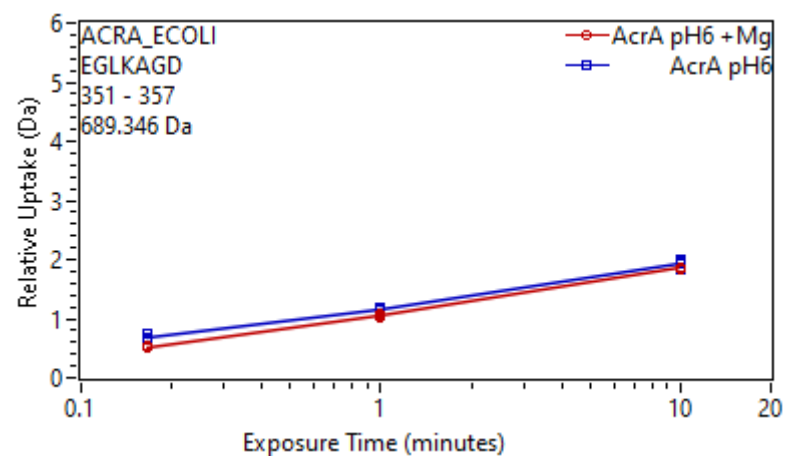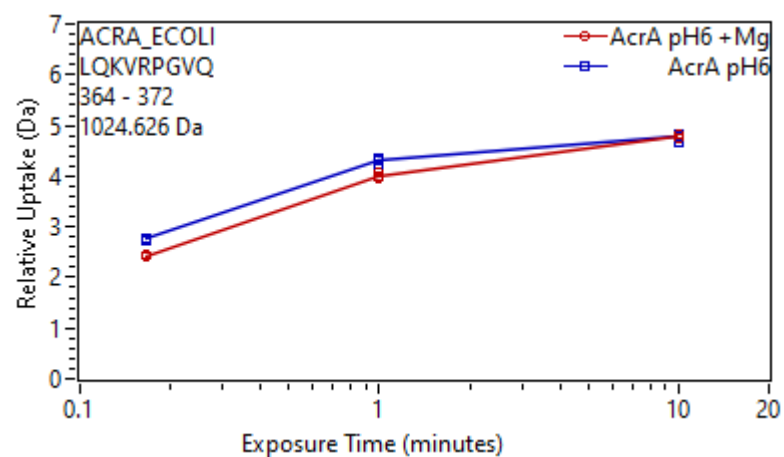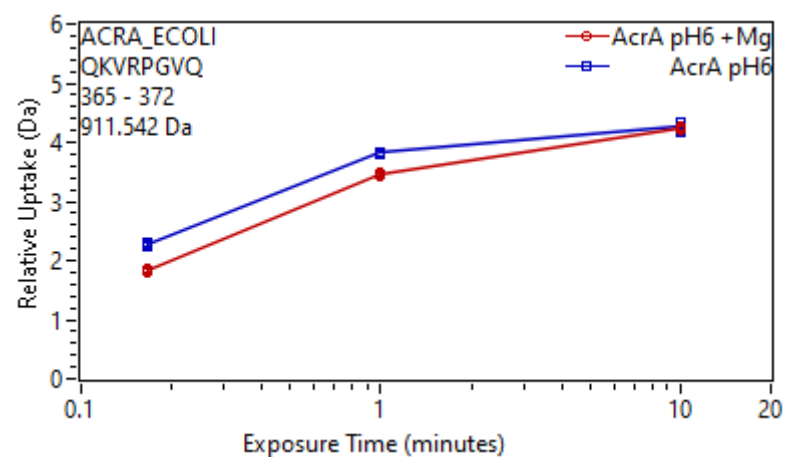

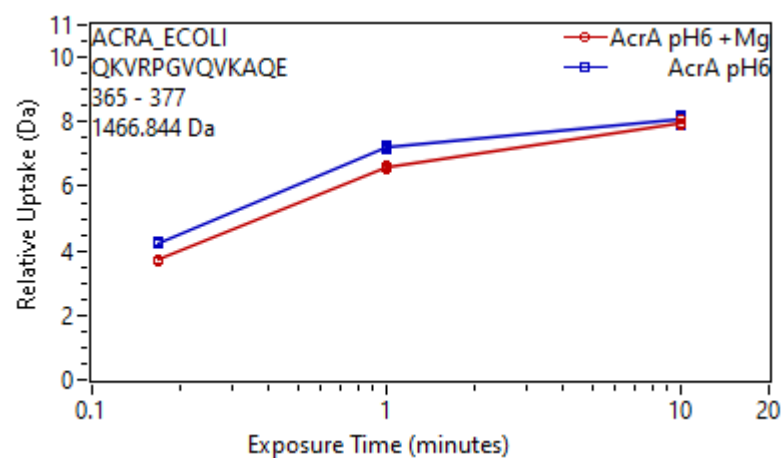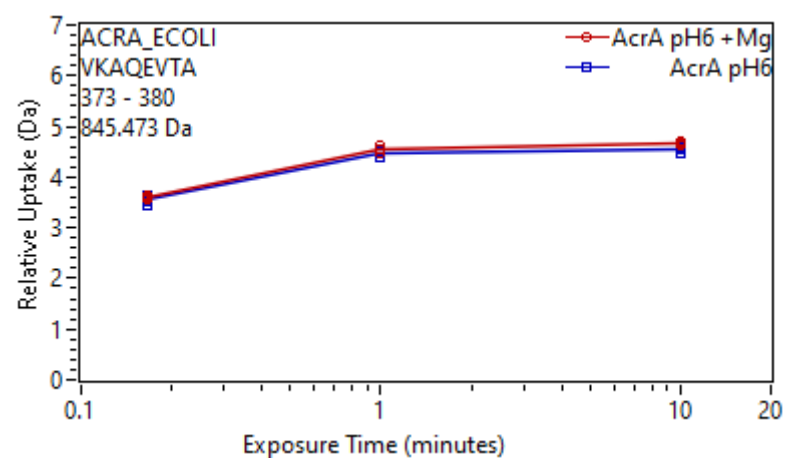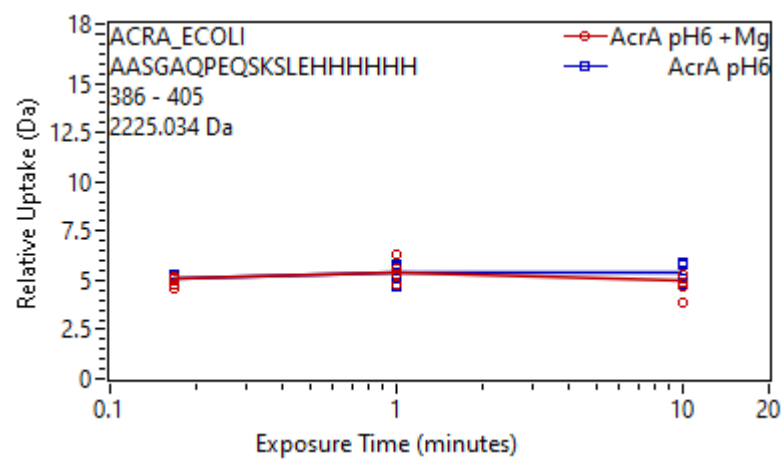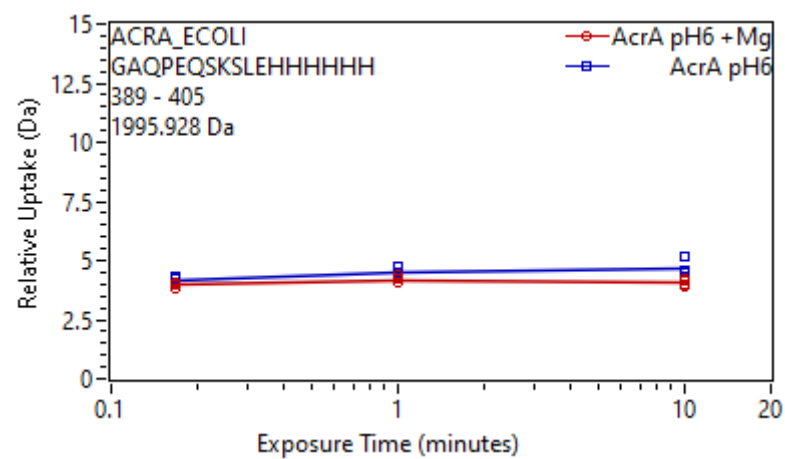

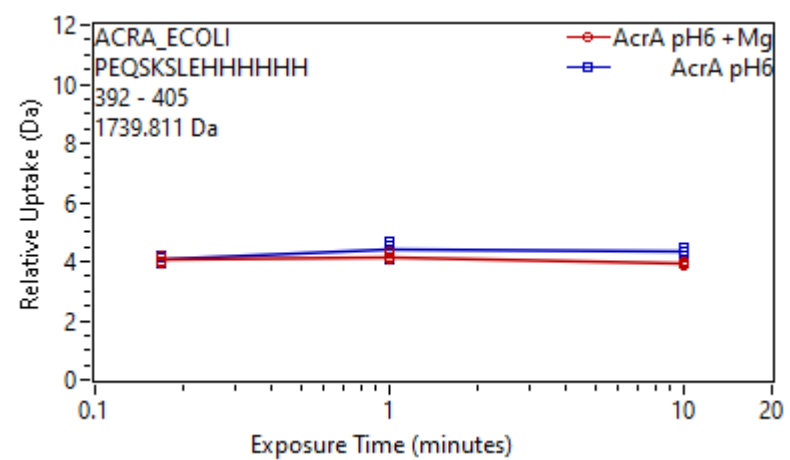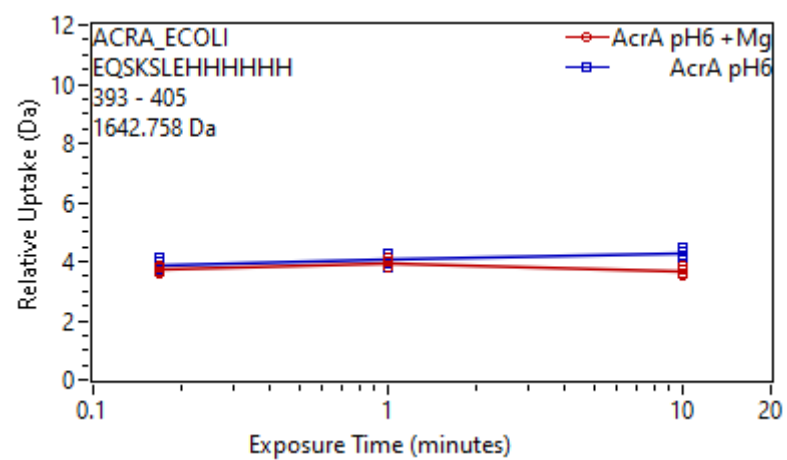

Supplement: Supplement 4 [file media-4.zip › Supplementary Data 3/Uptake plots 2.pdf]

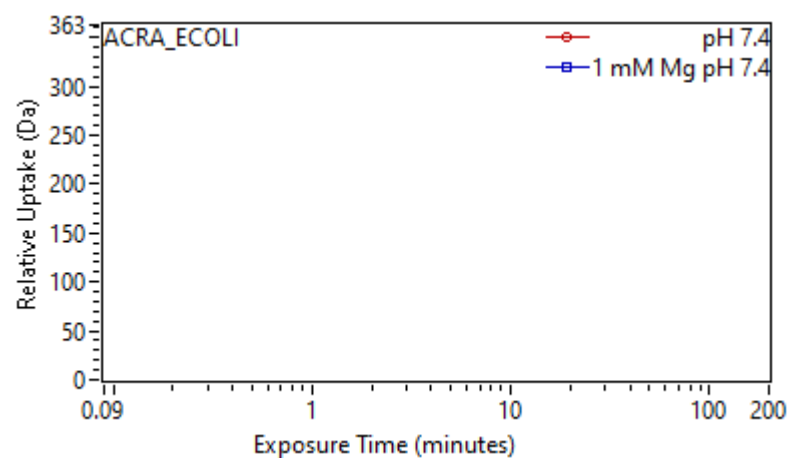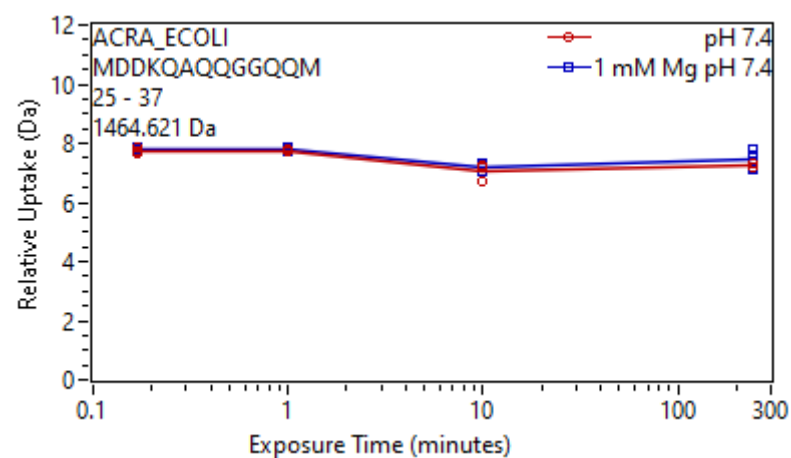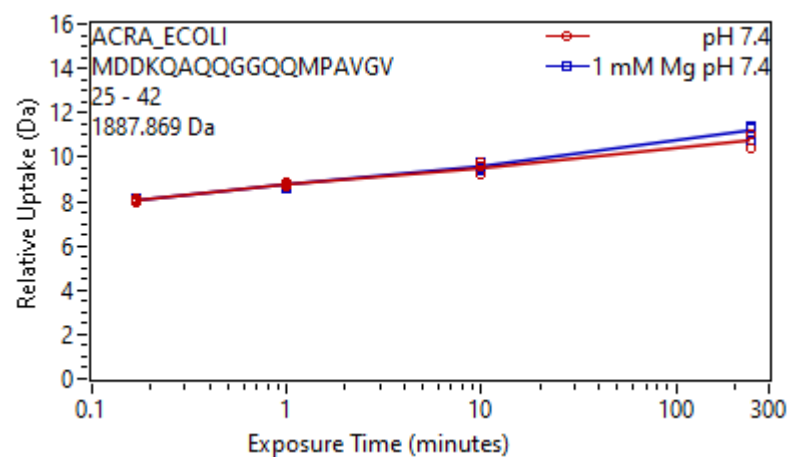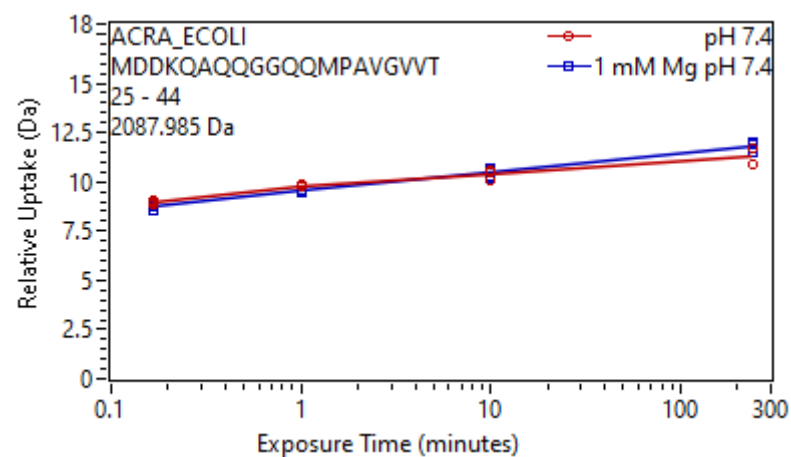

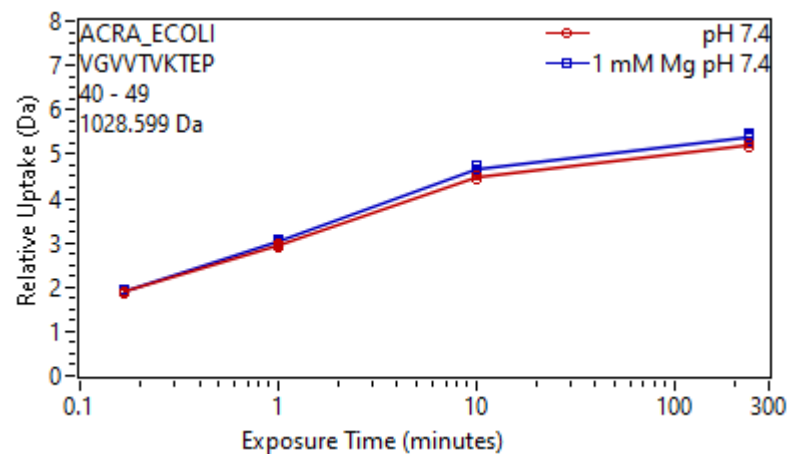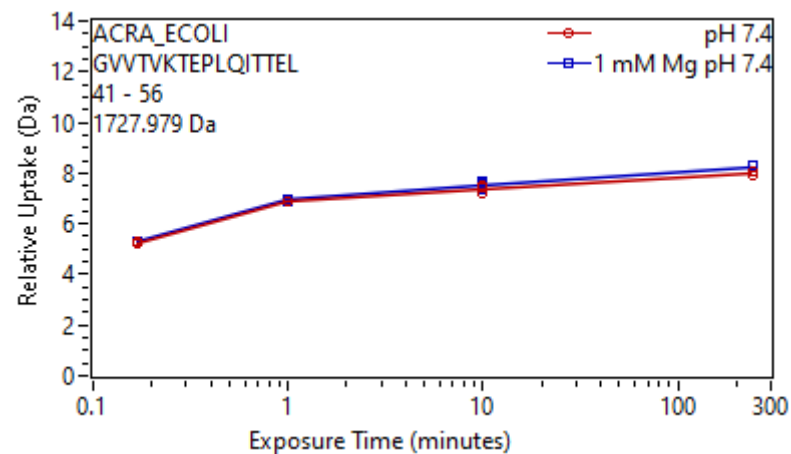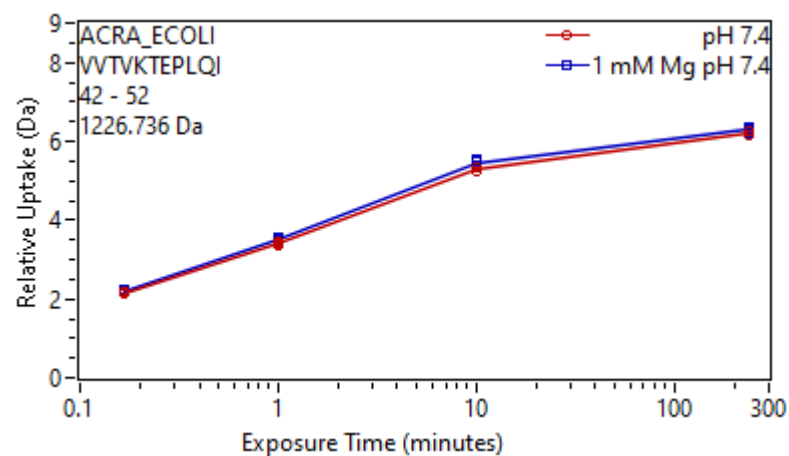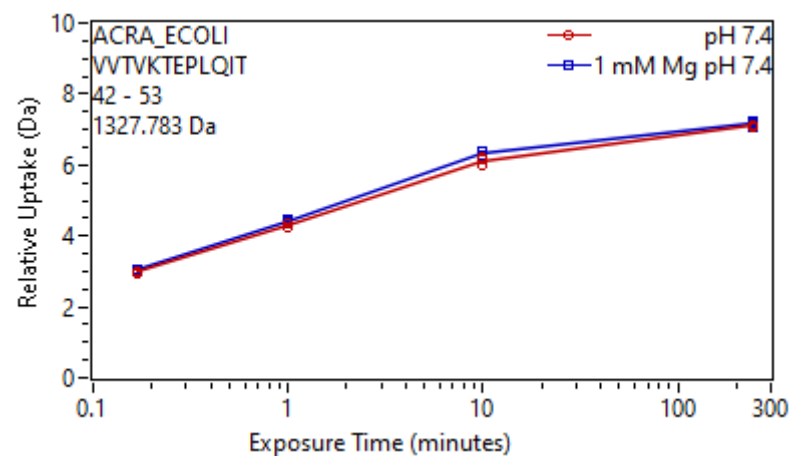

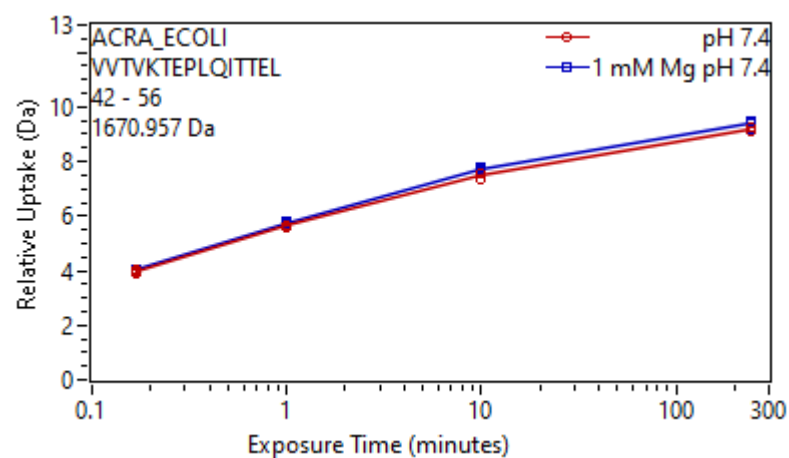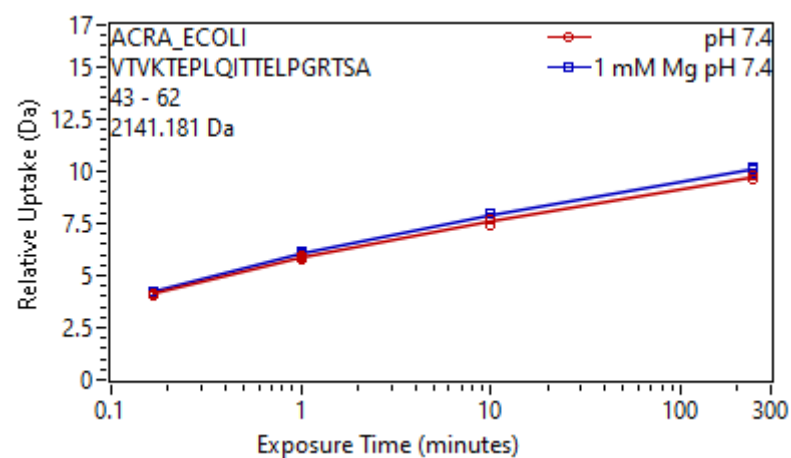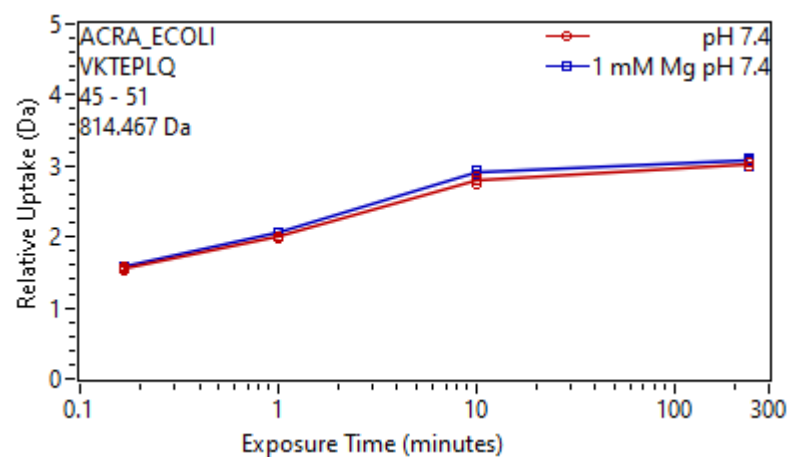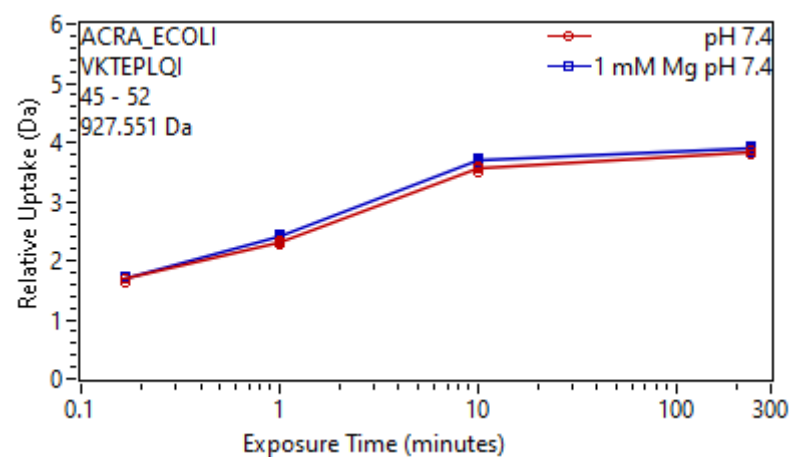

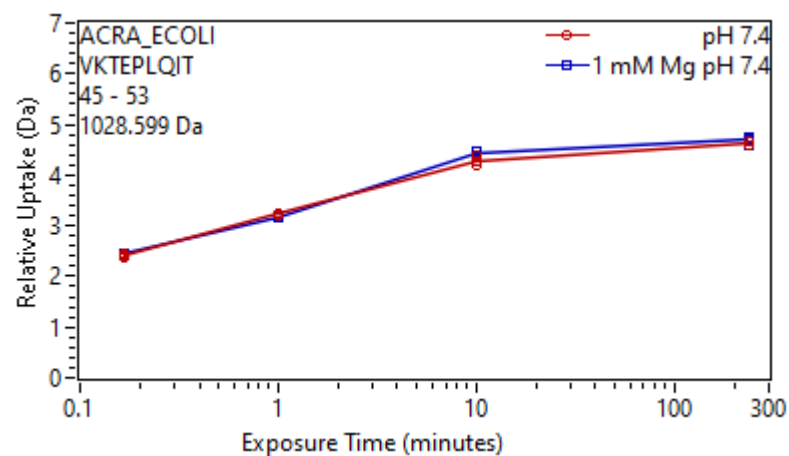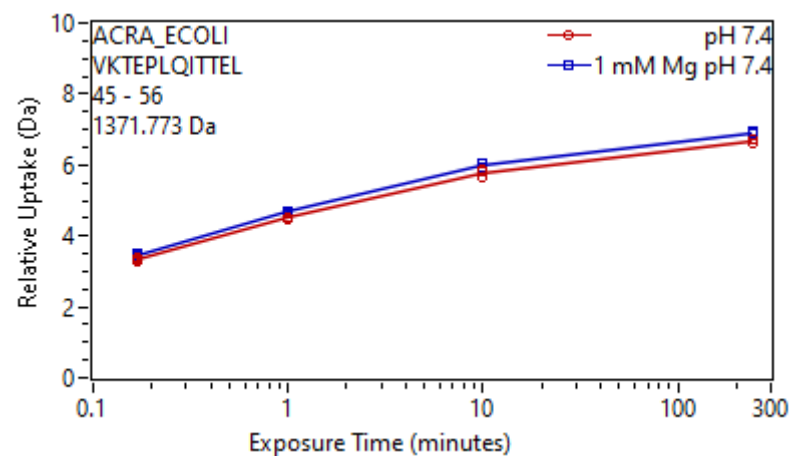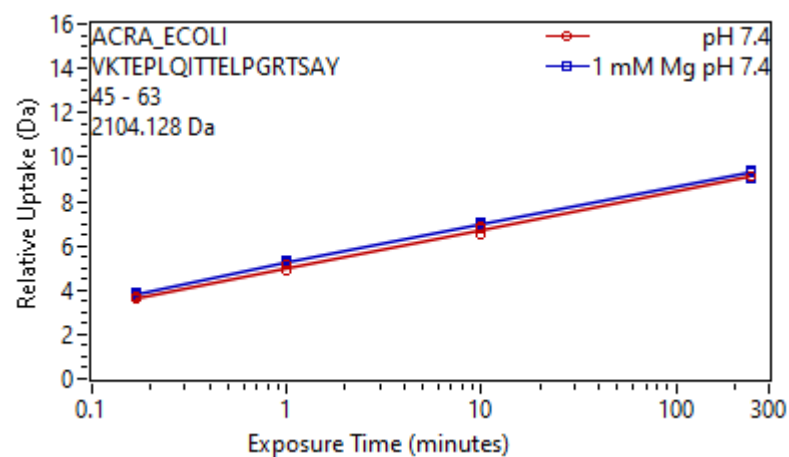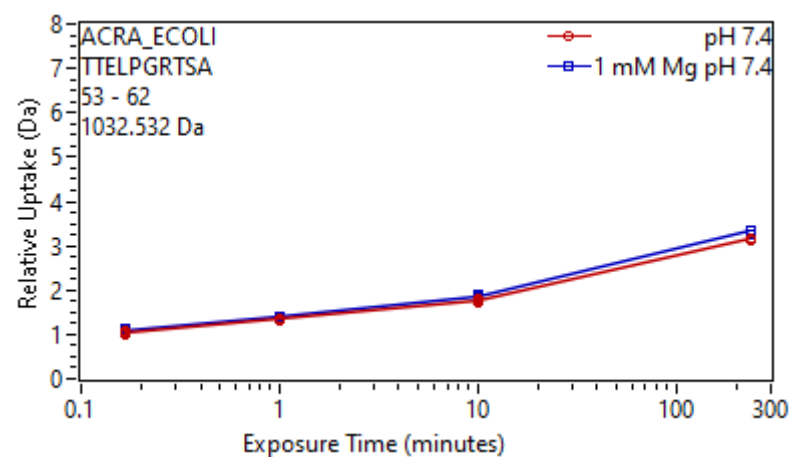

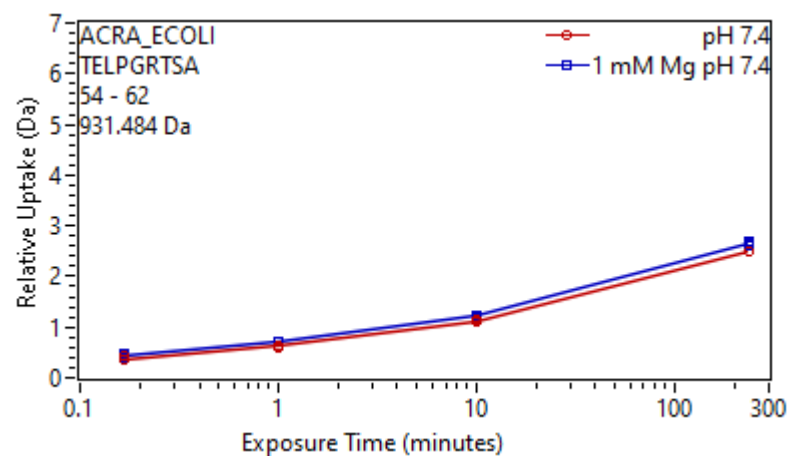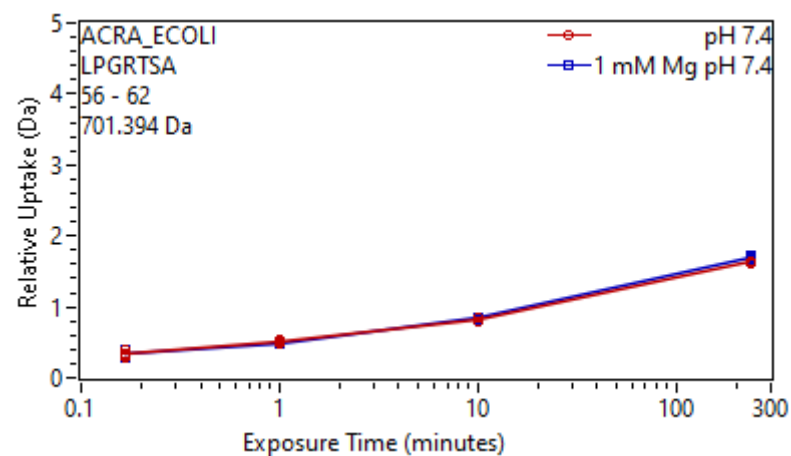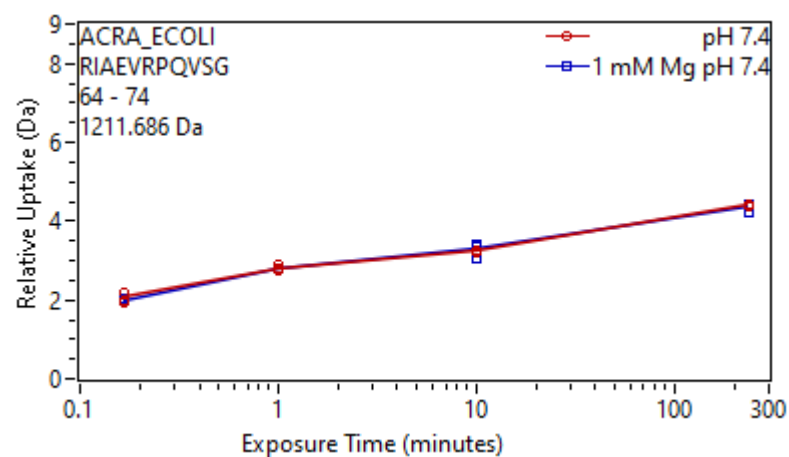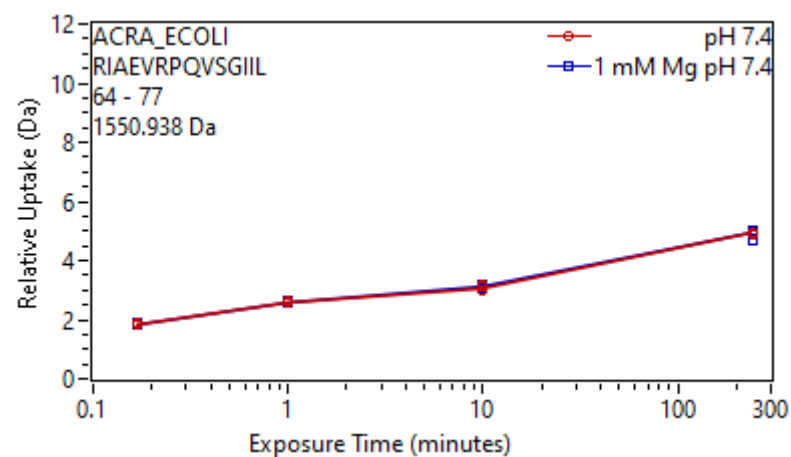

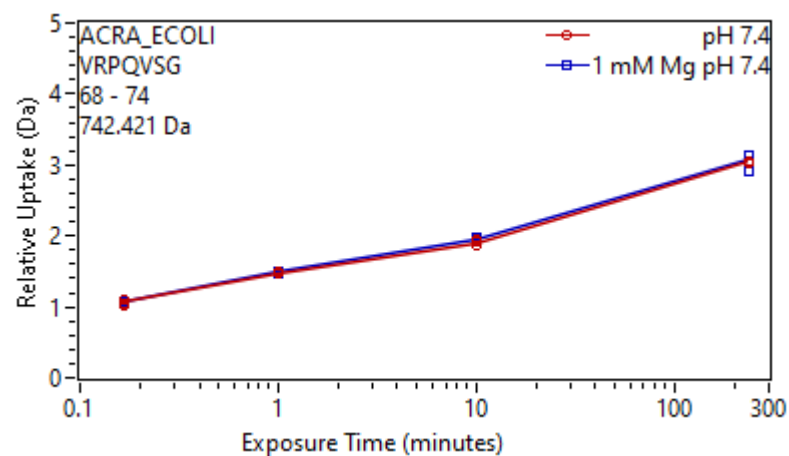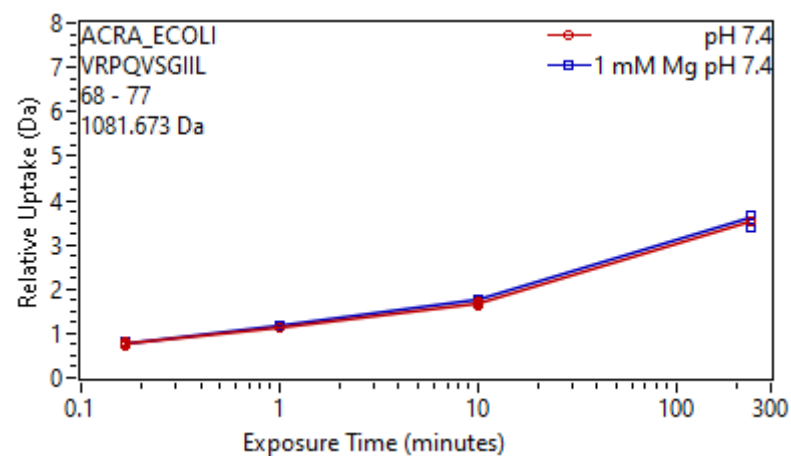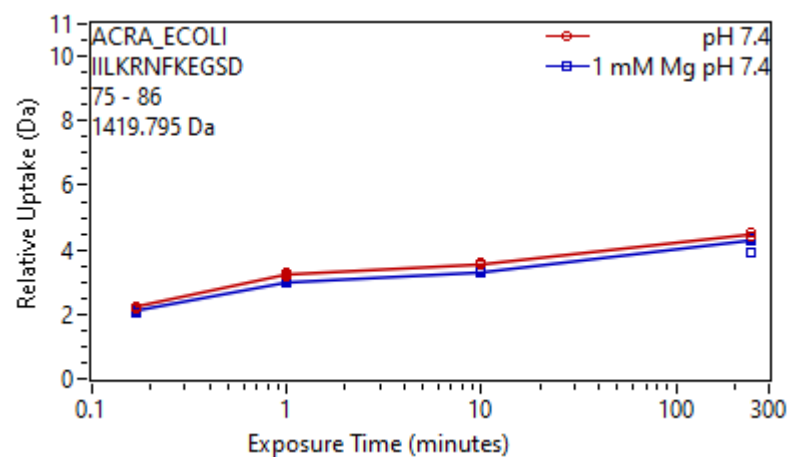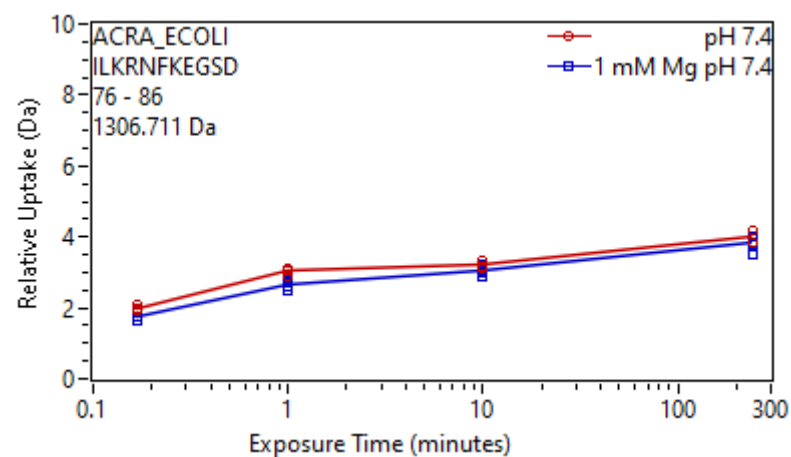

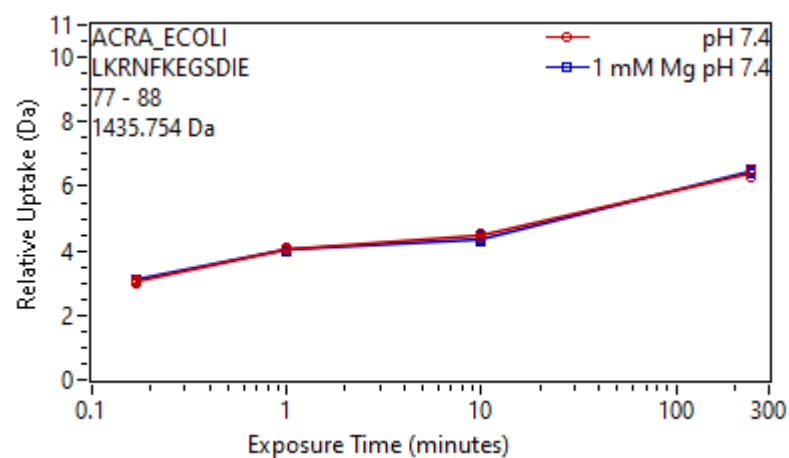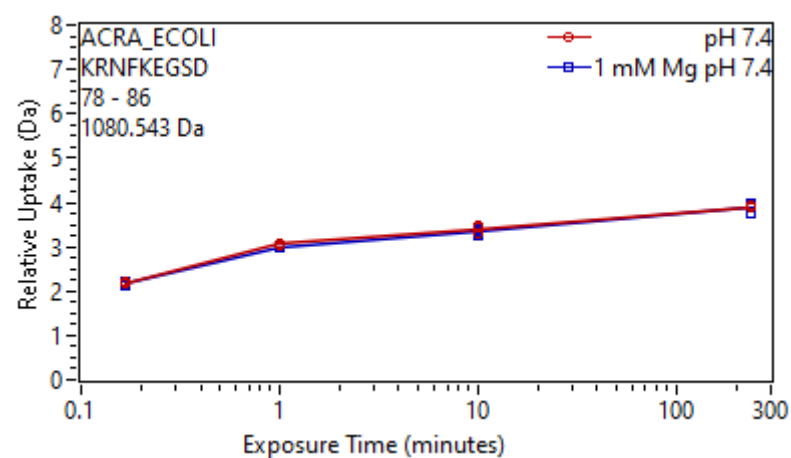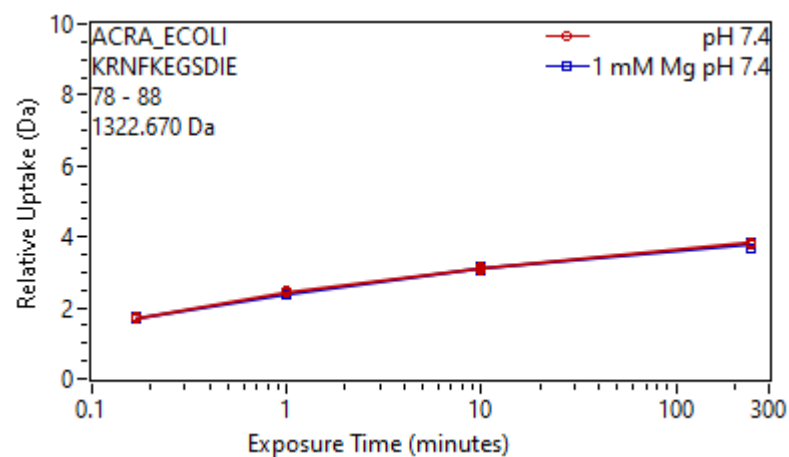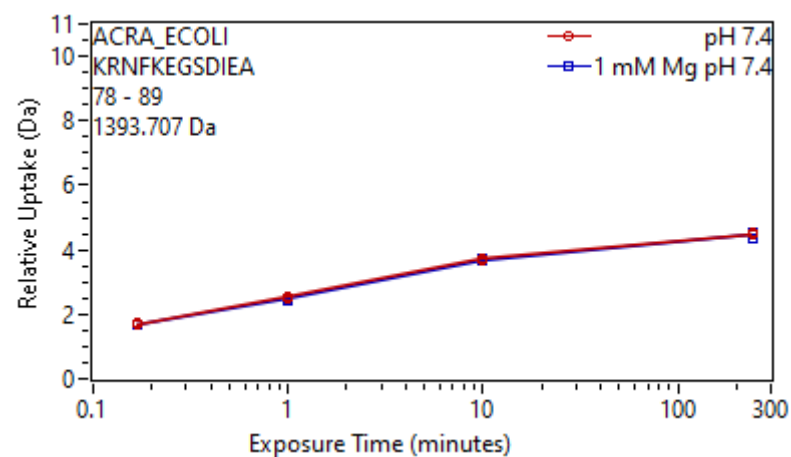

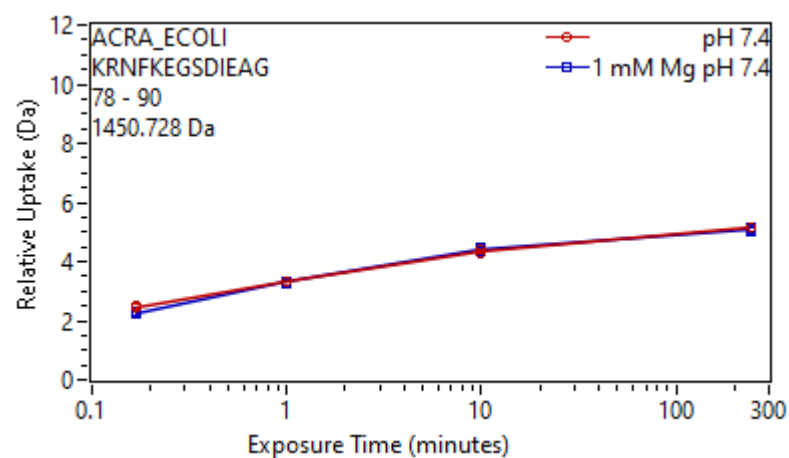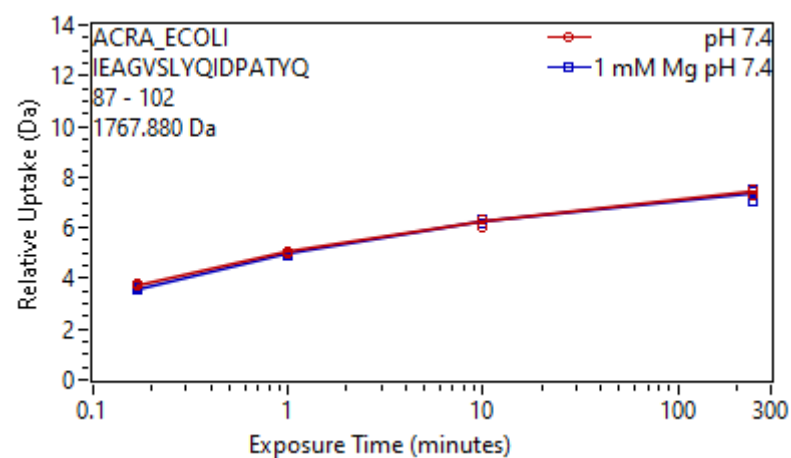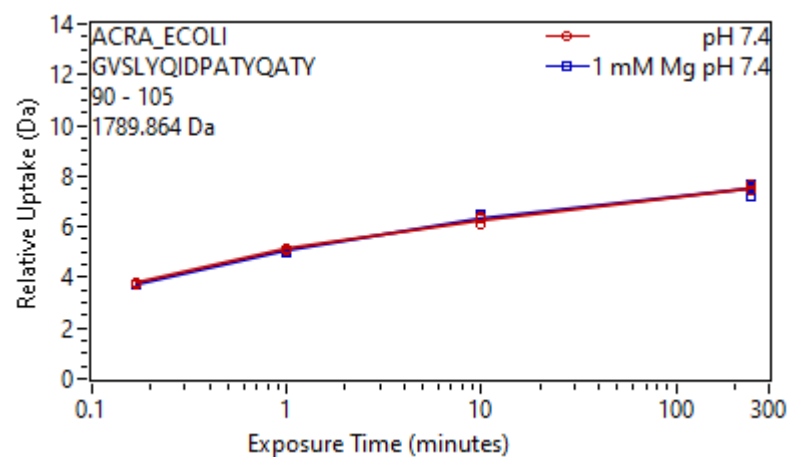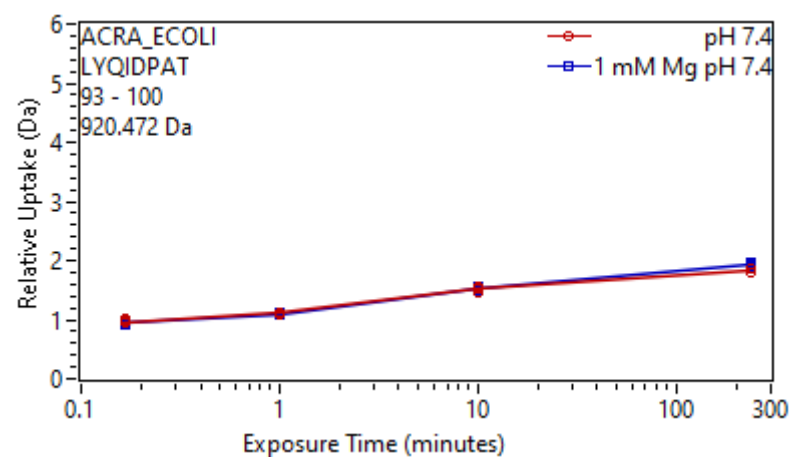

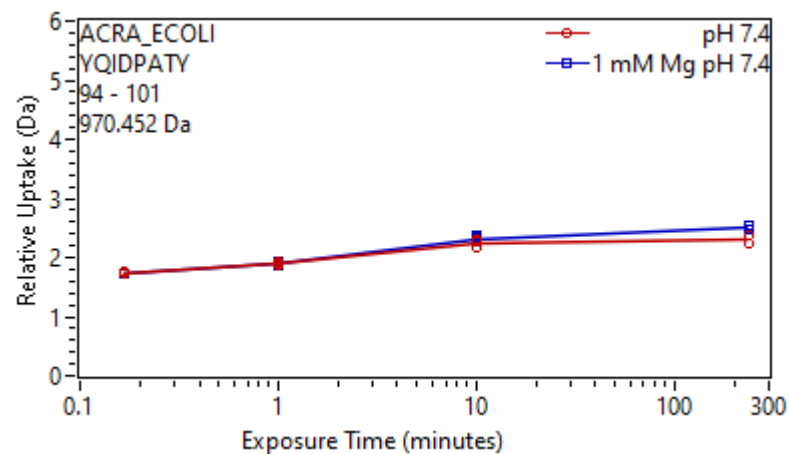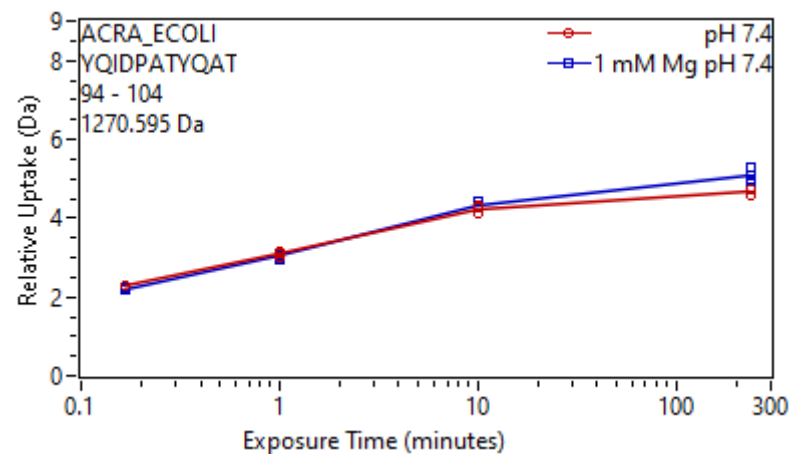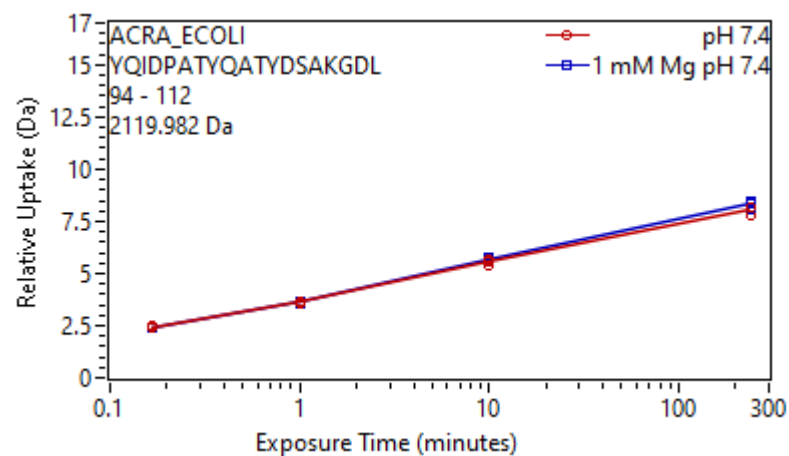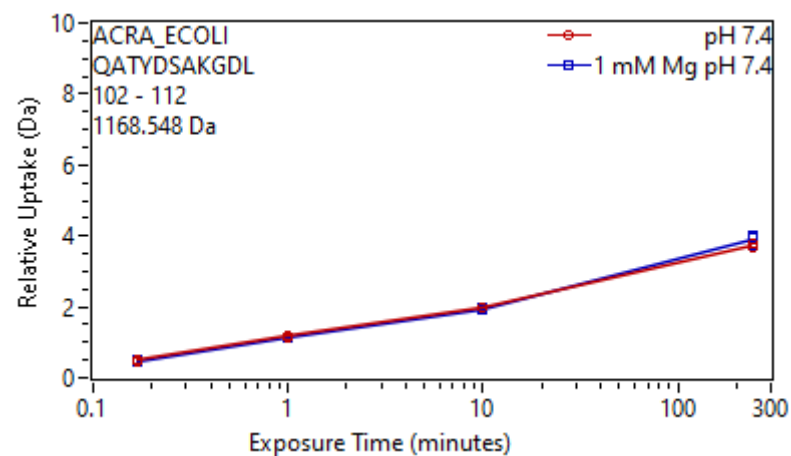

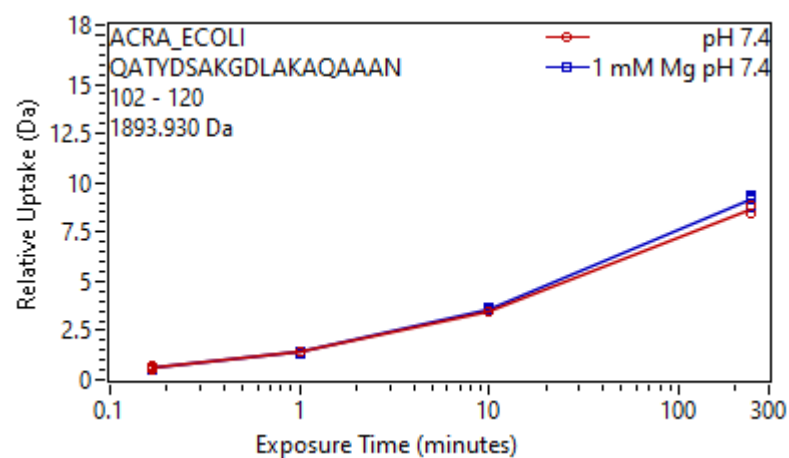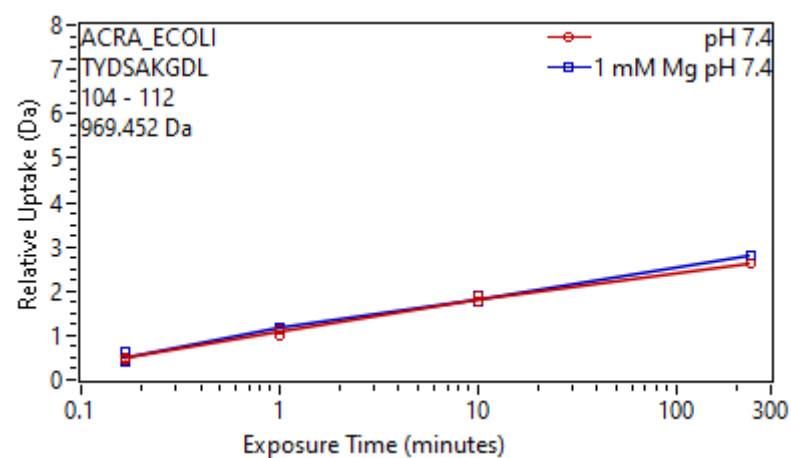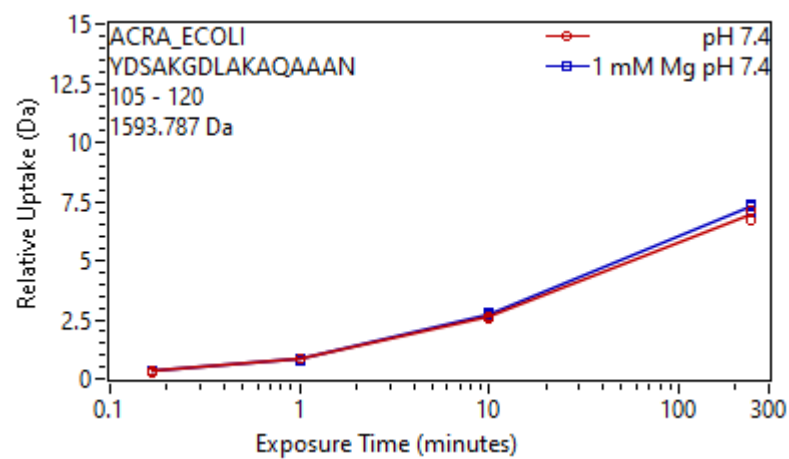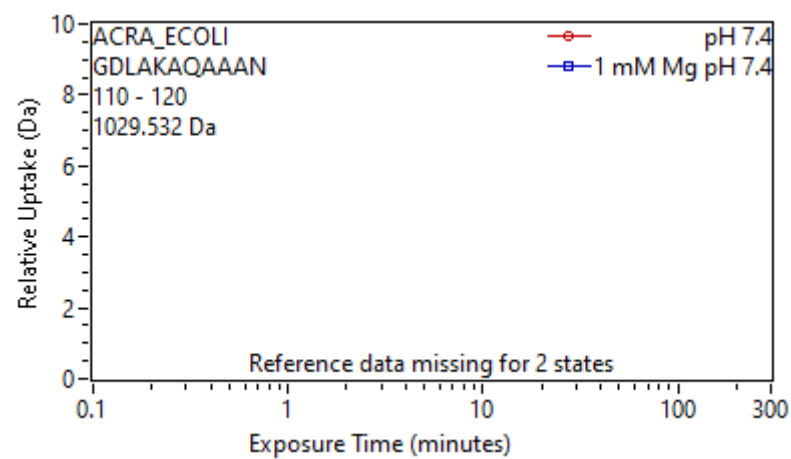

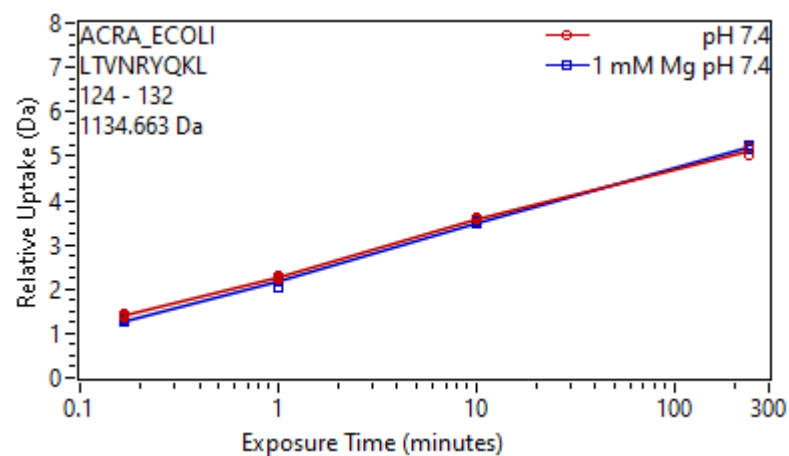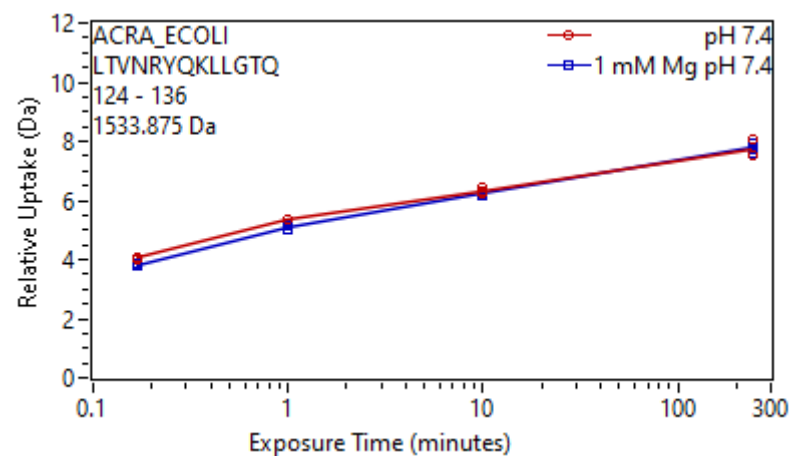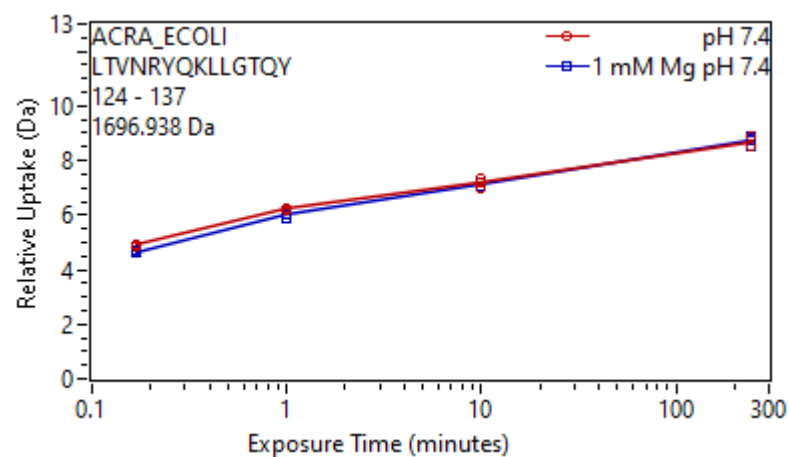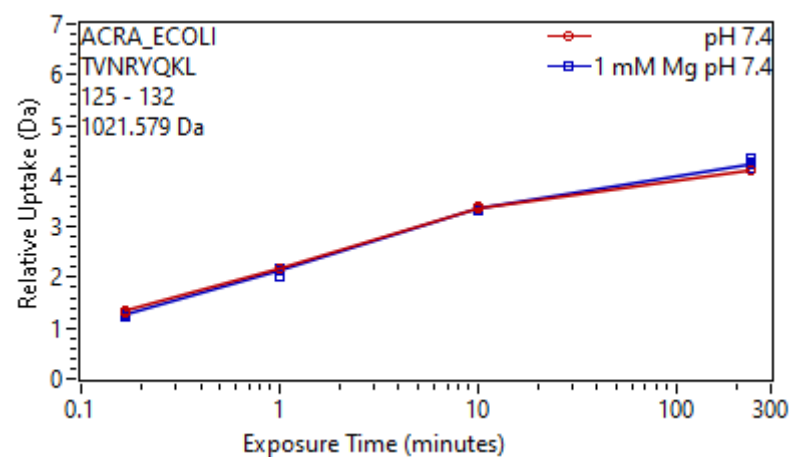

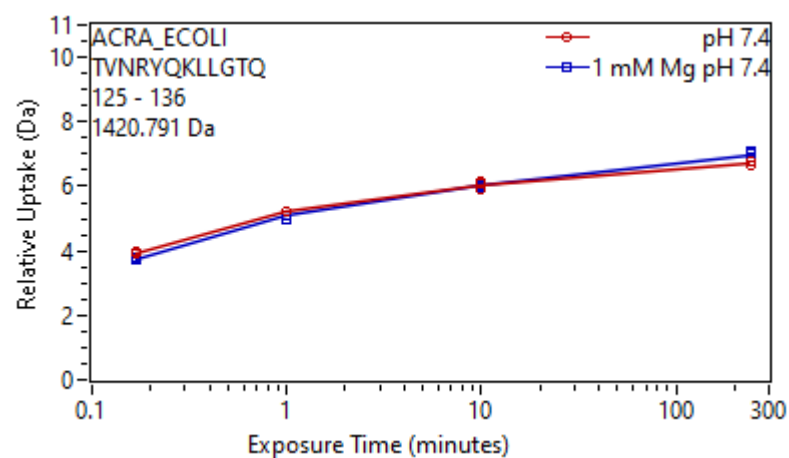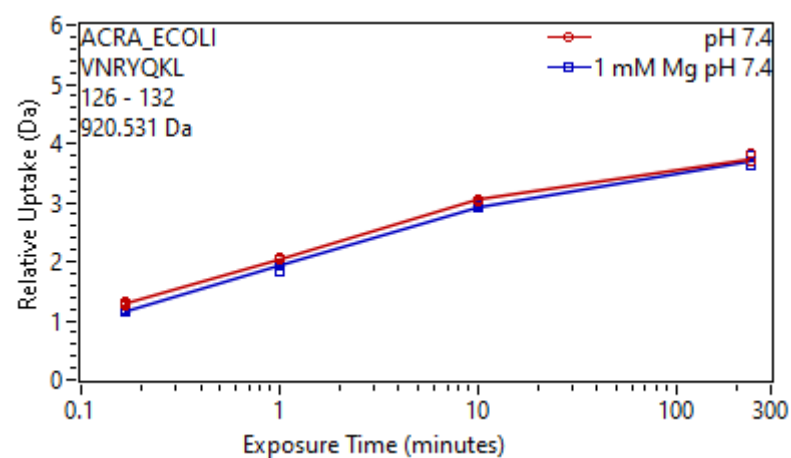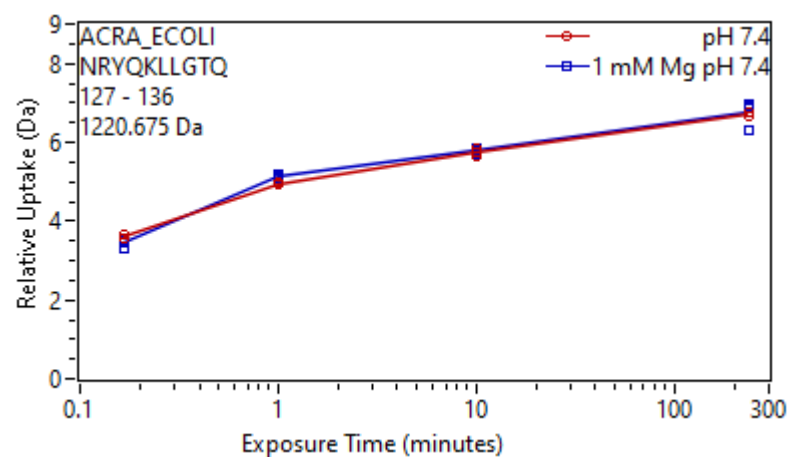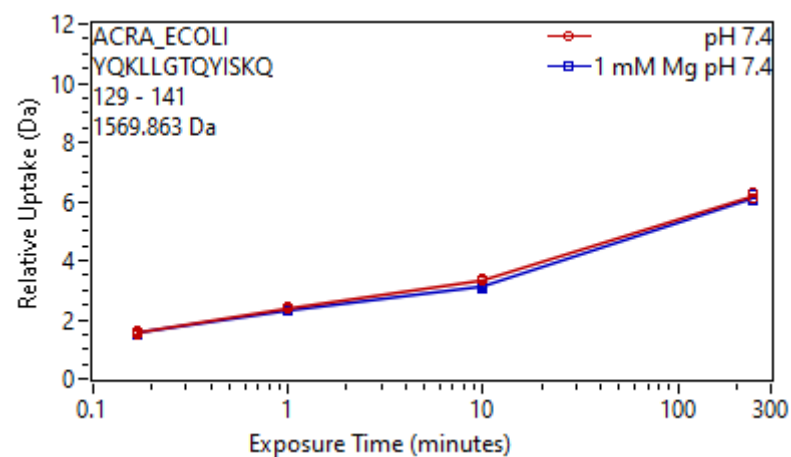

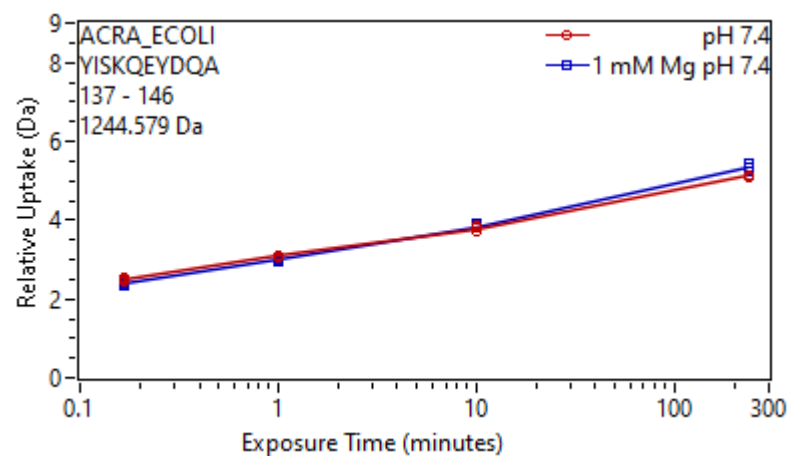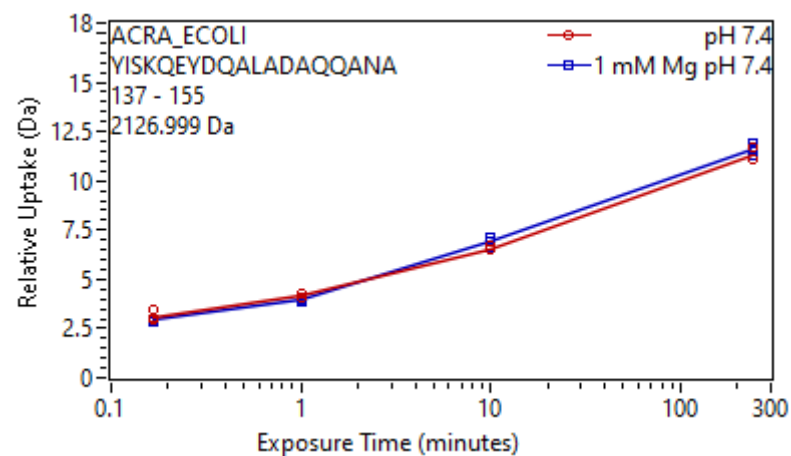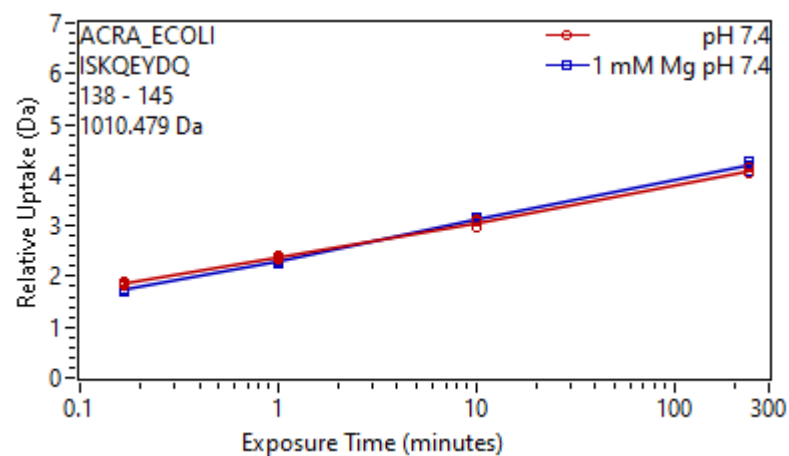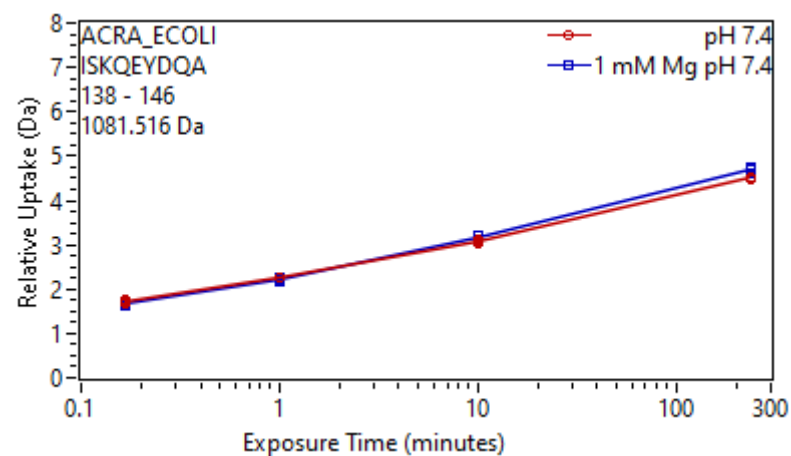

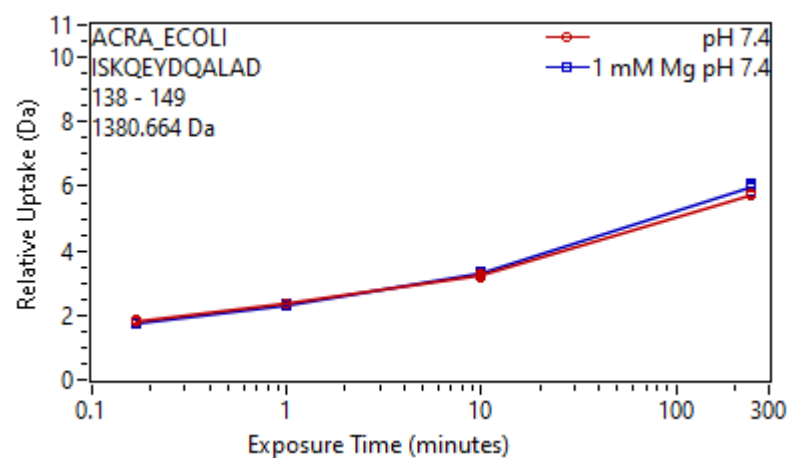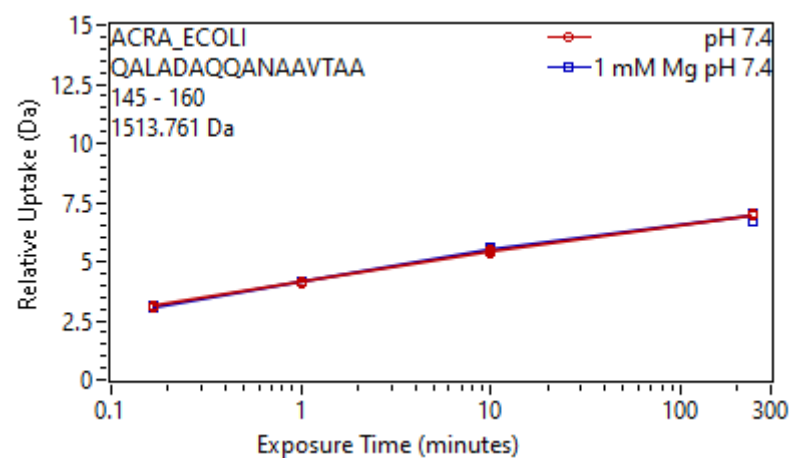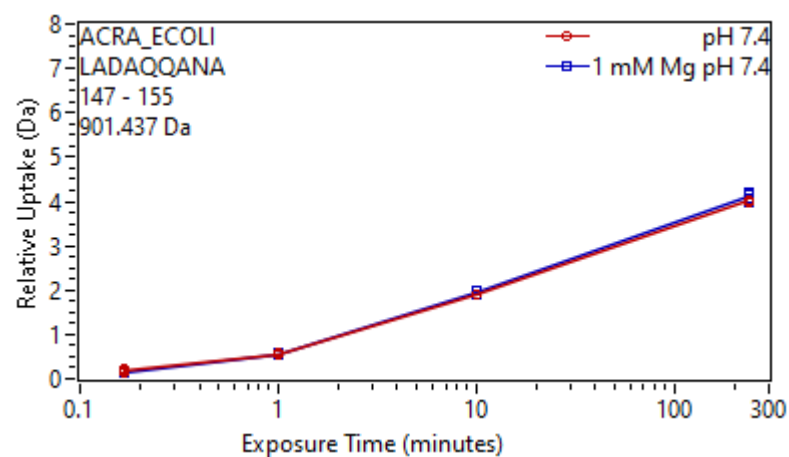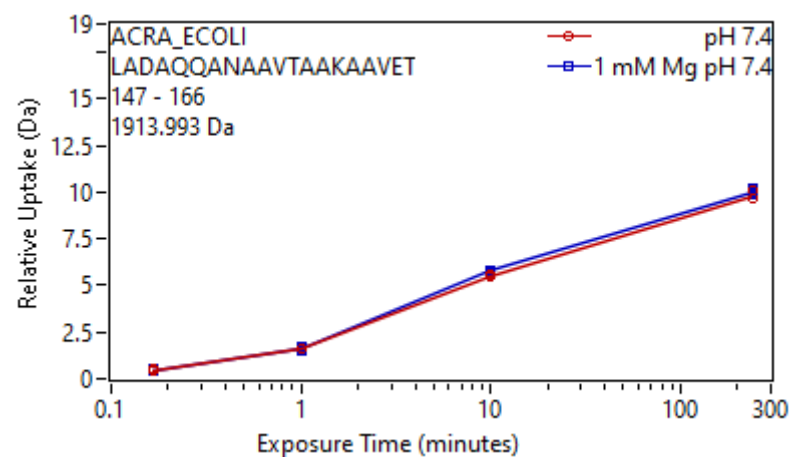

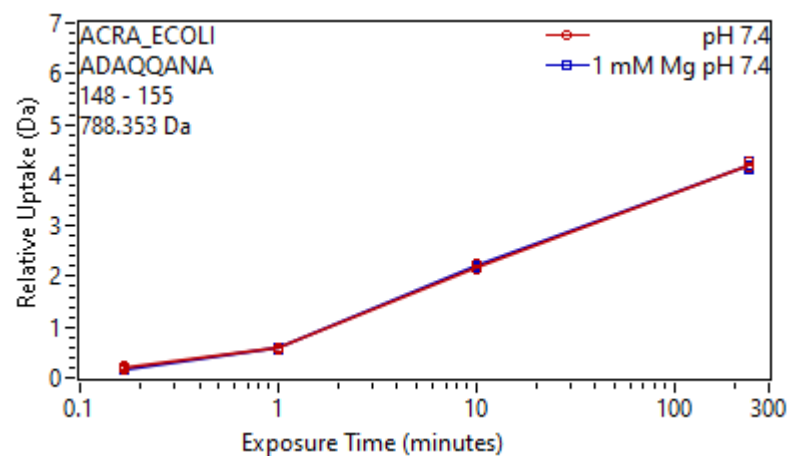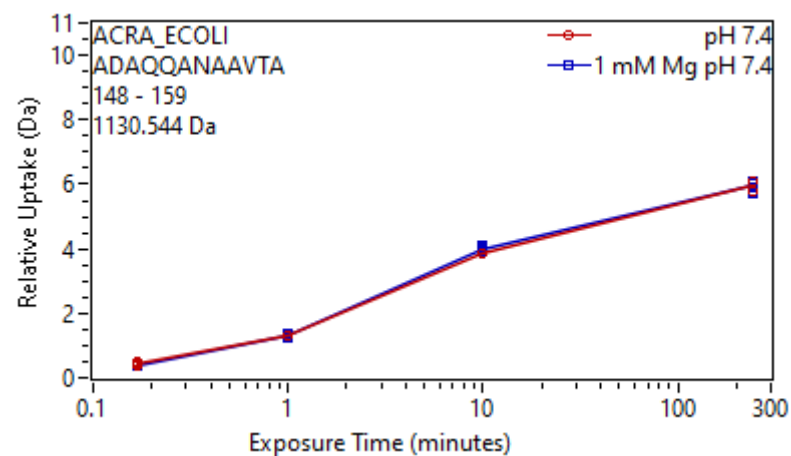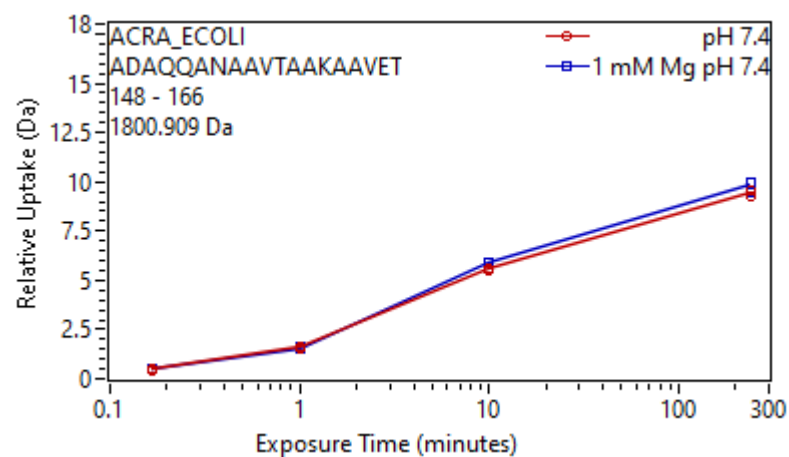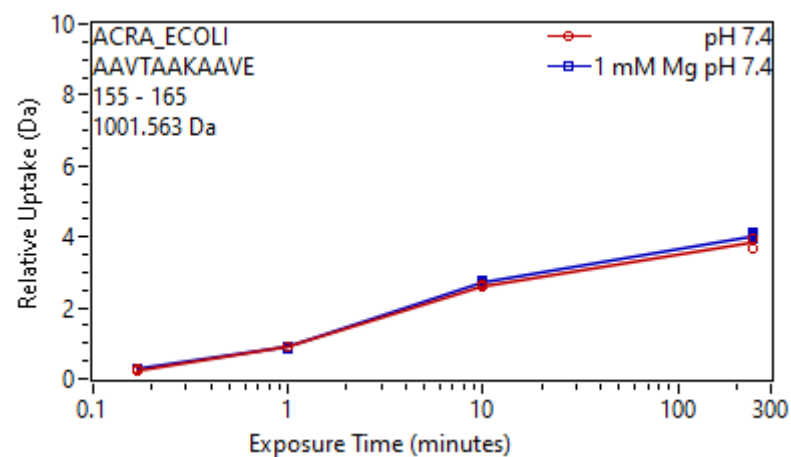

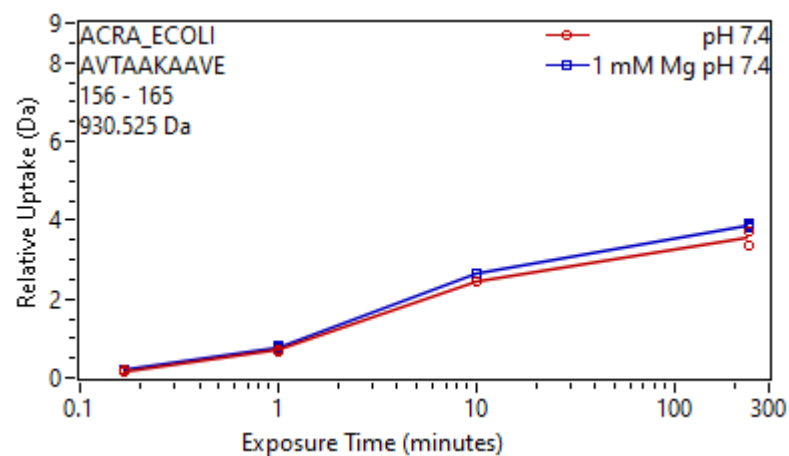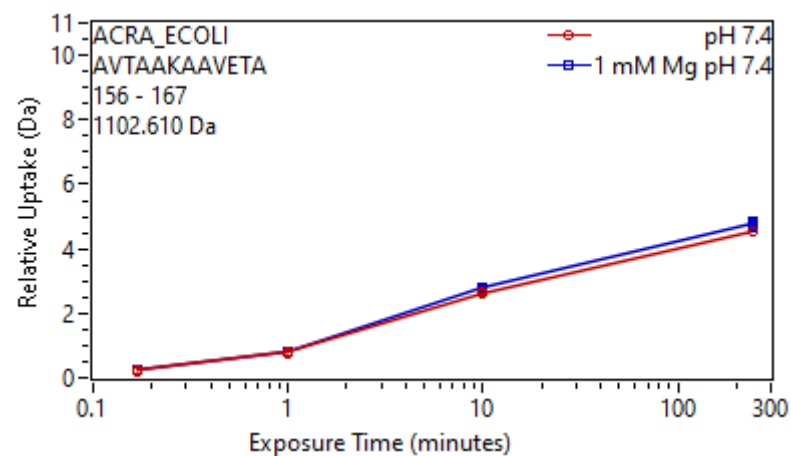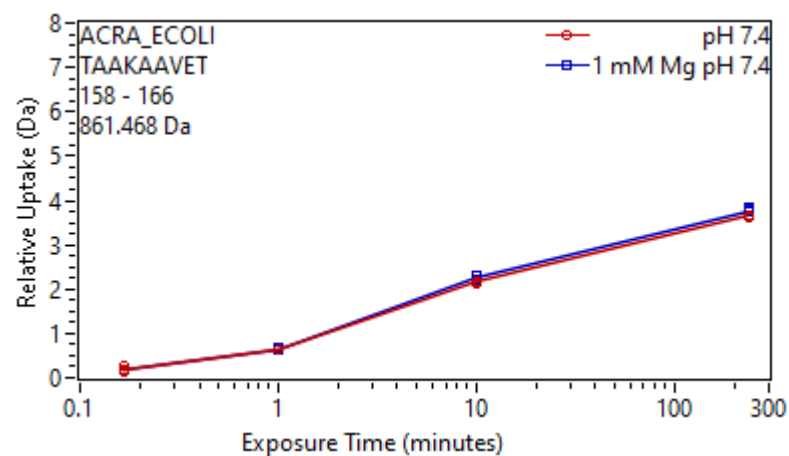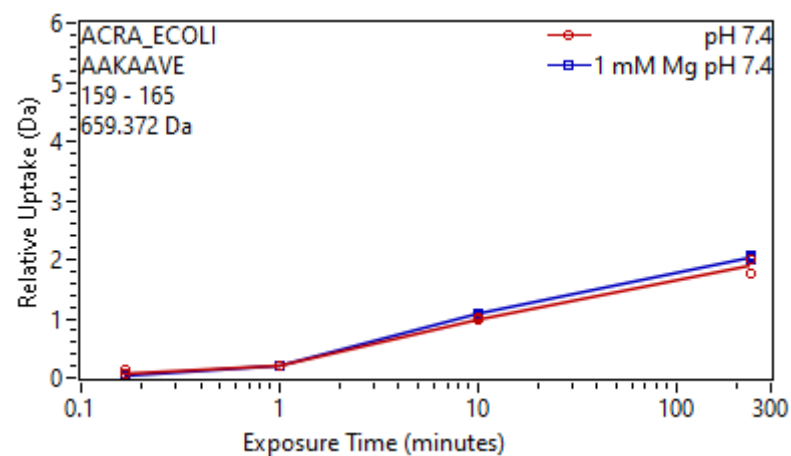

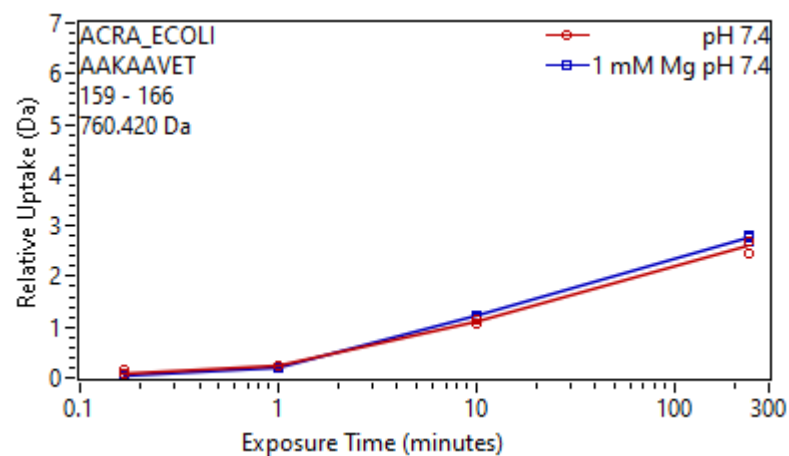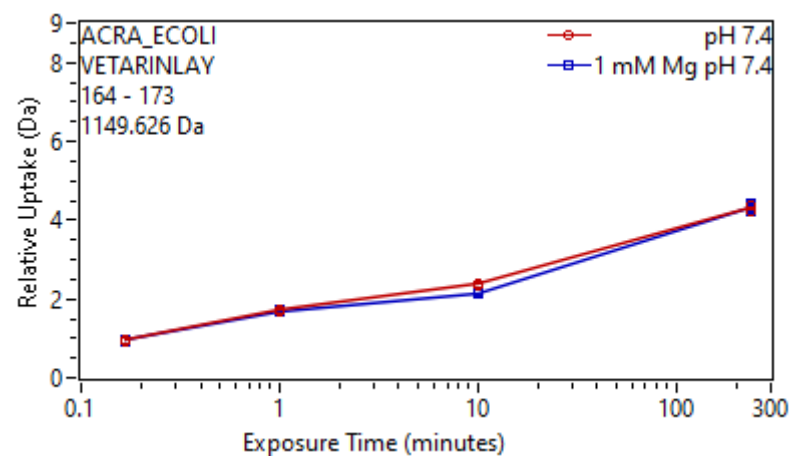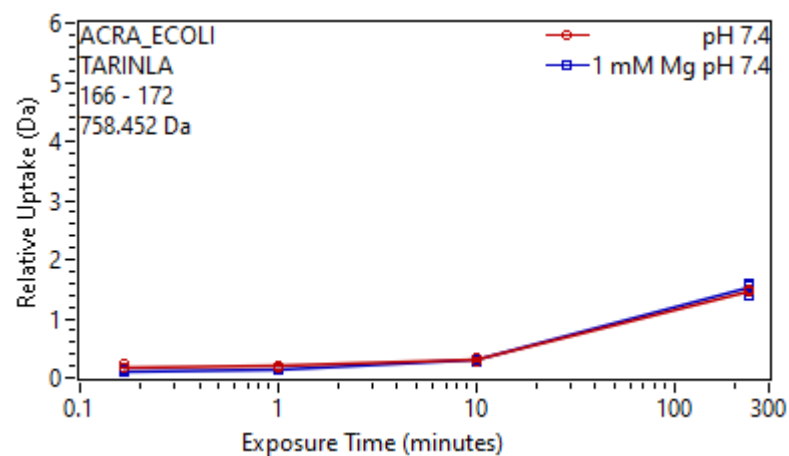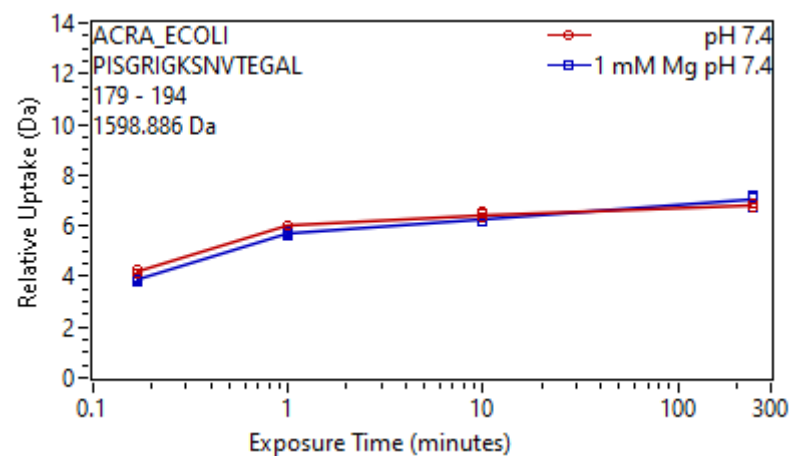

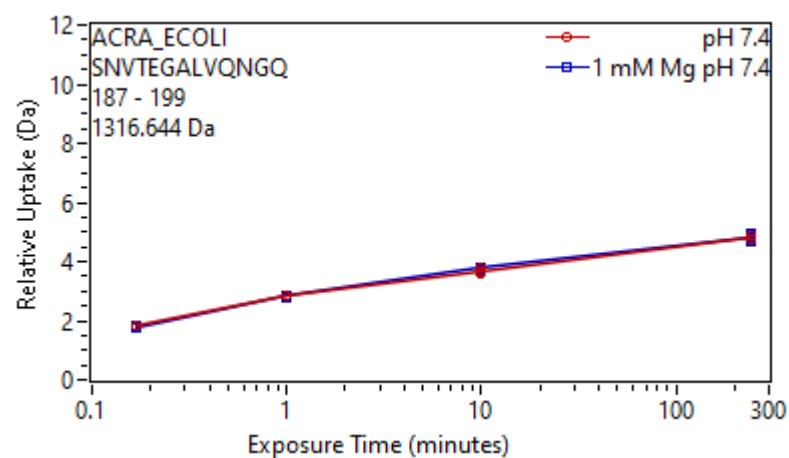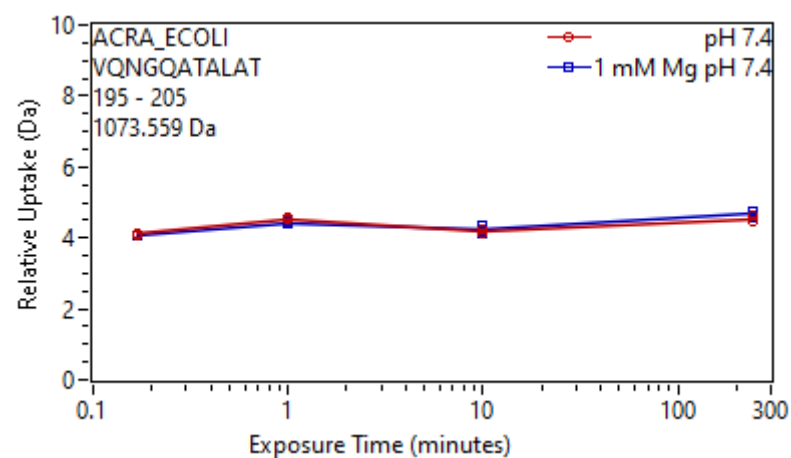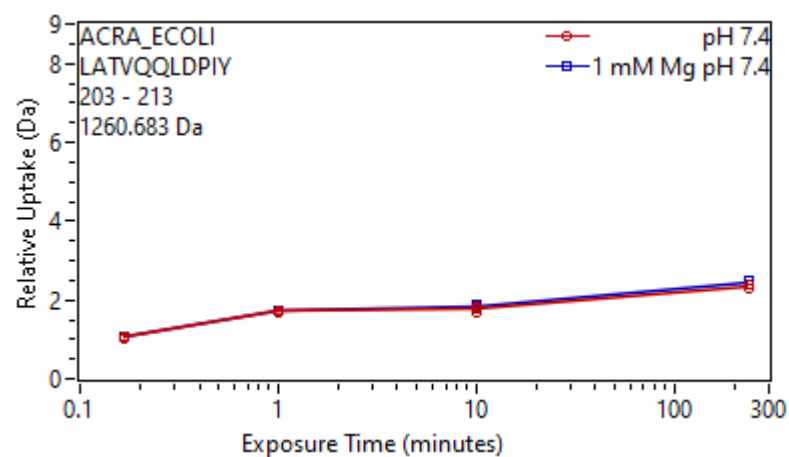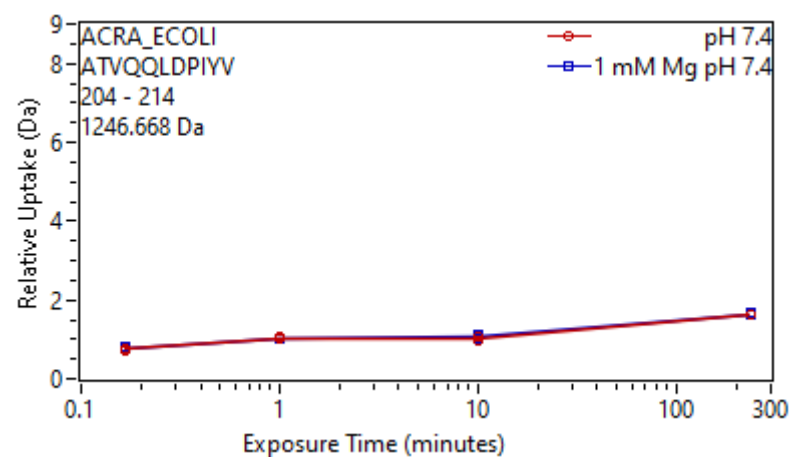

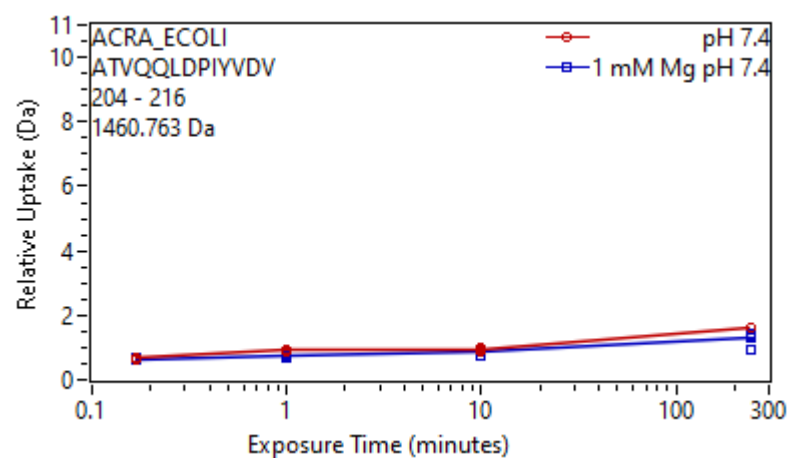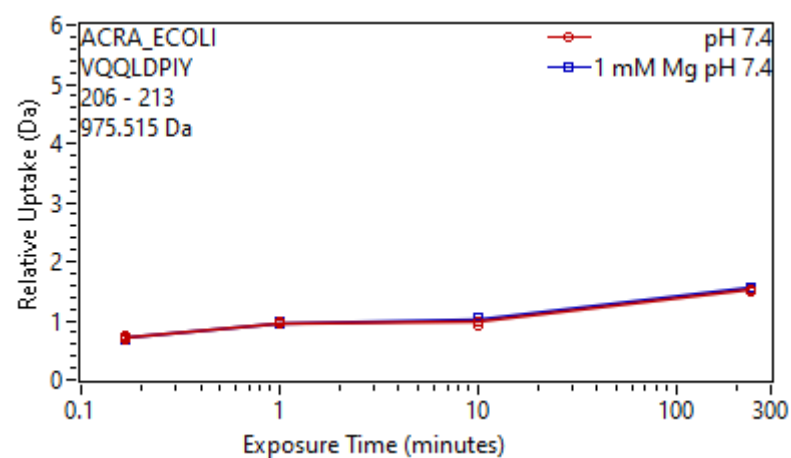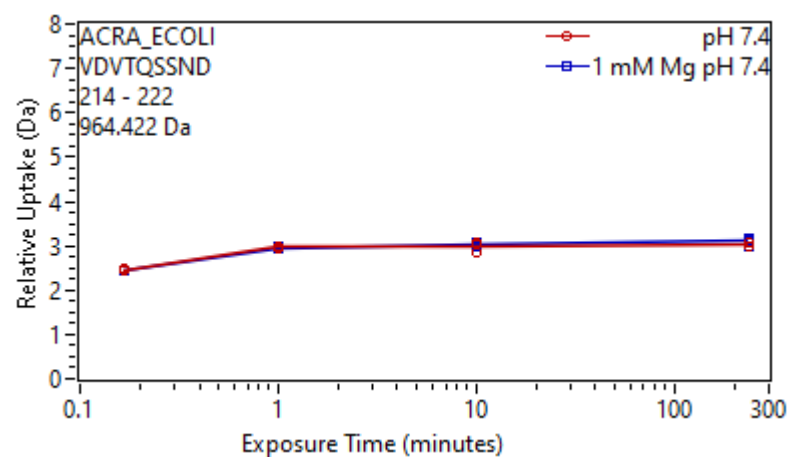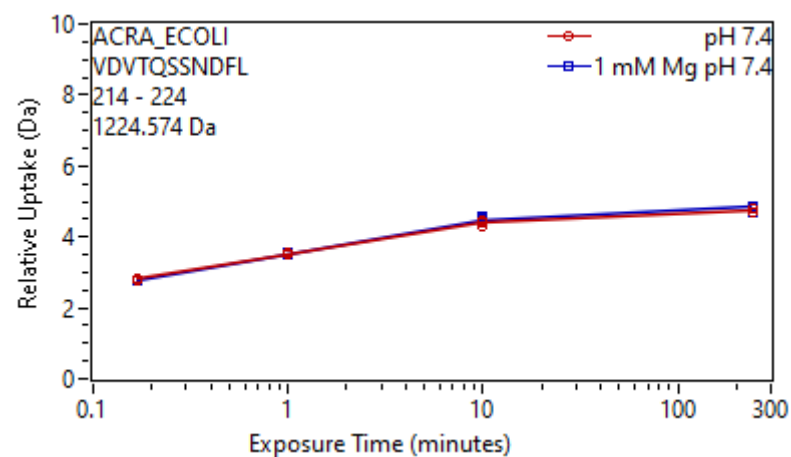

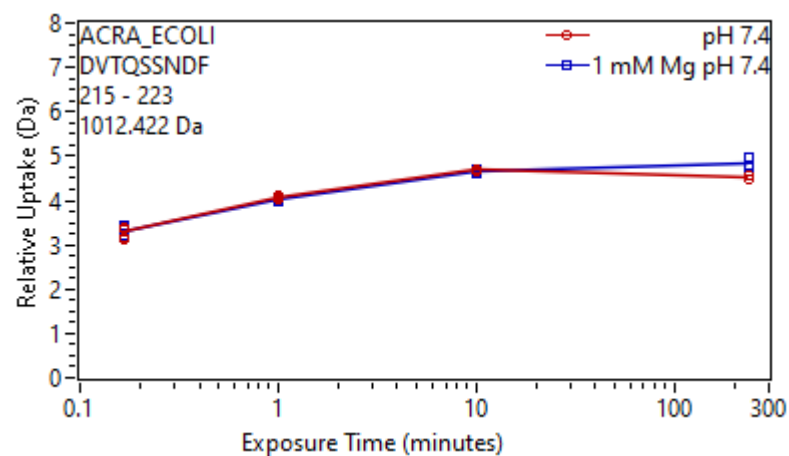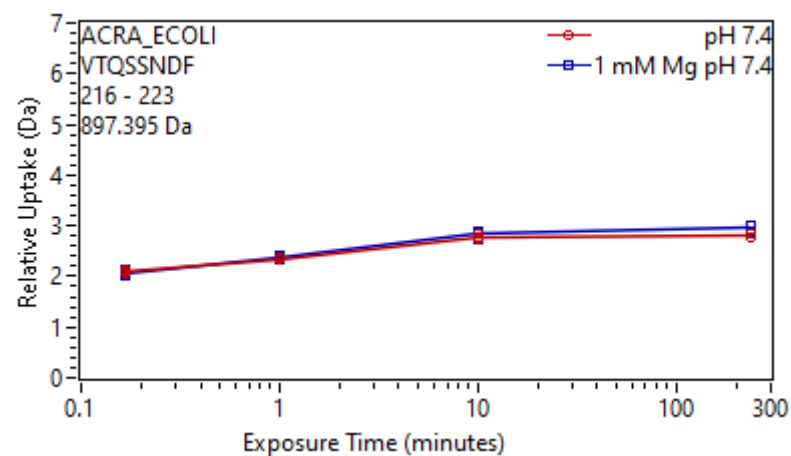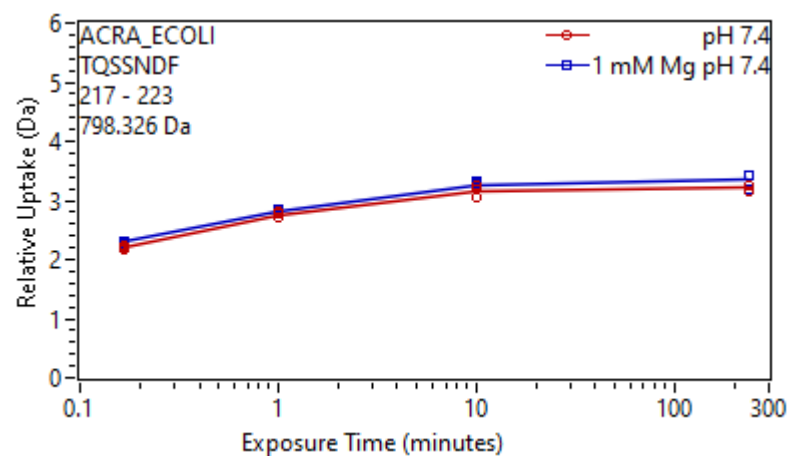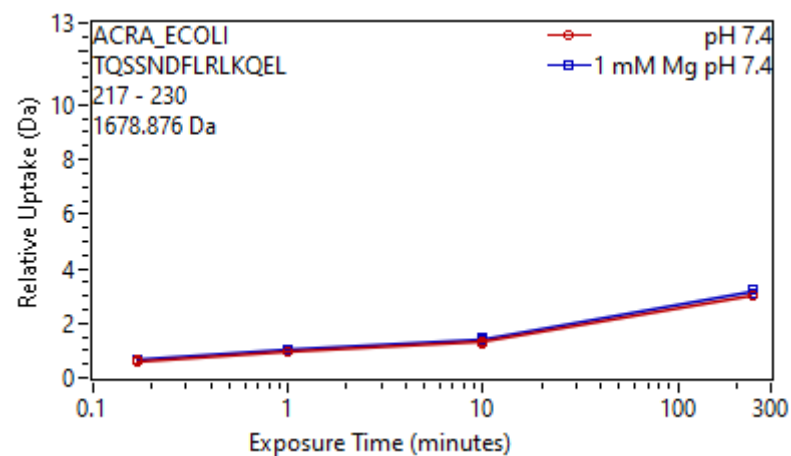

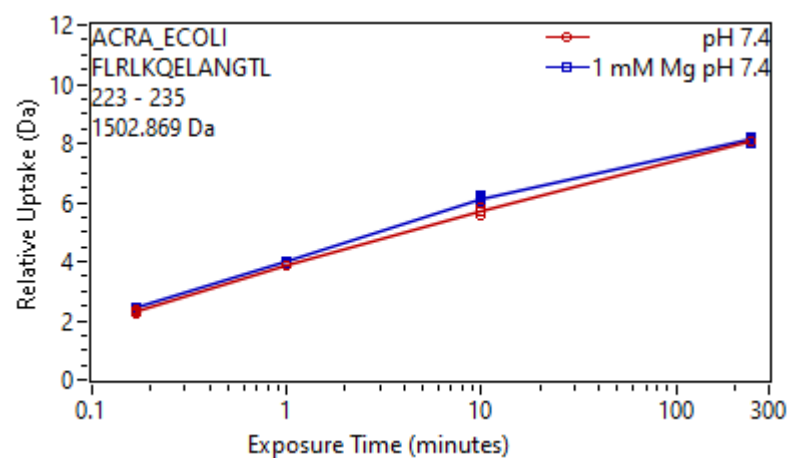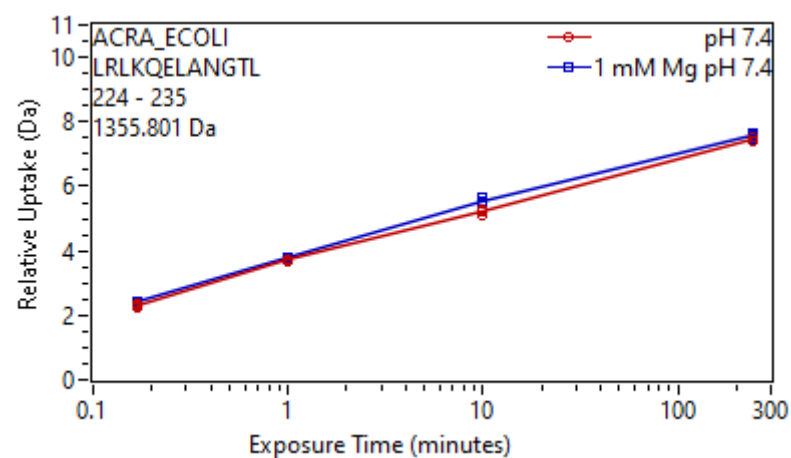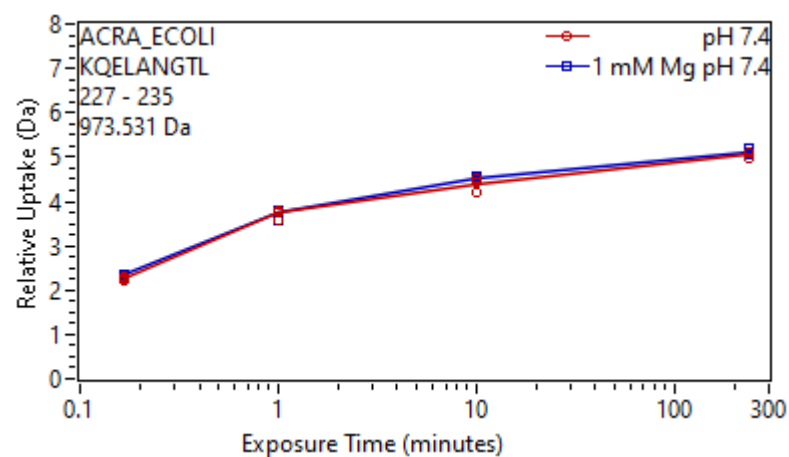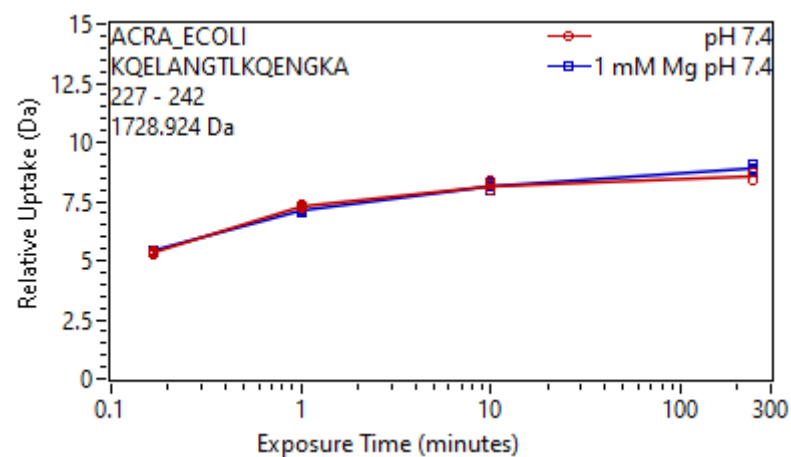

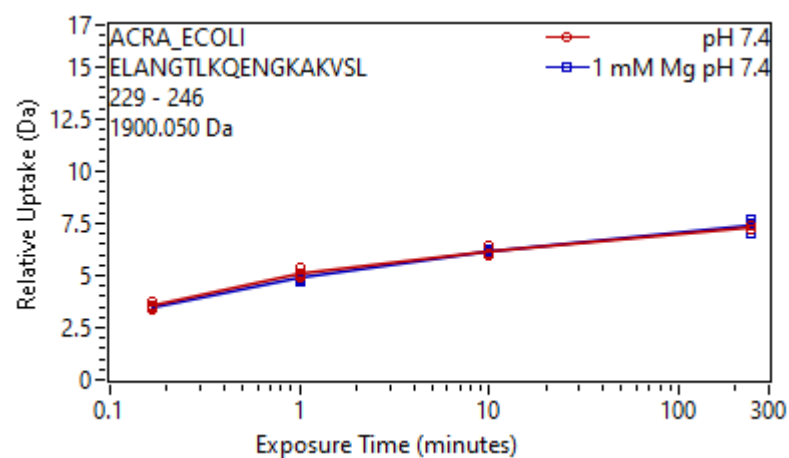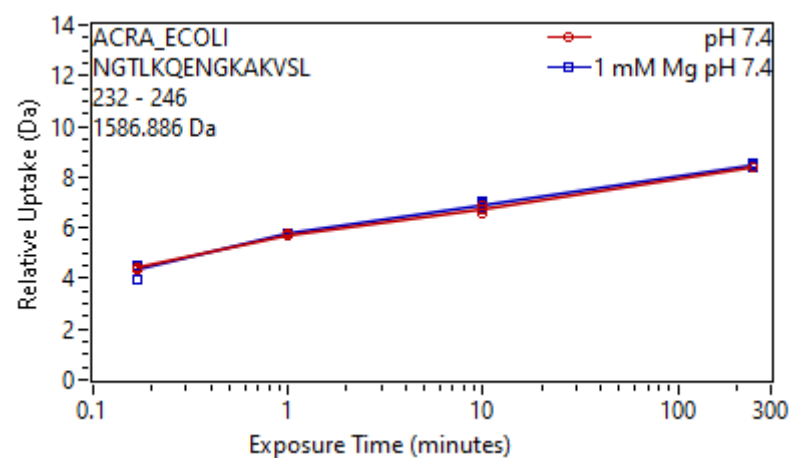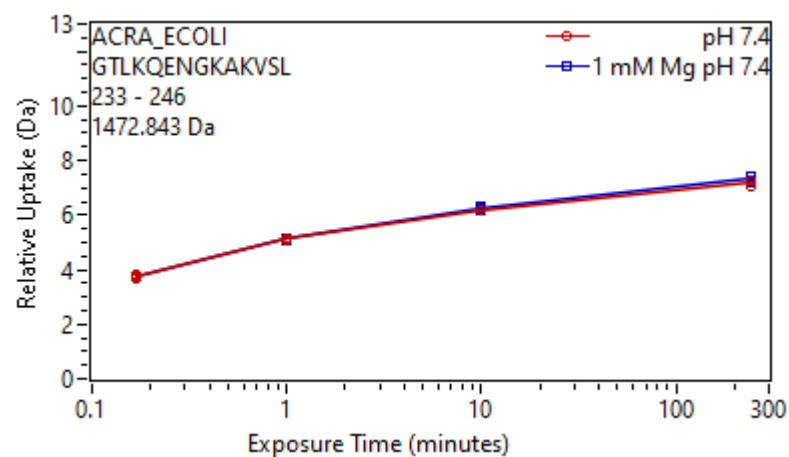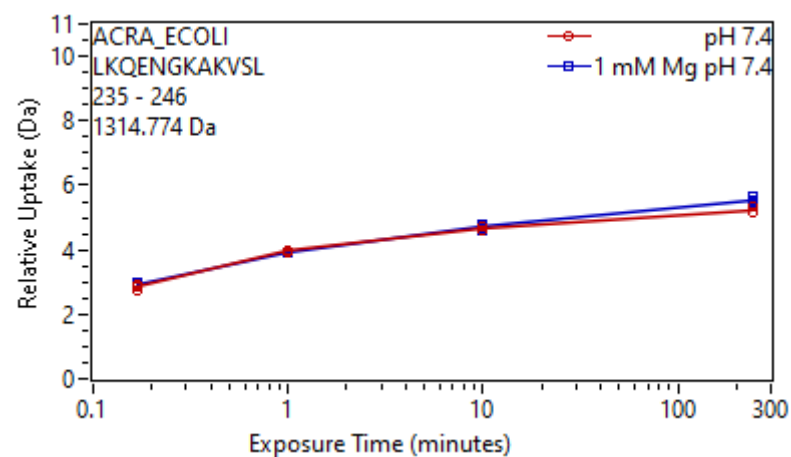

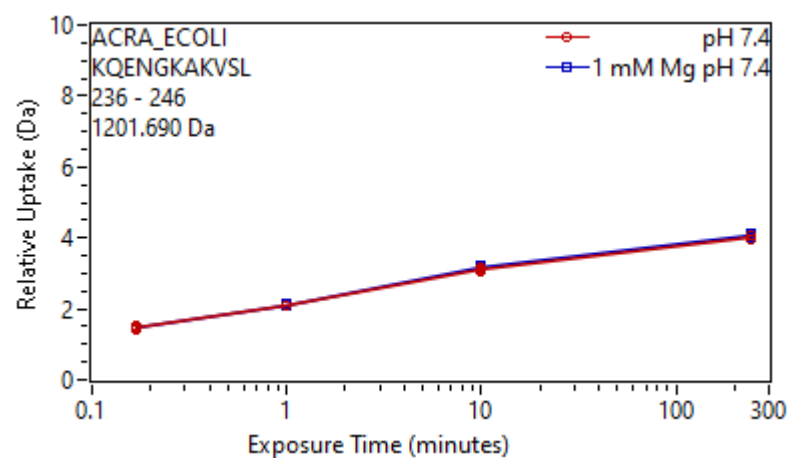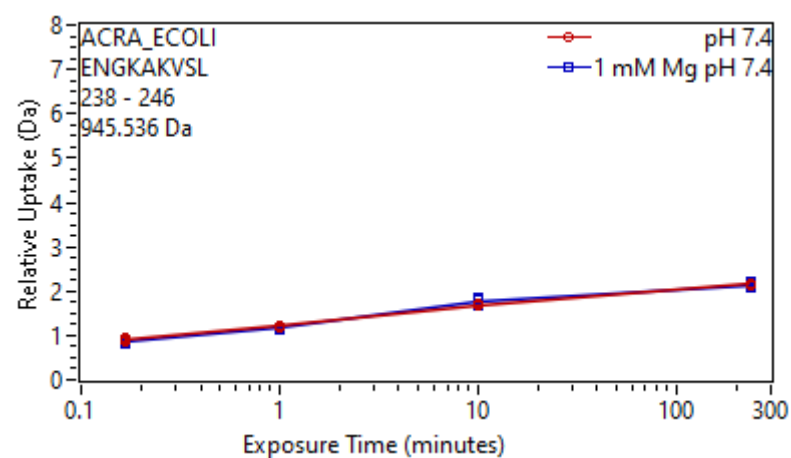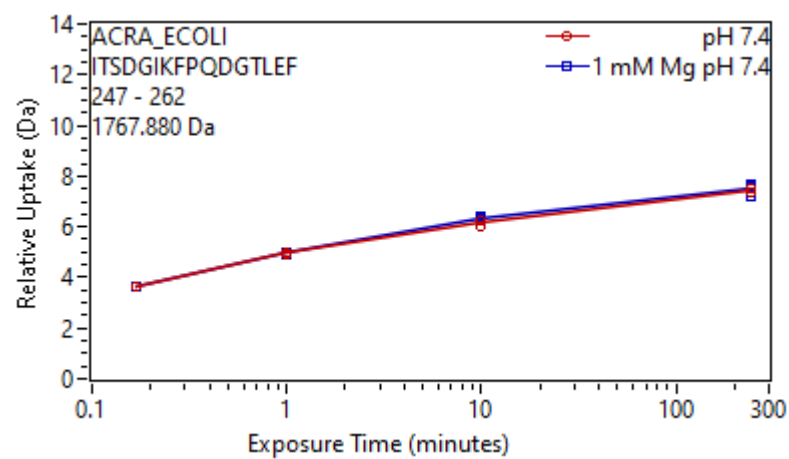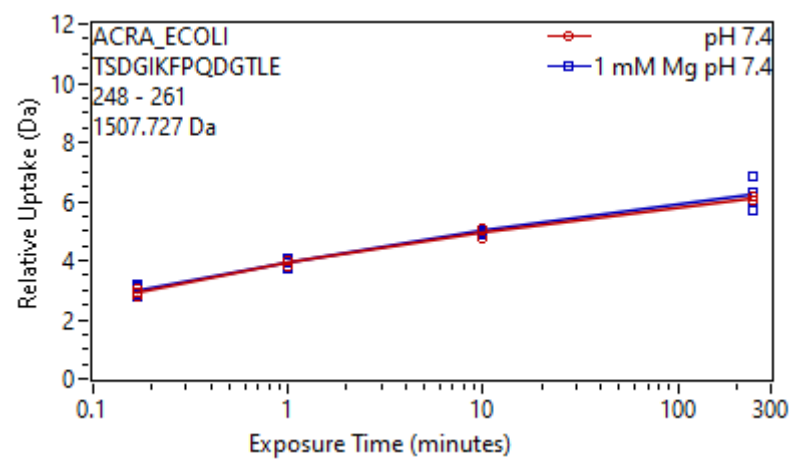

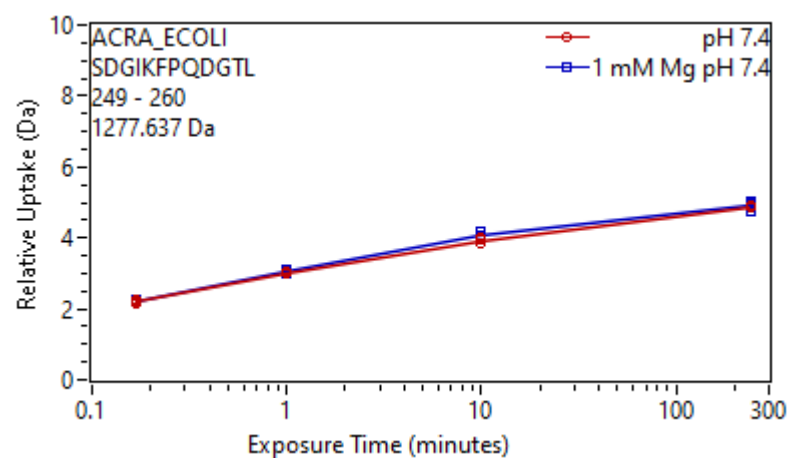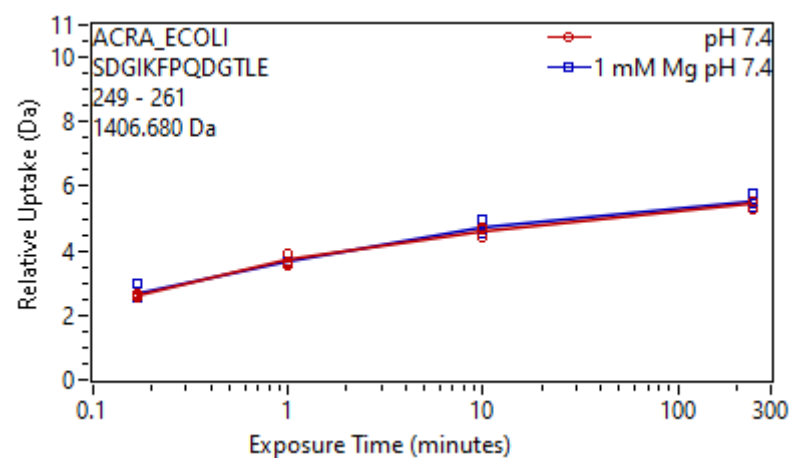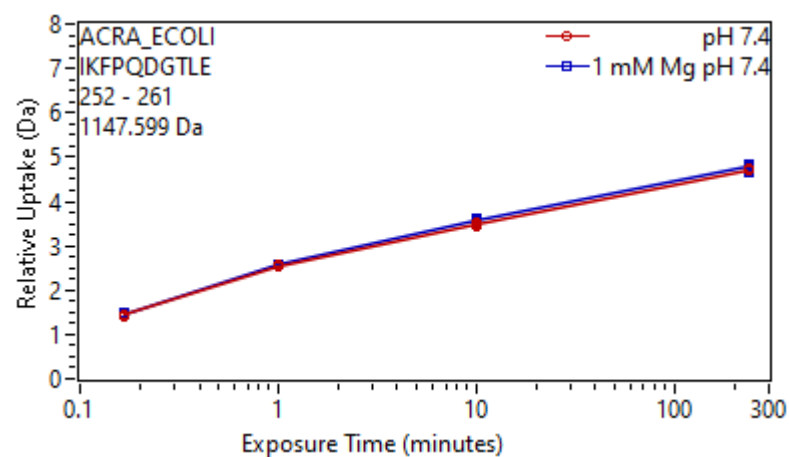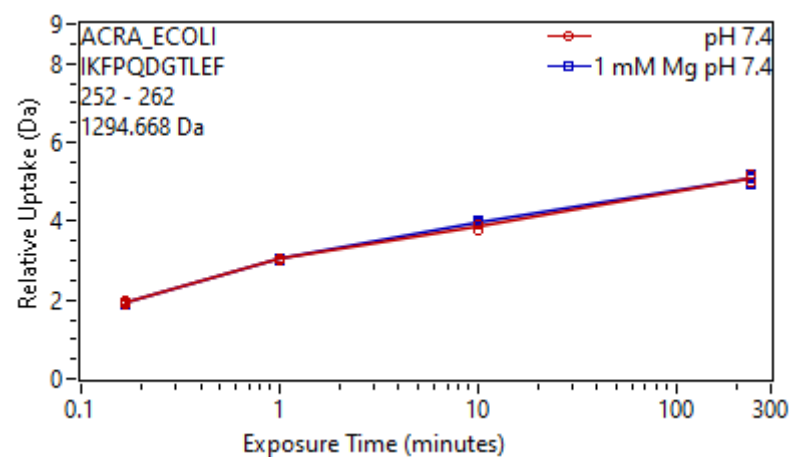

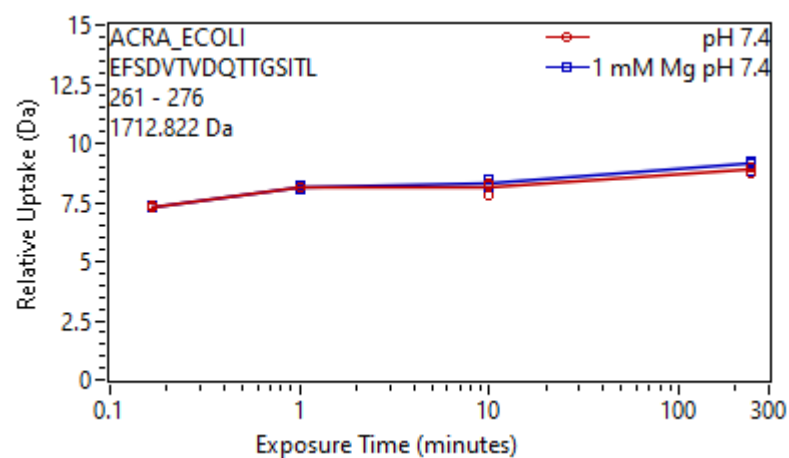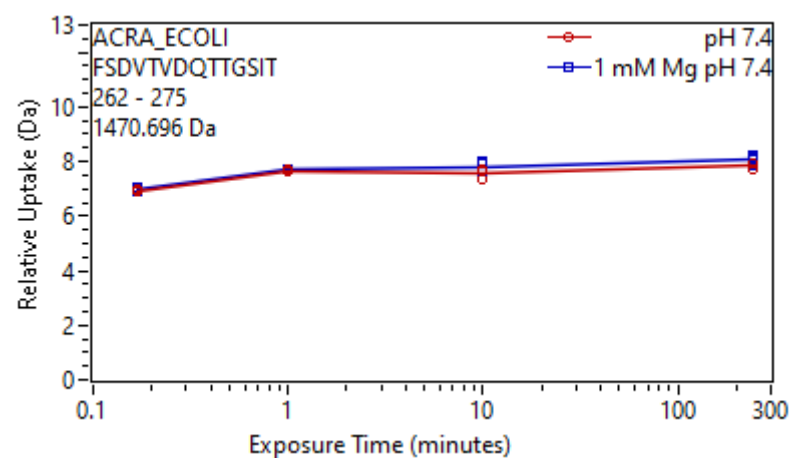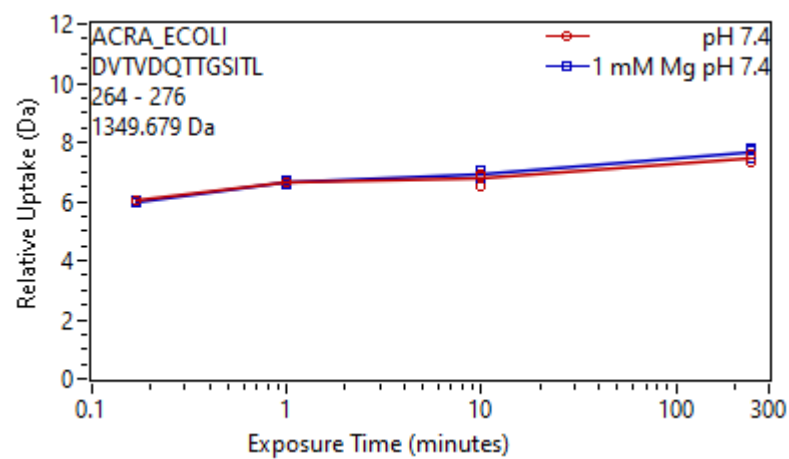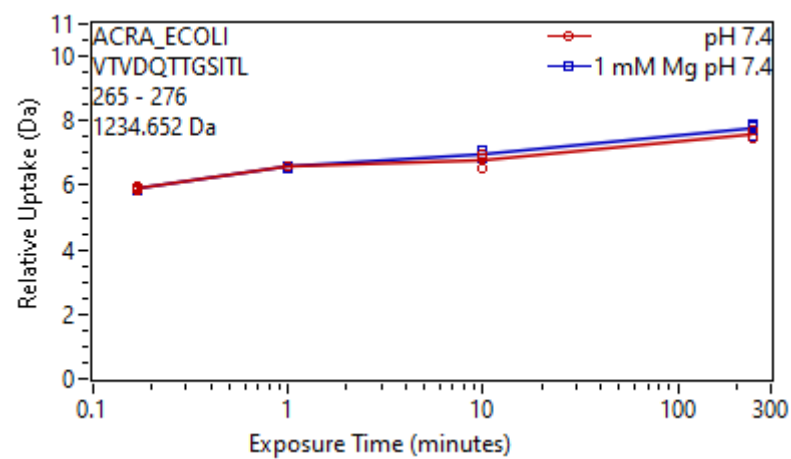

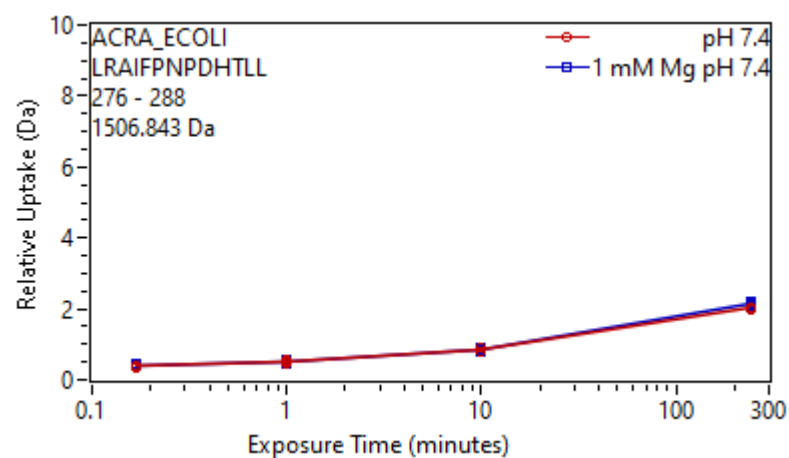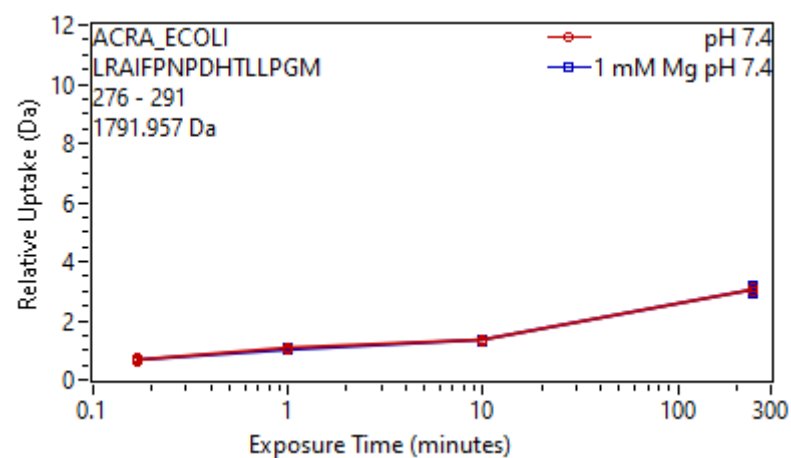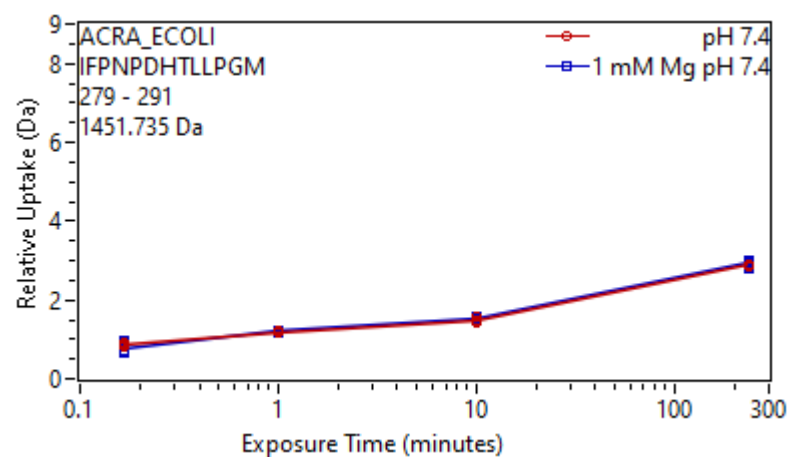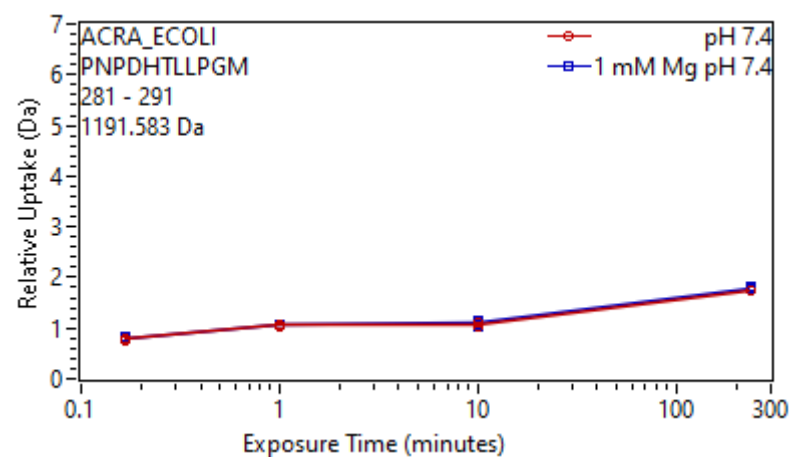

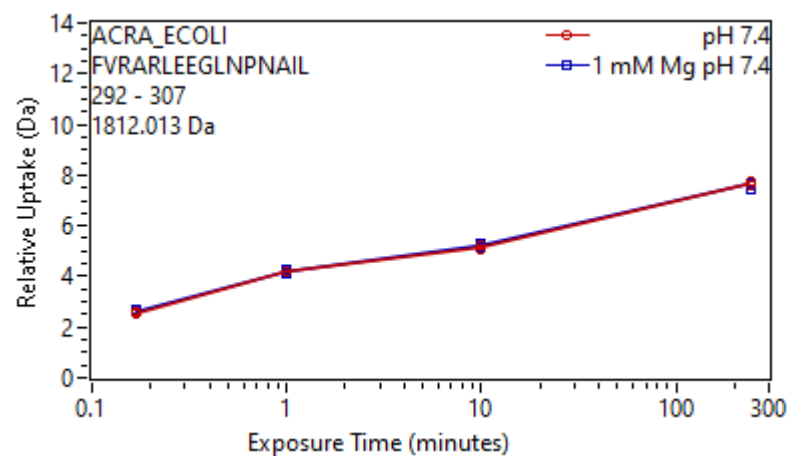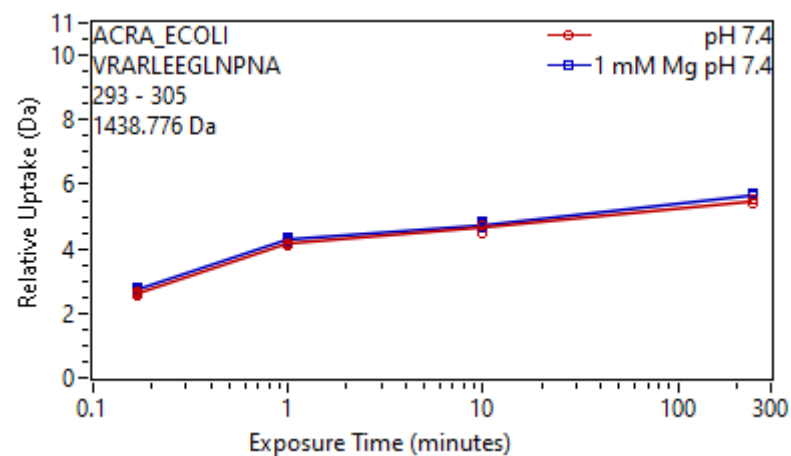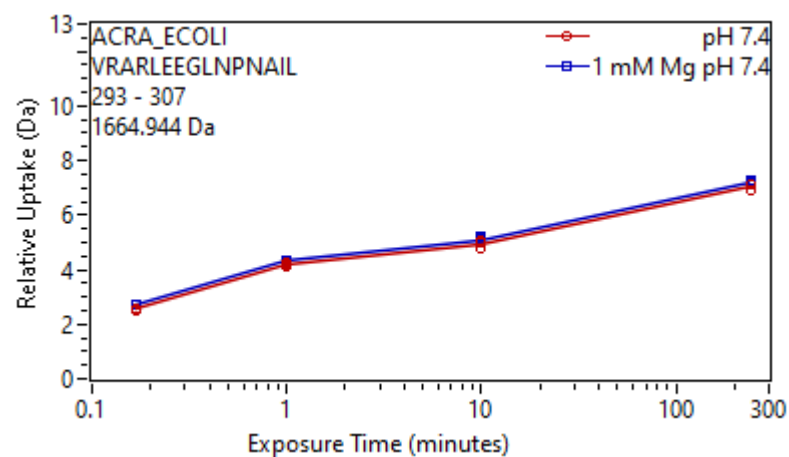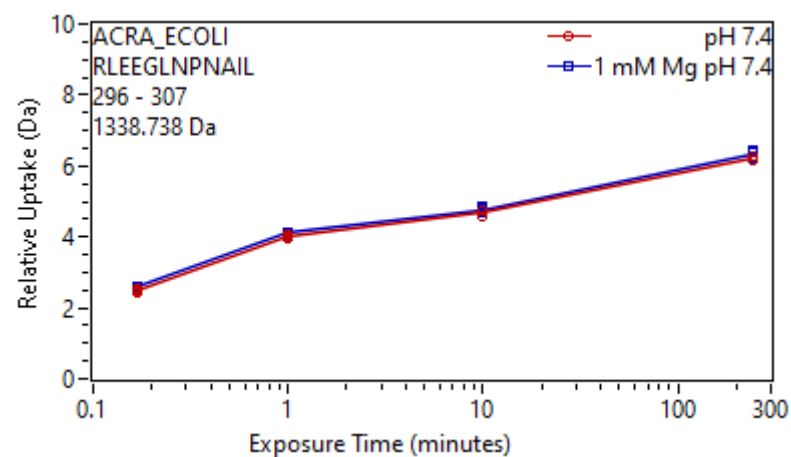

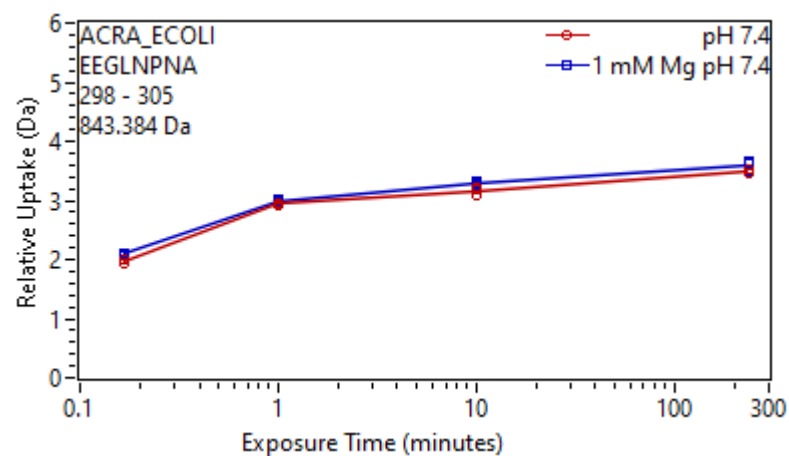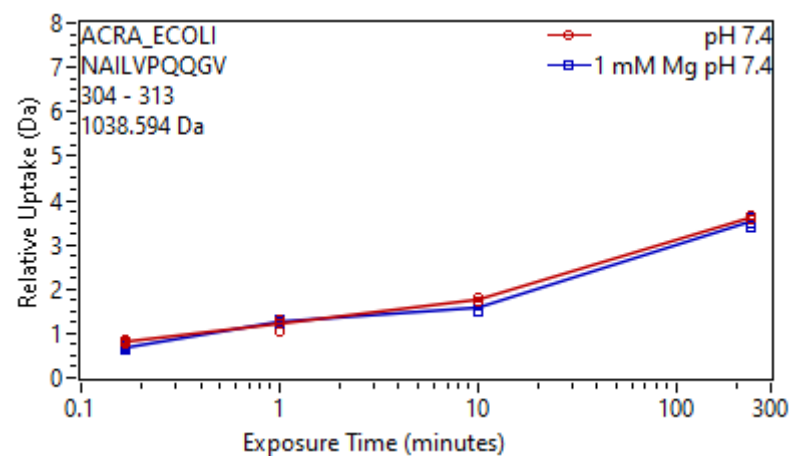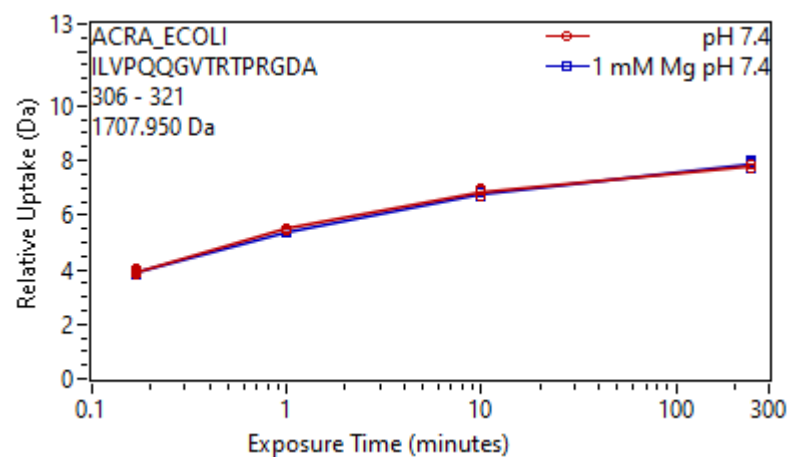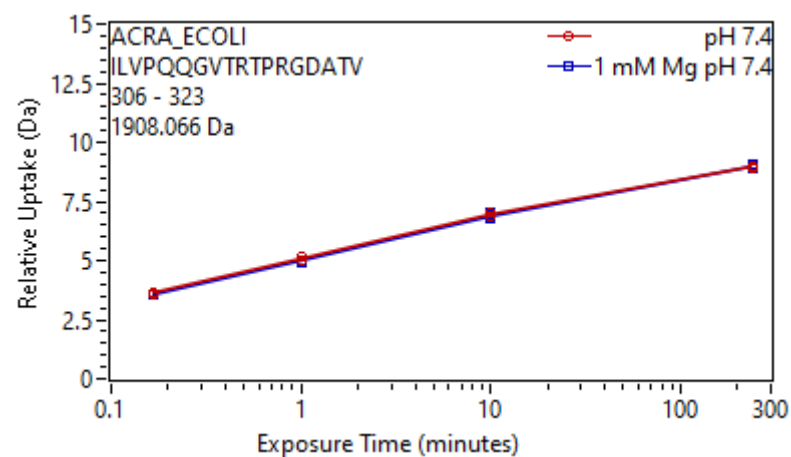

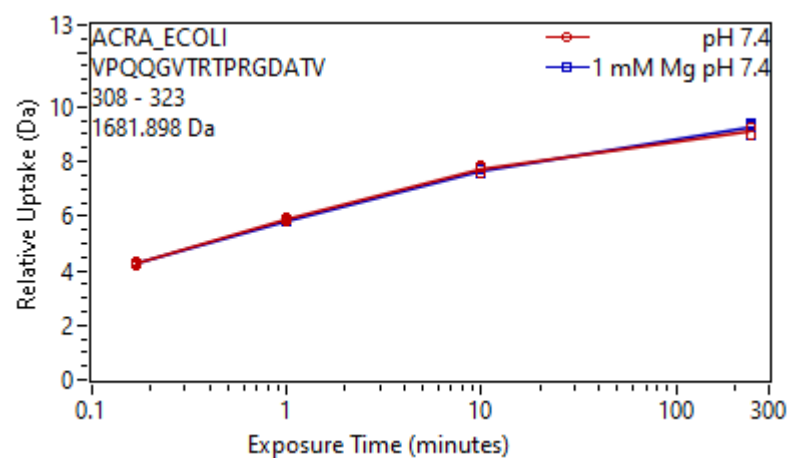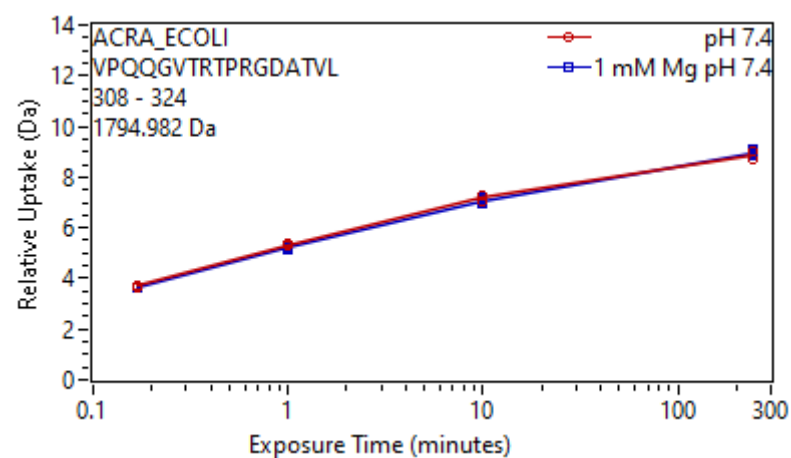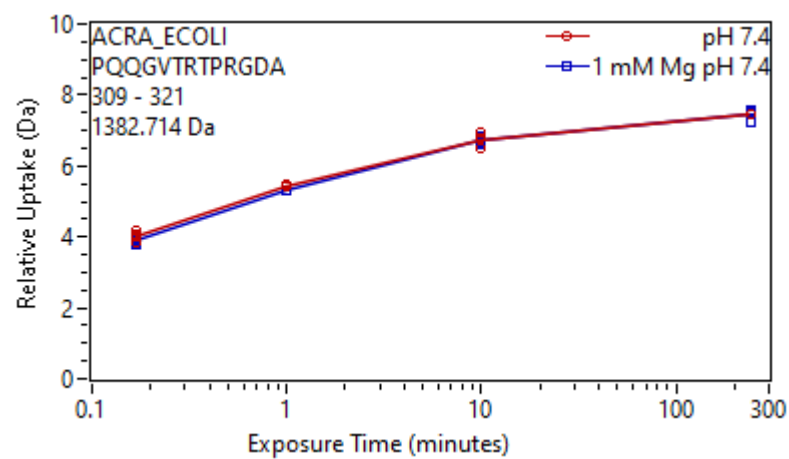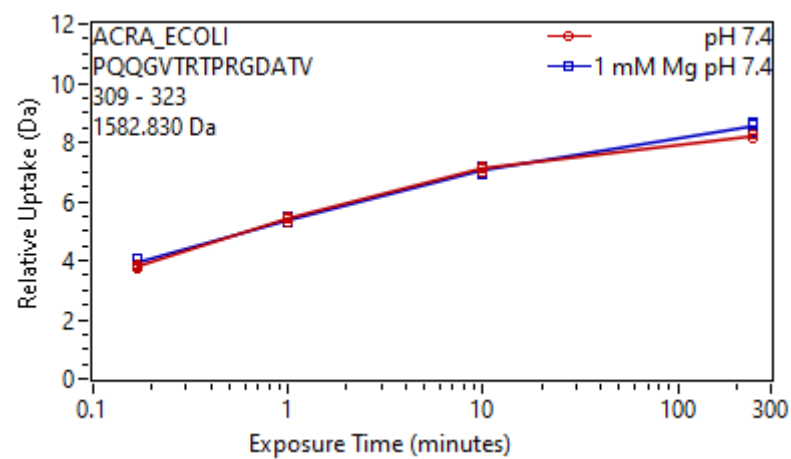

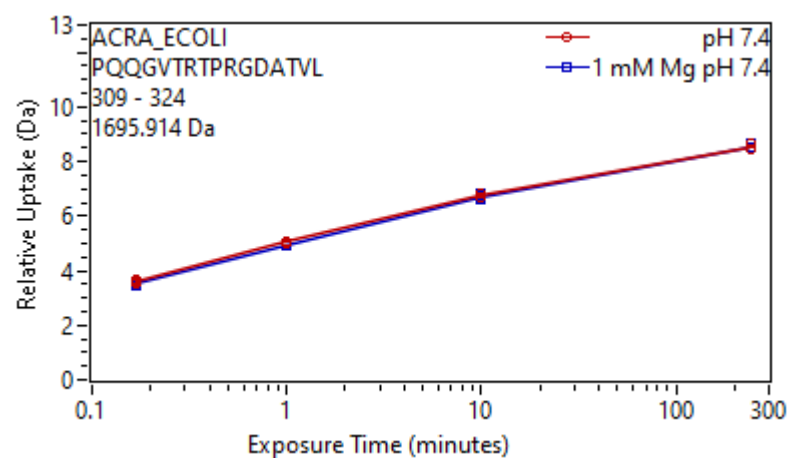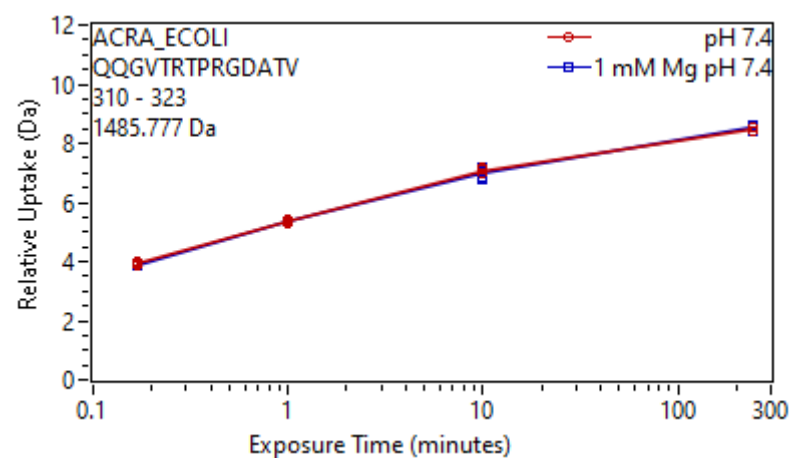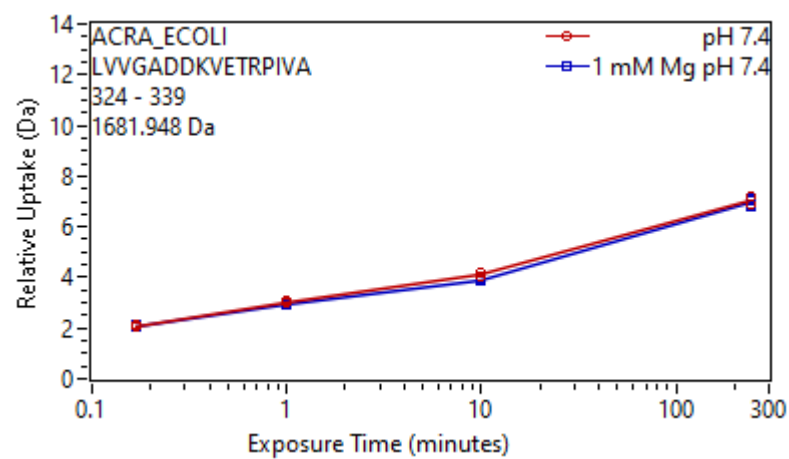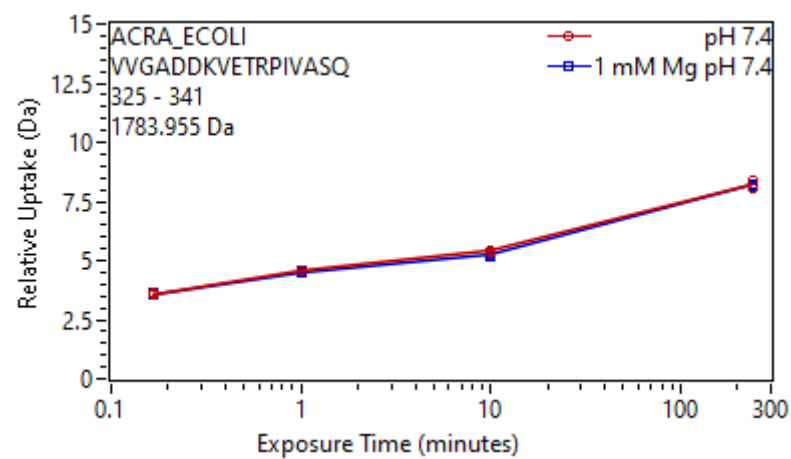

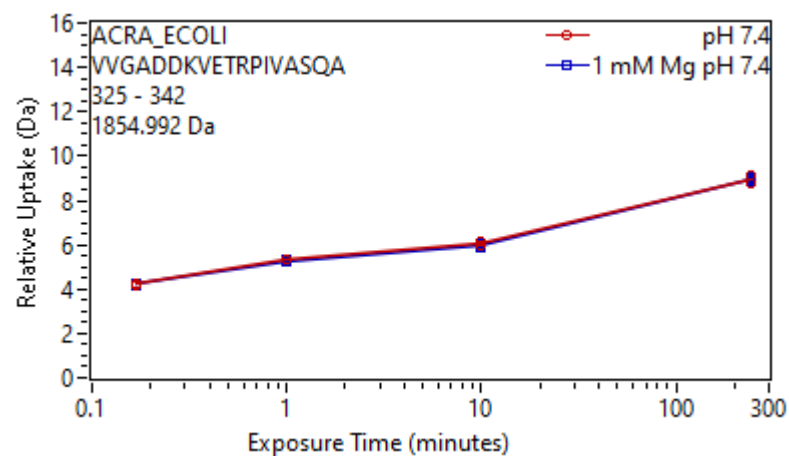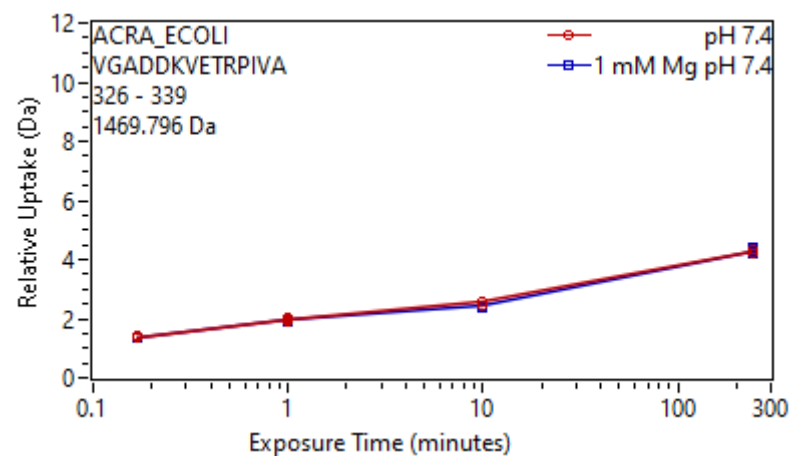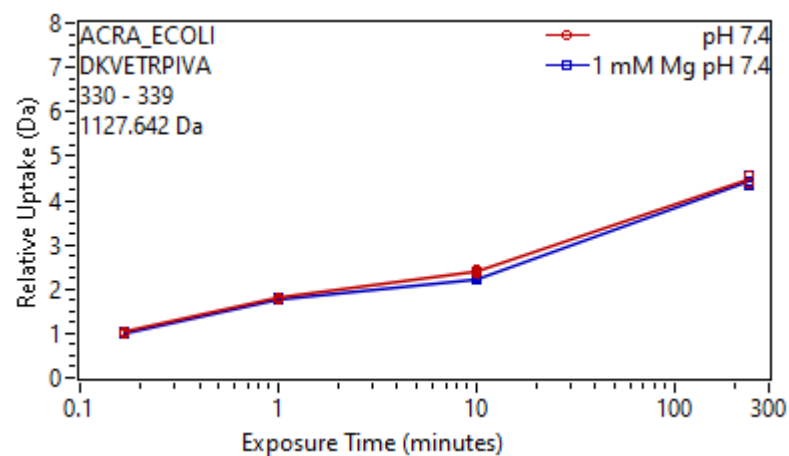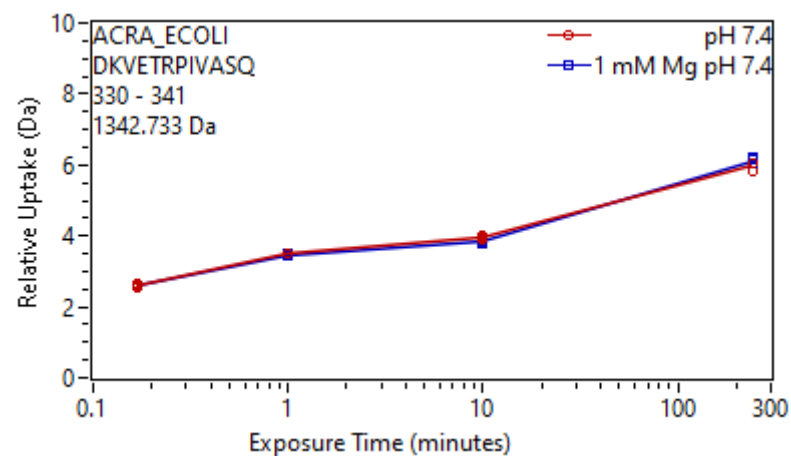

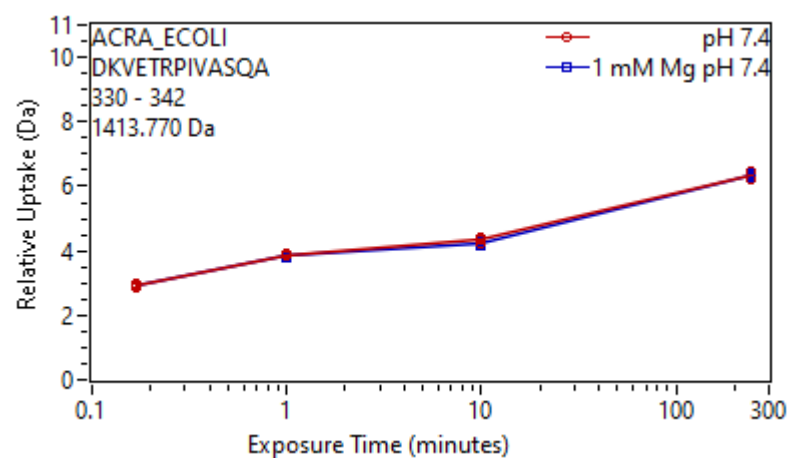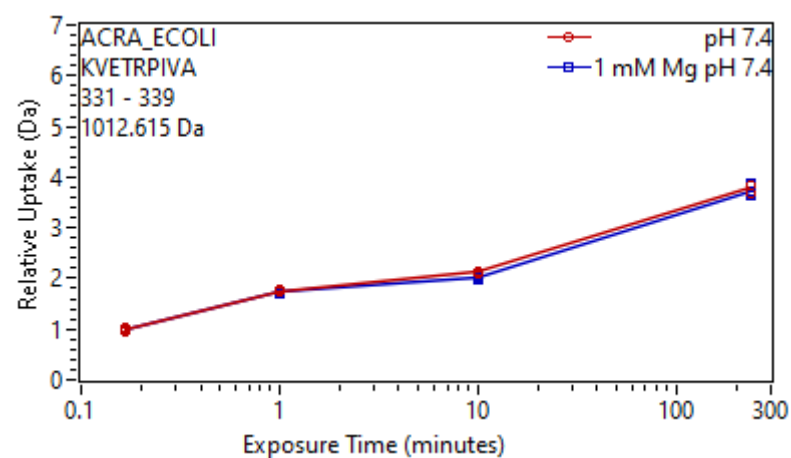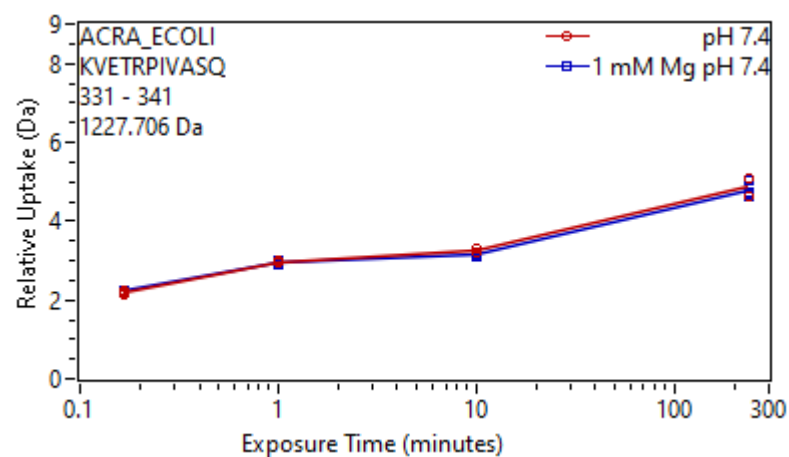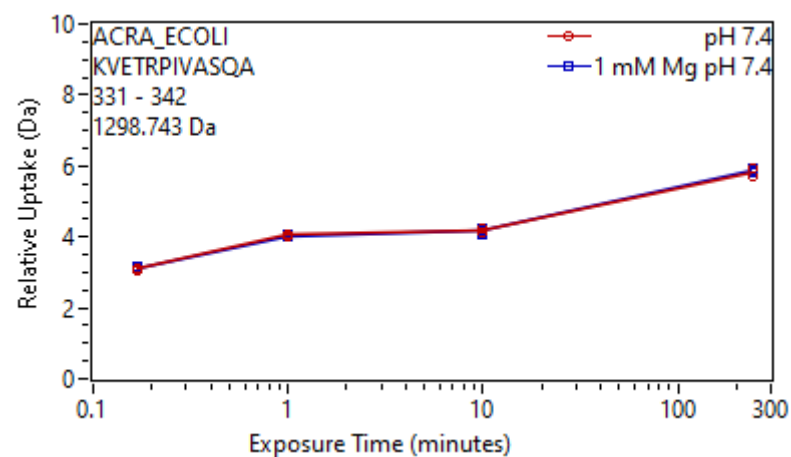

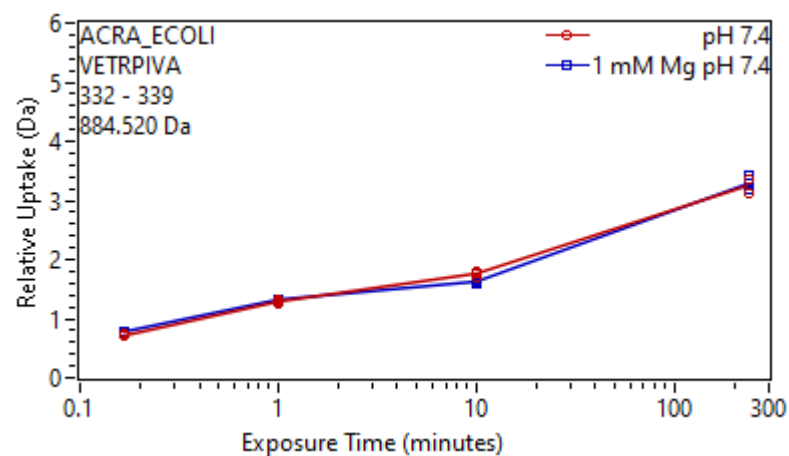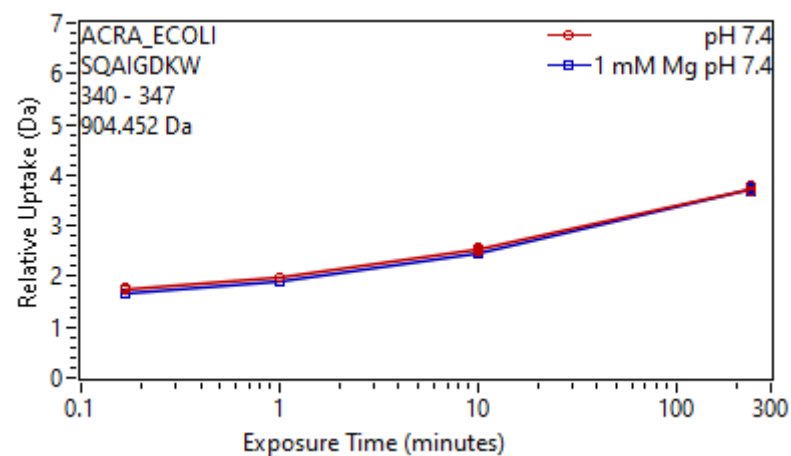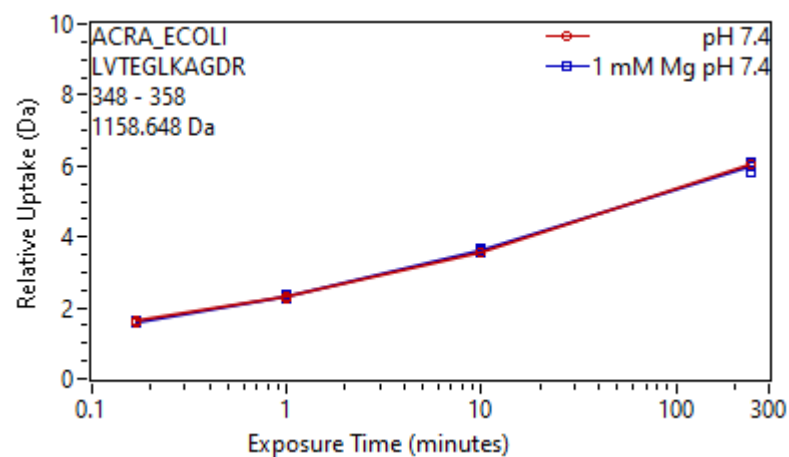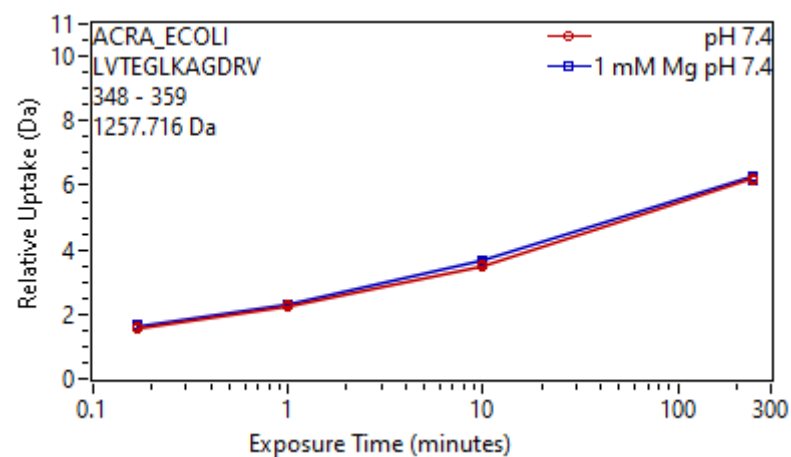

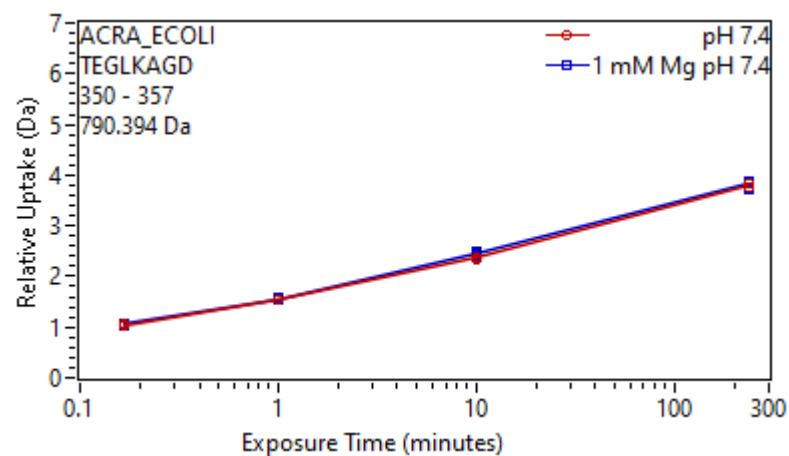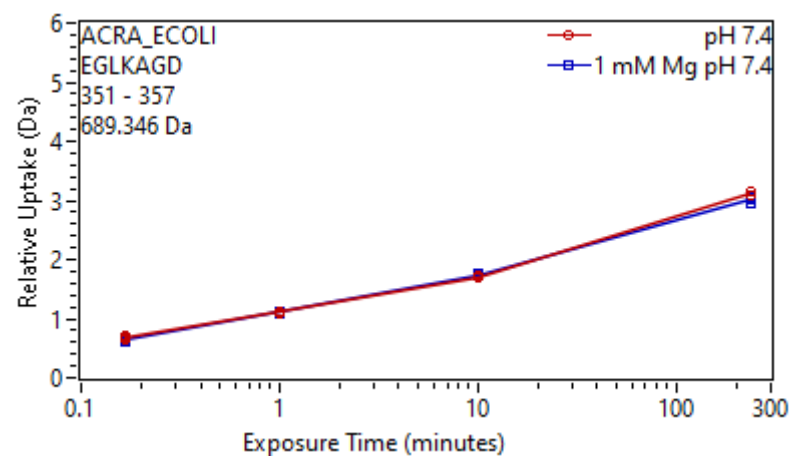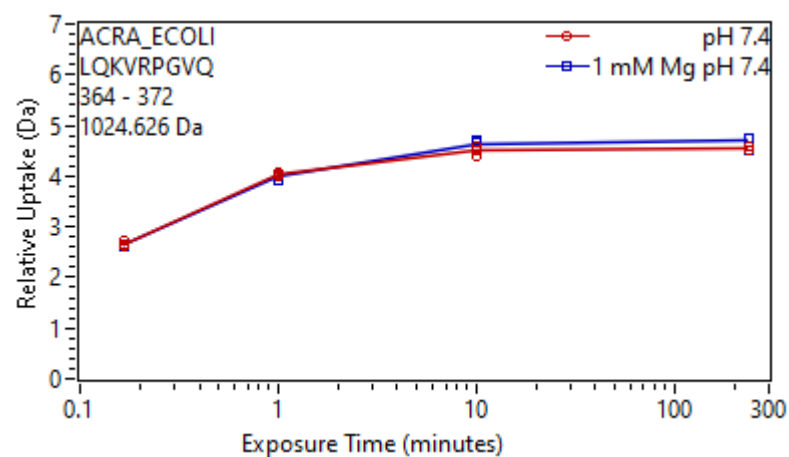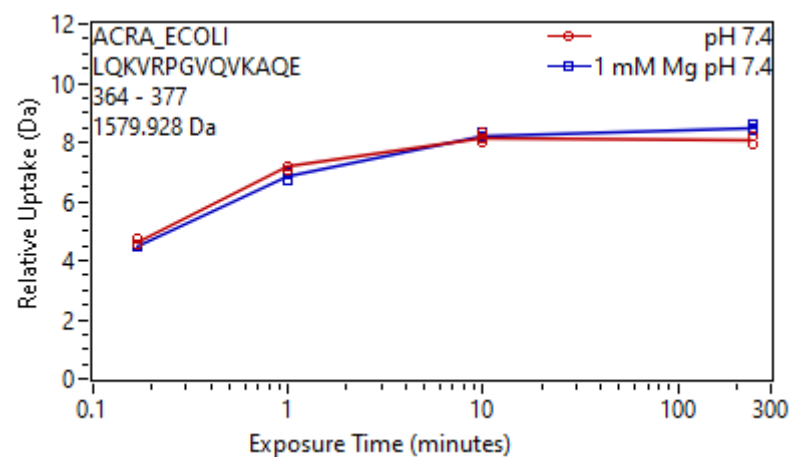

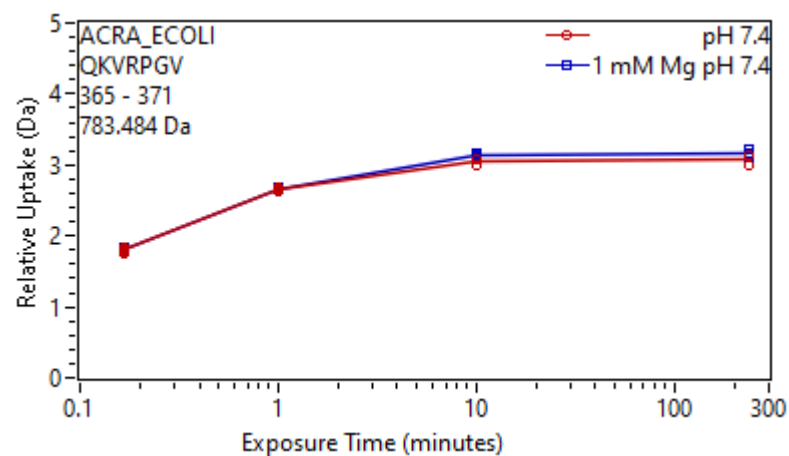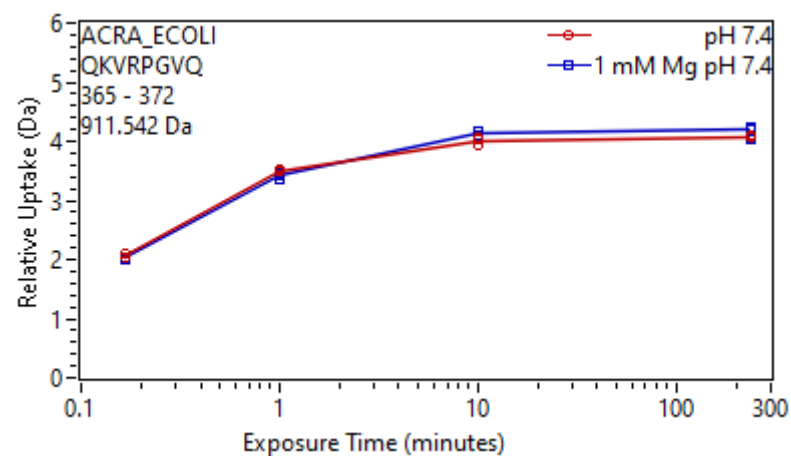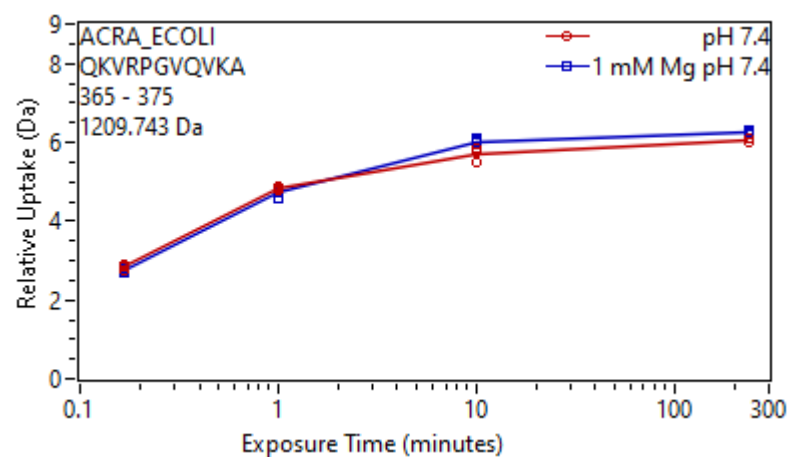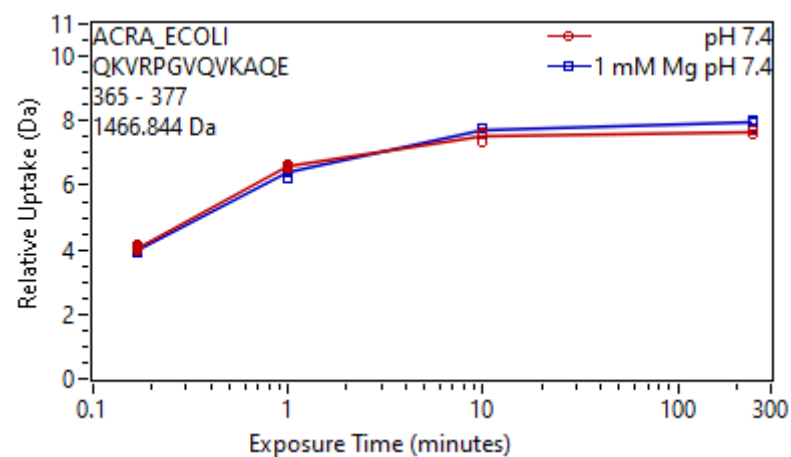

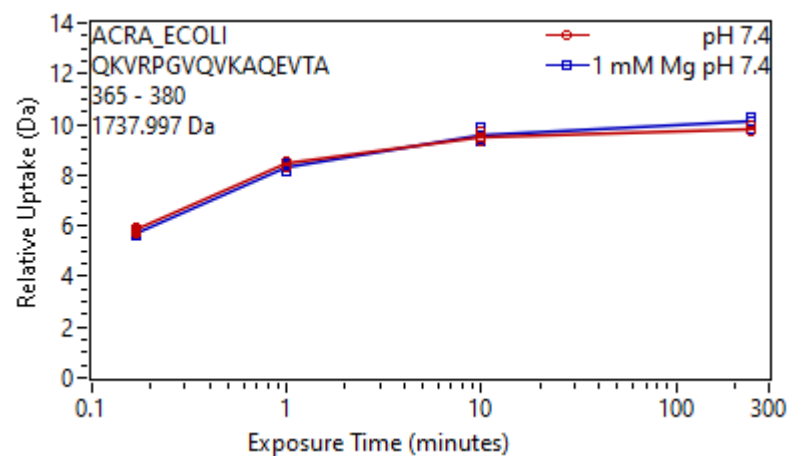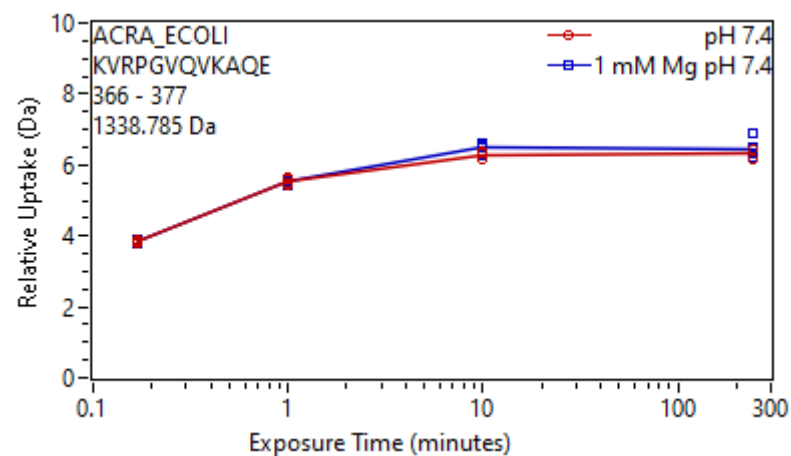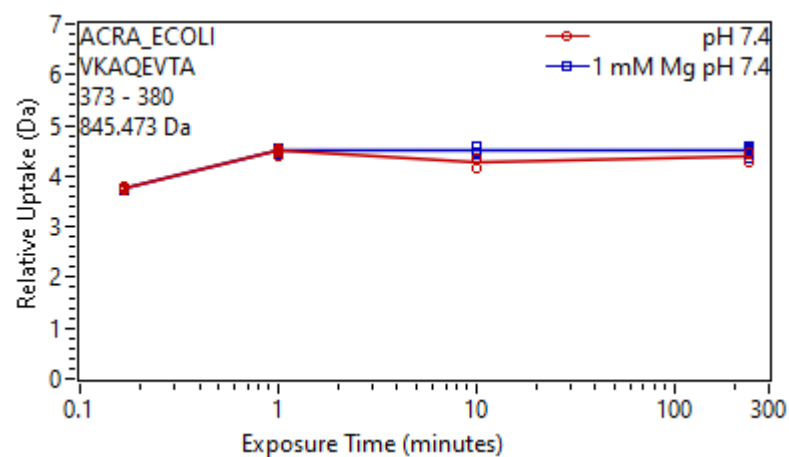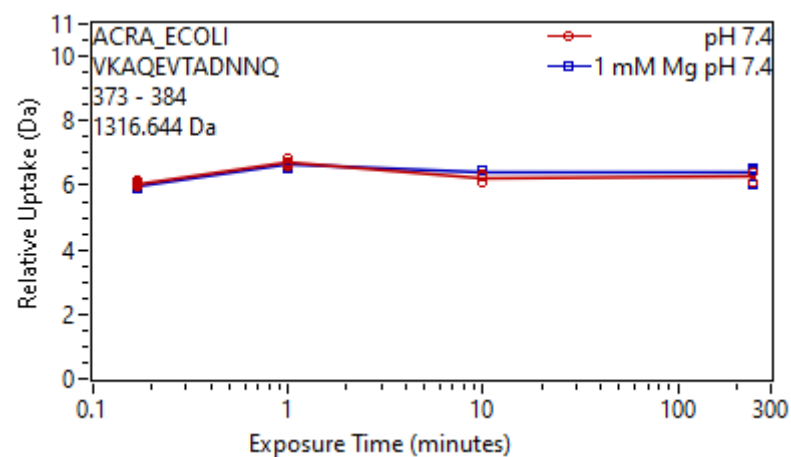

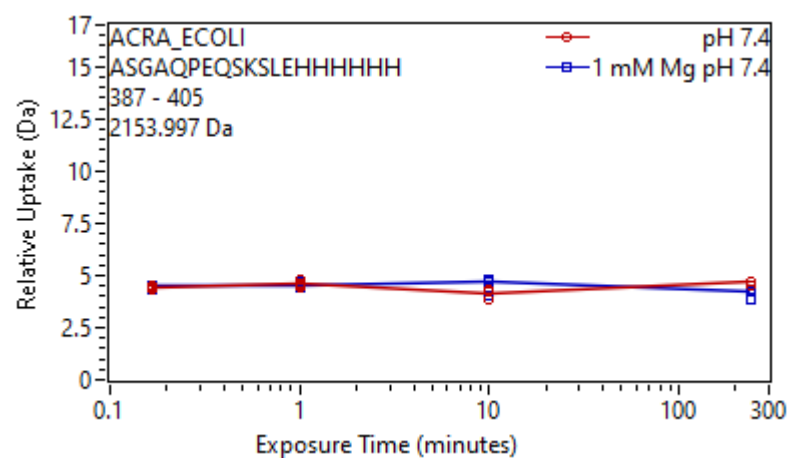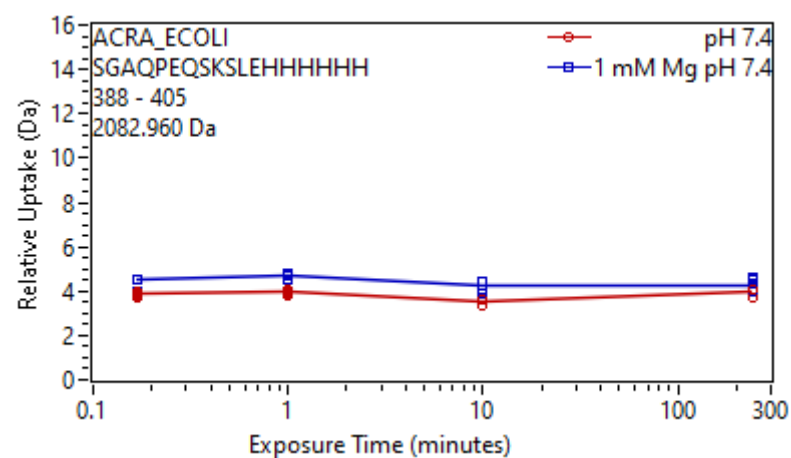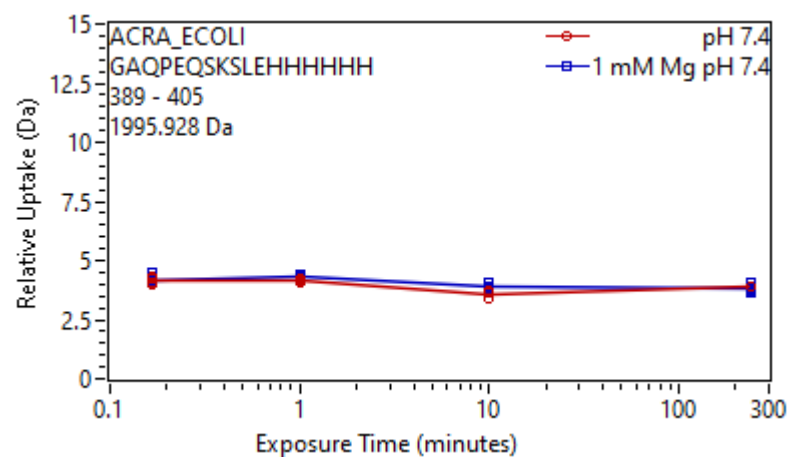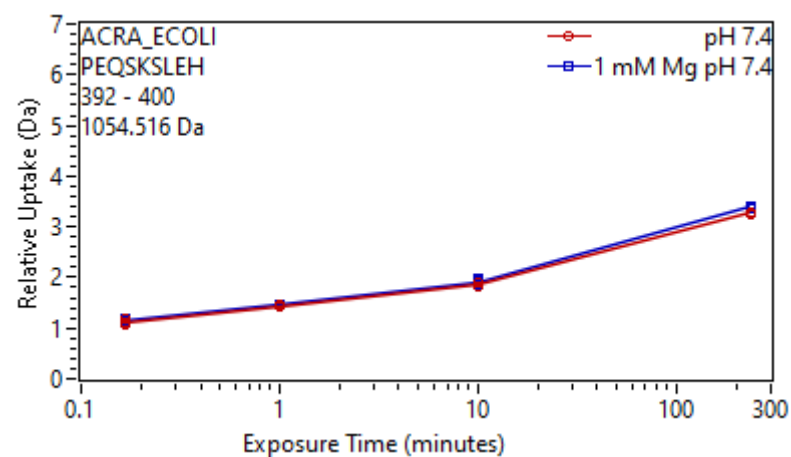

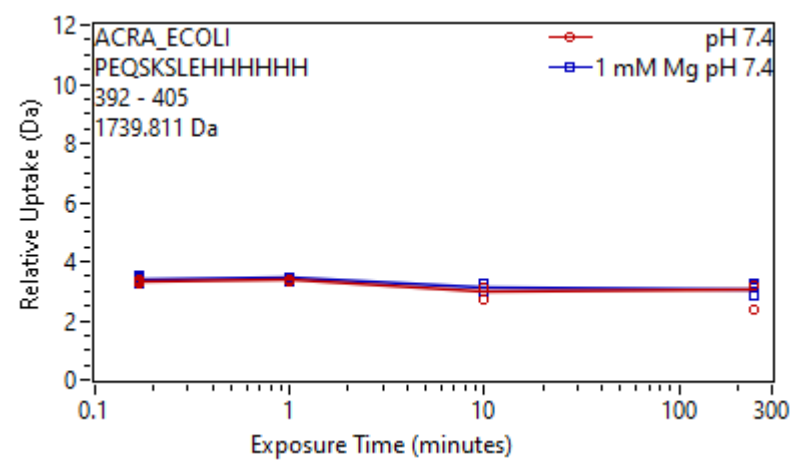

Supplement: Supplement 4 [file media-4.zip › Supplementary Data 3/Uptake plots 3.pdf]

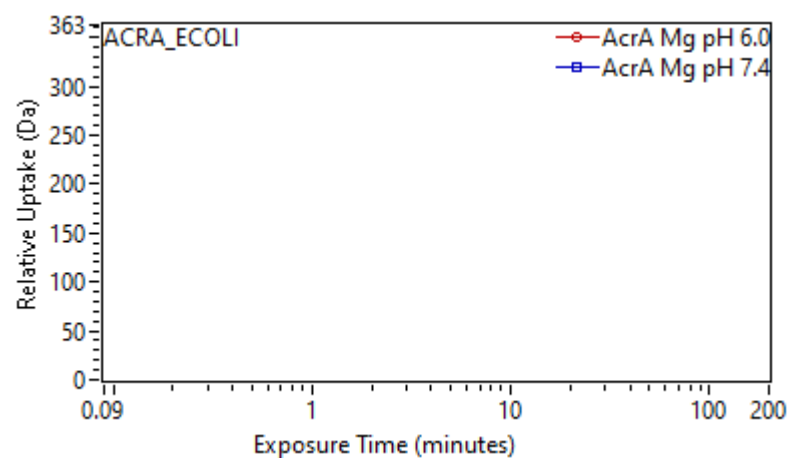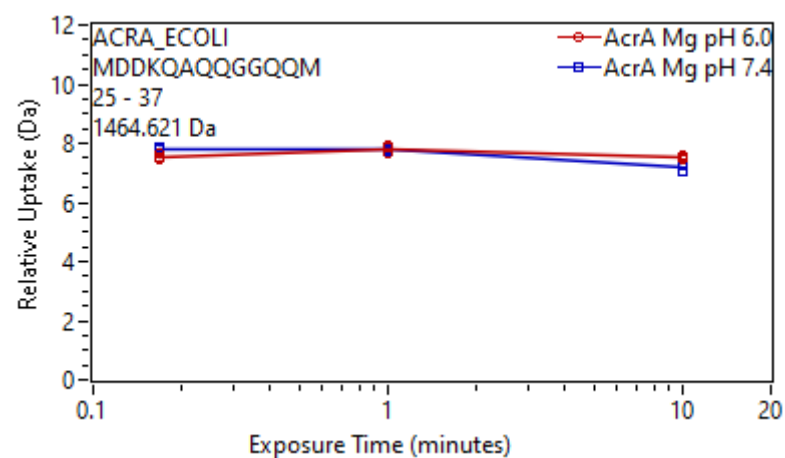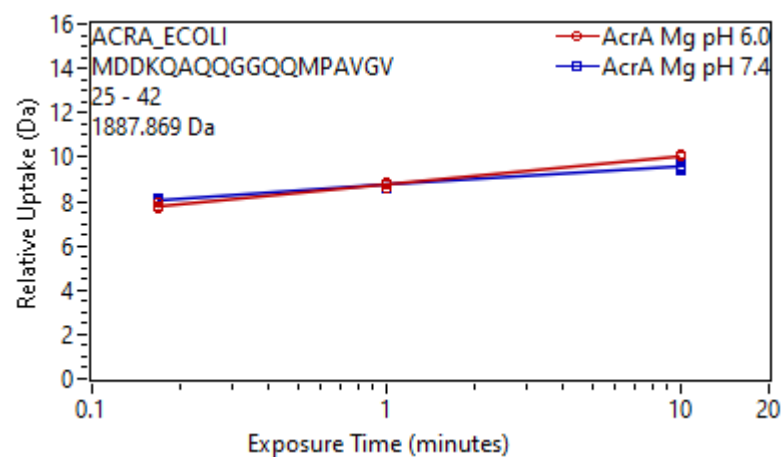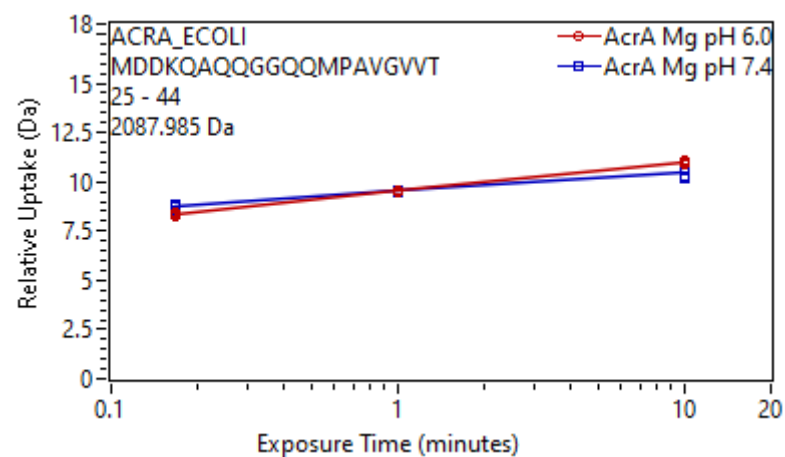

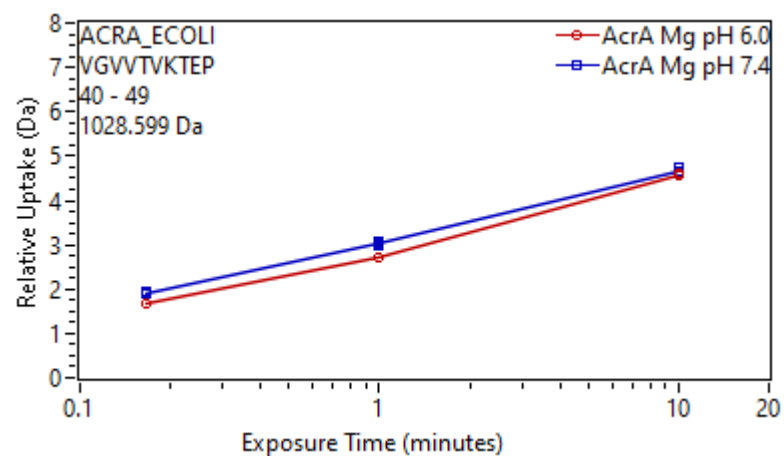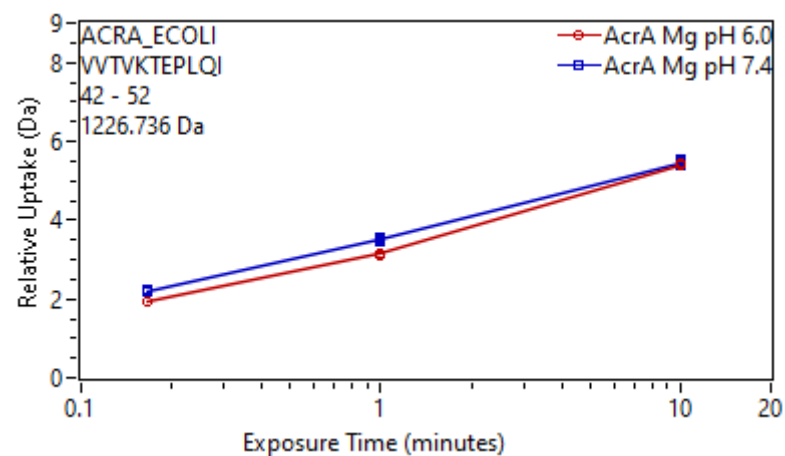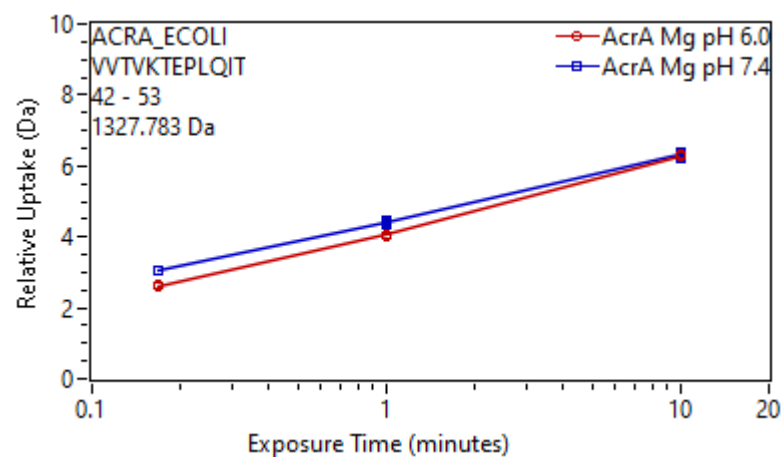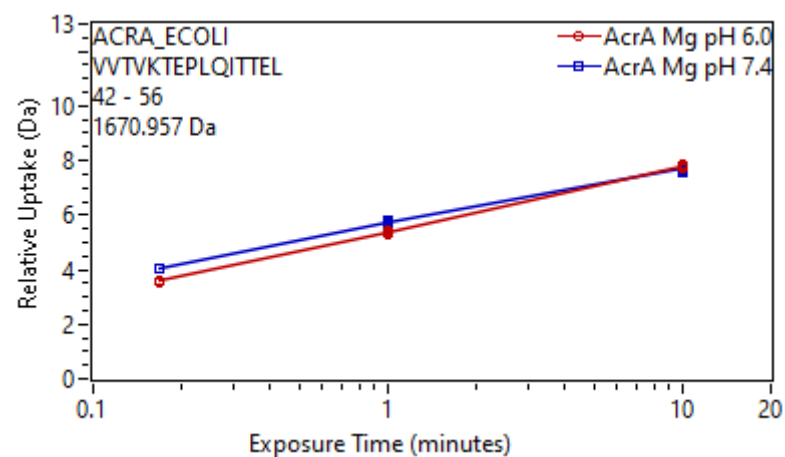

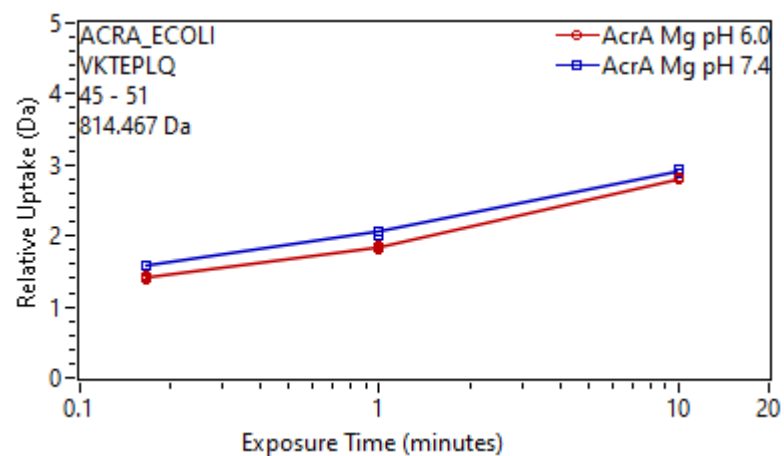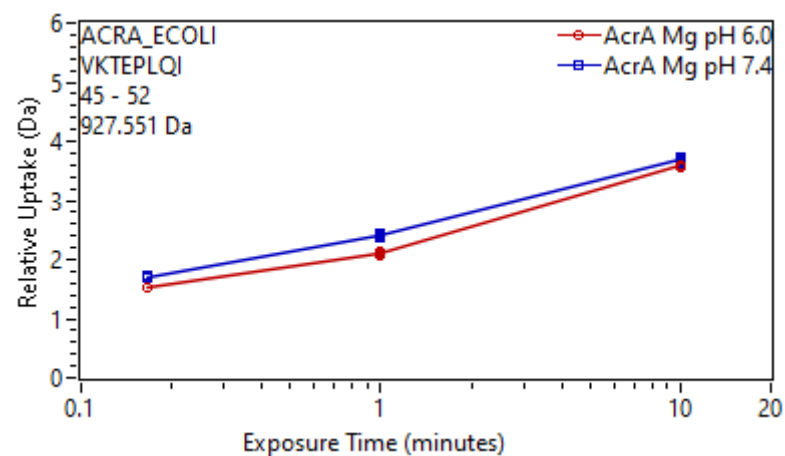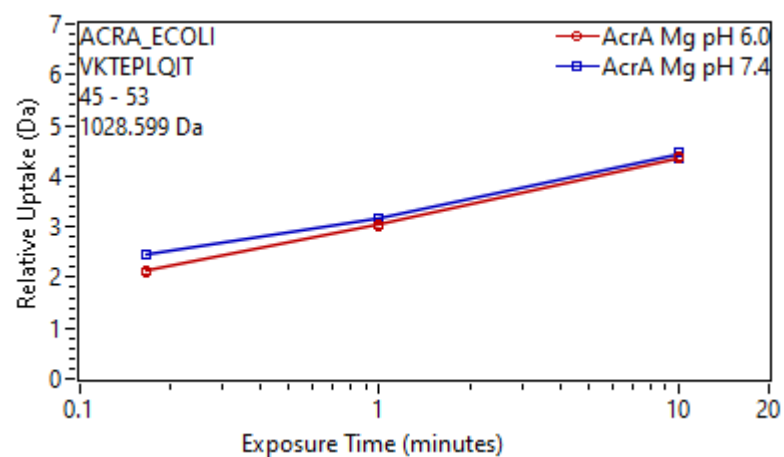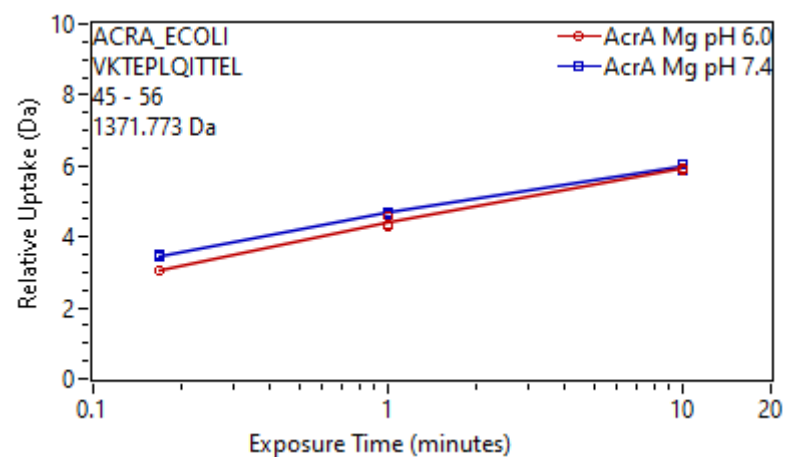

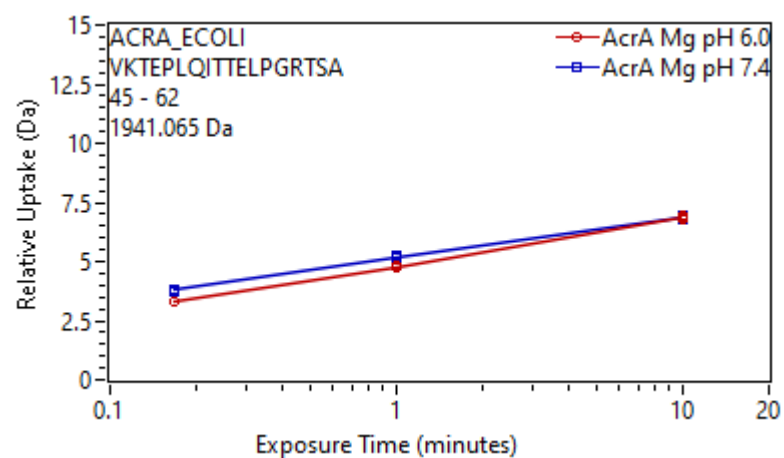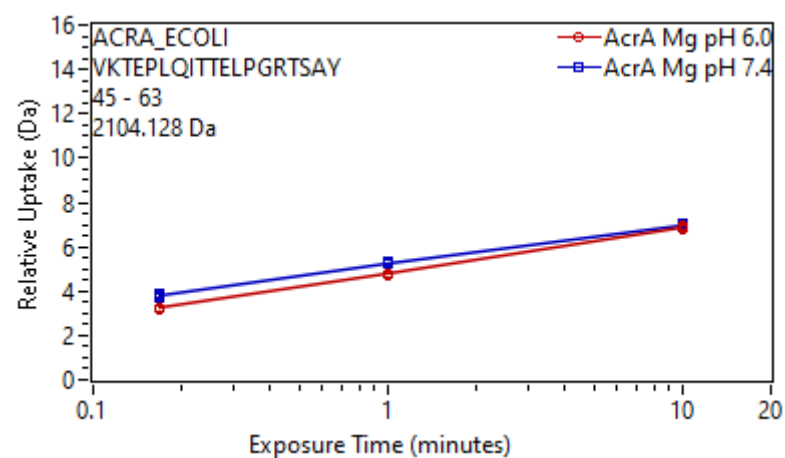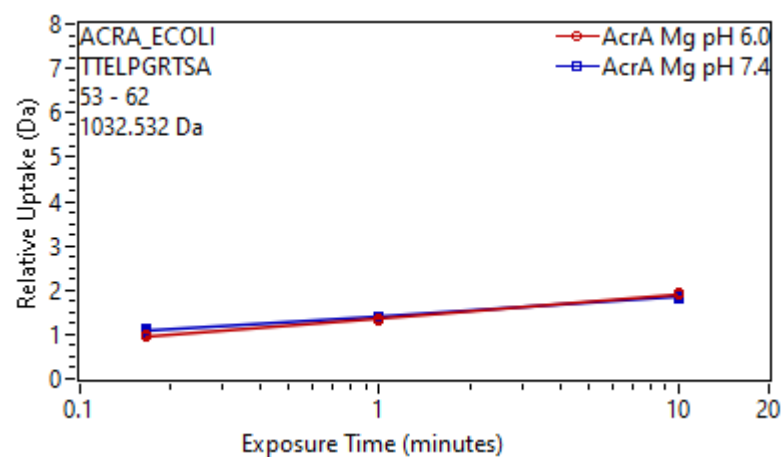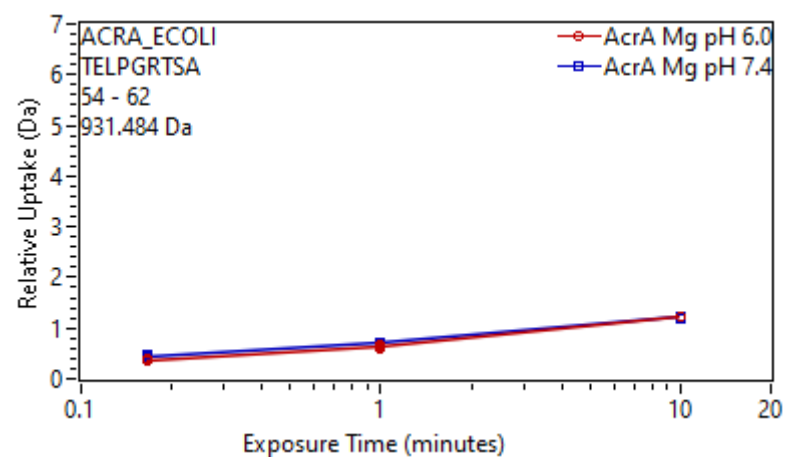

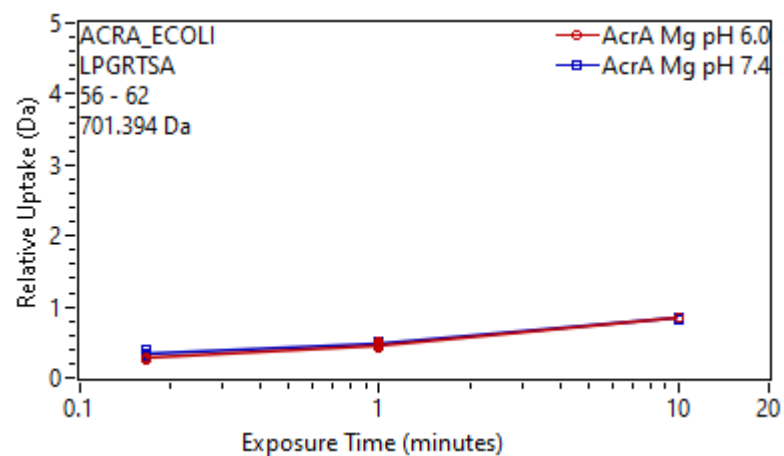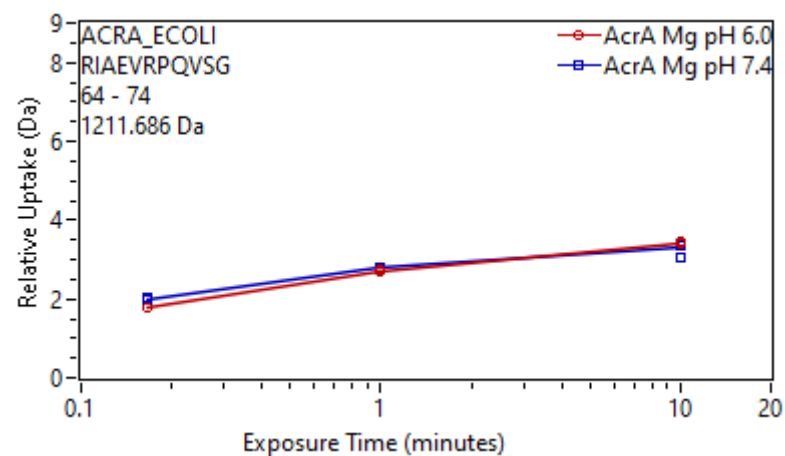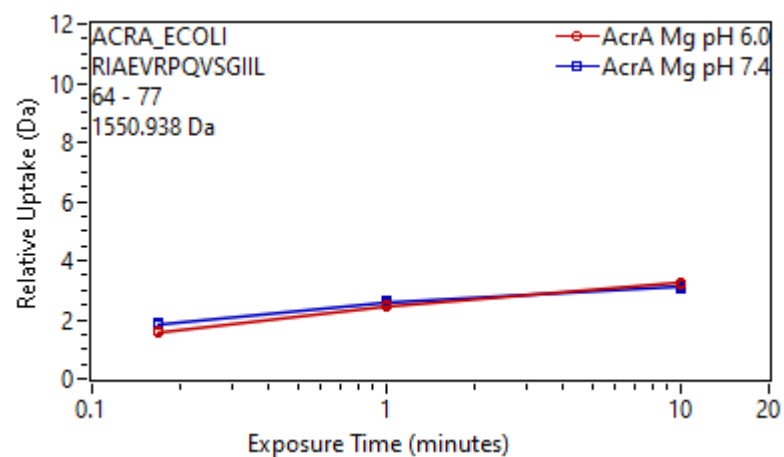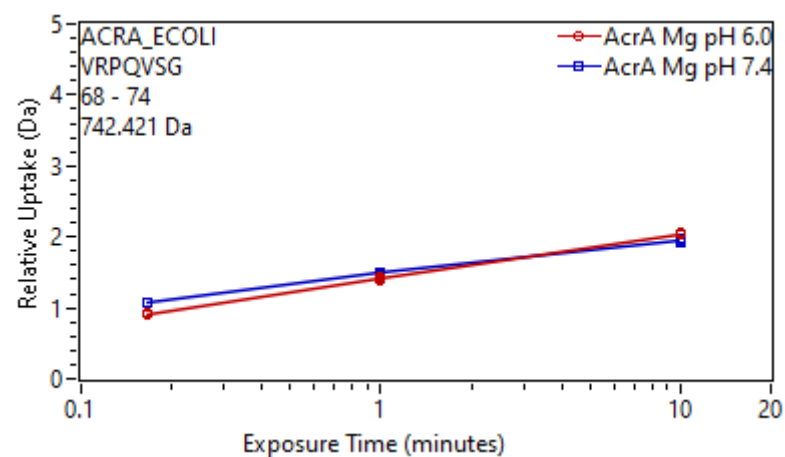

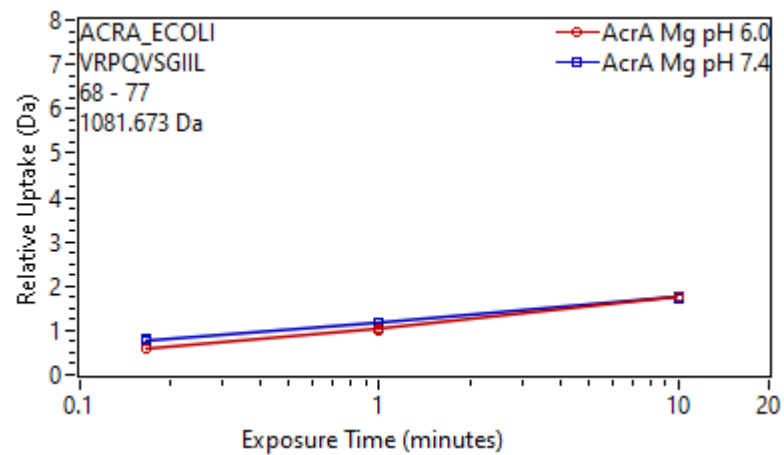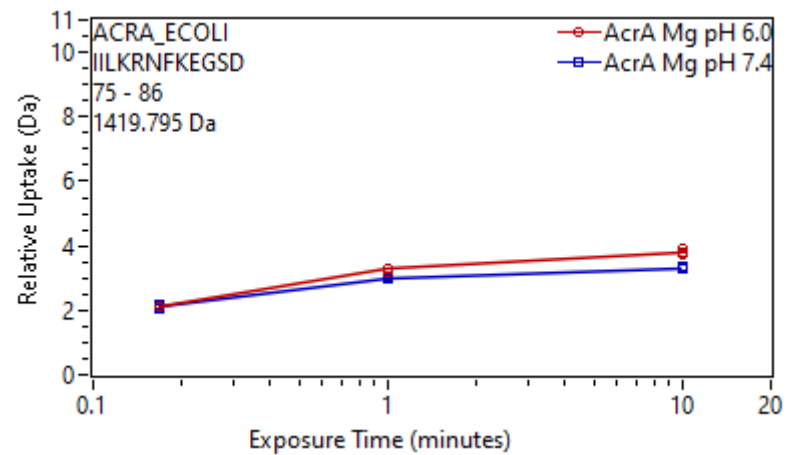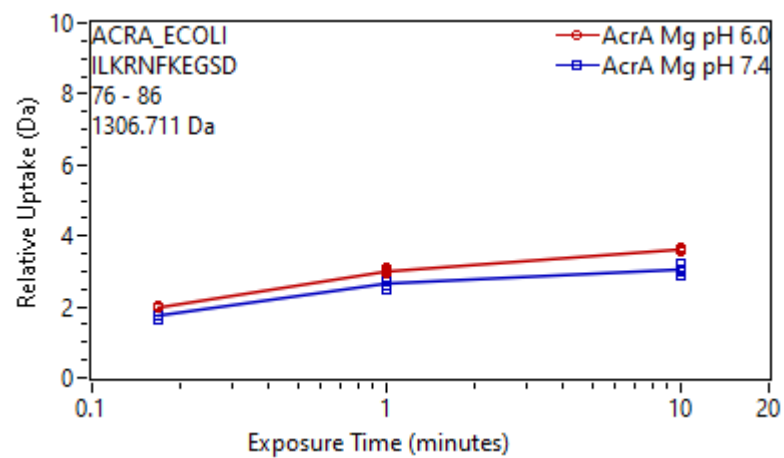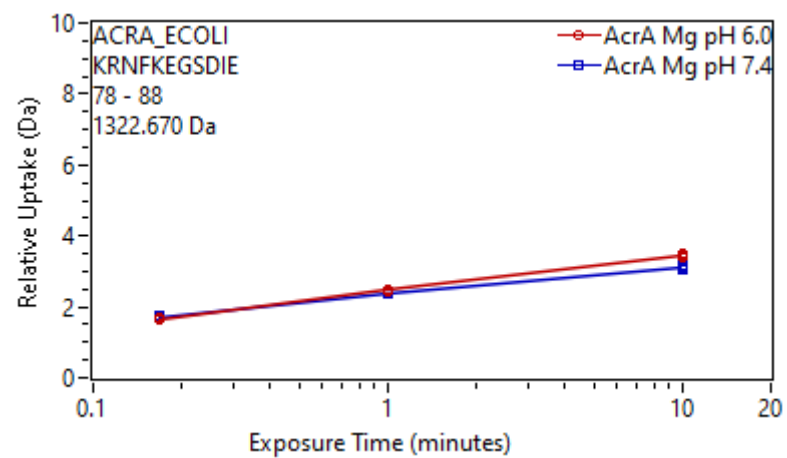

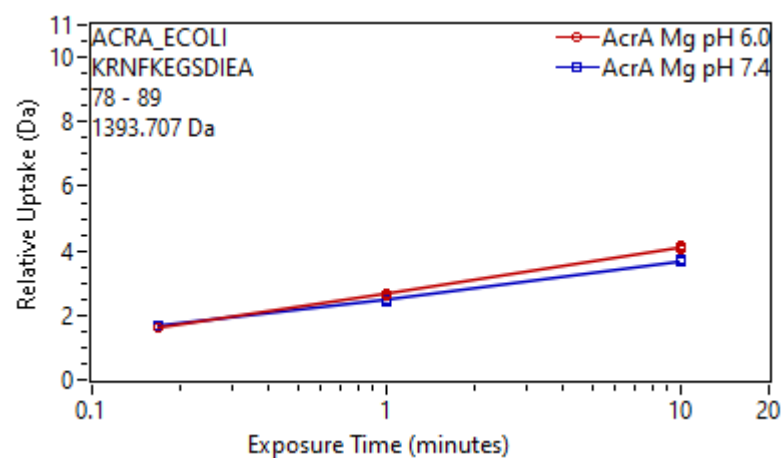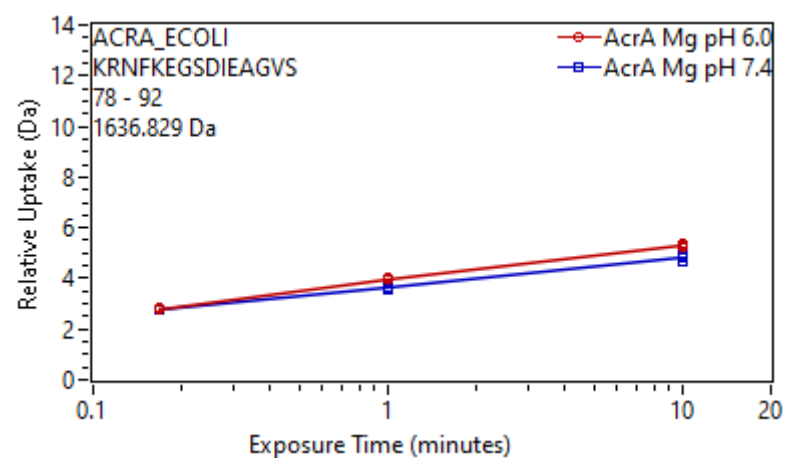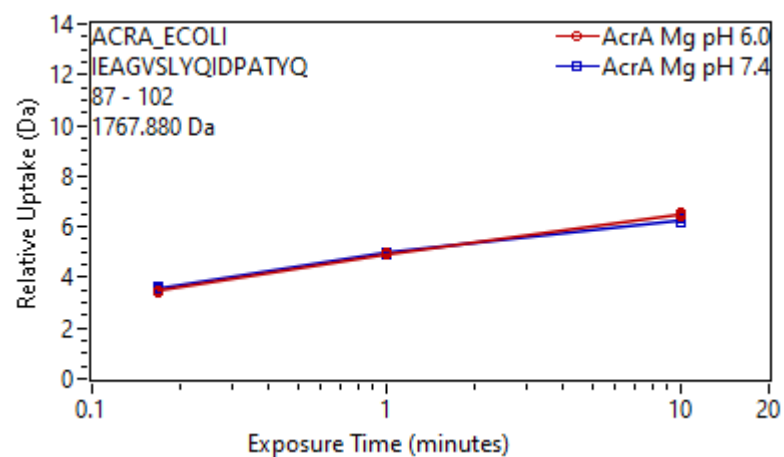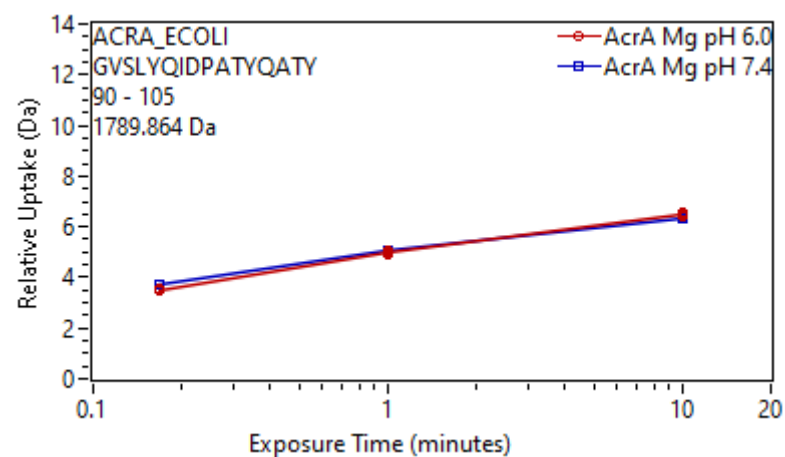

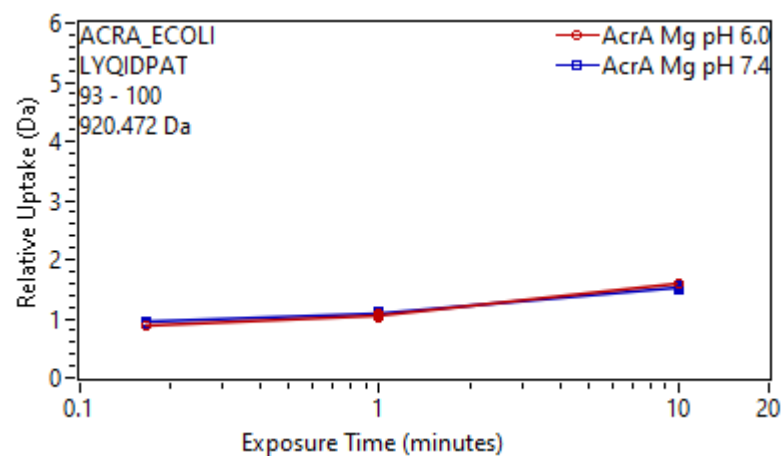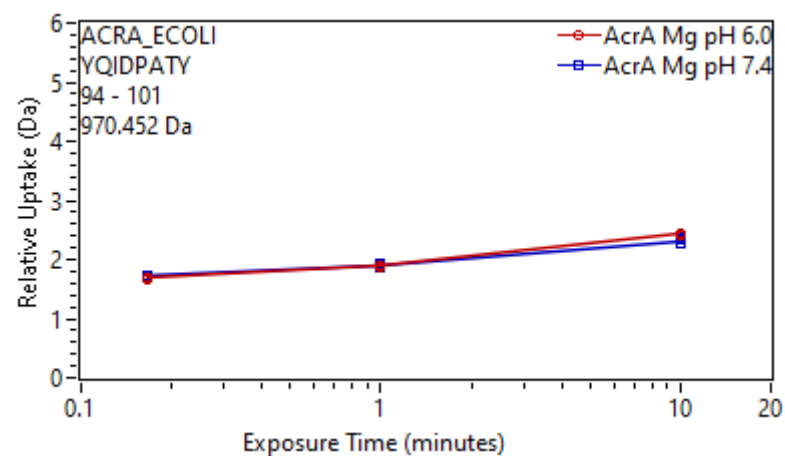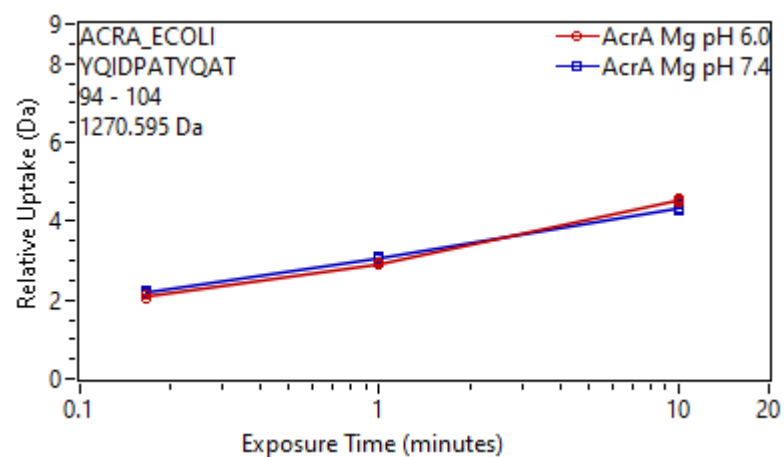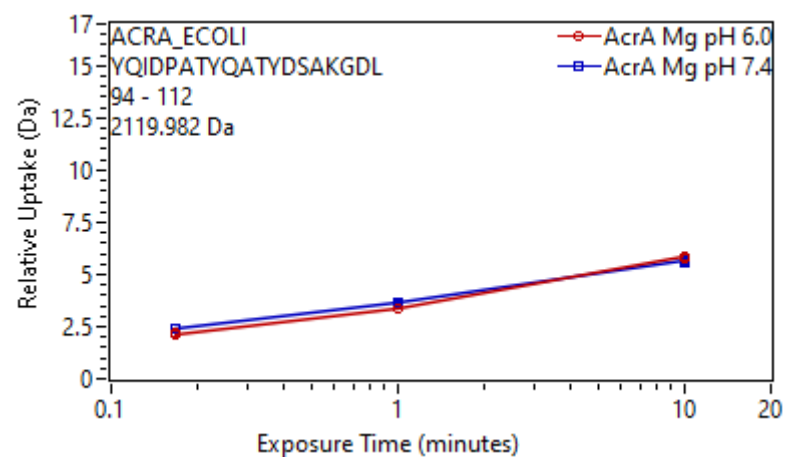

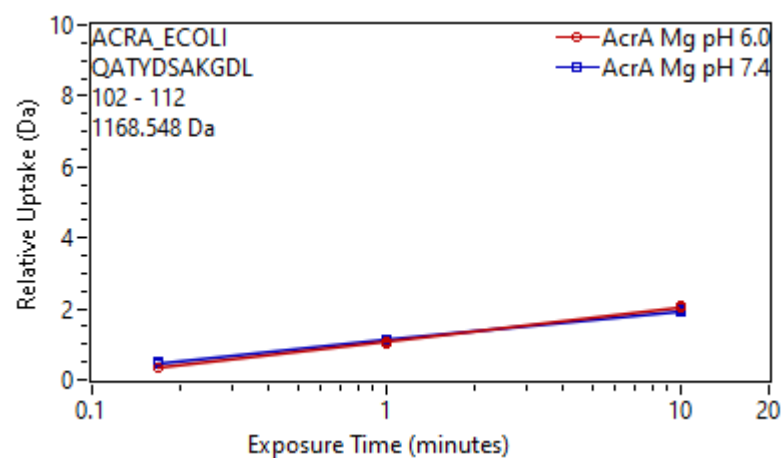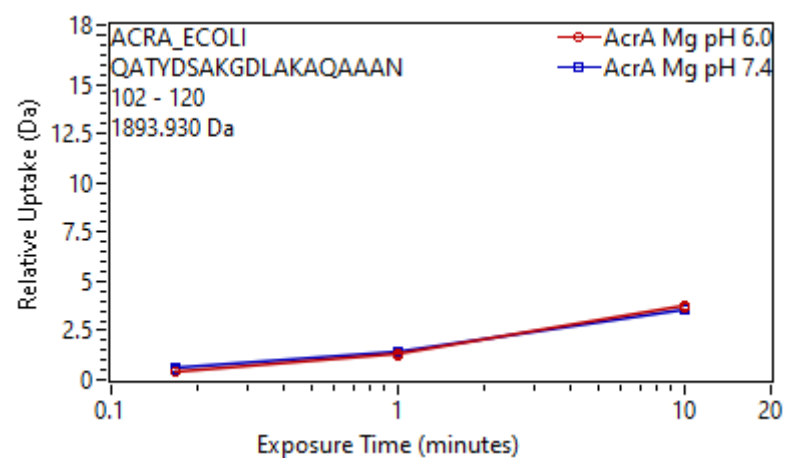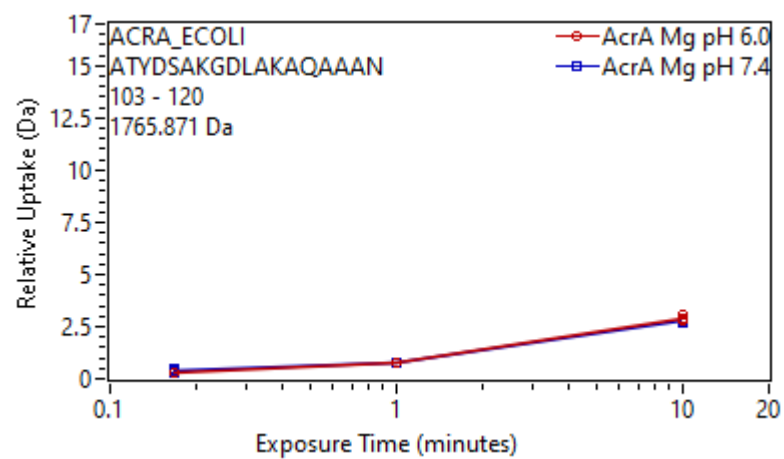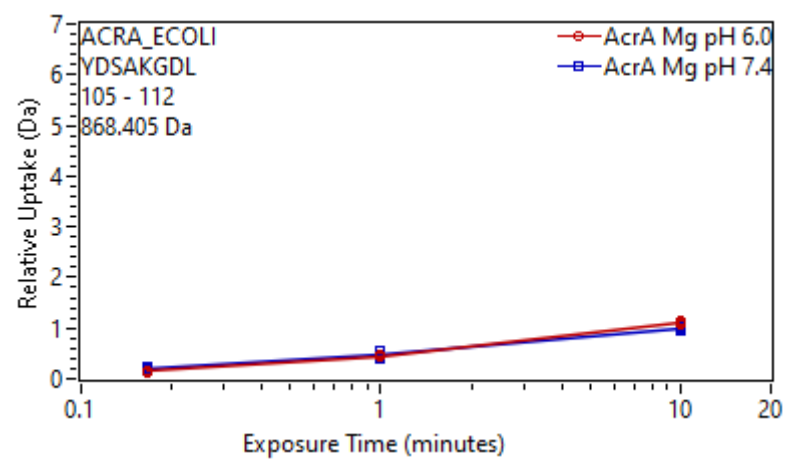

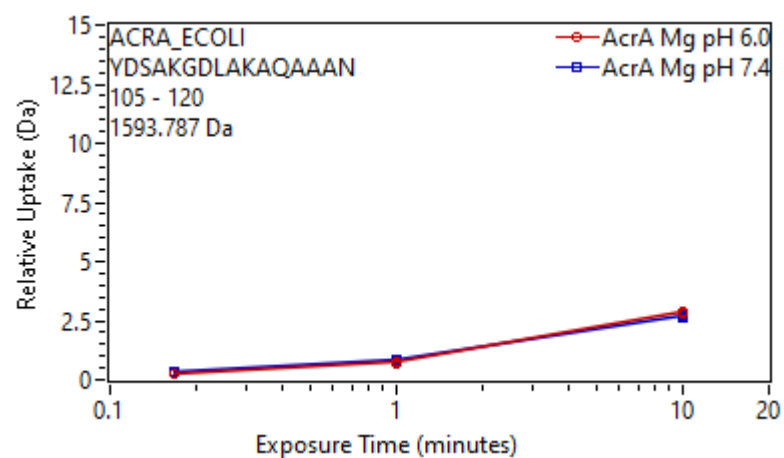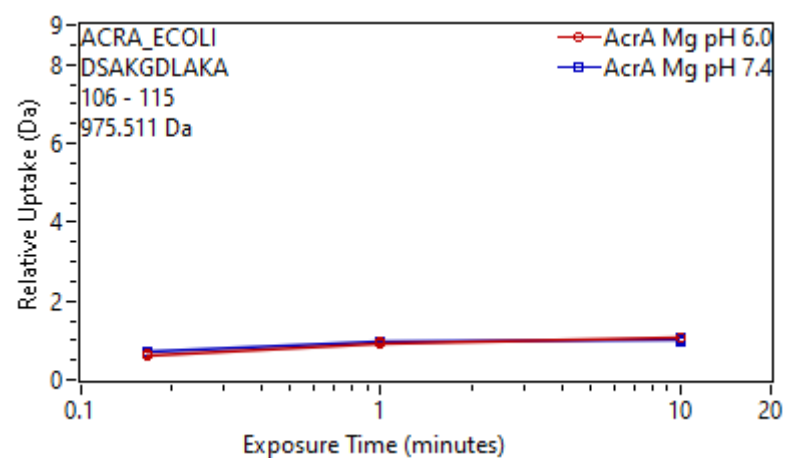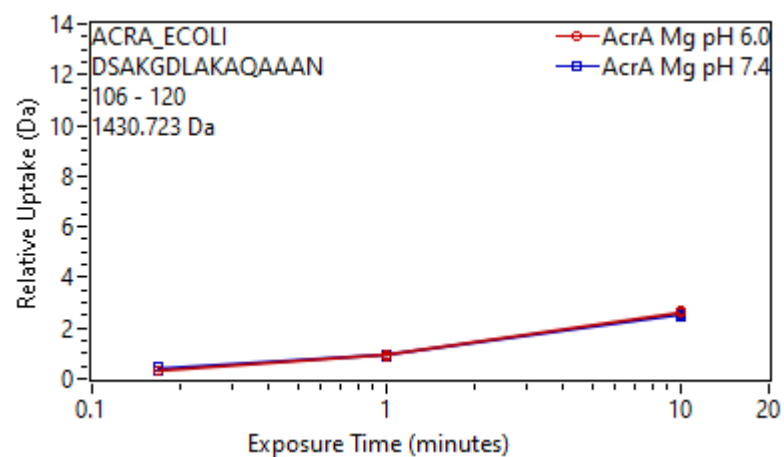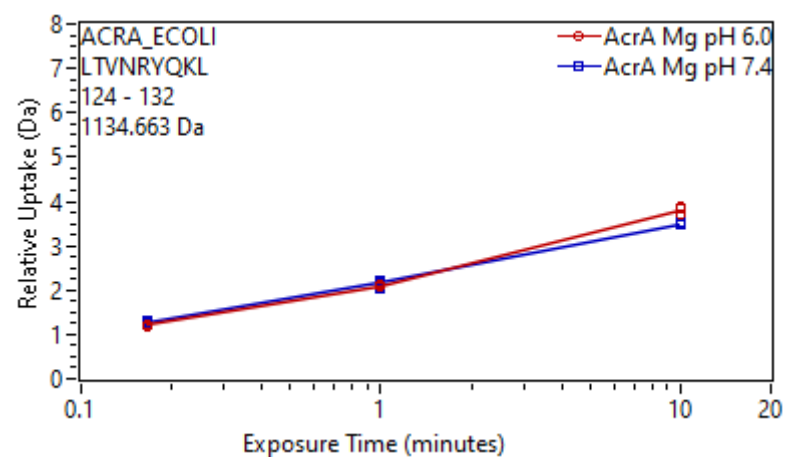

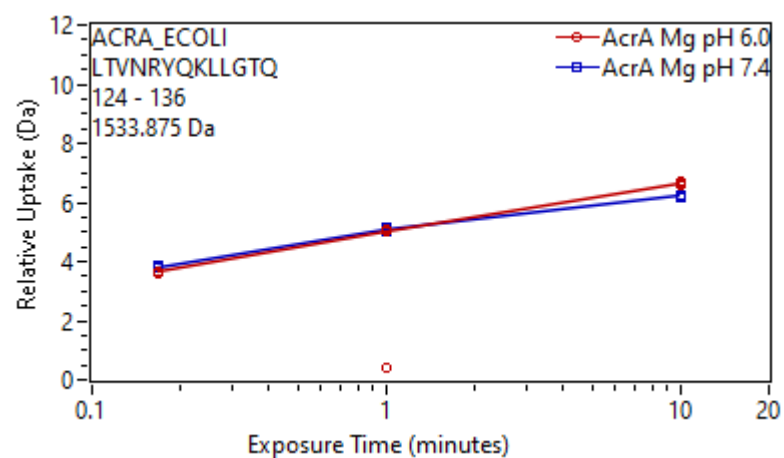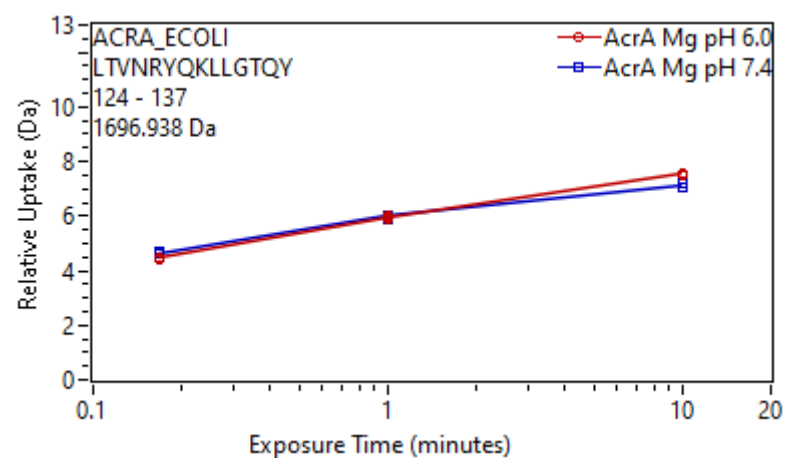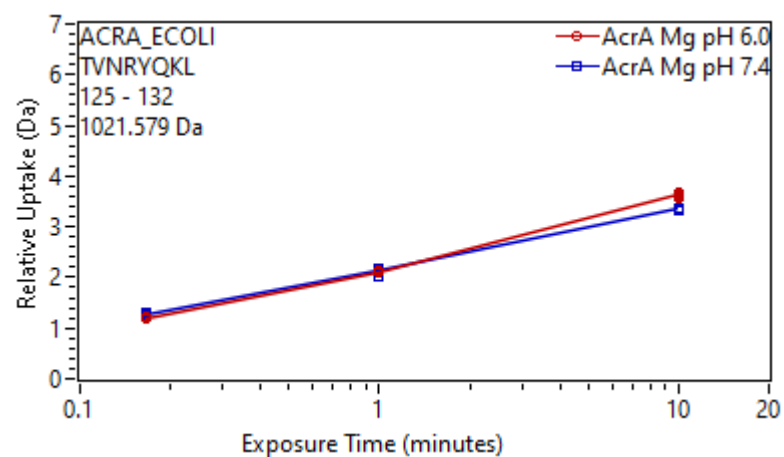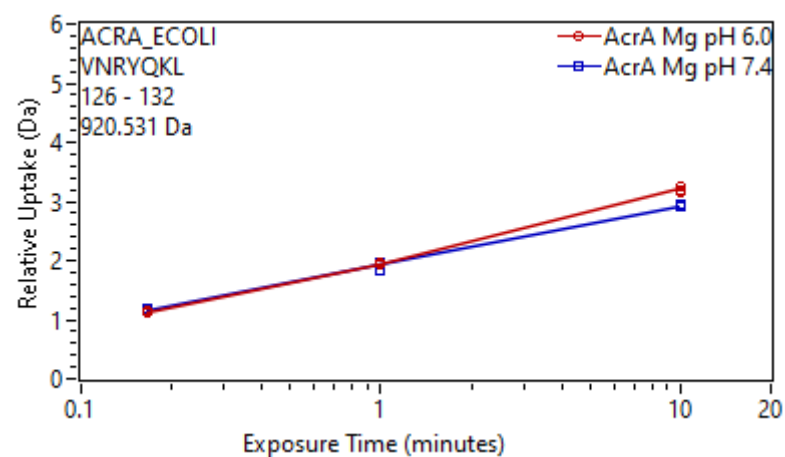

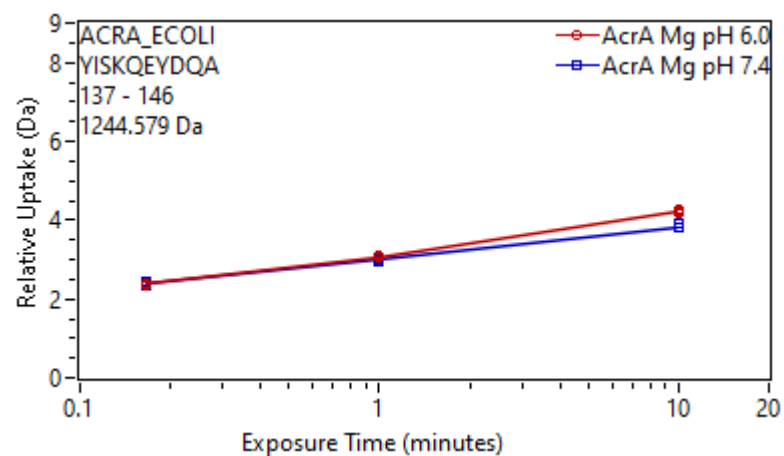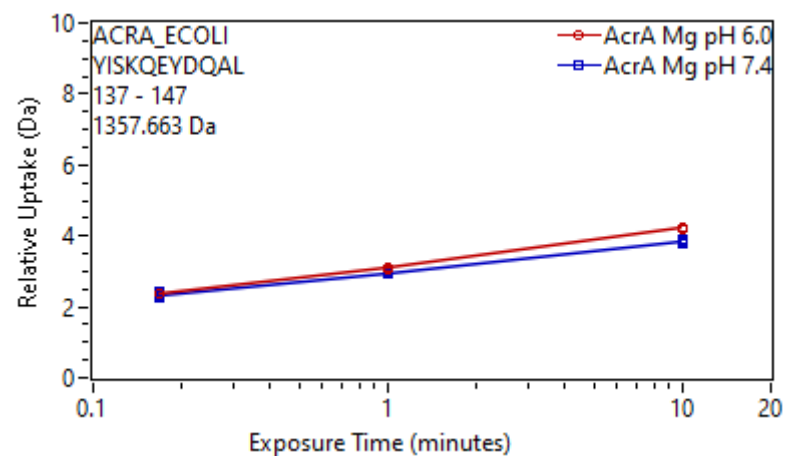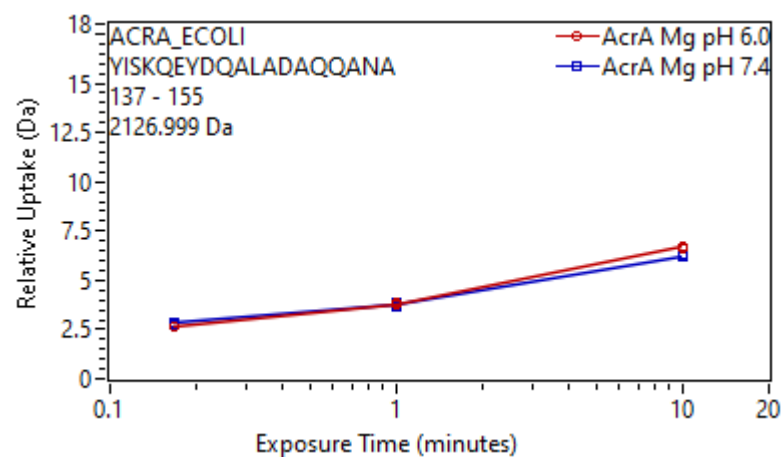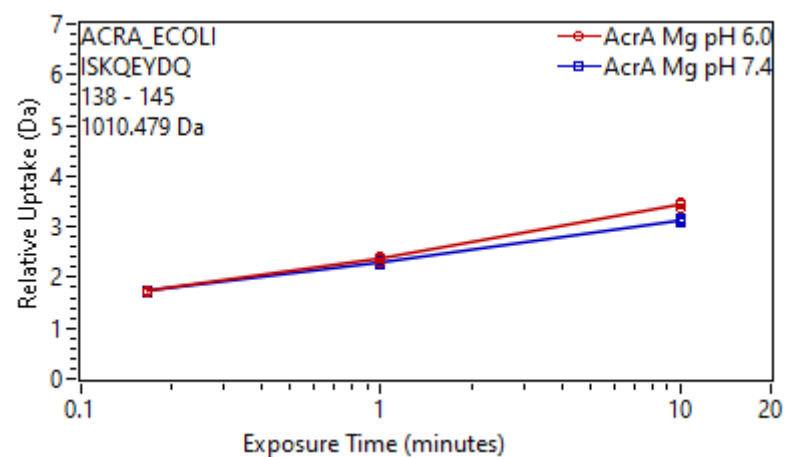

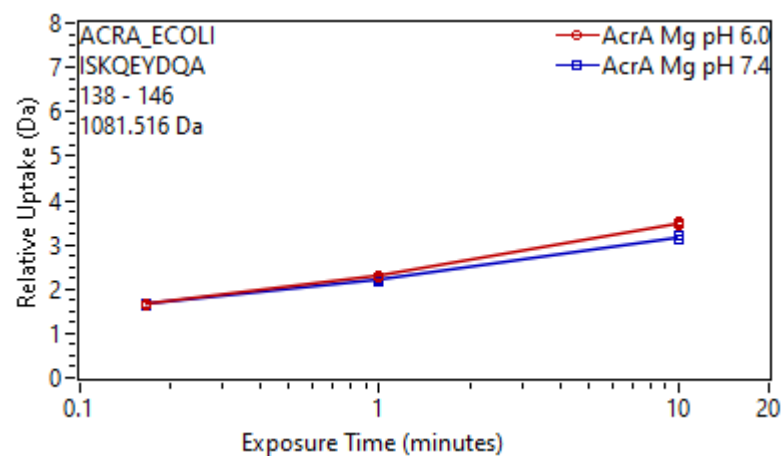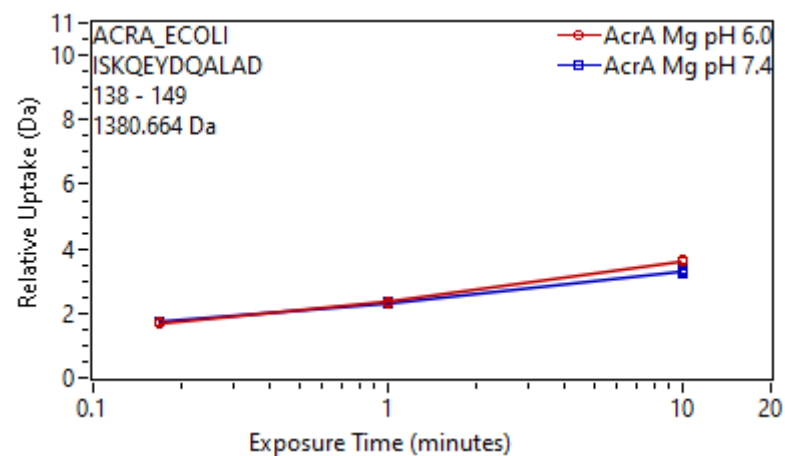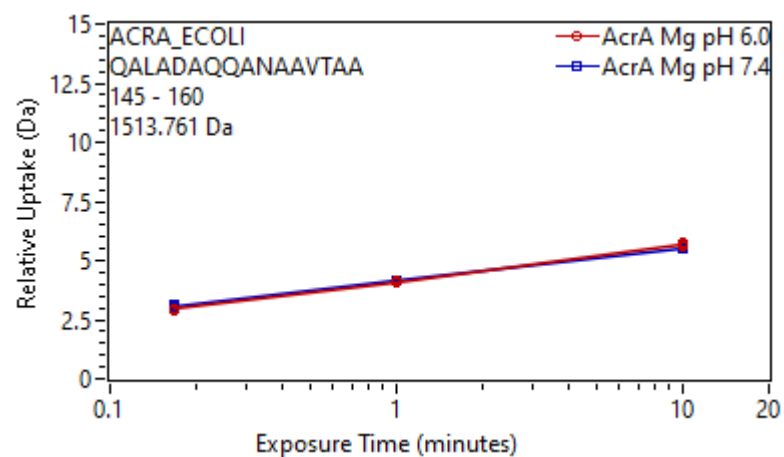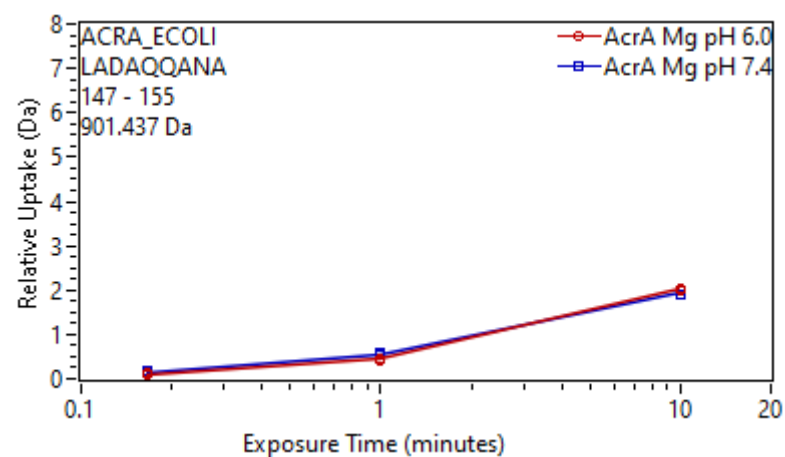

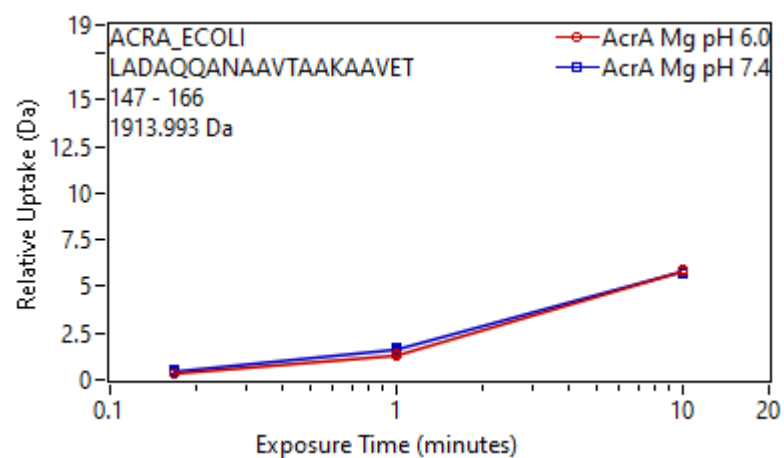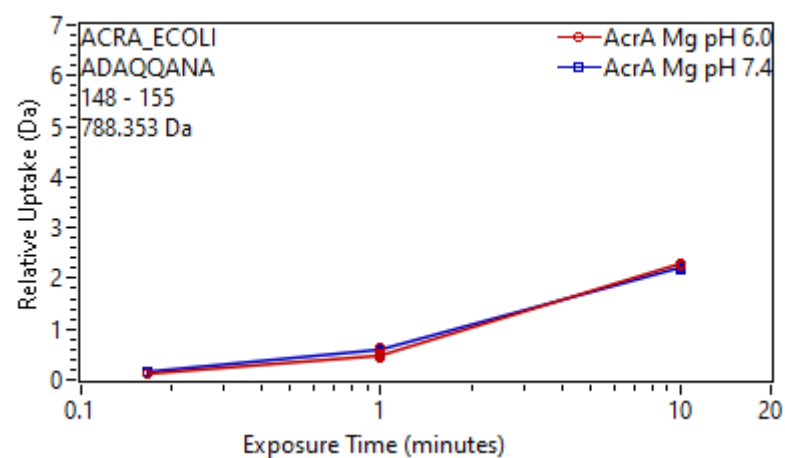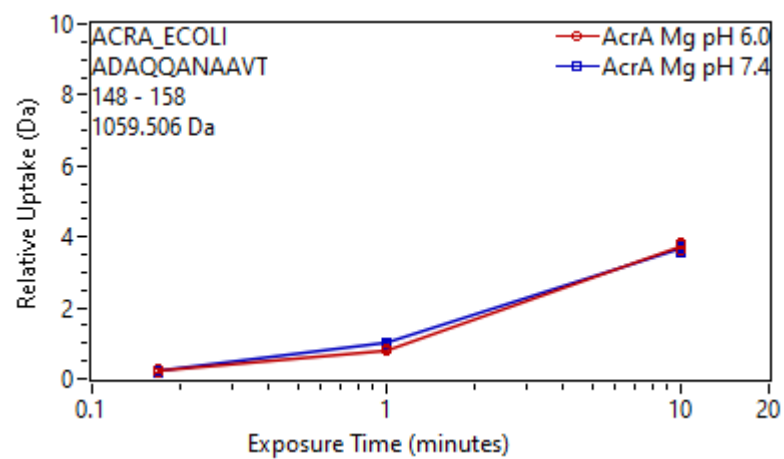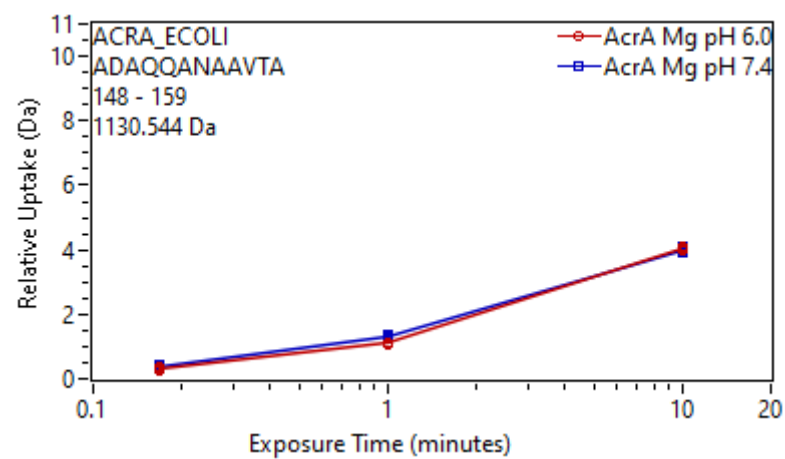

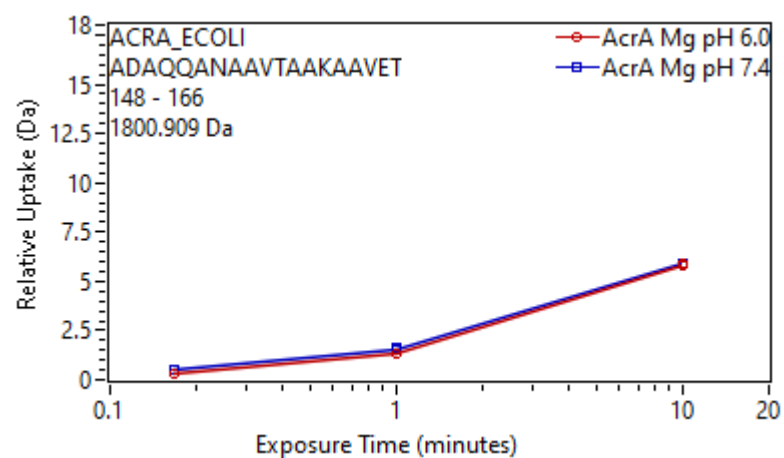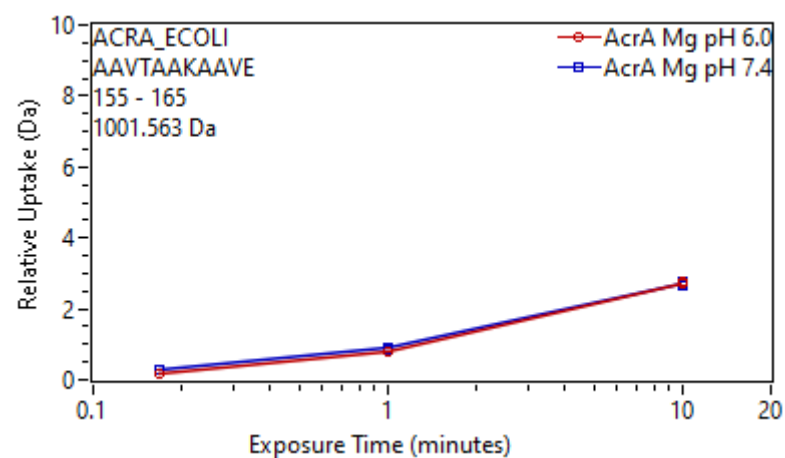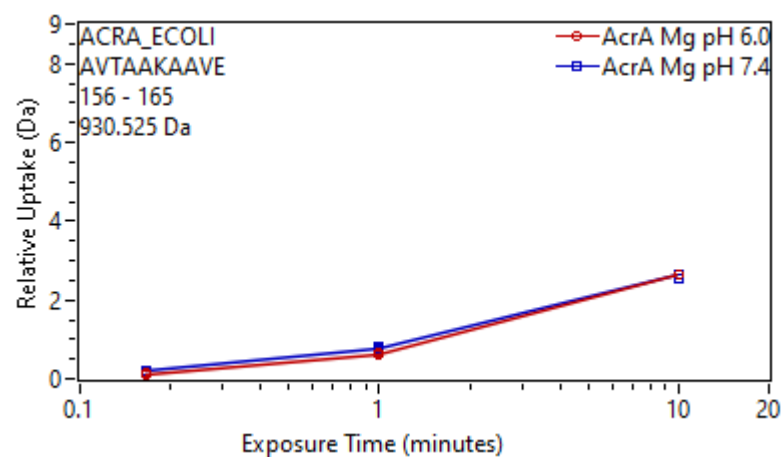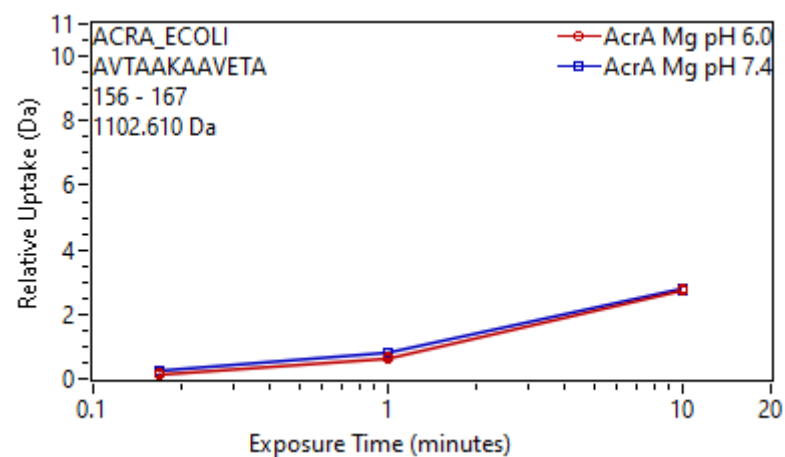

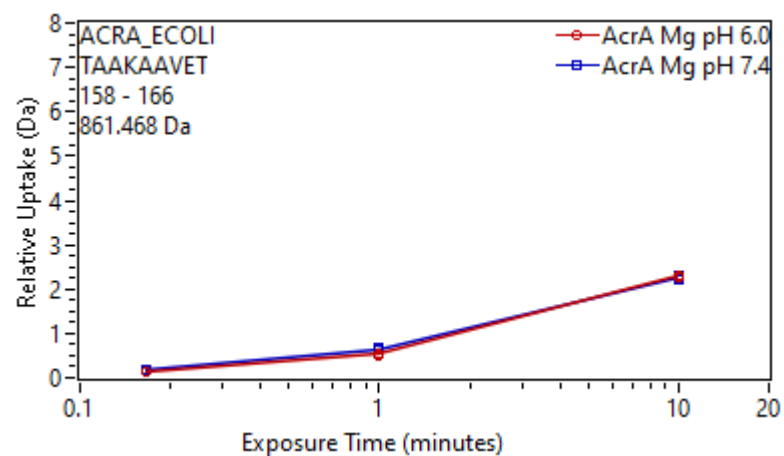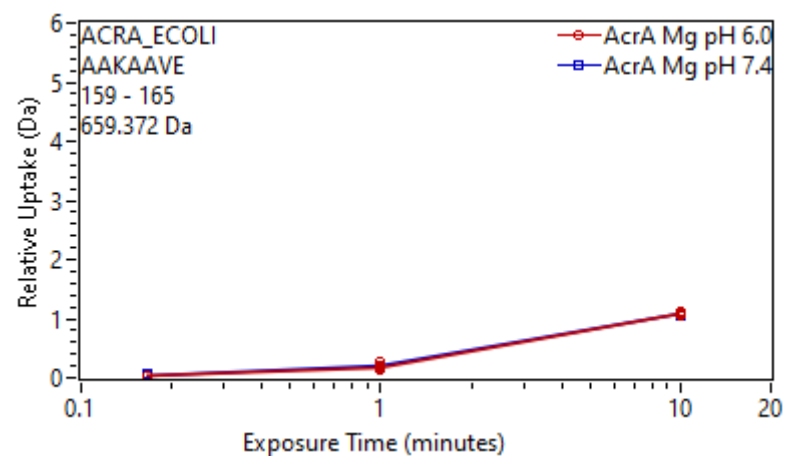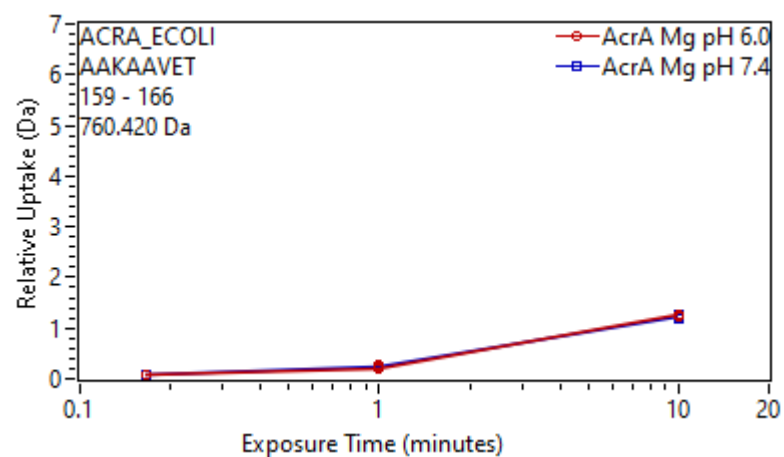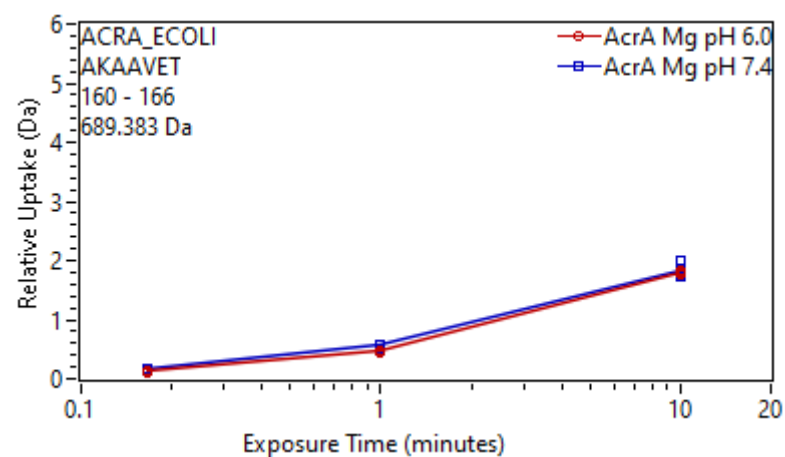

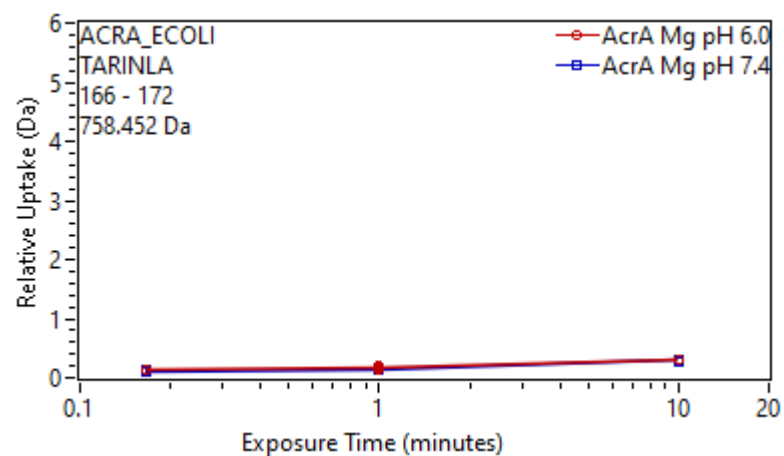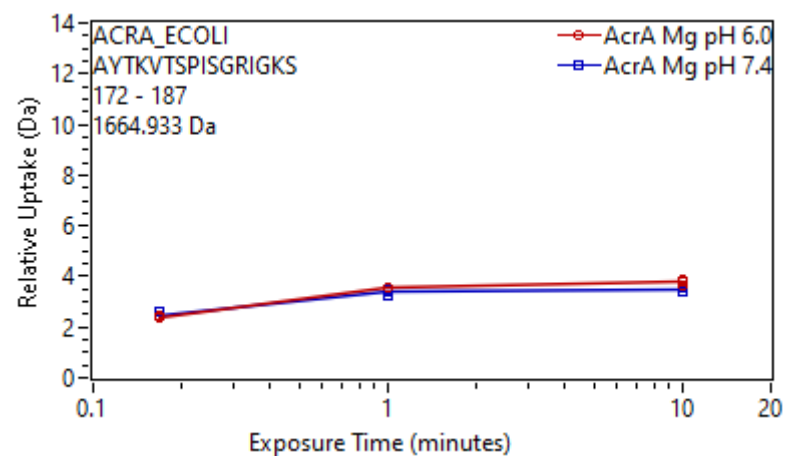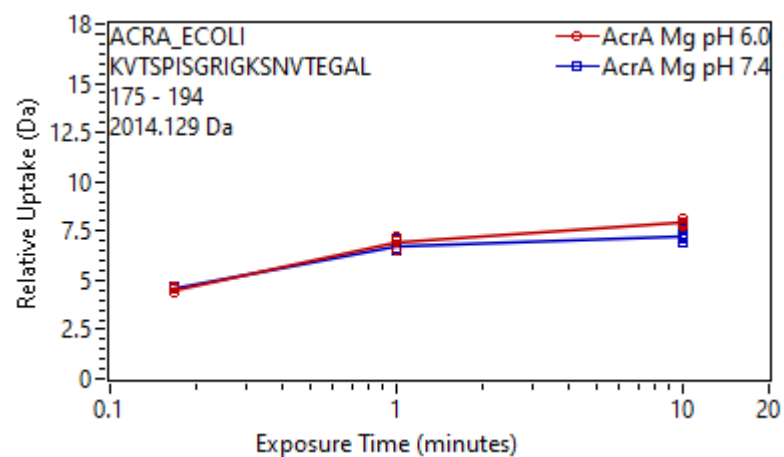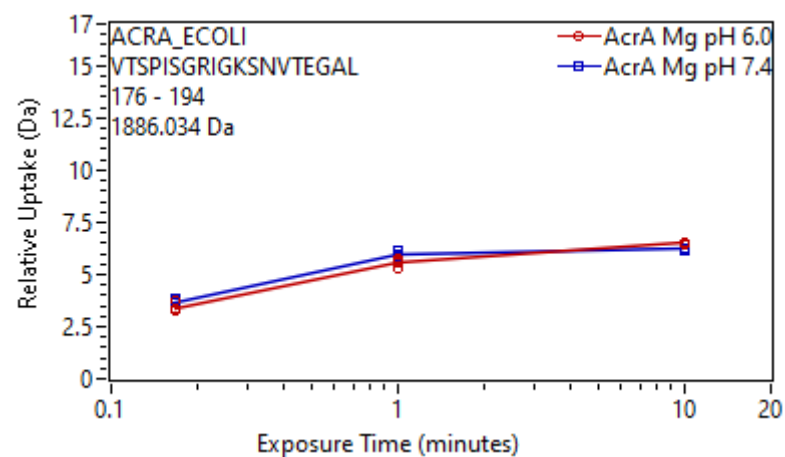

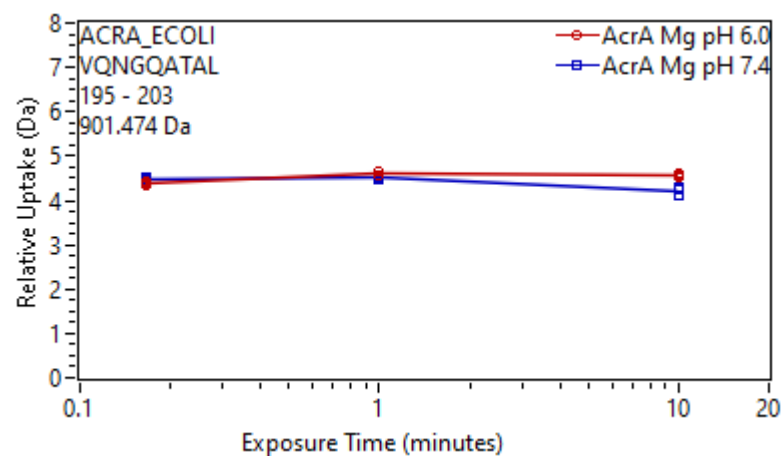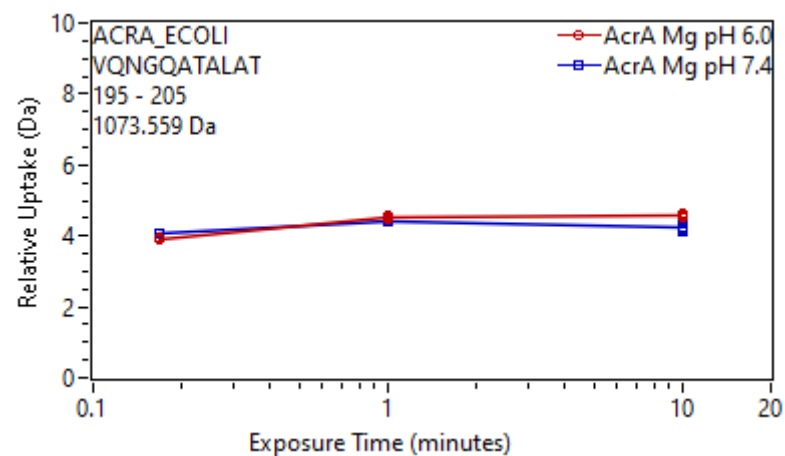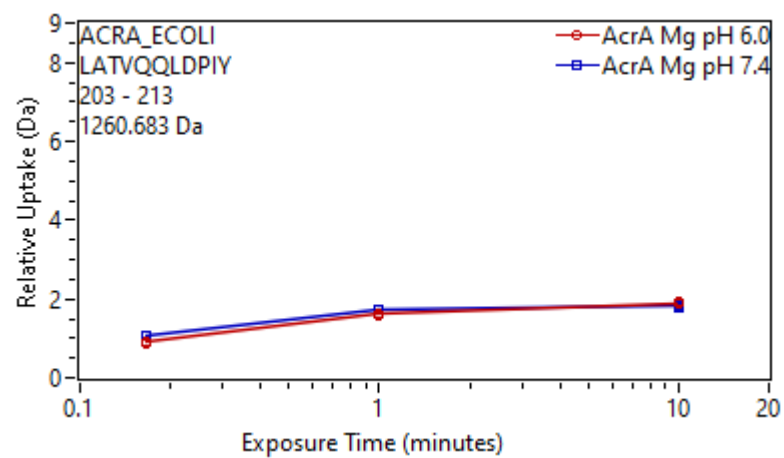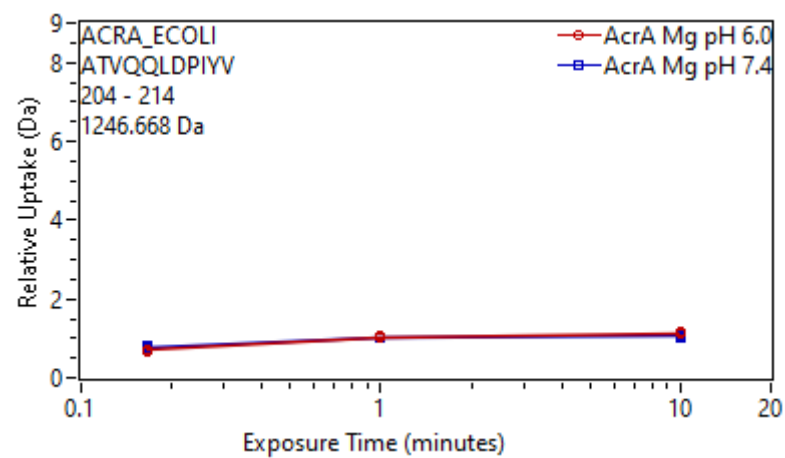

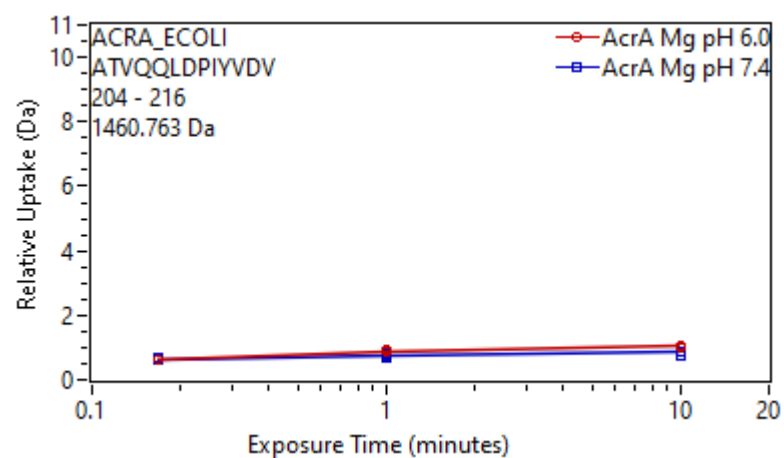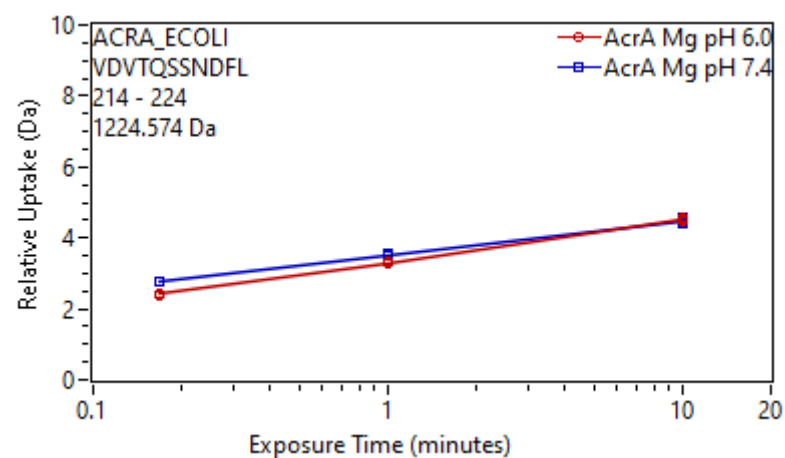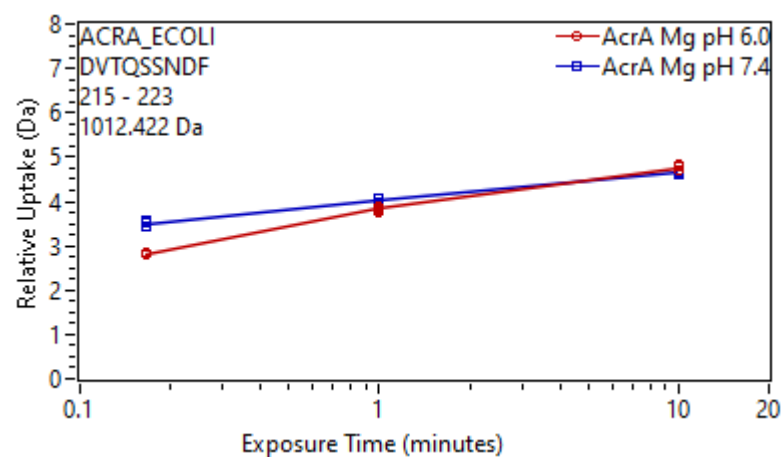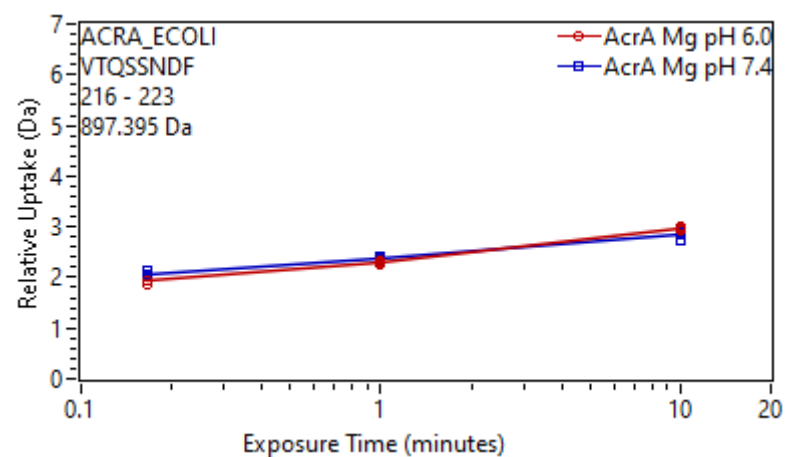

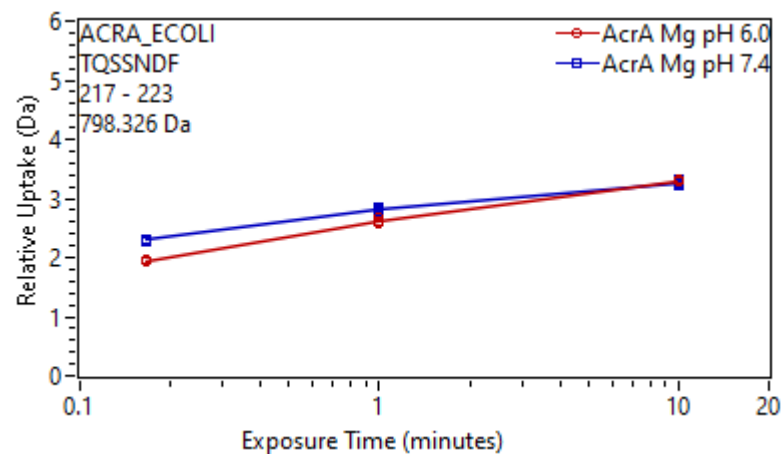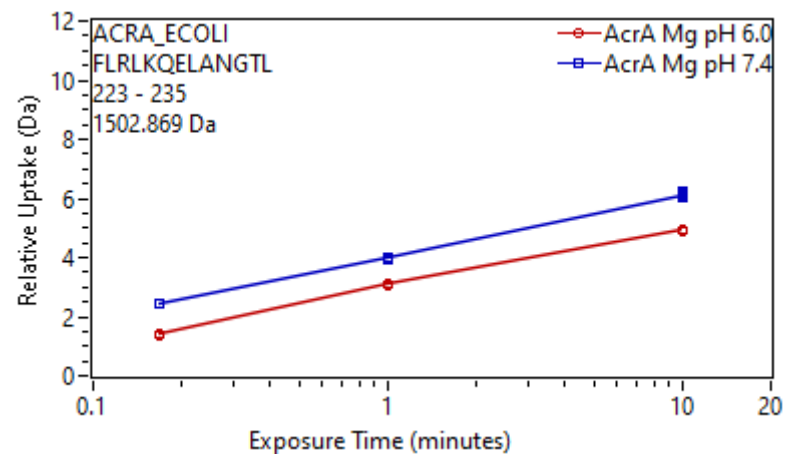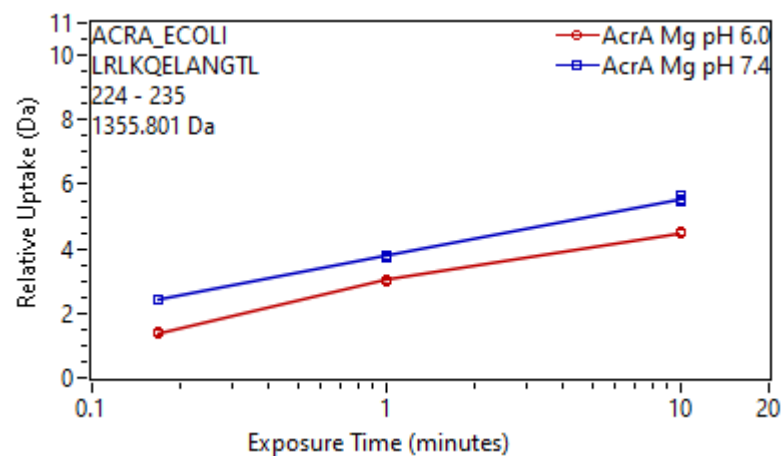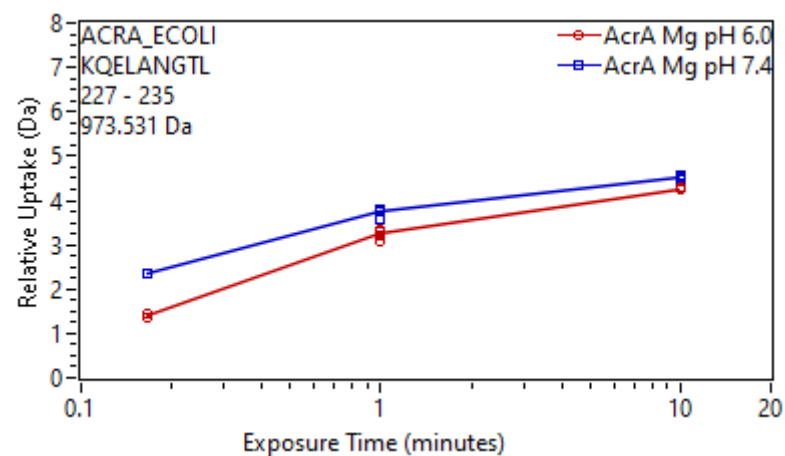

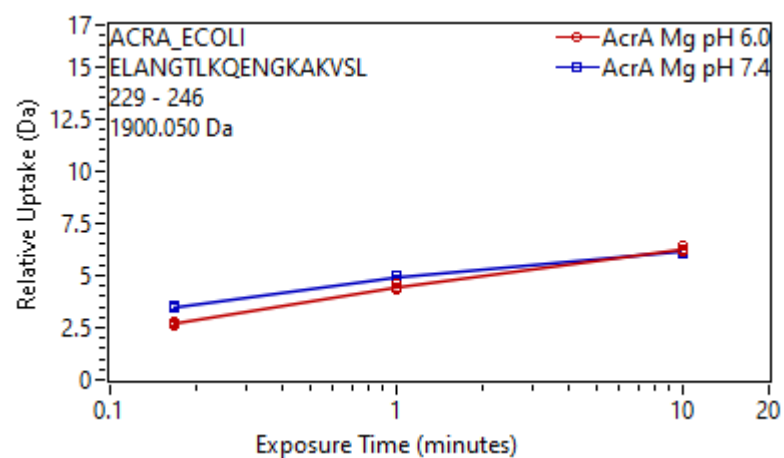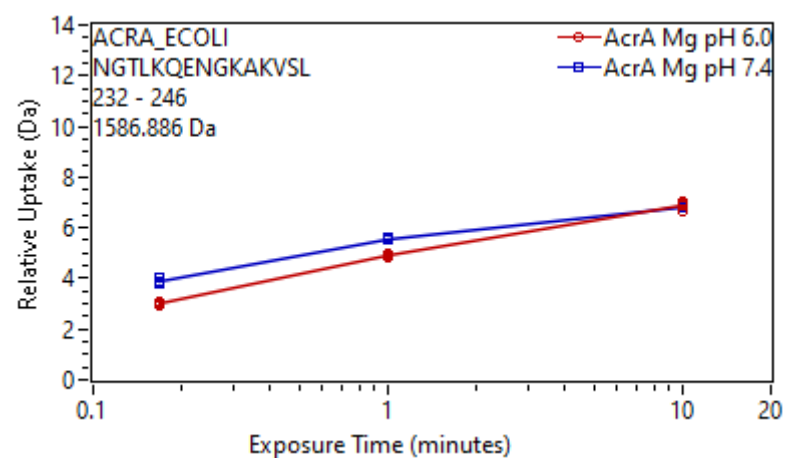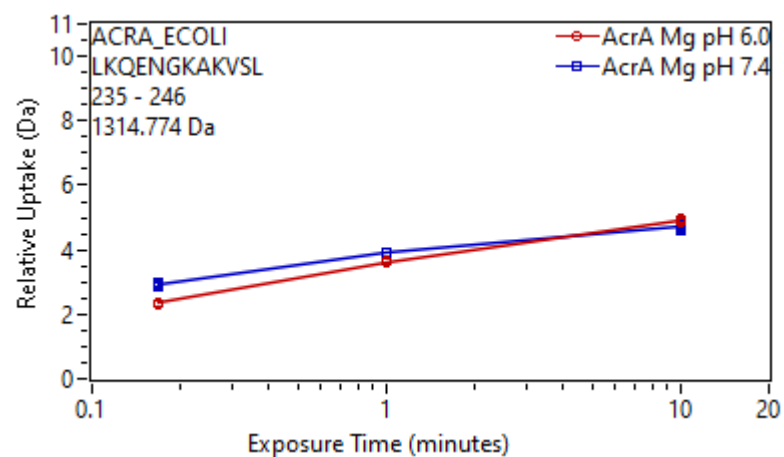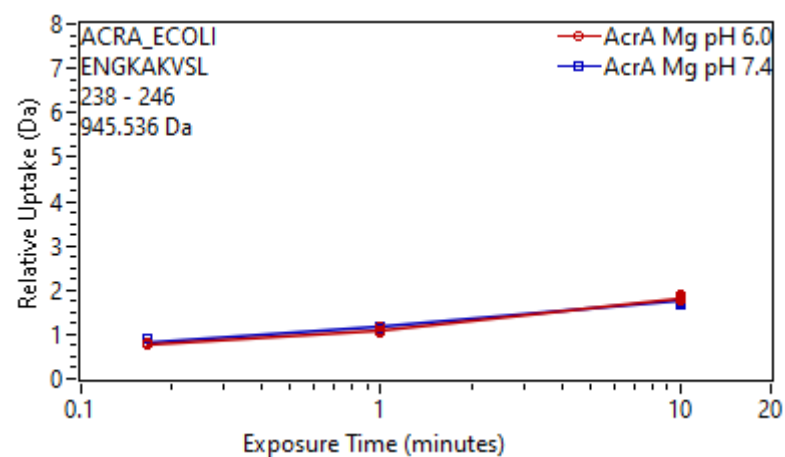

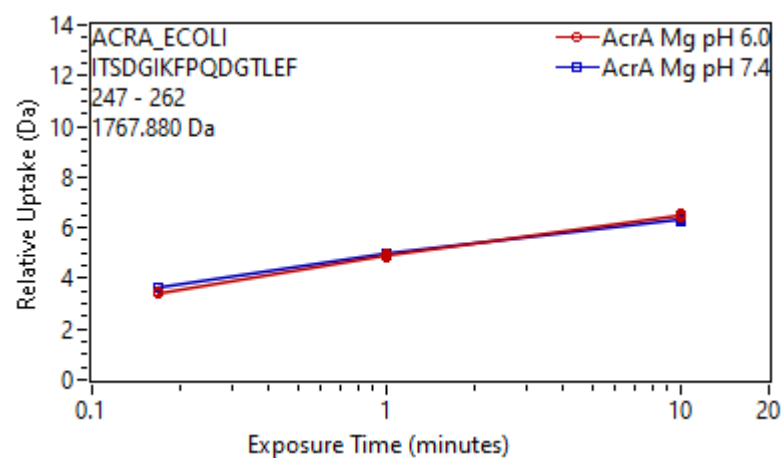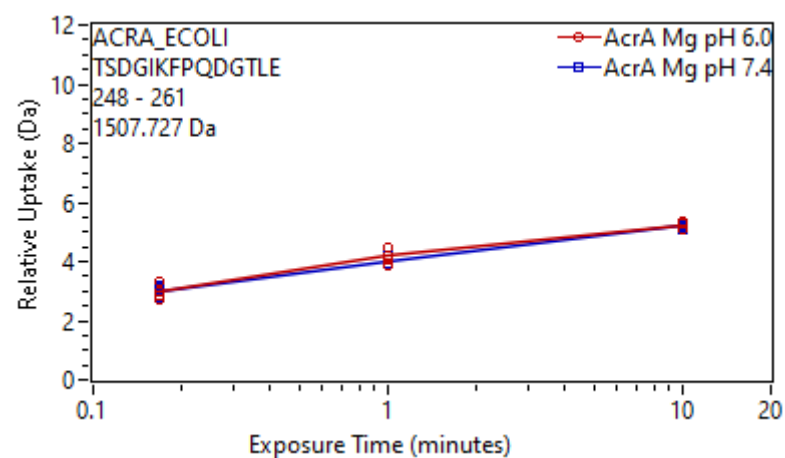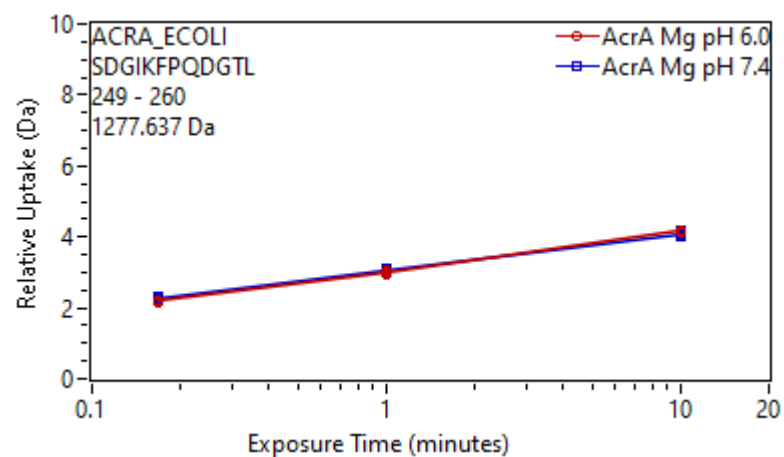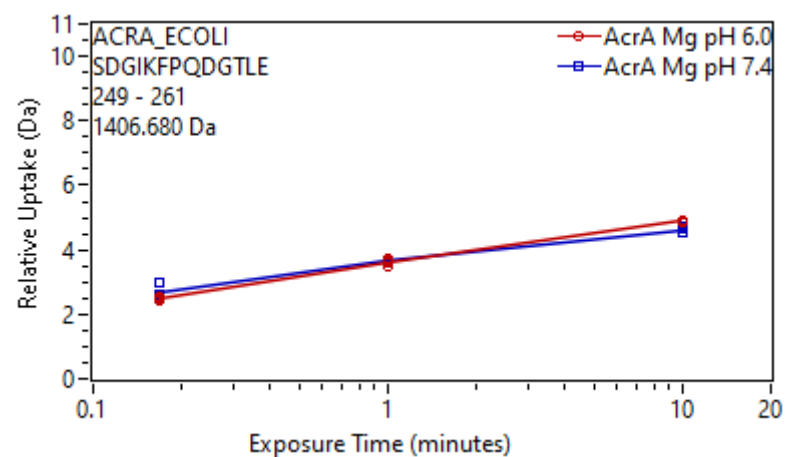

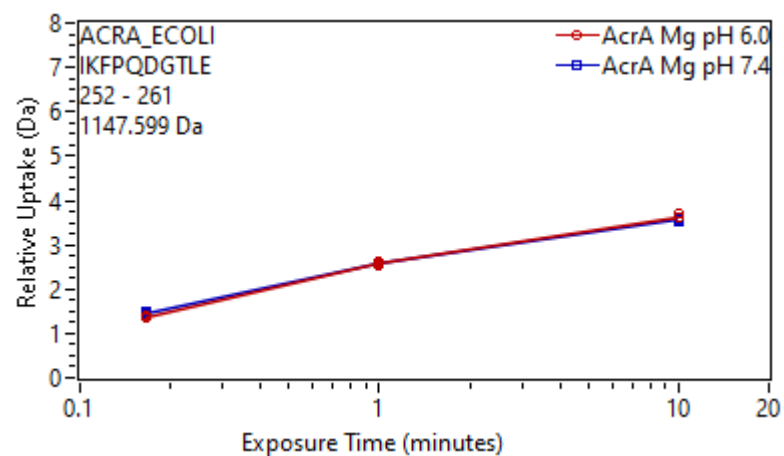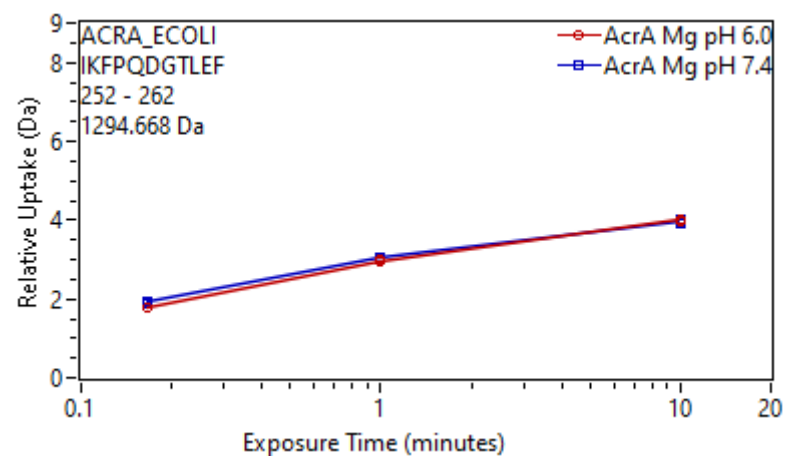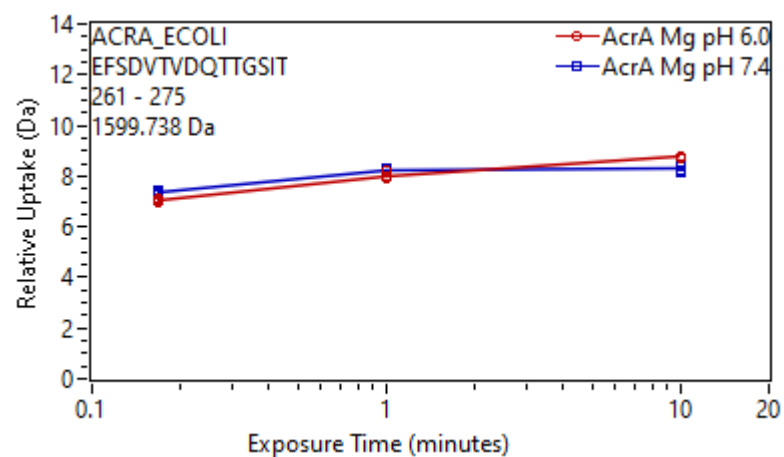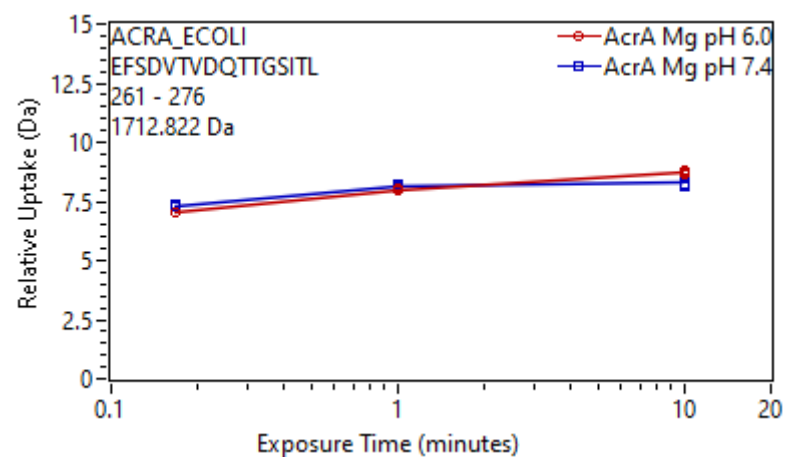

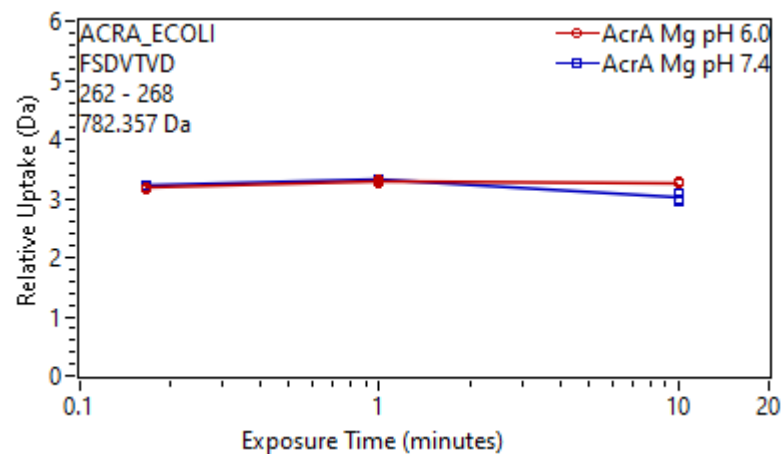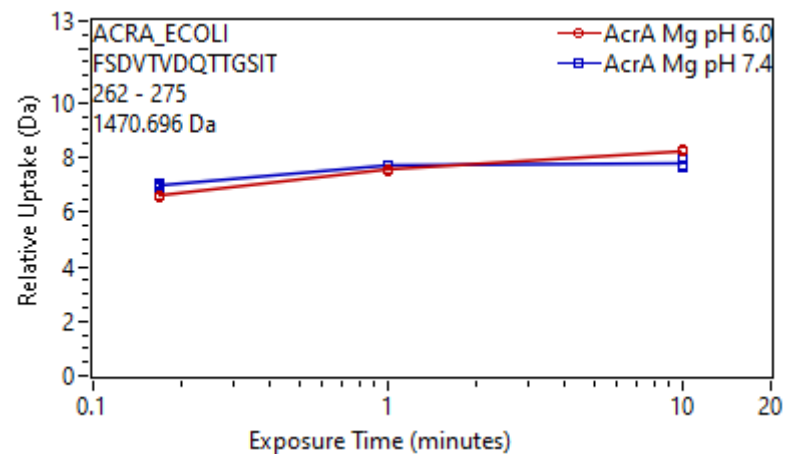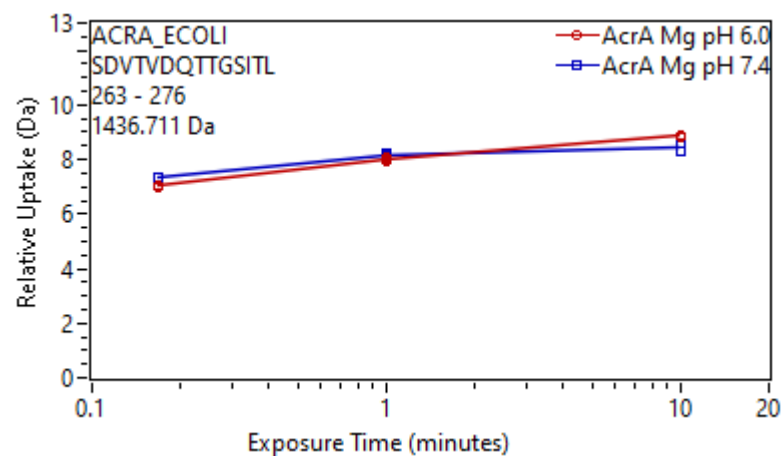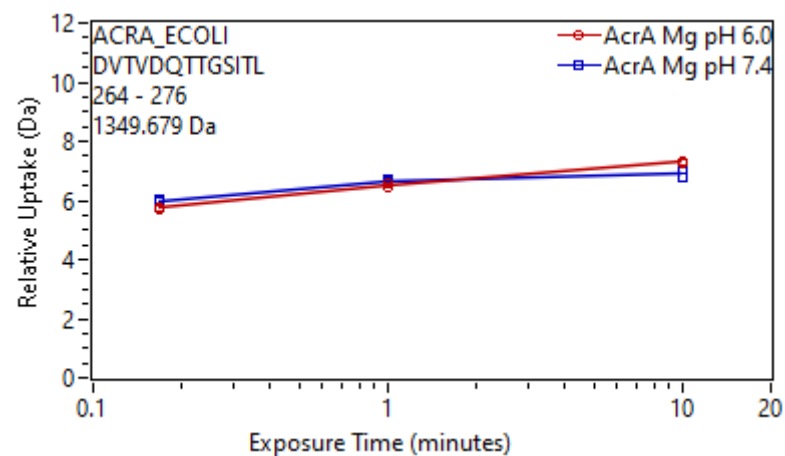

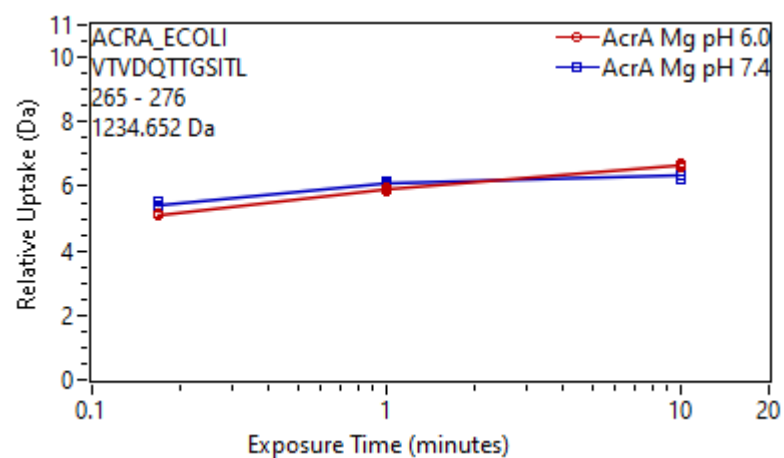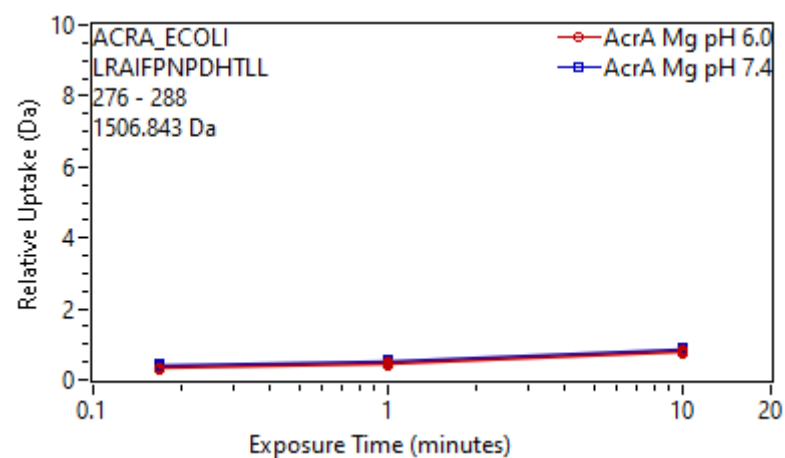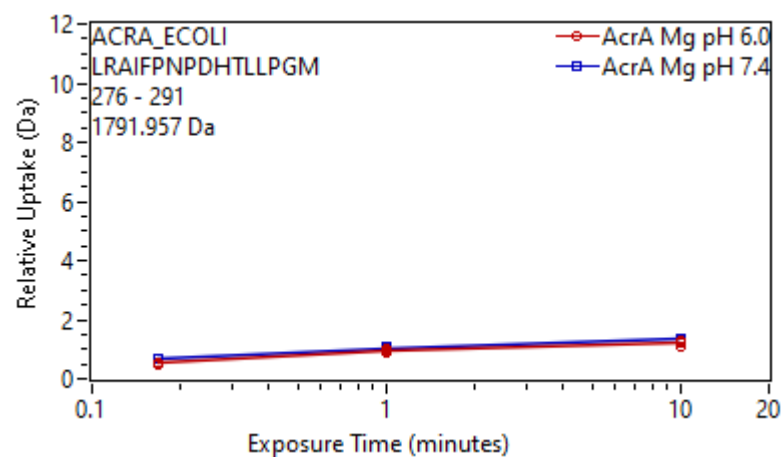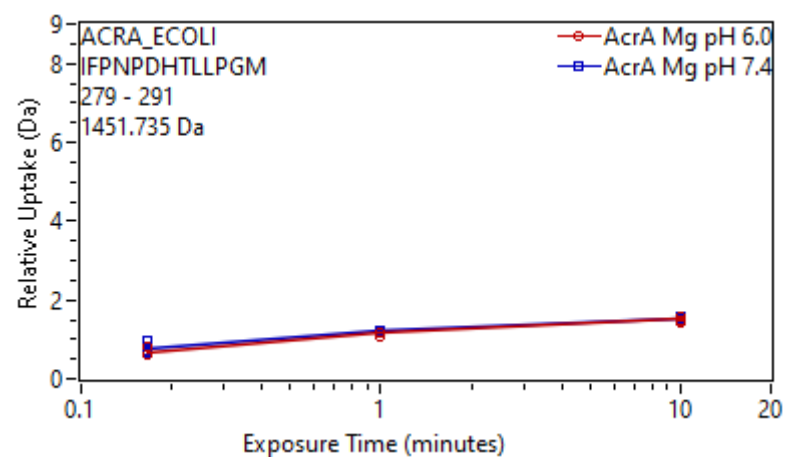

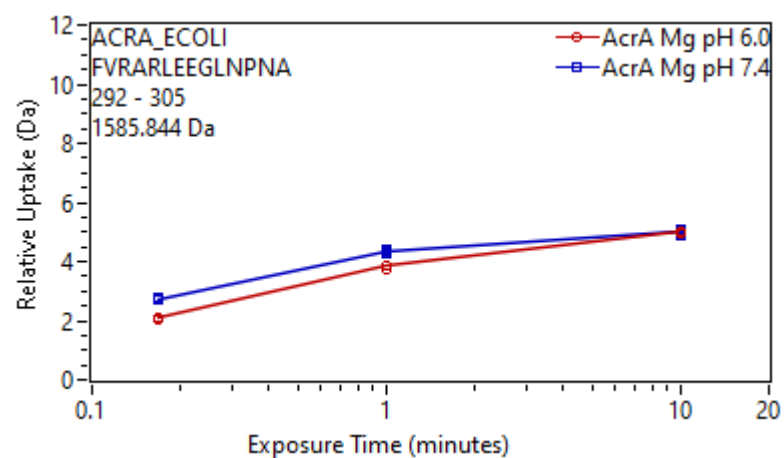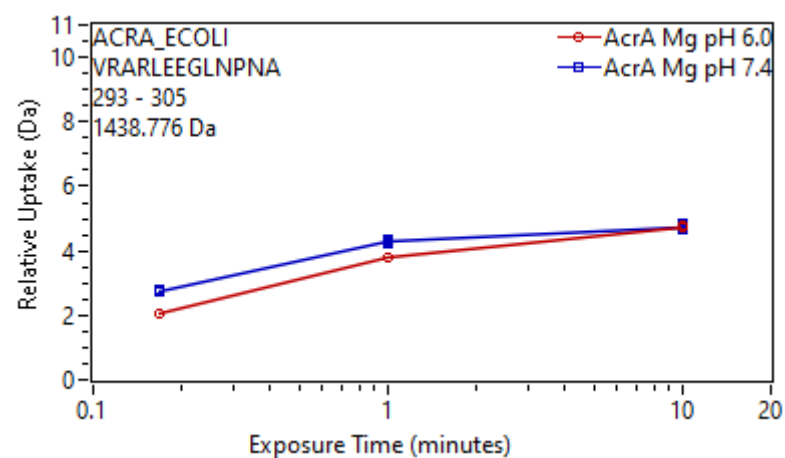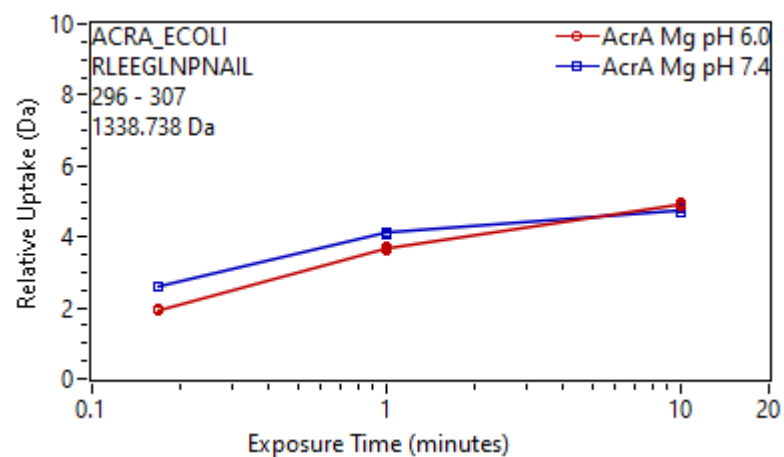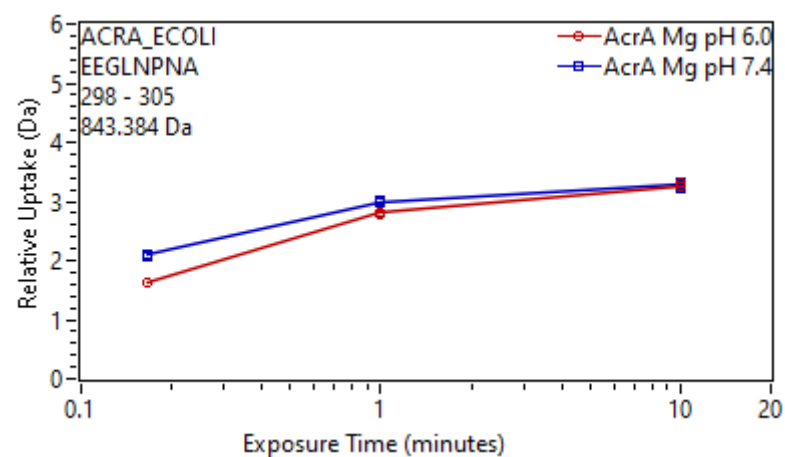

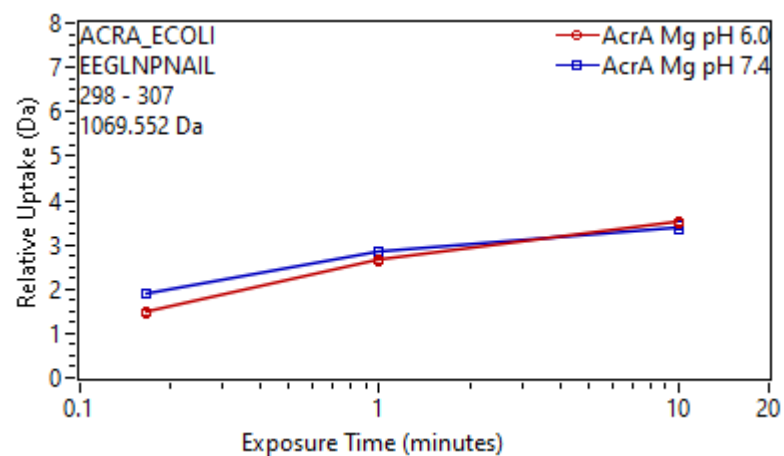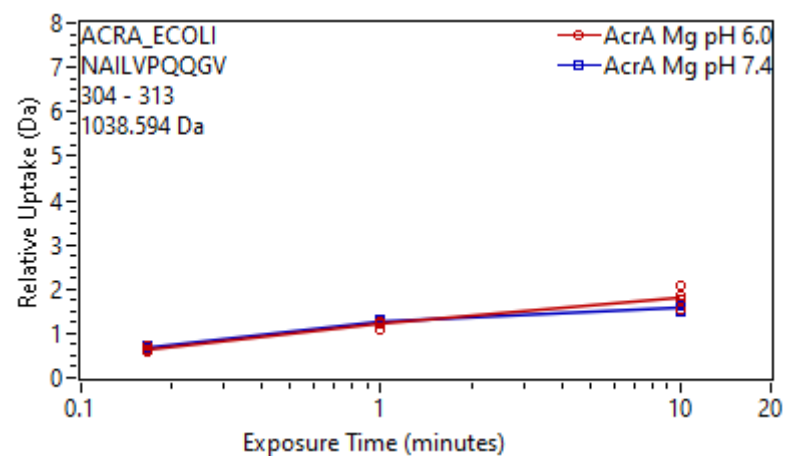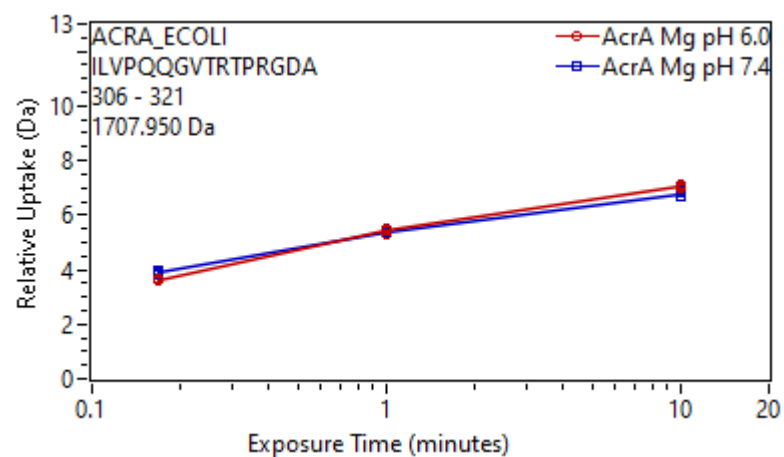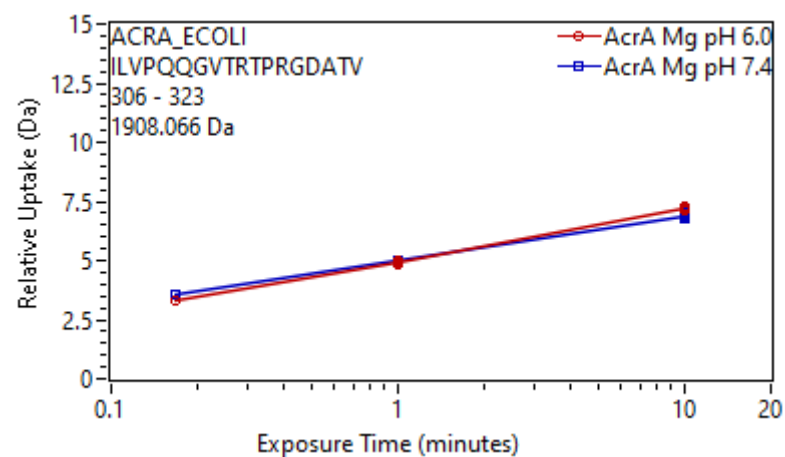

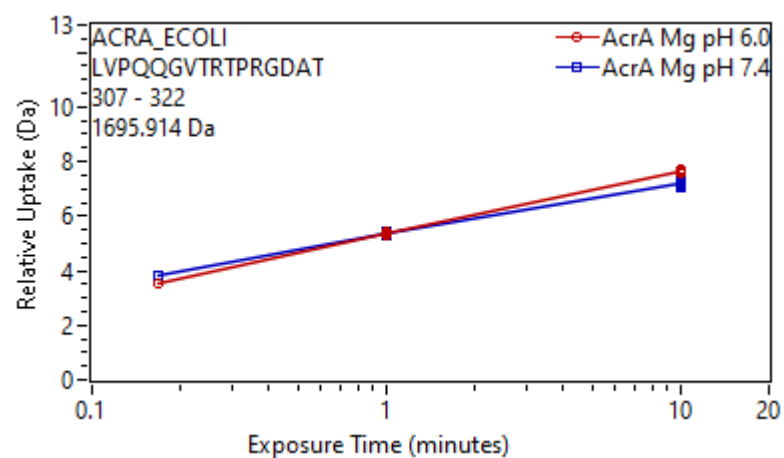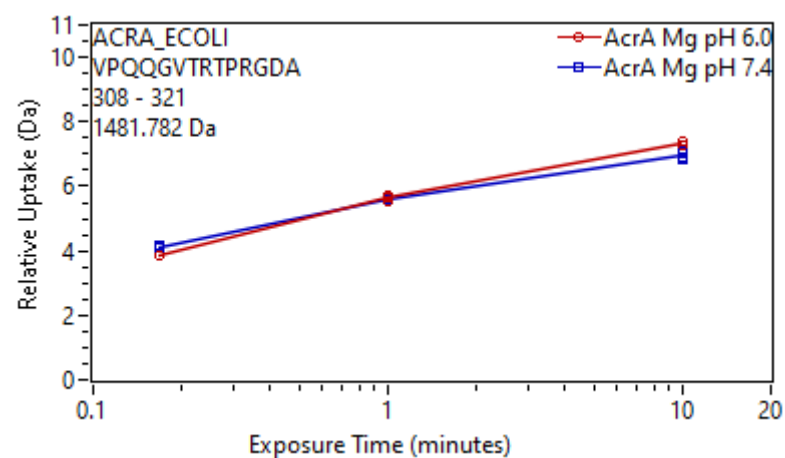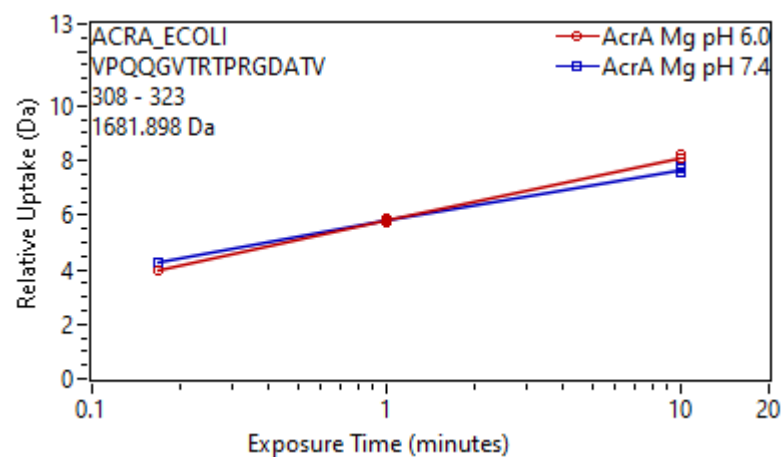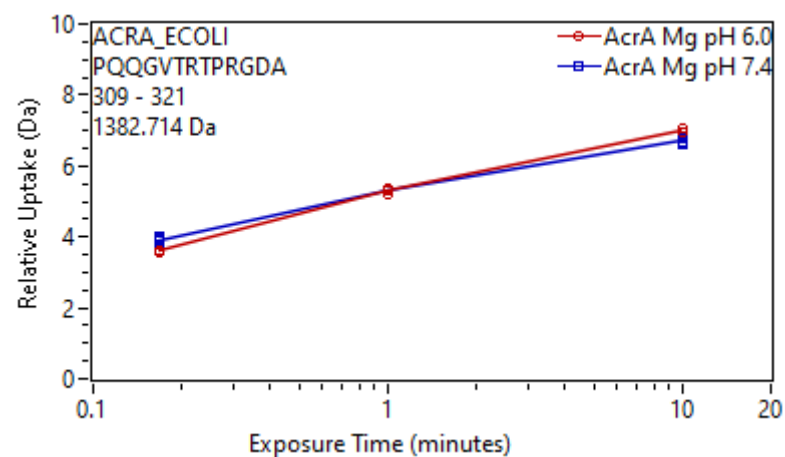

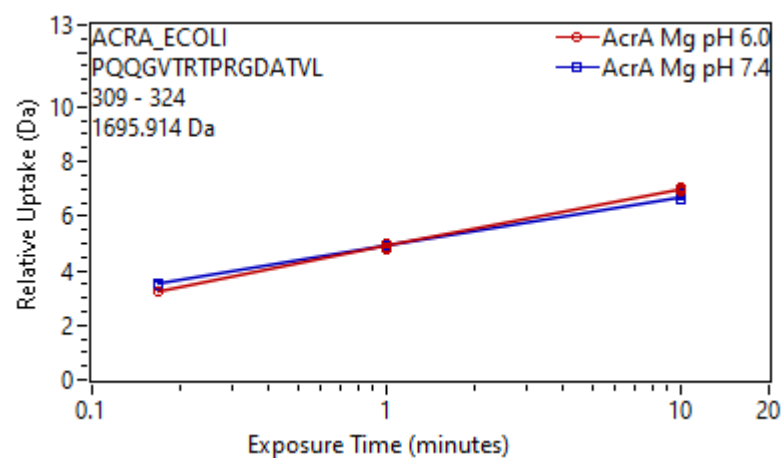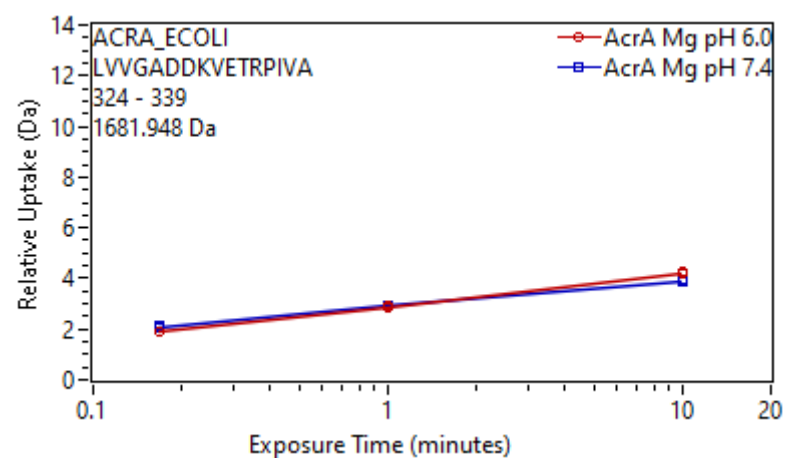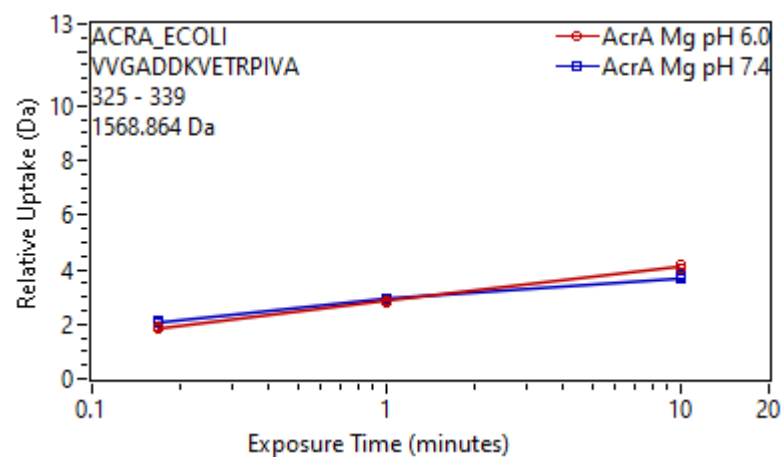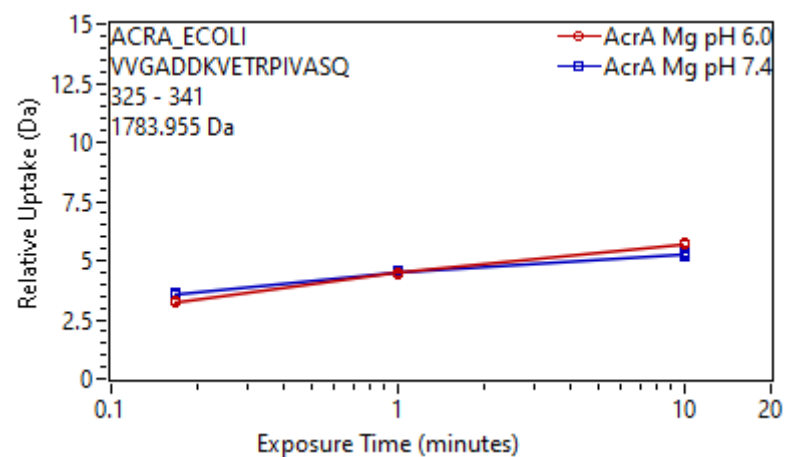

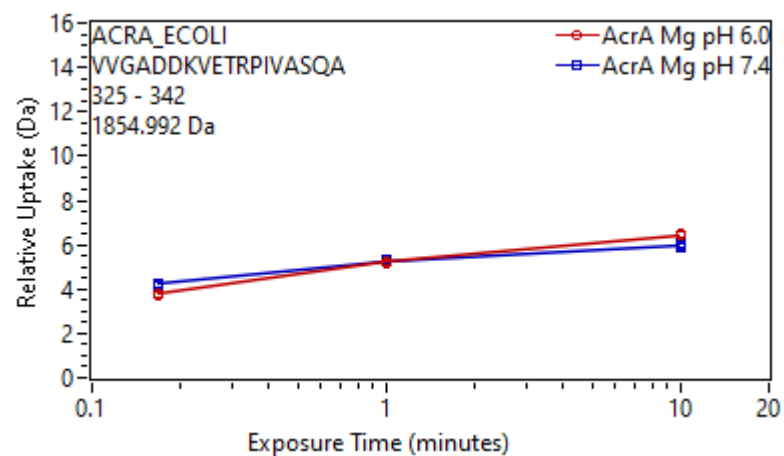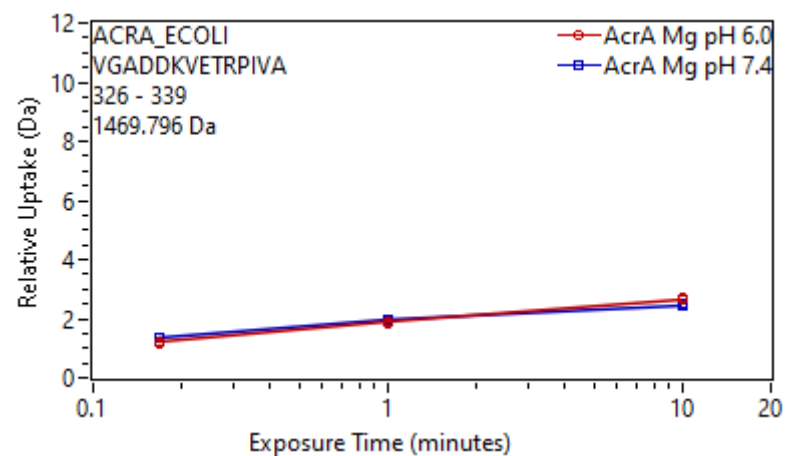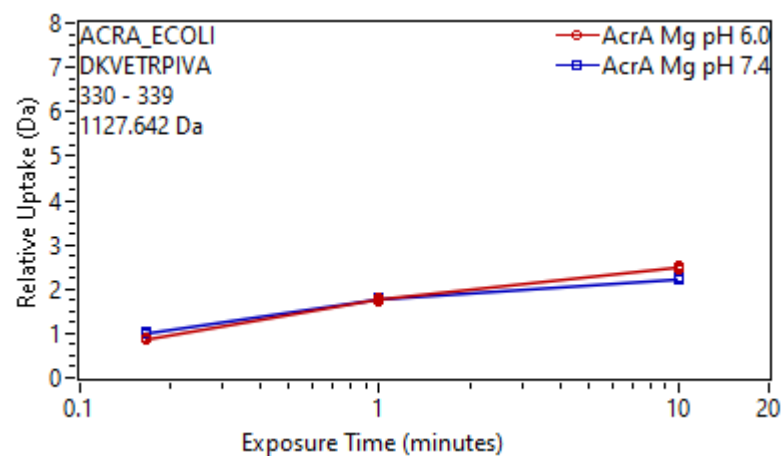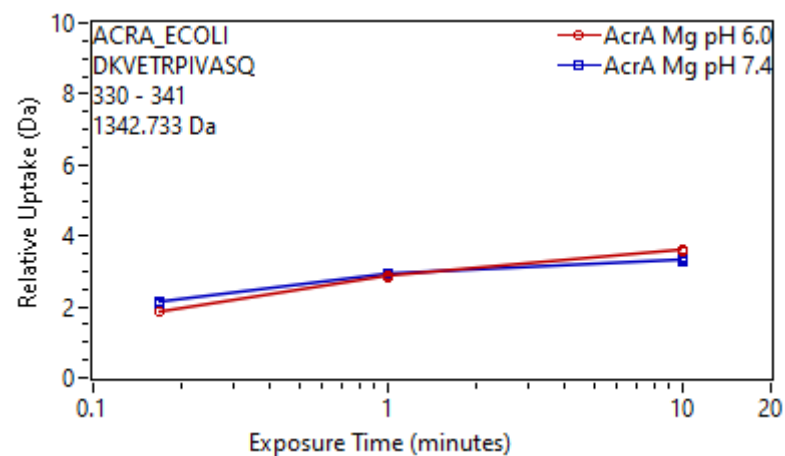

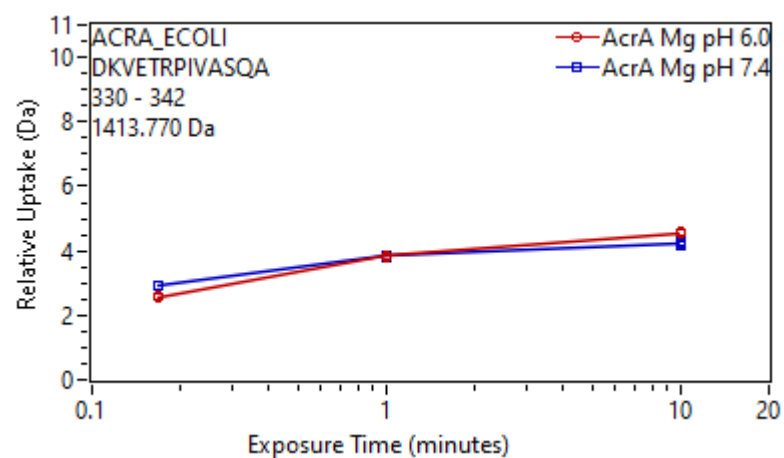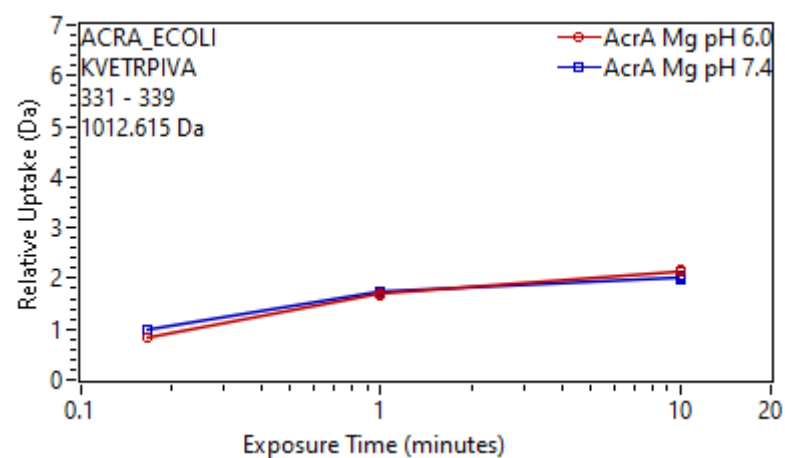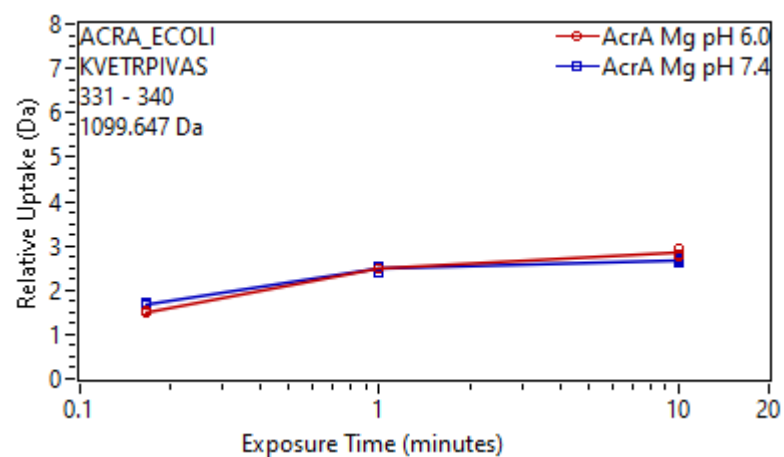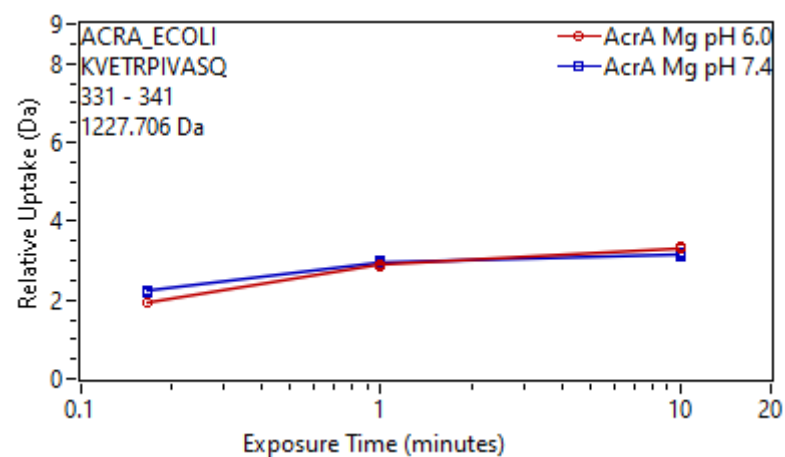

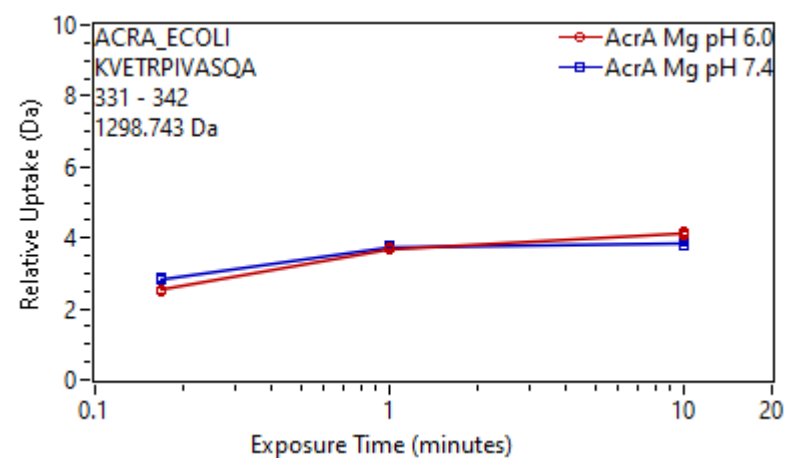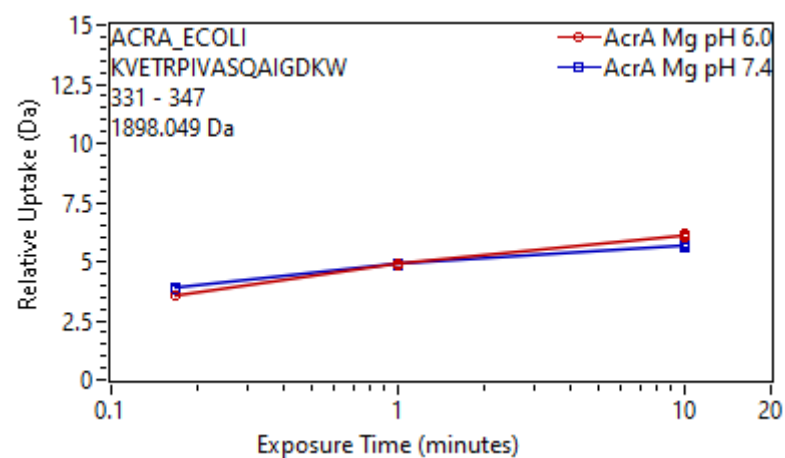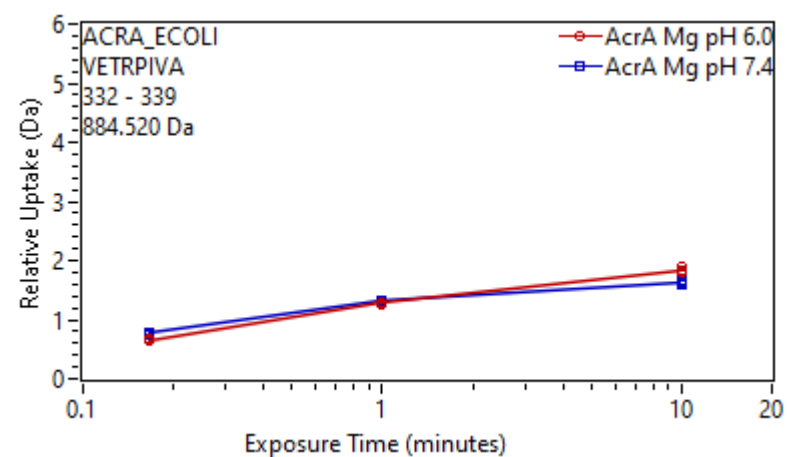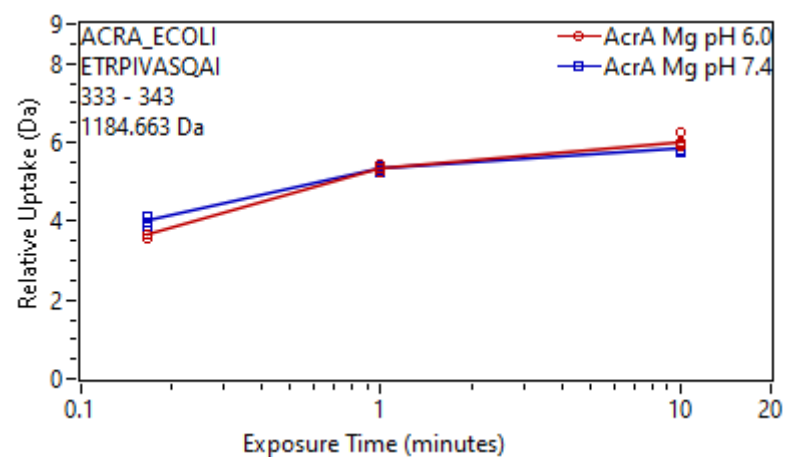

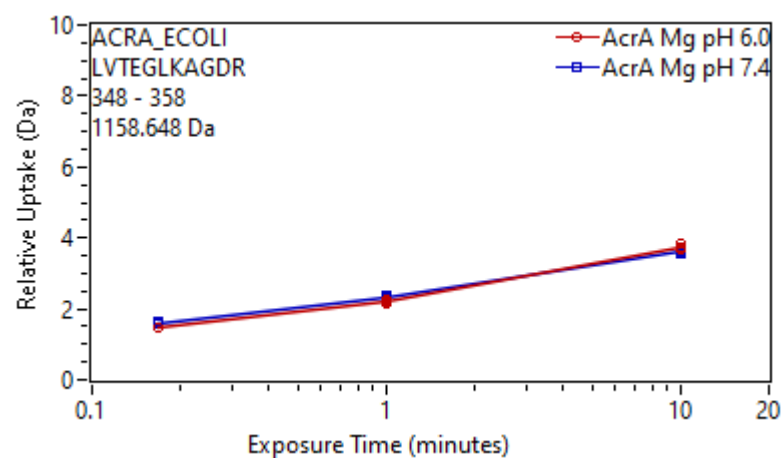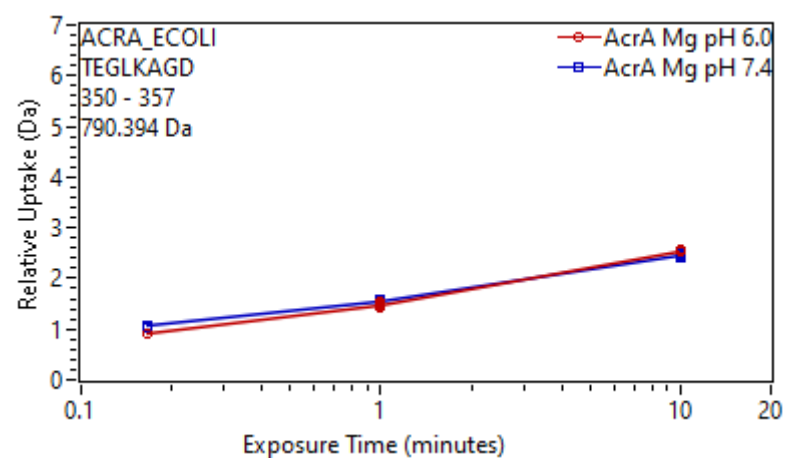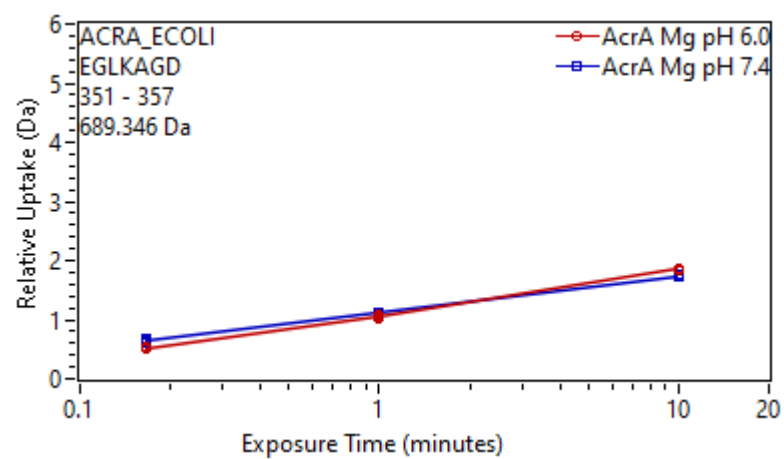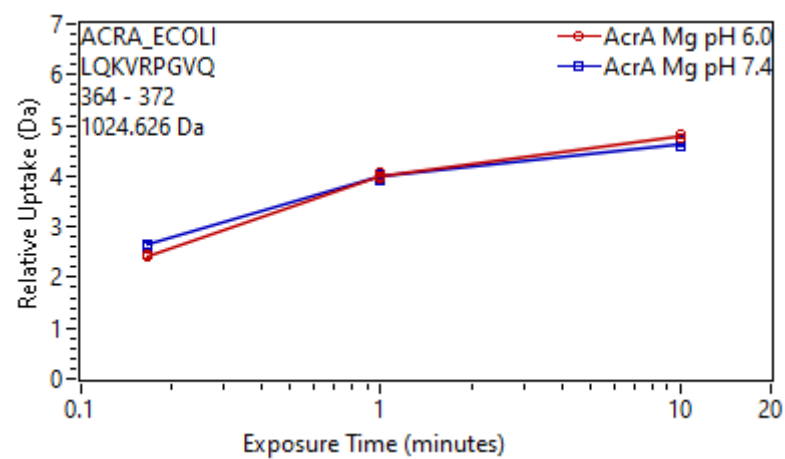

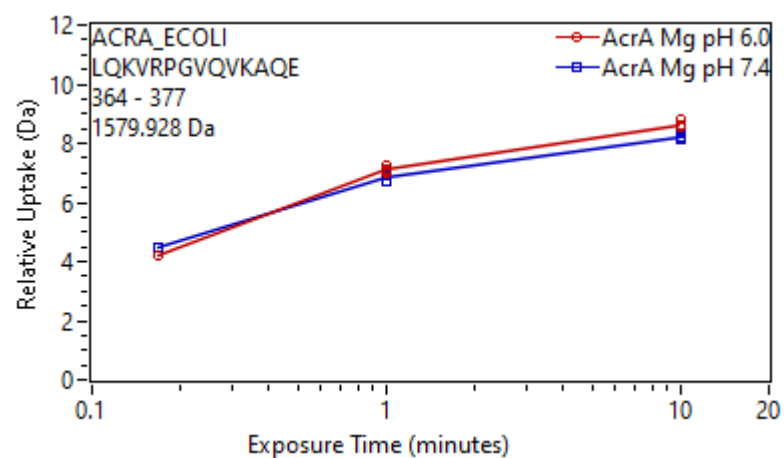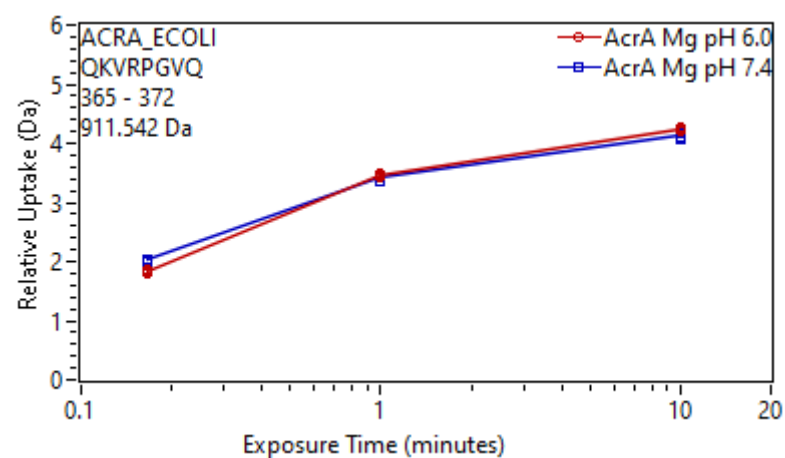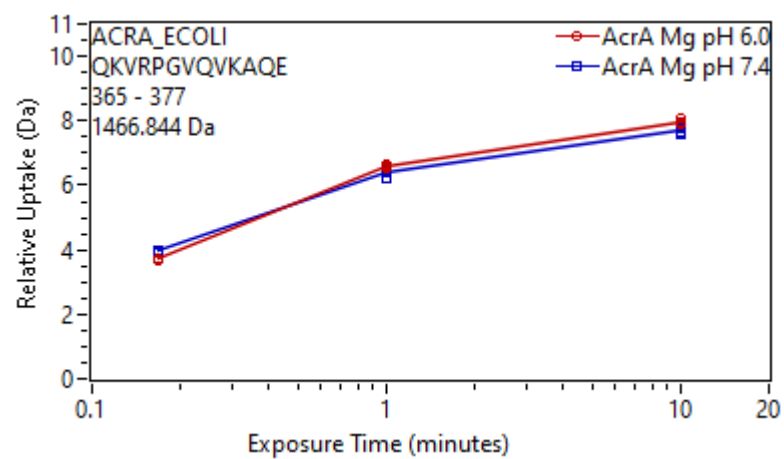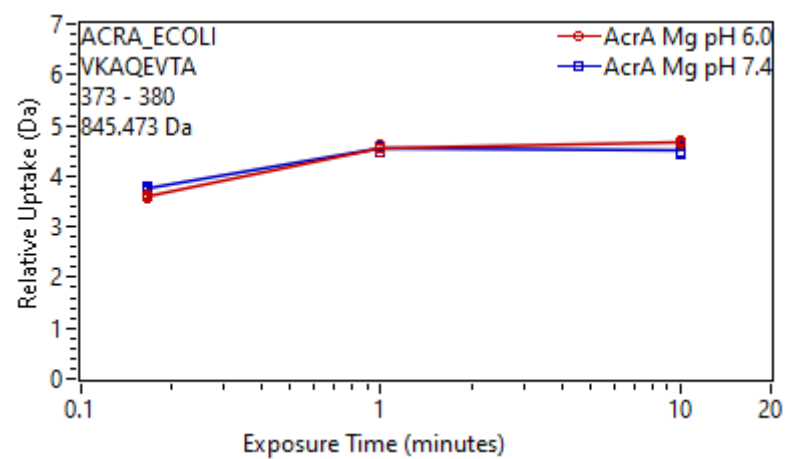

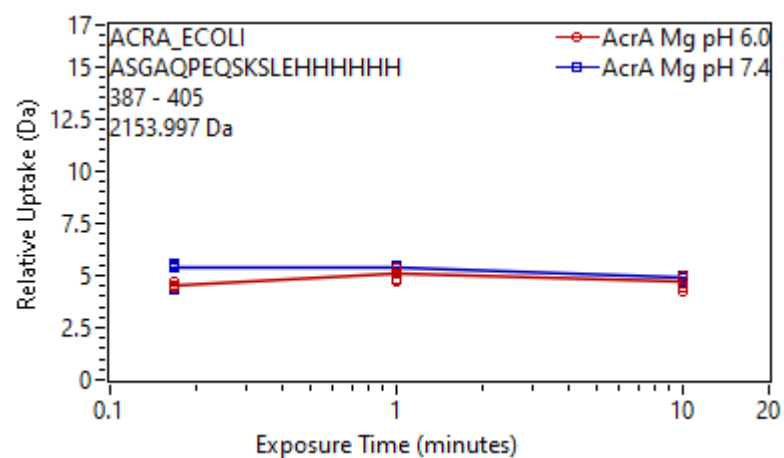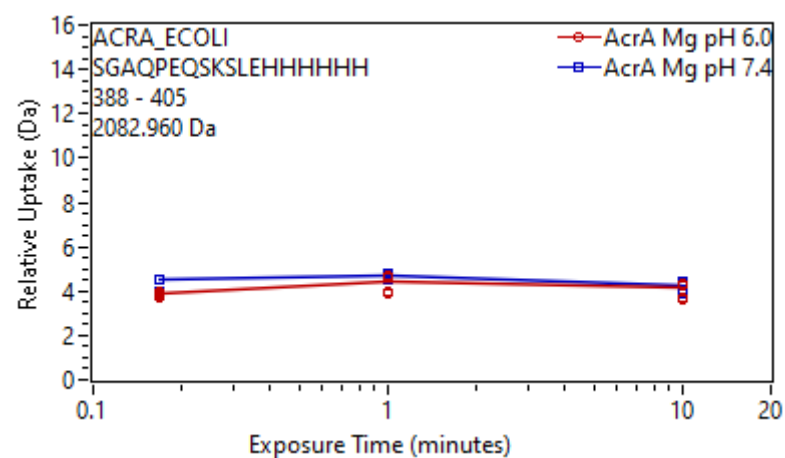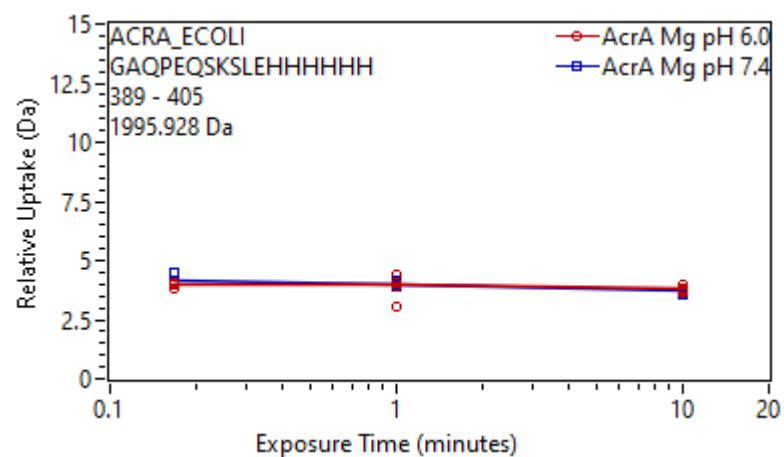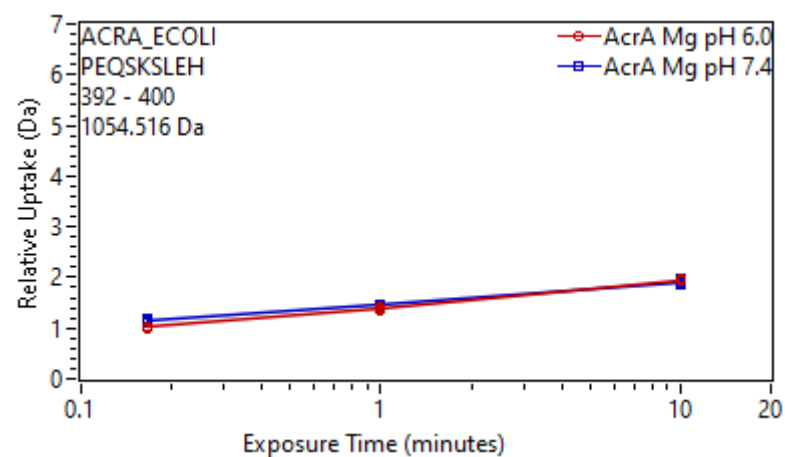

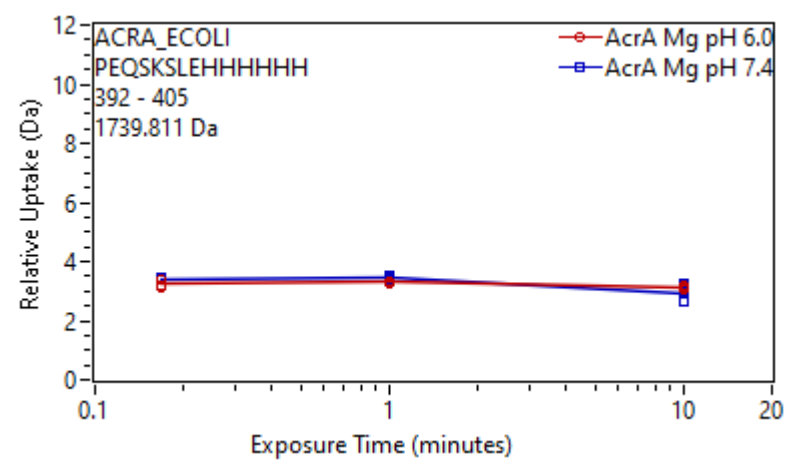

Supplement: Supplement 4 [file media-4.zip › Supplementary Data 3/Uptake plots 4.pdf]

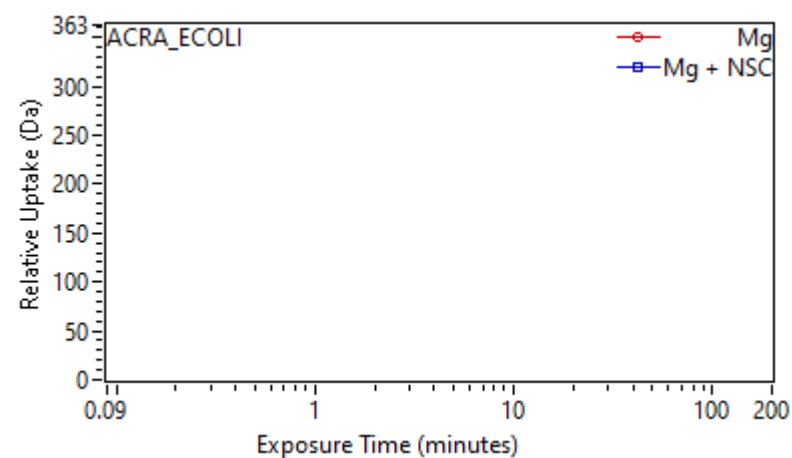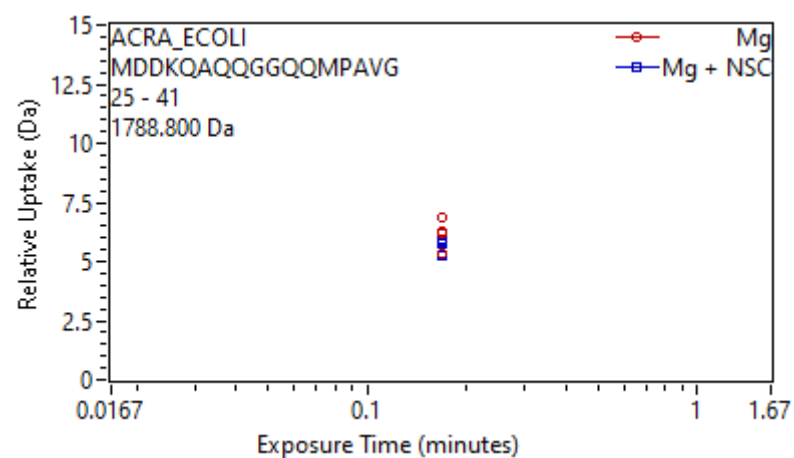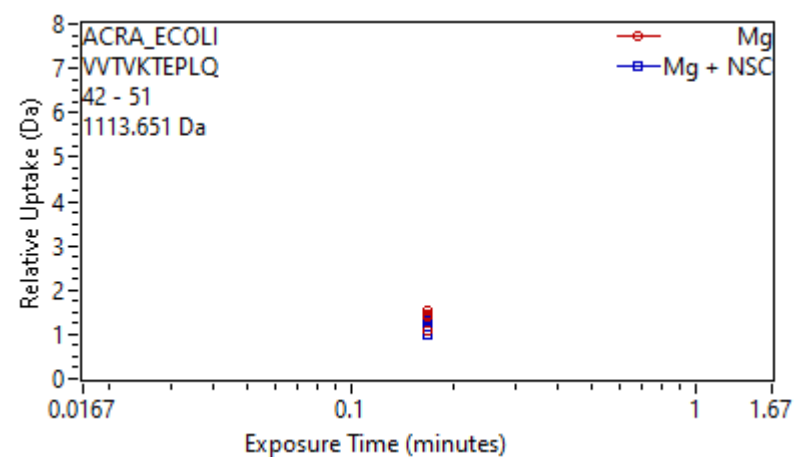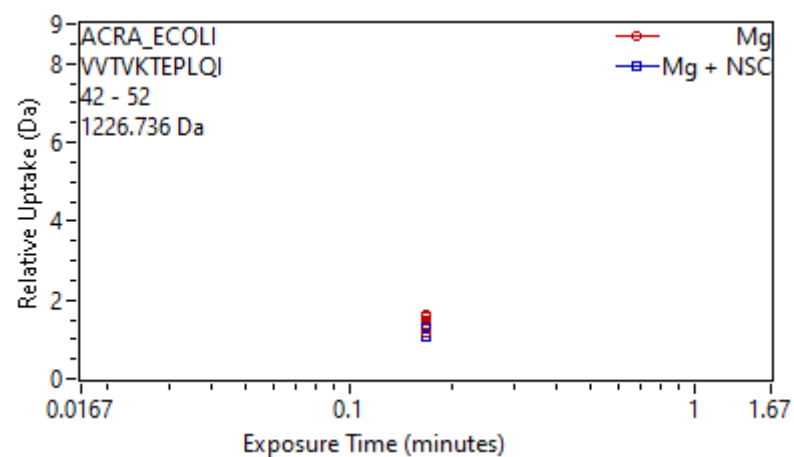

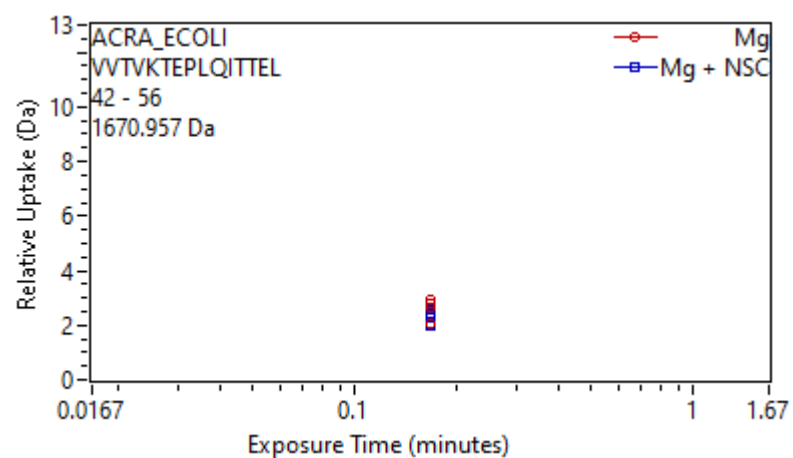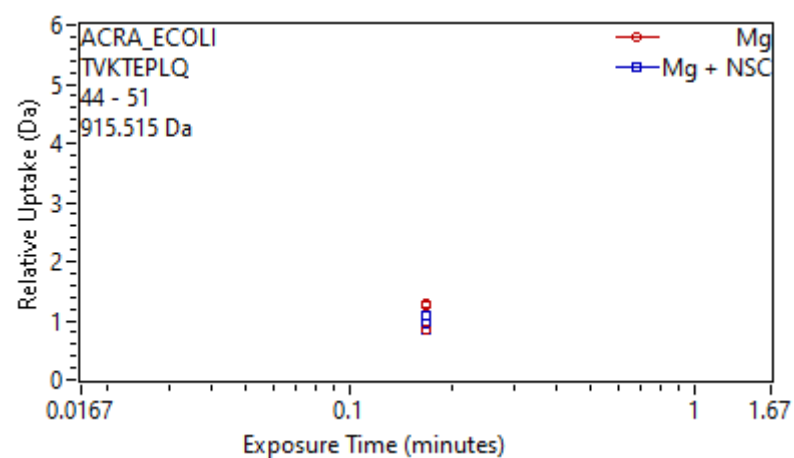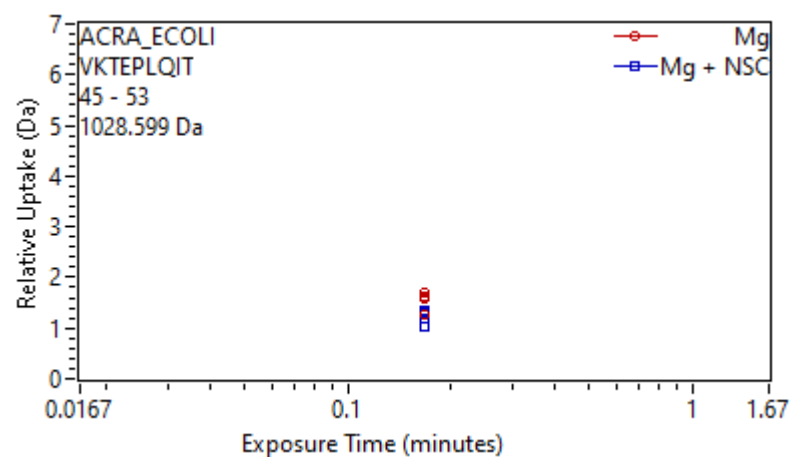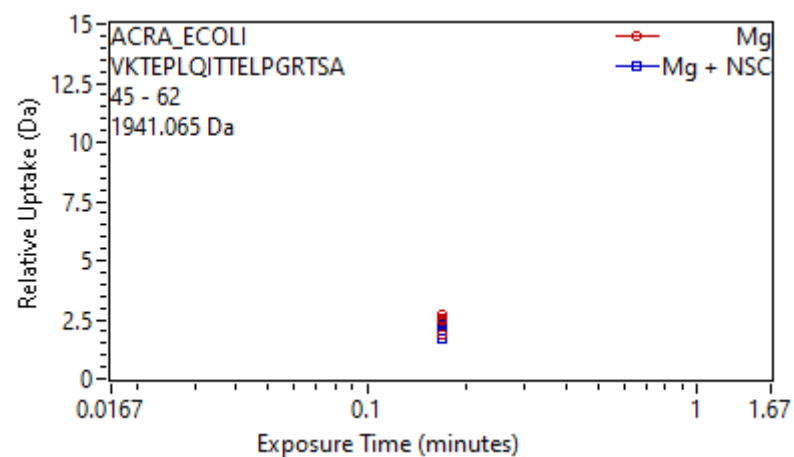

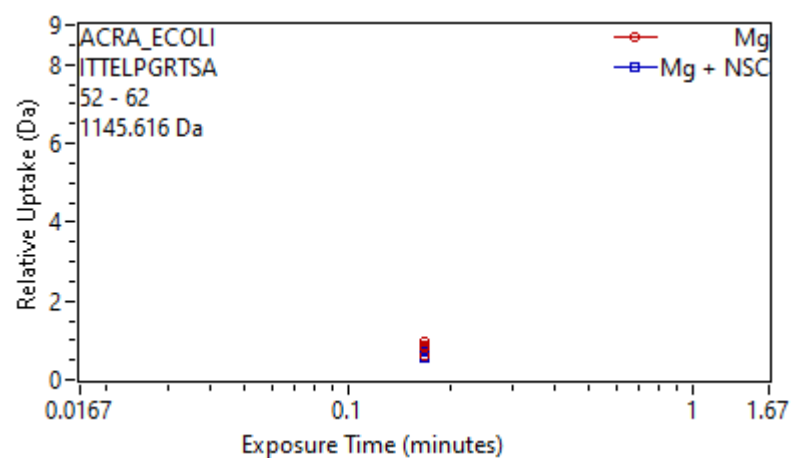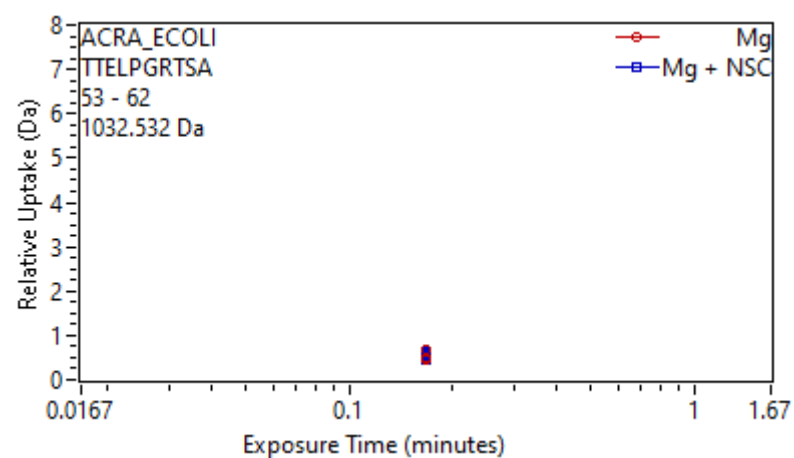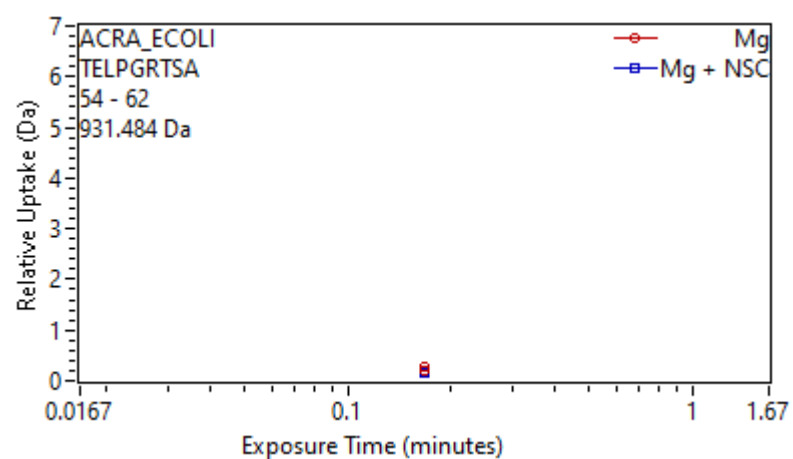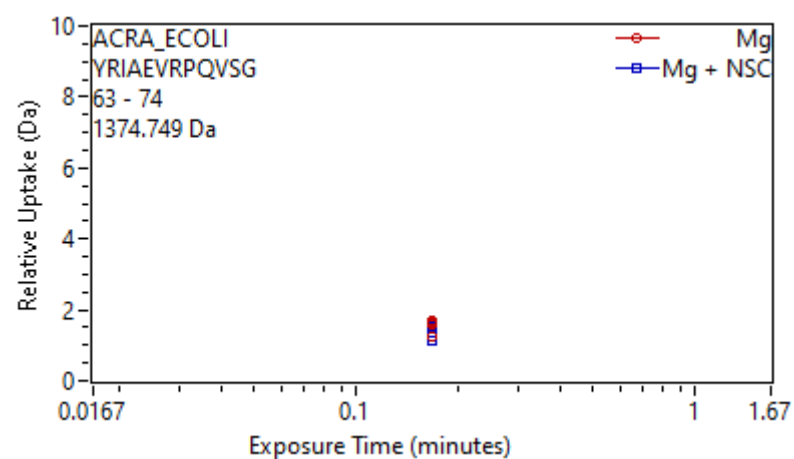

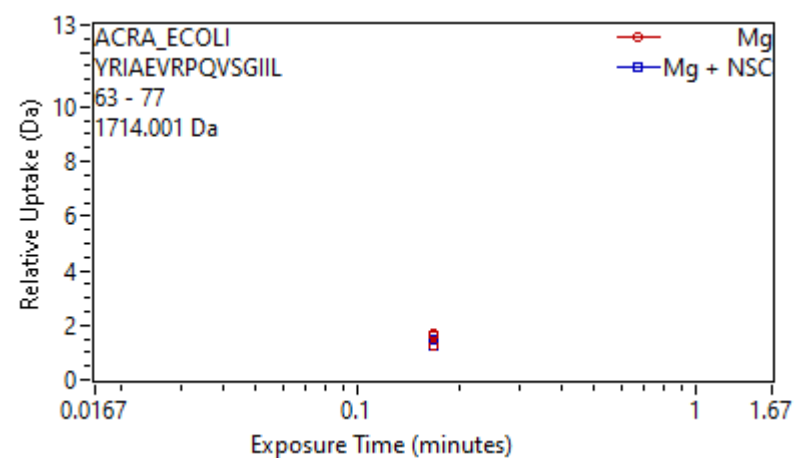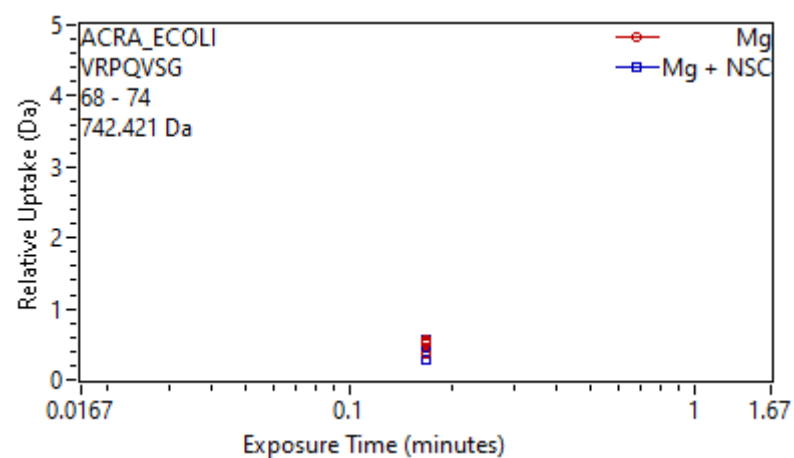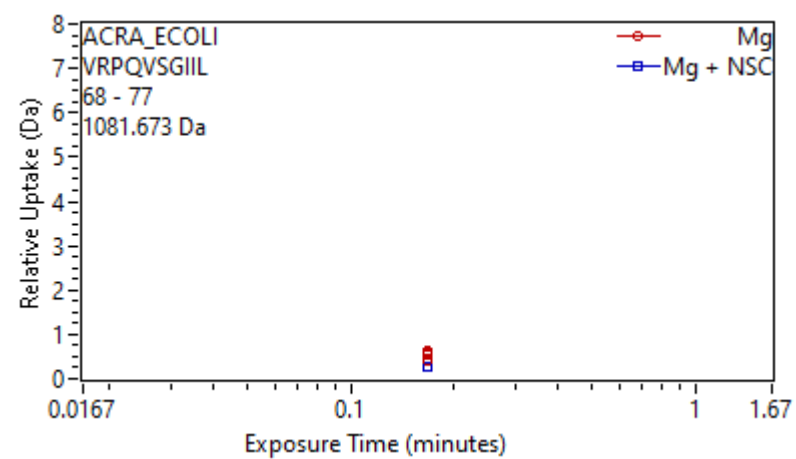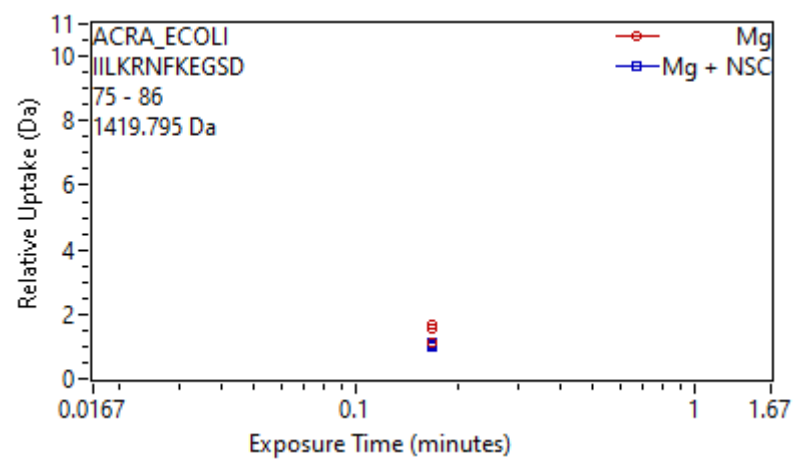

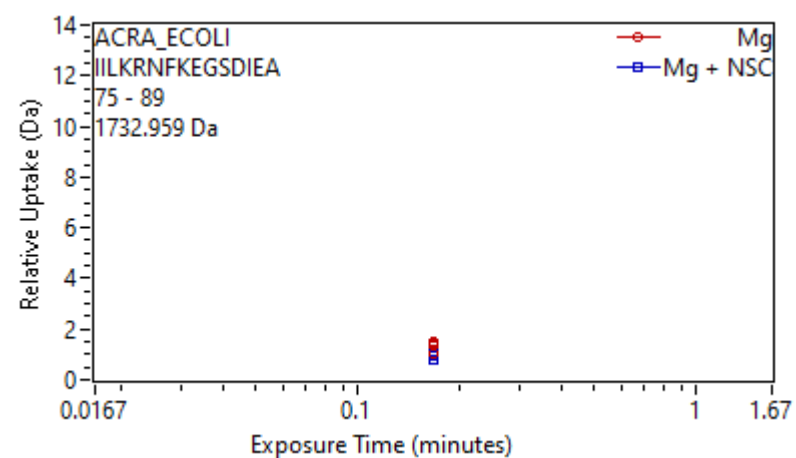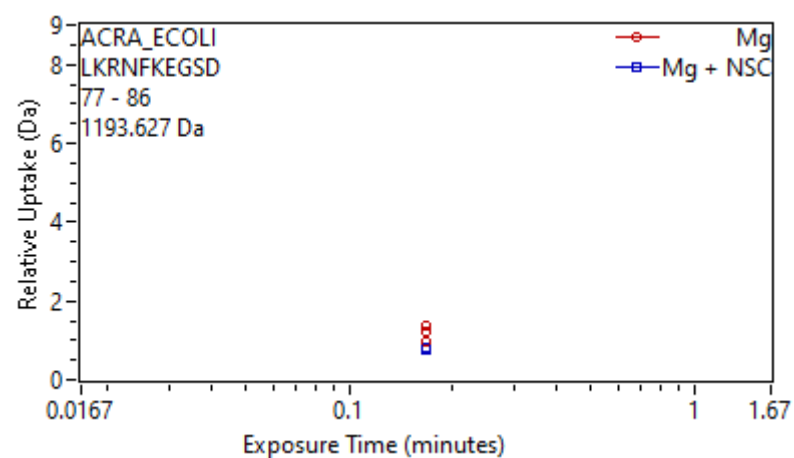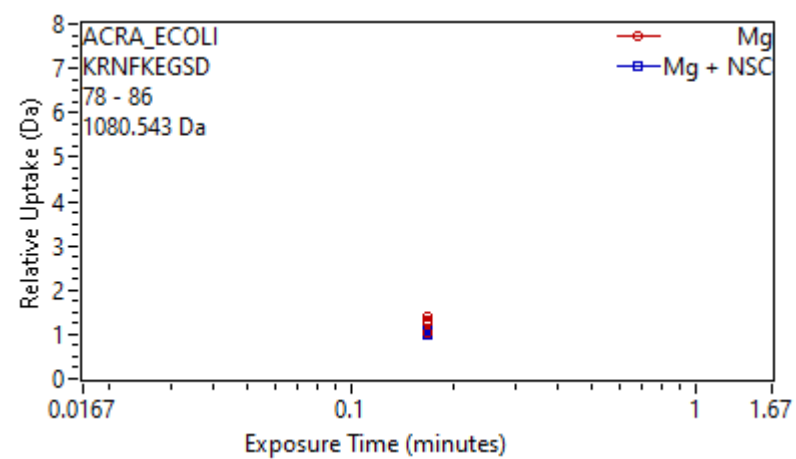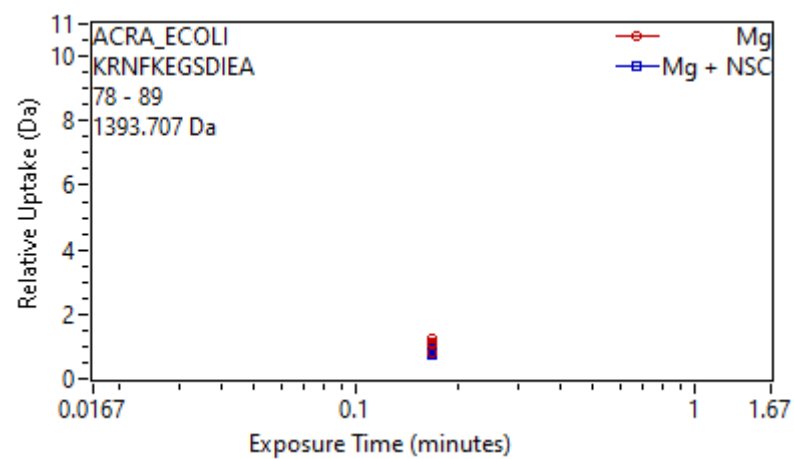

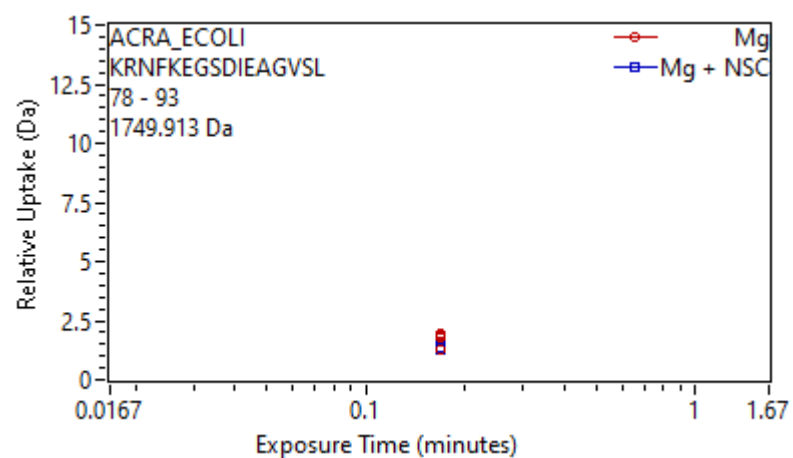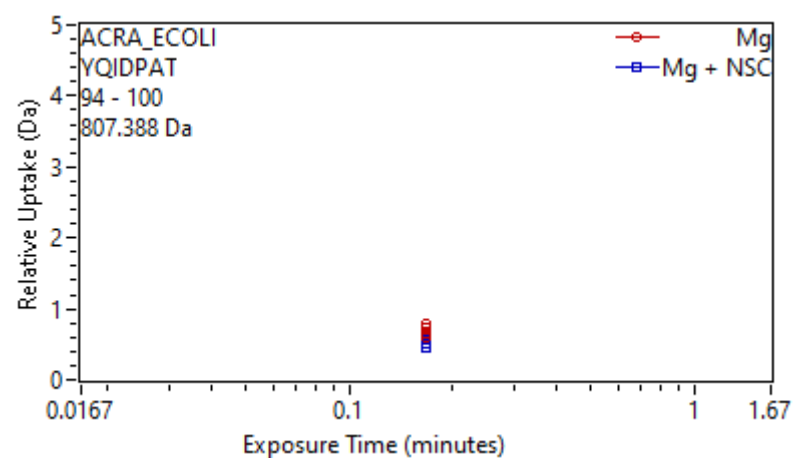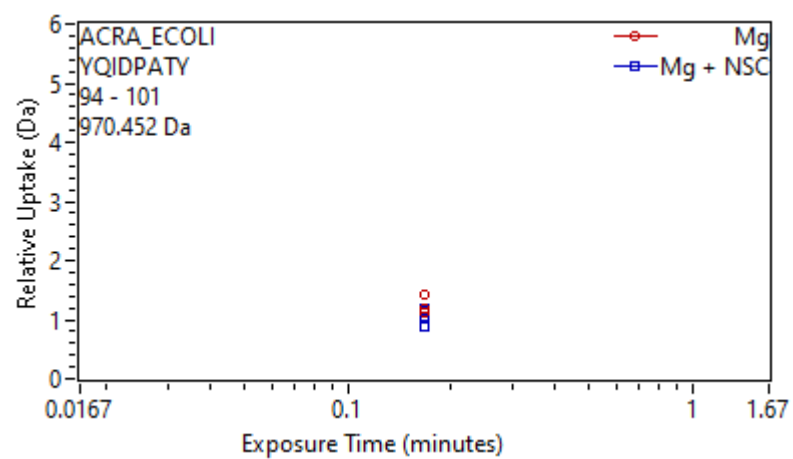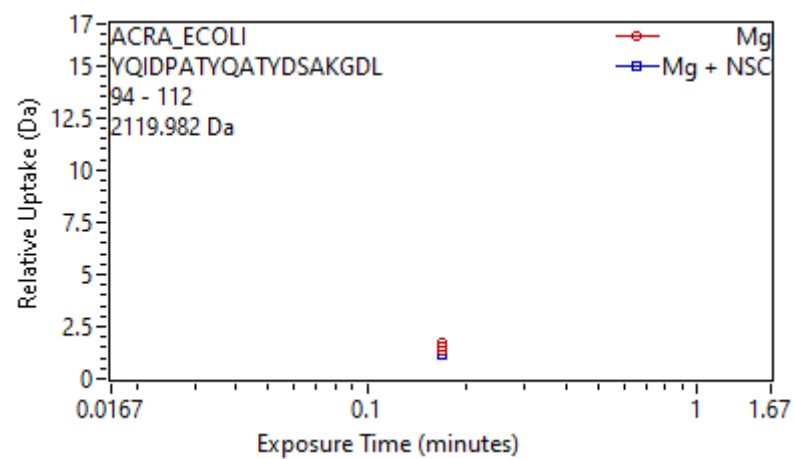

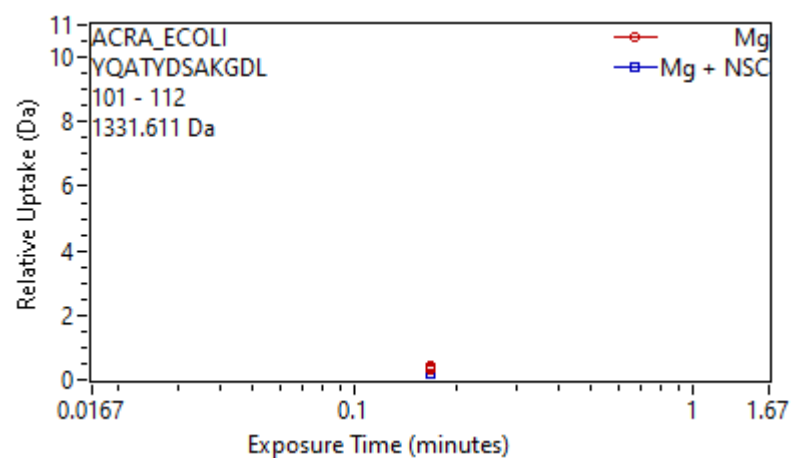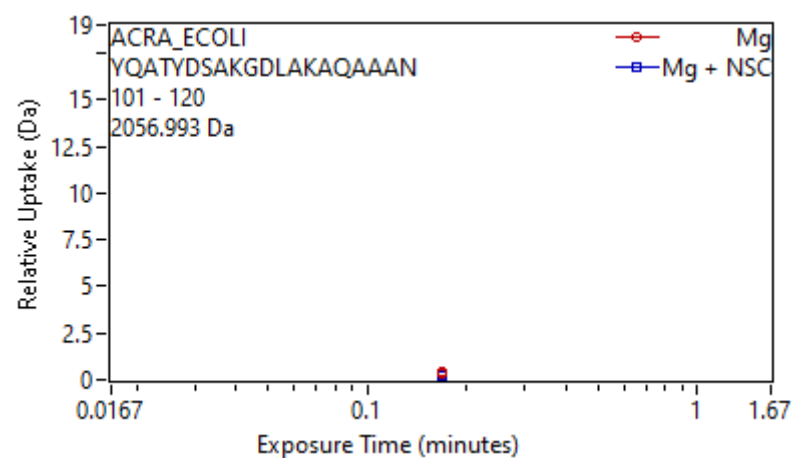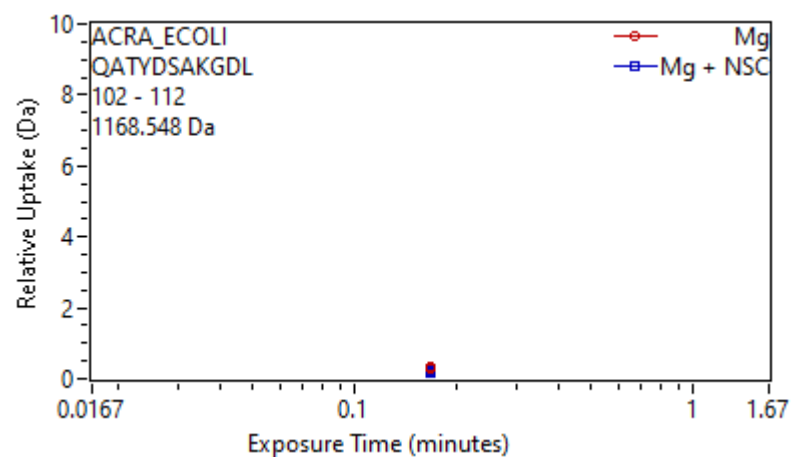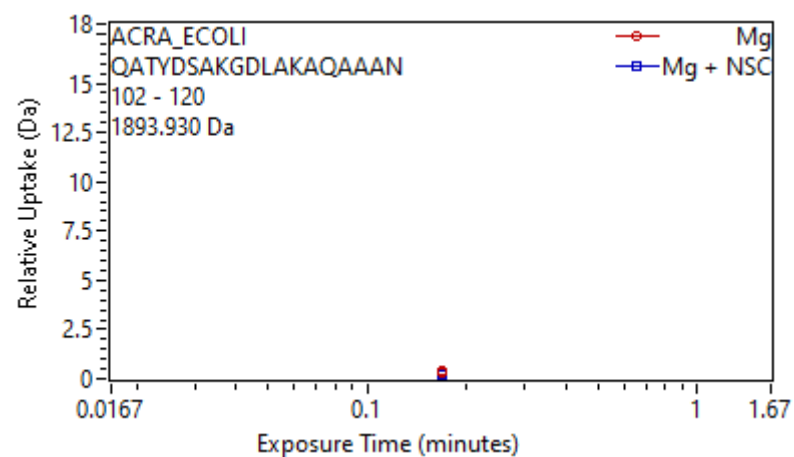

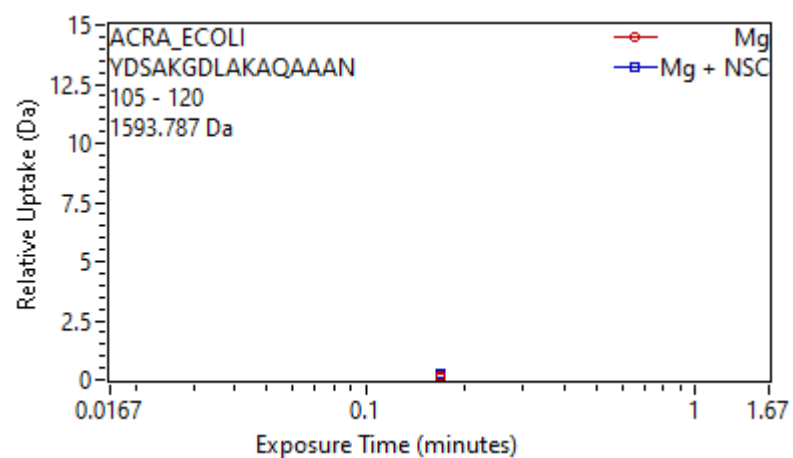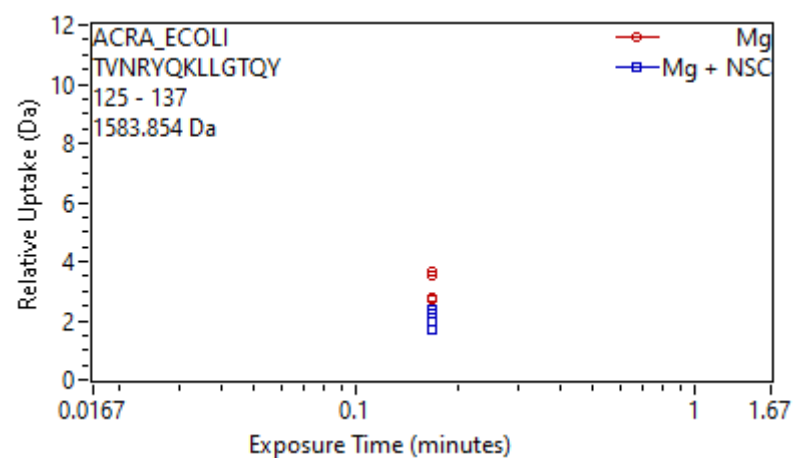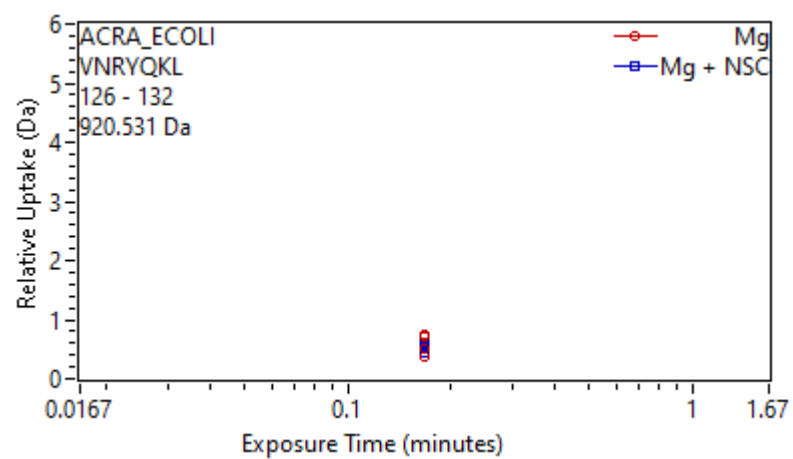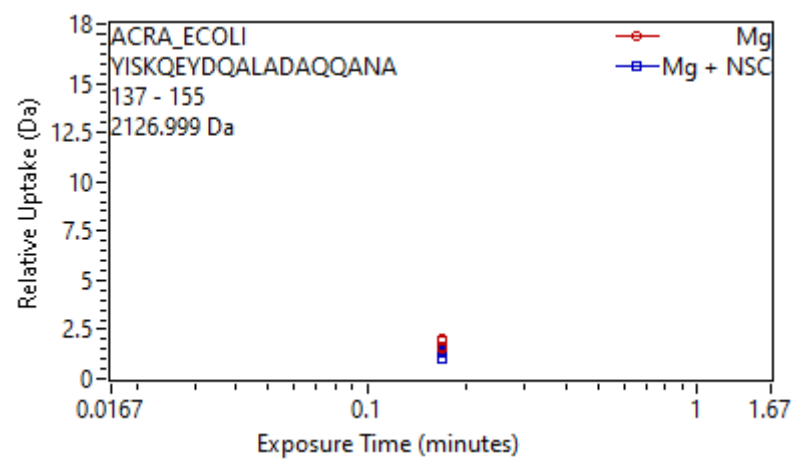

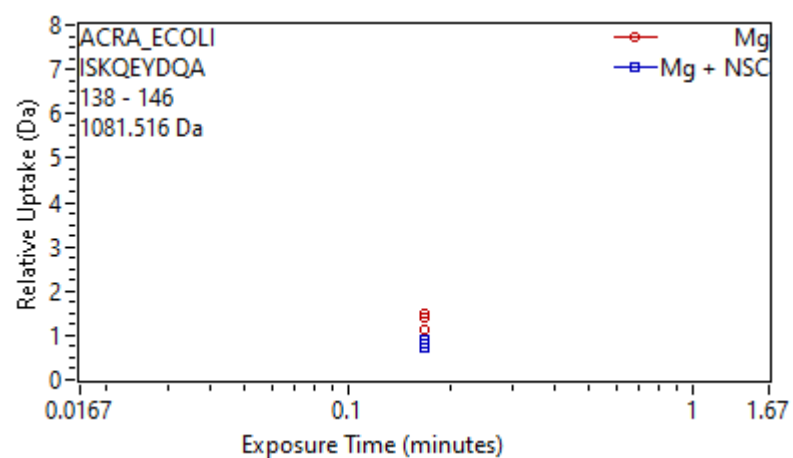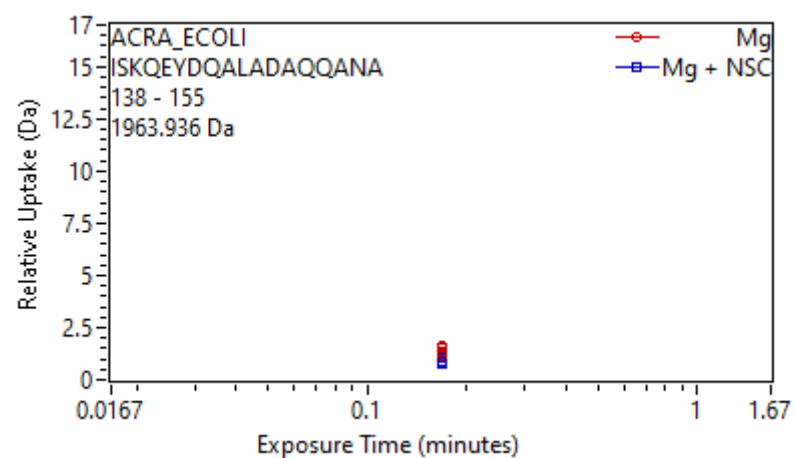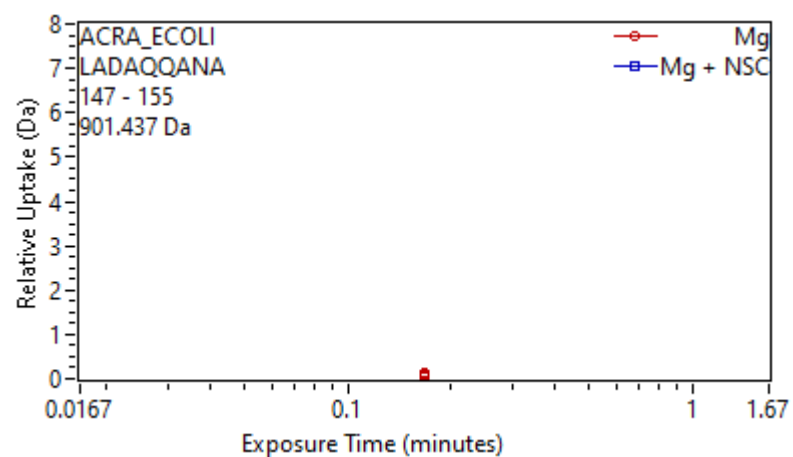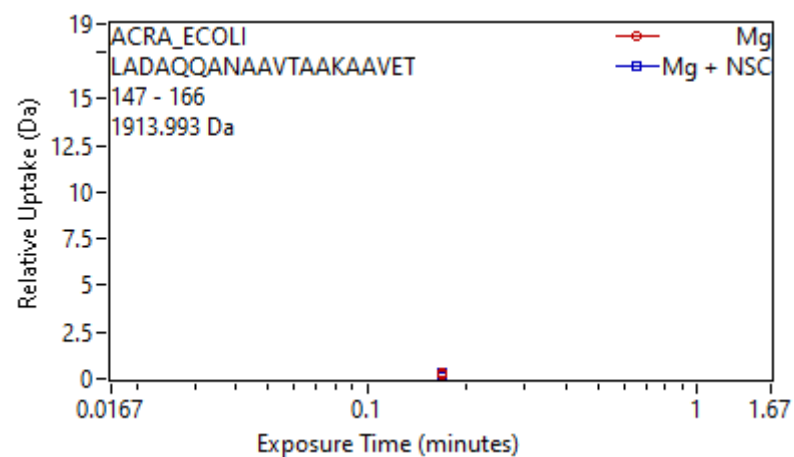

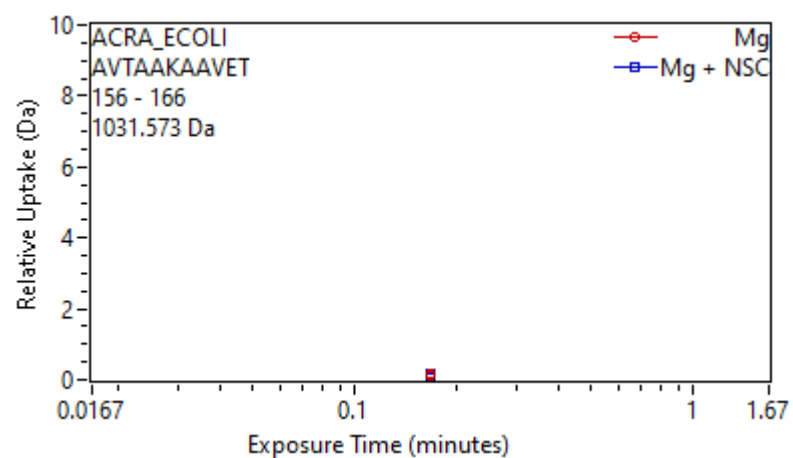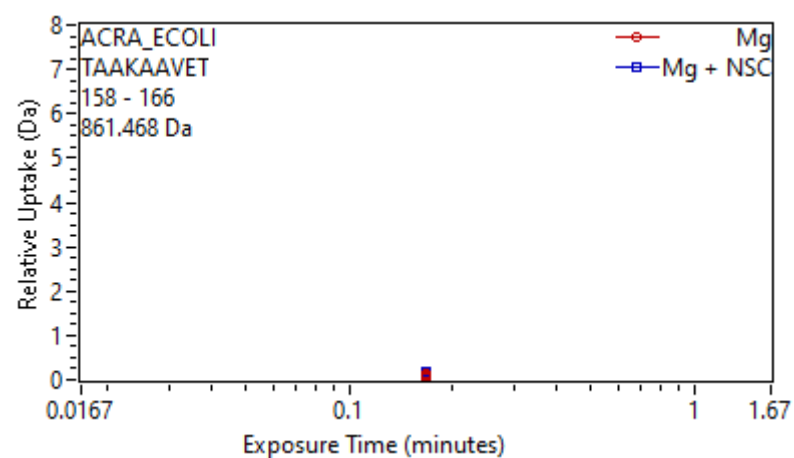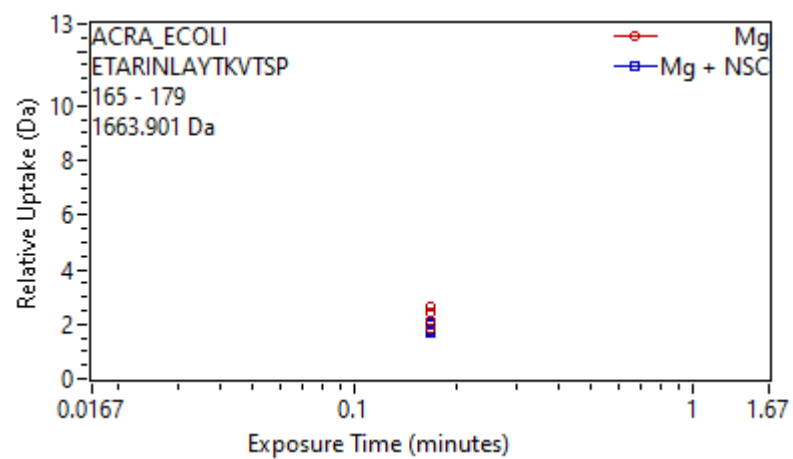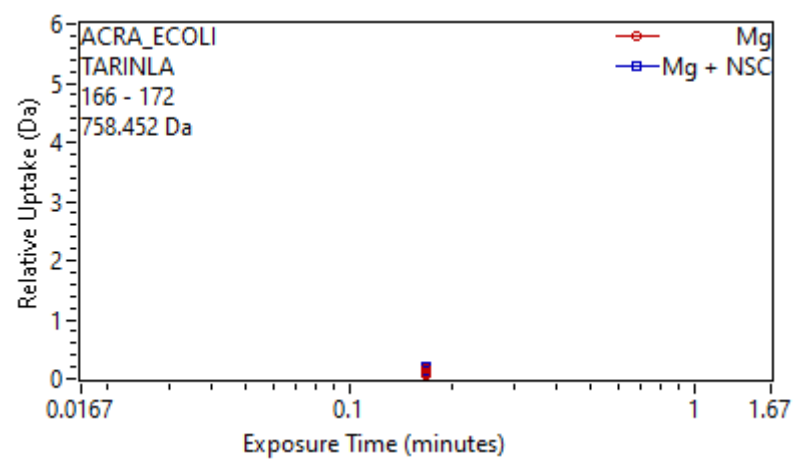

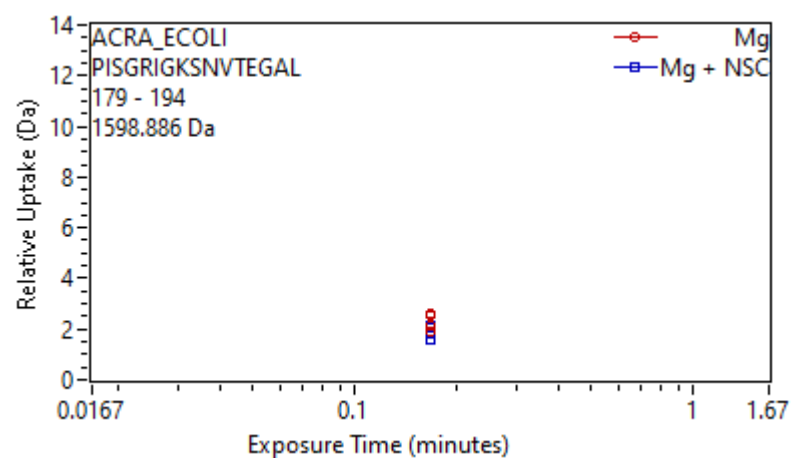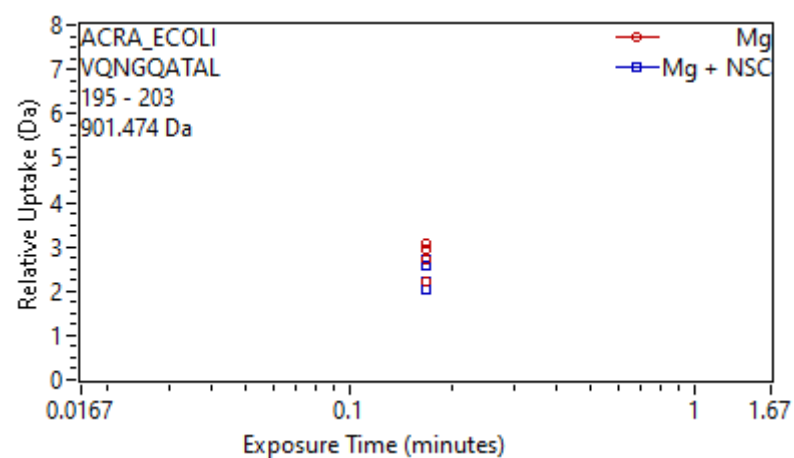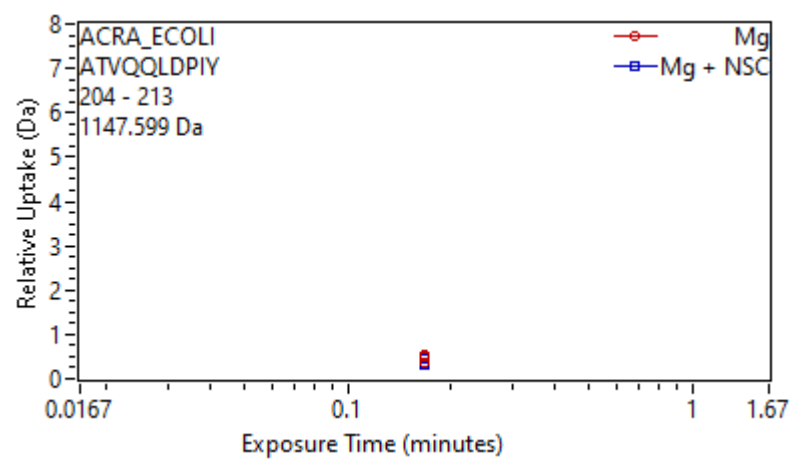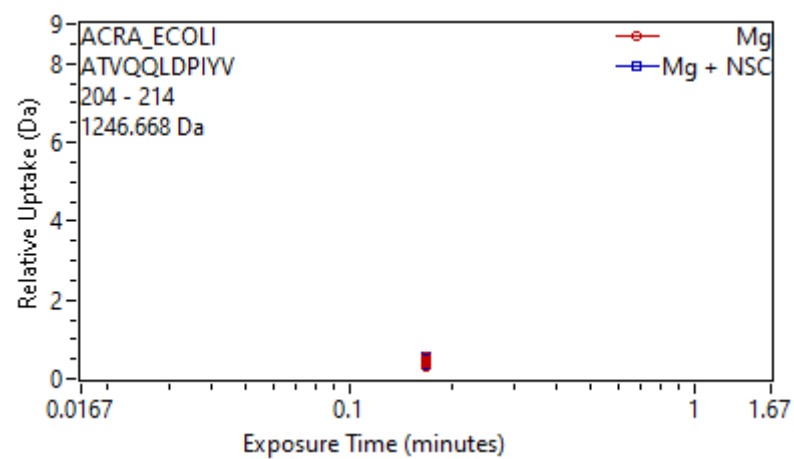

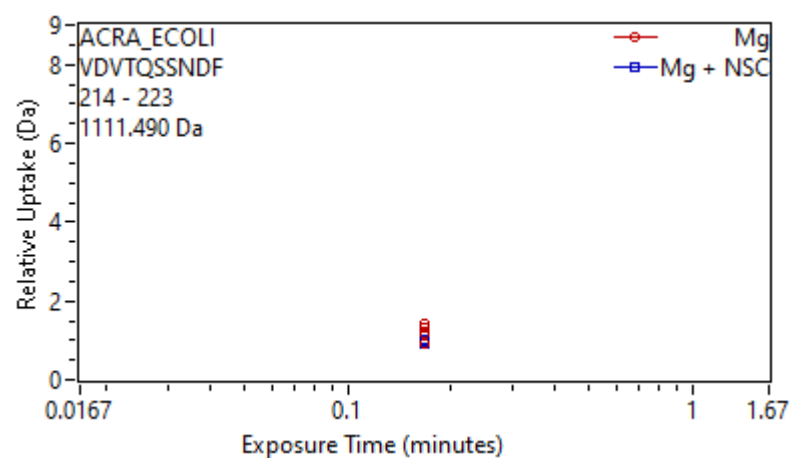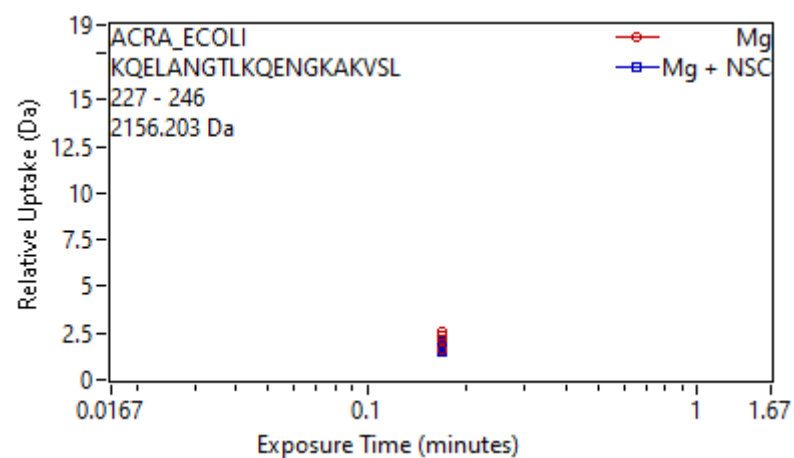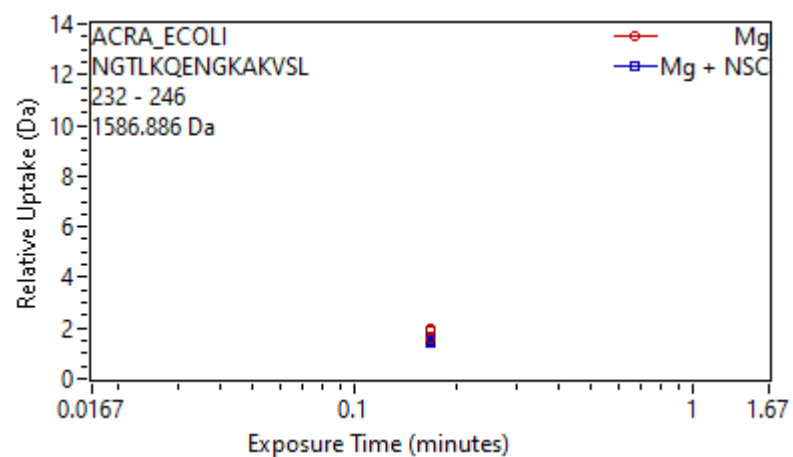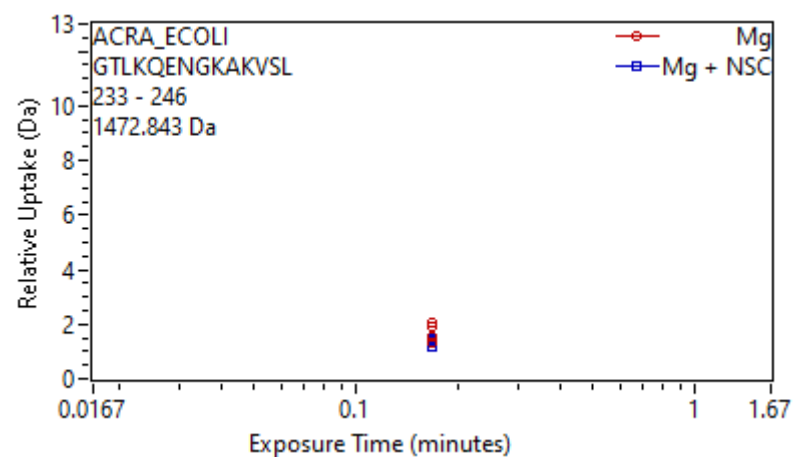

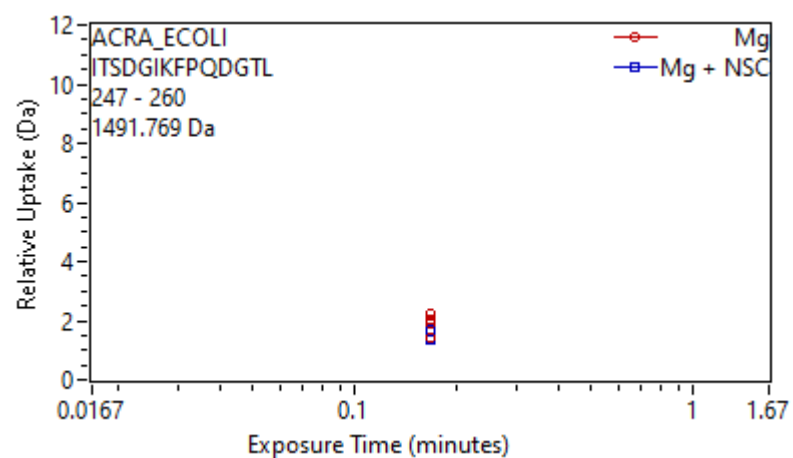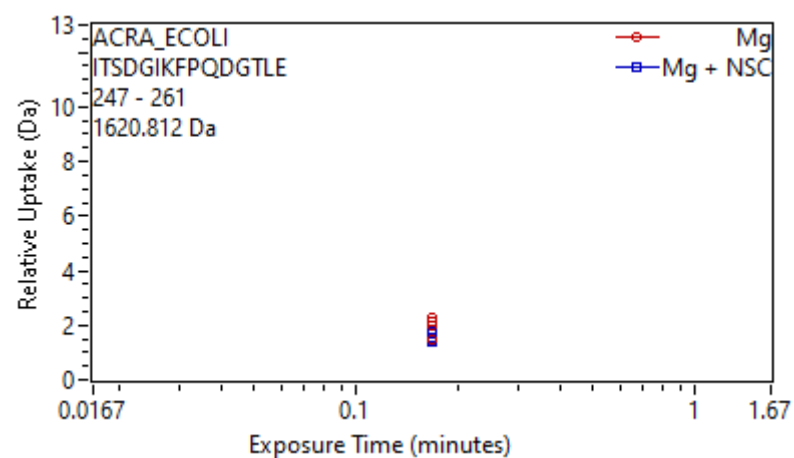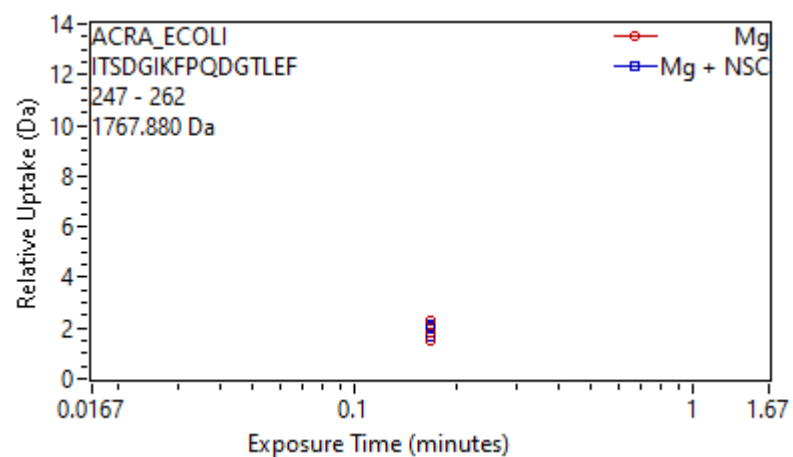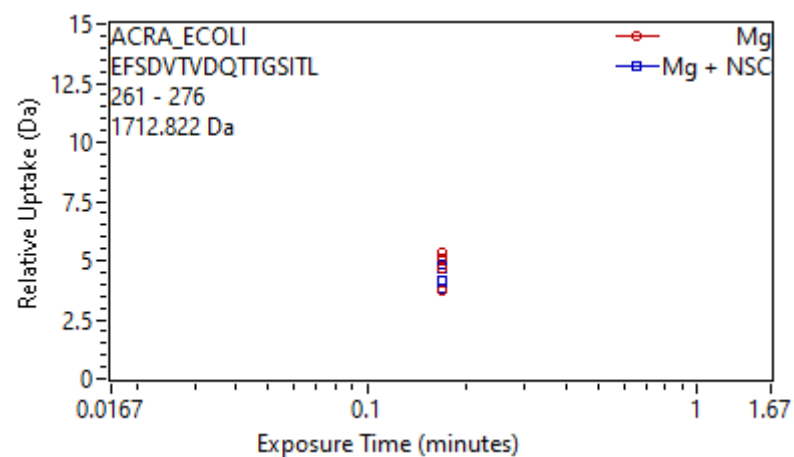

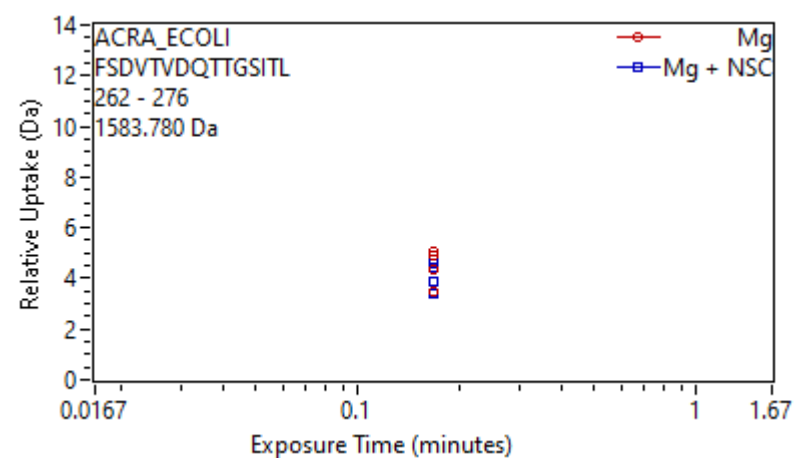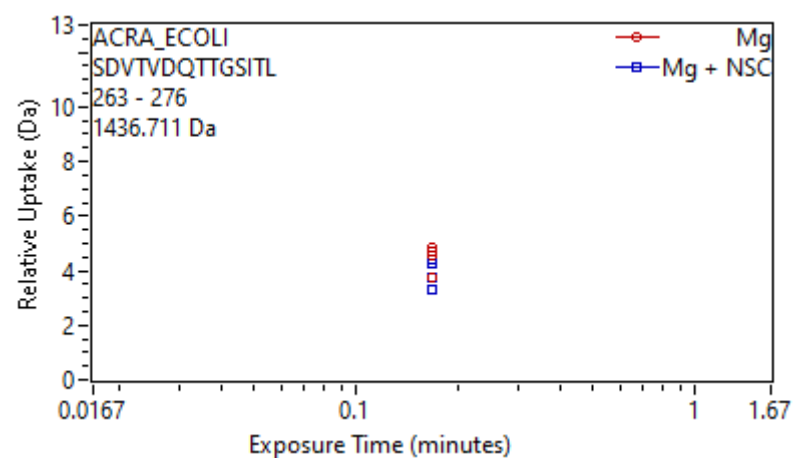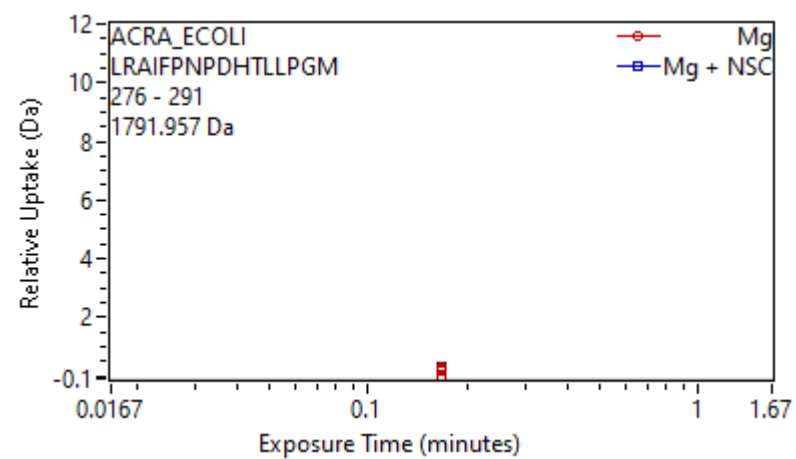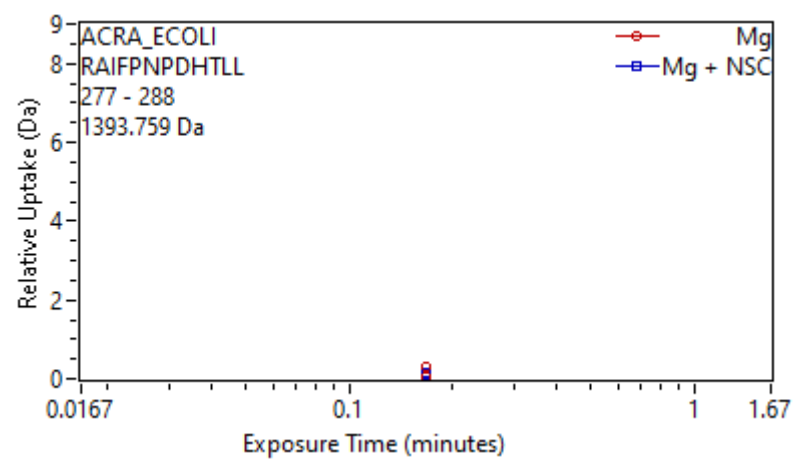

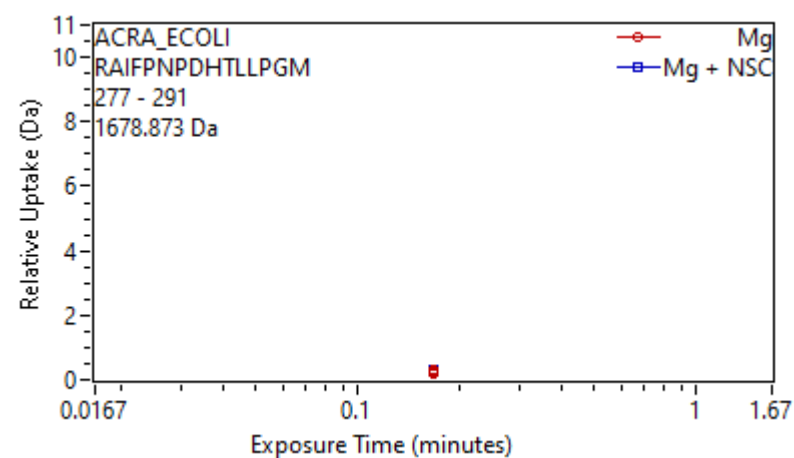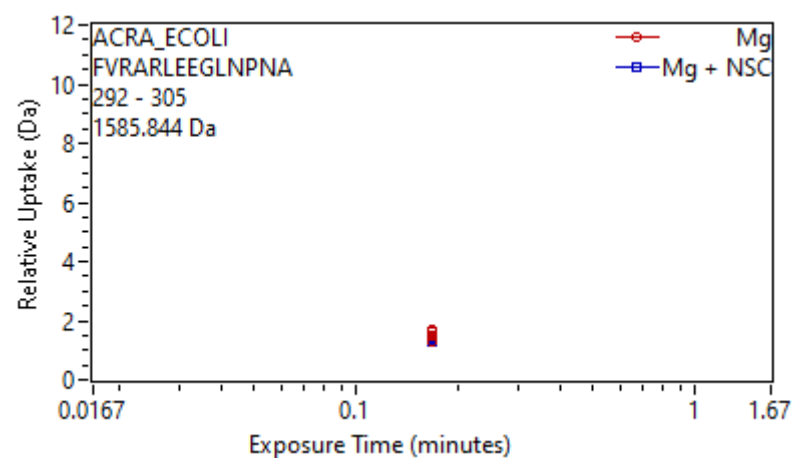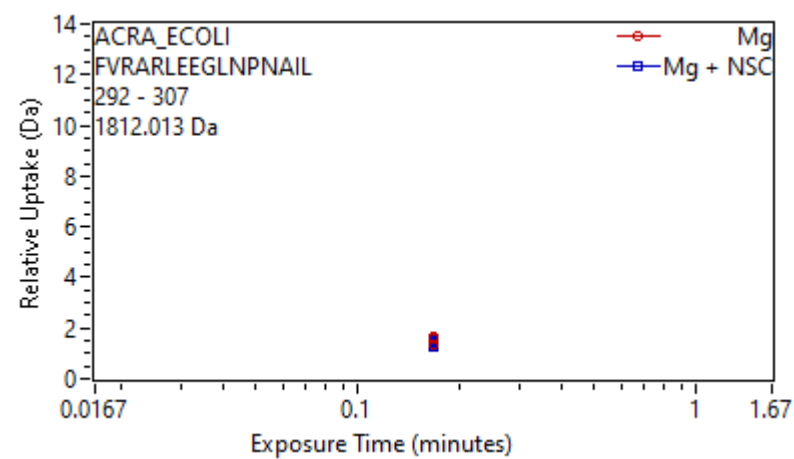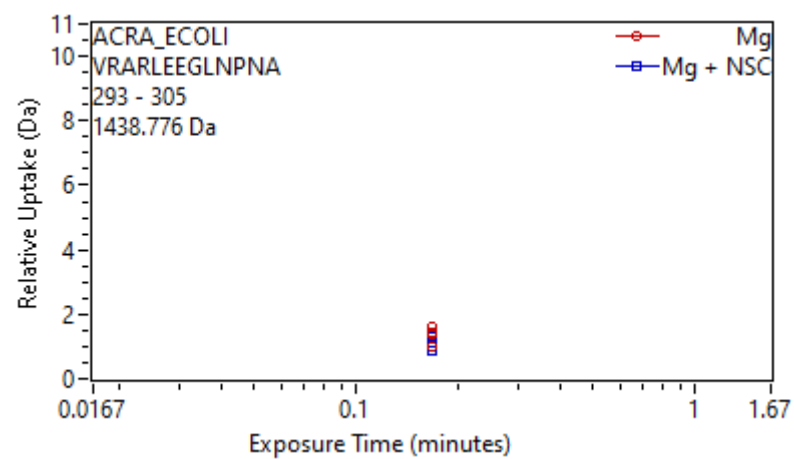

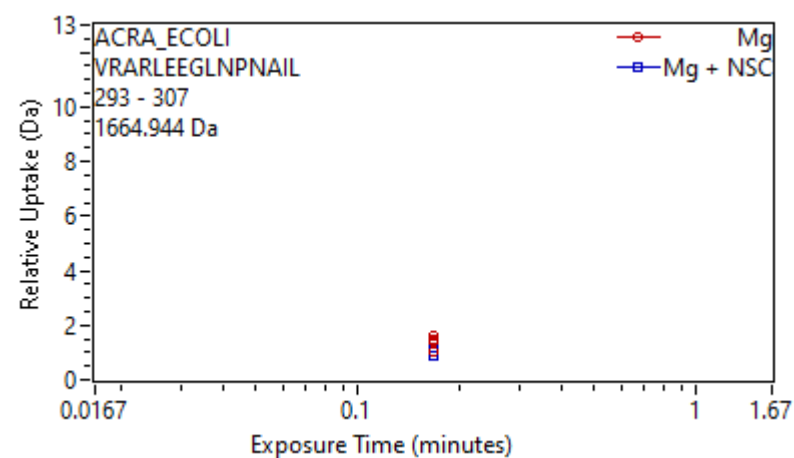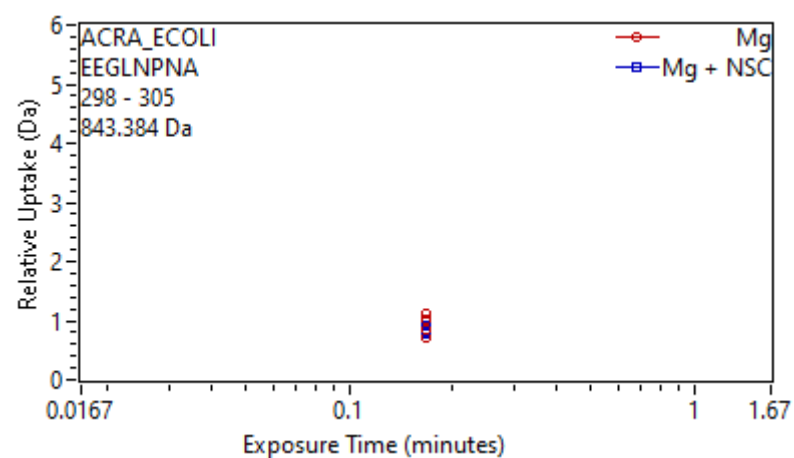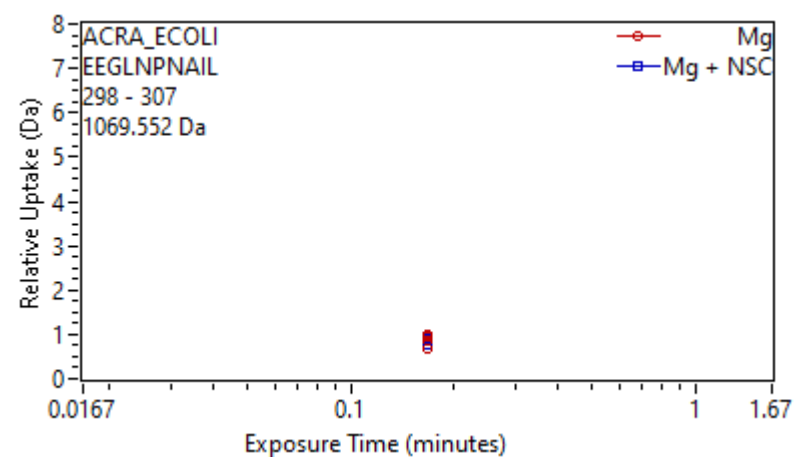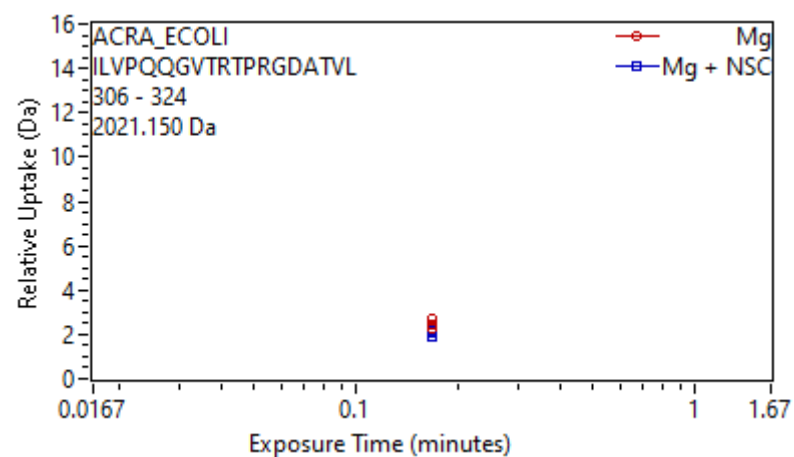

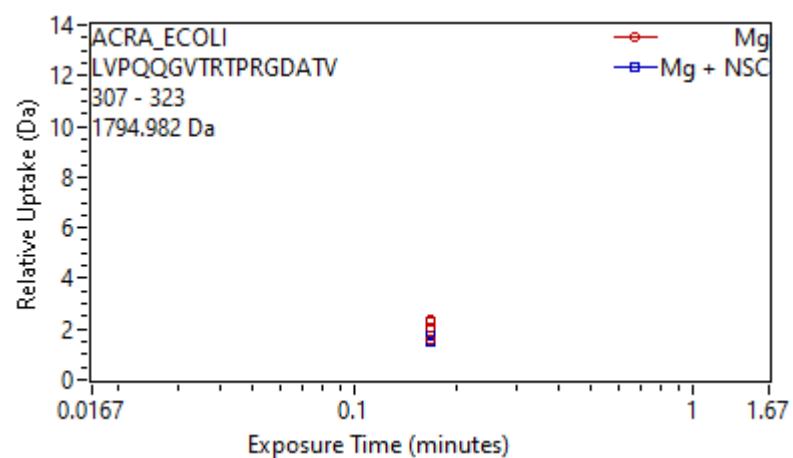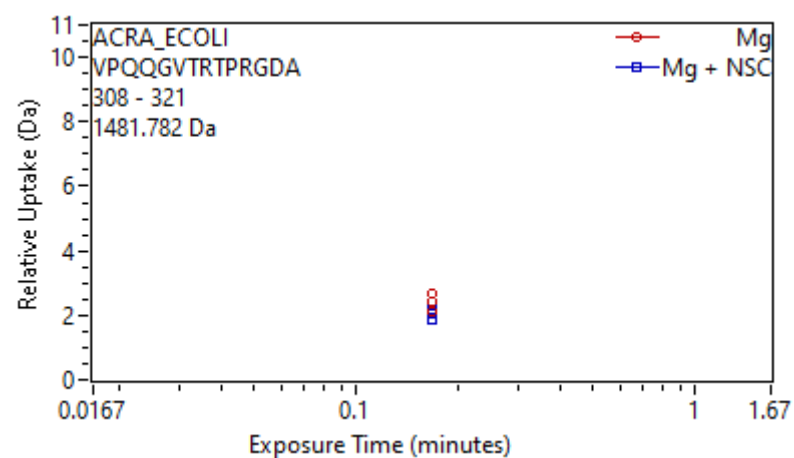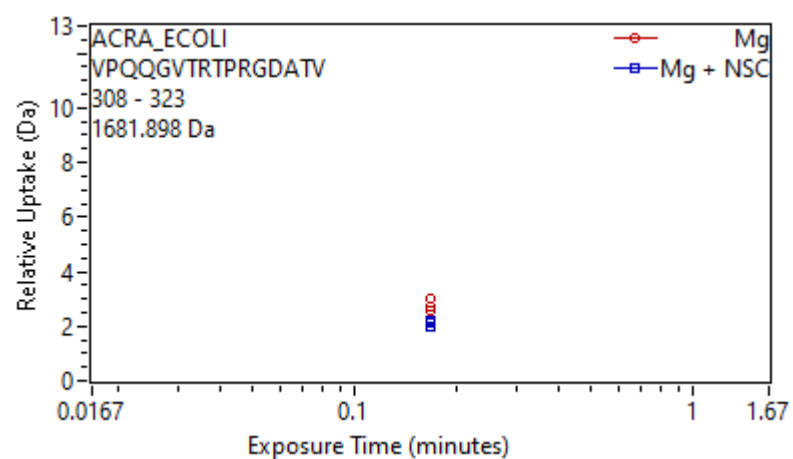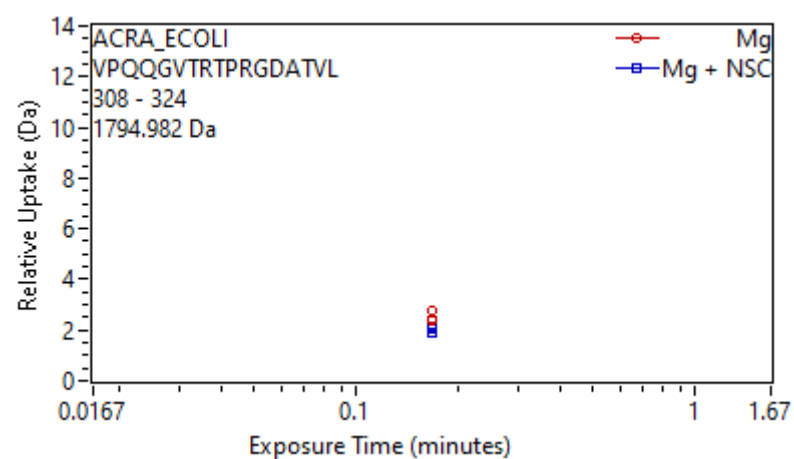

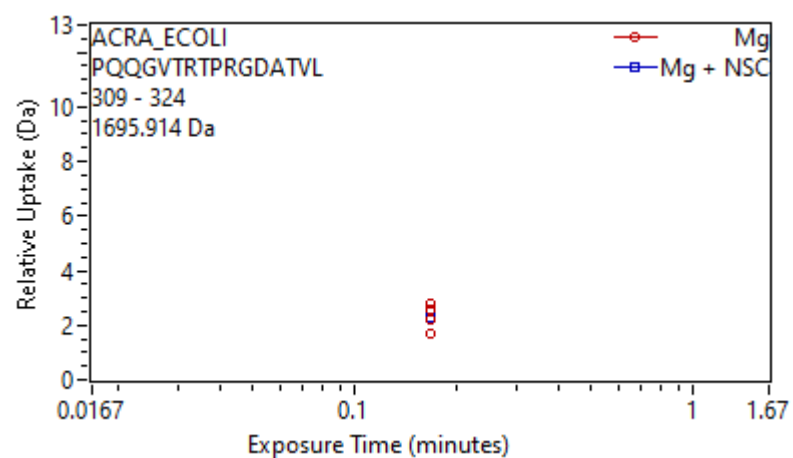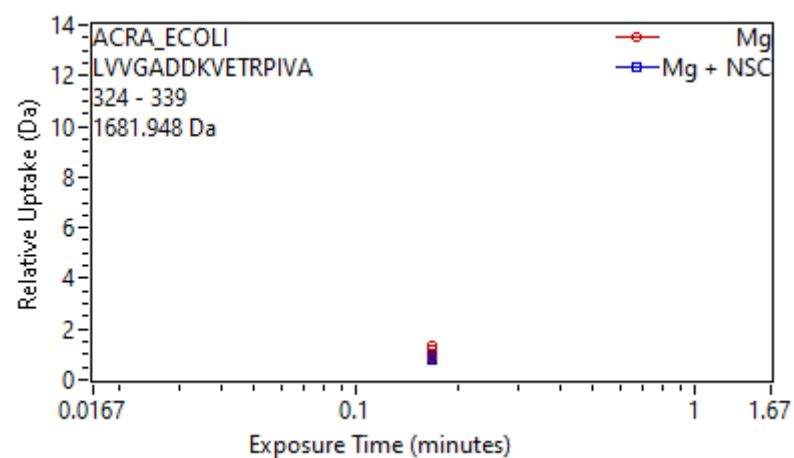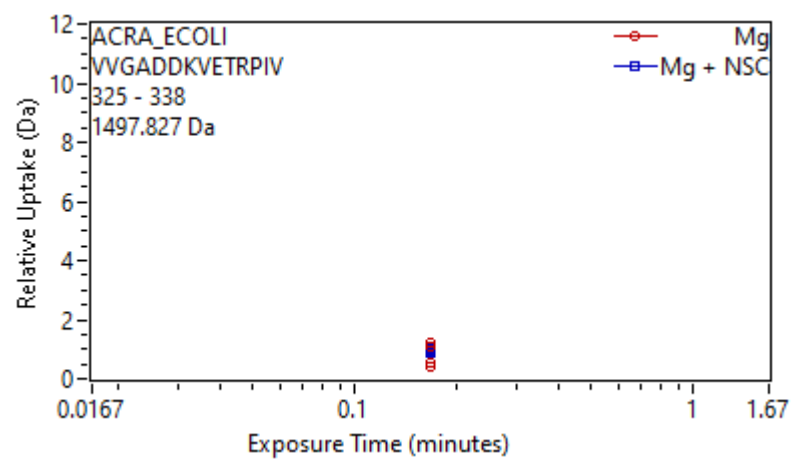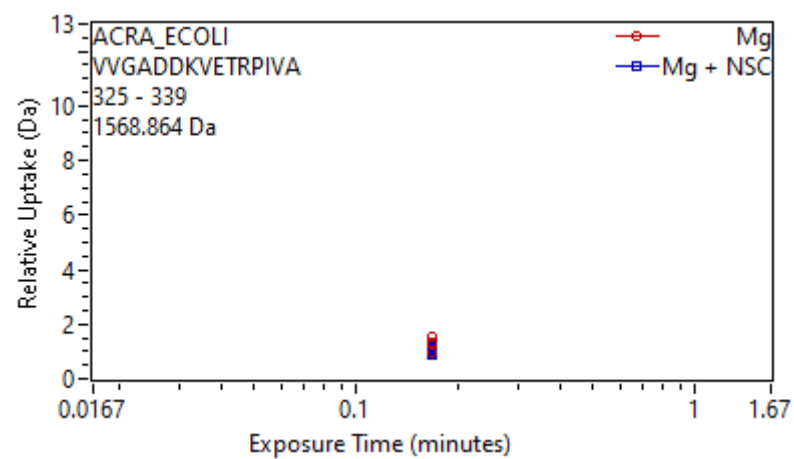

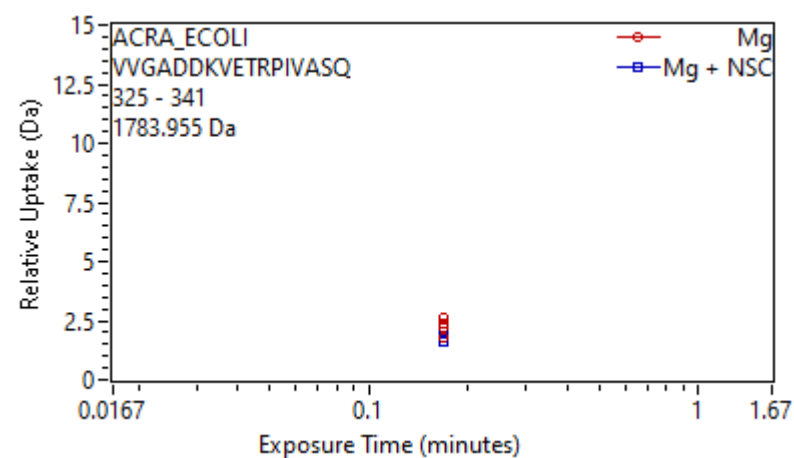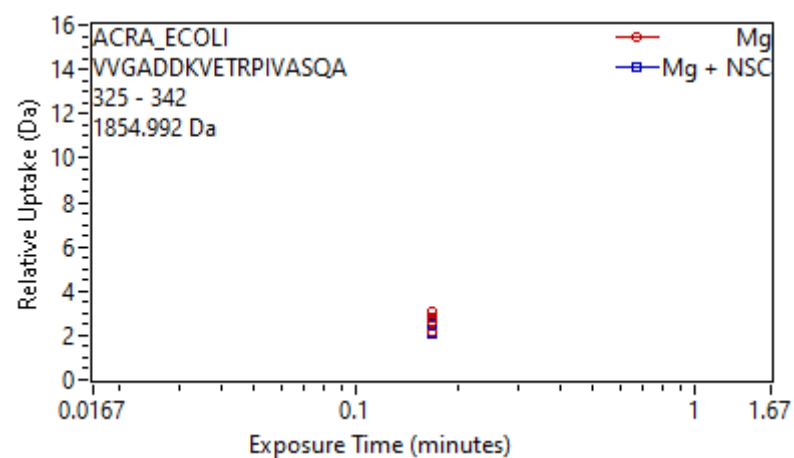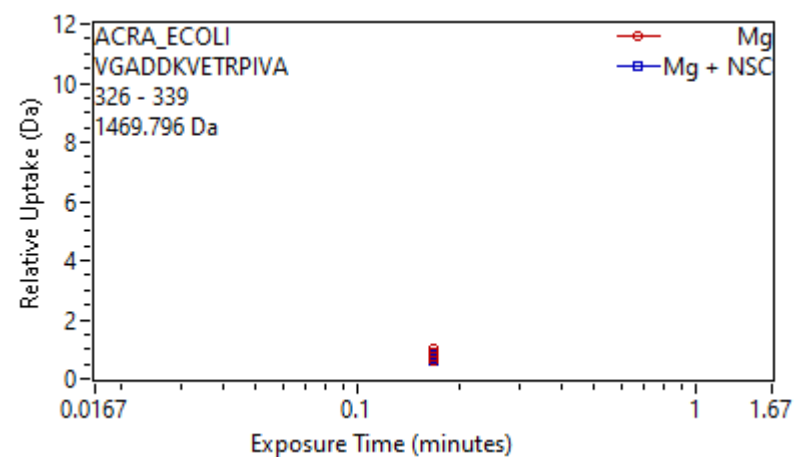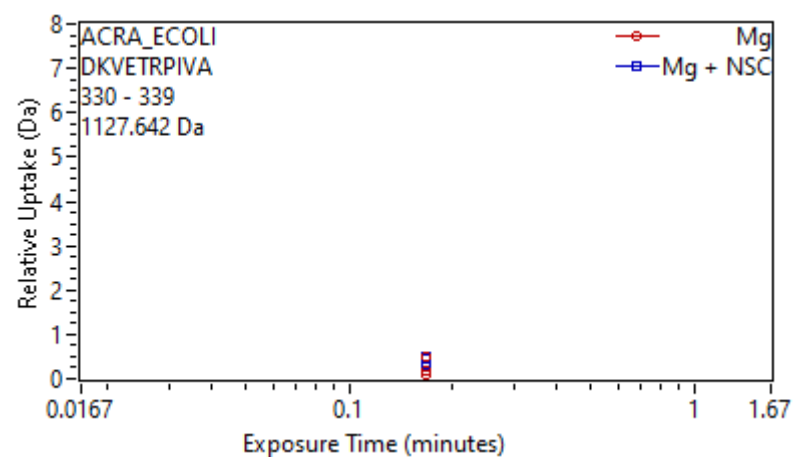

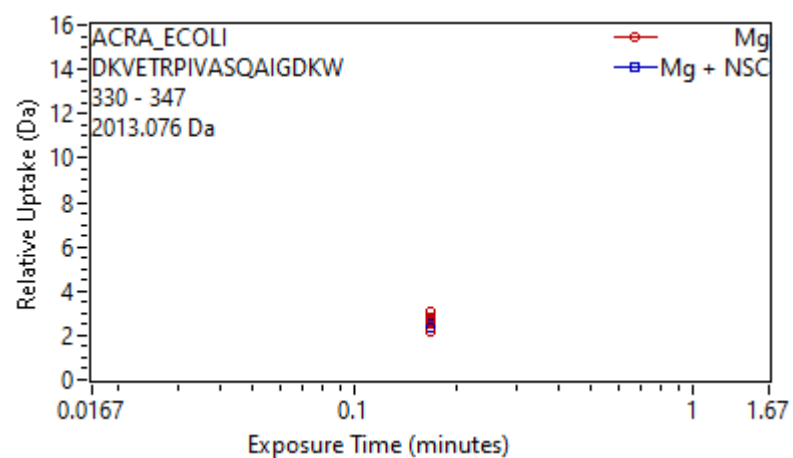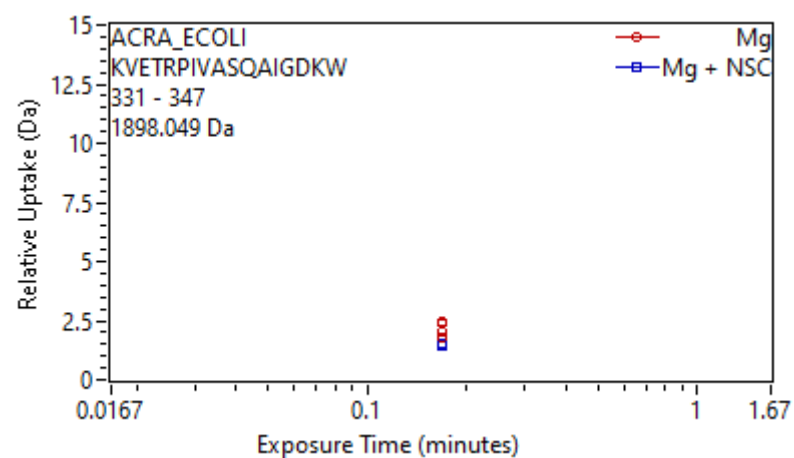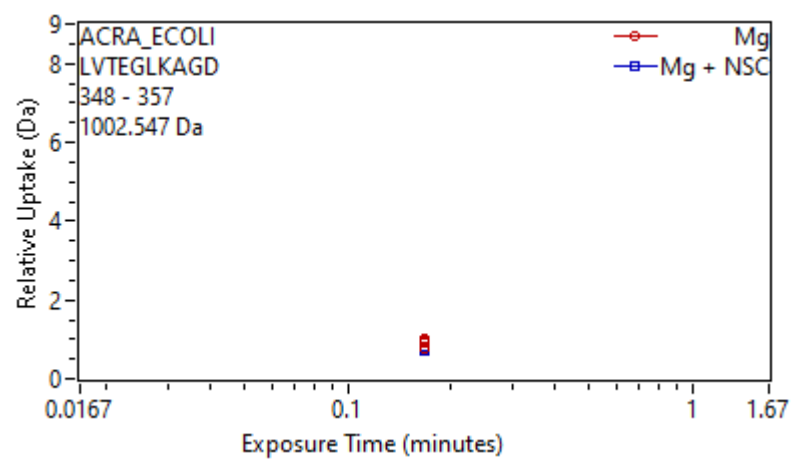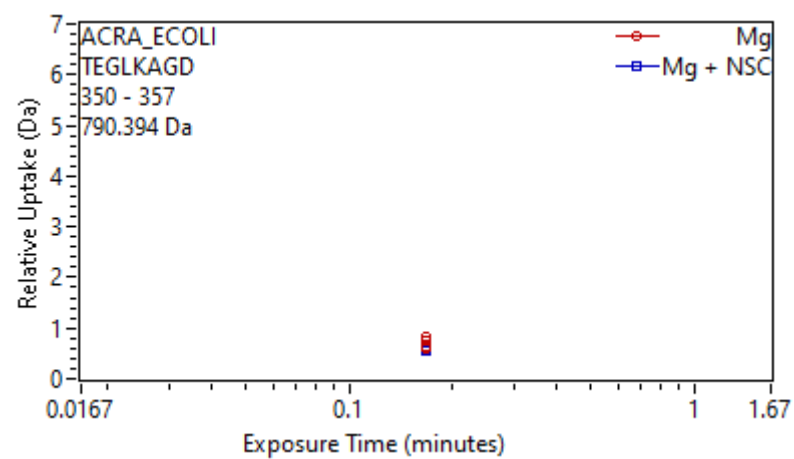

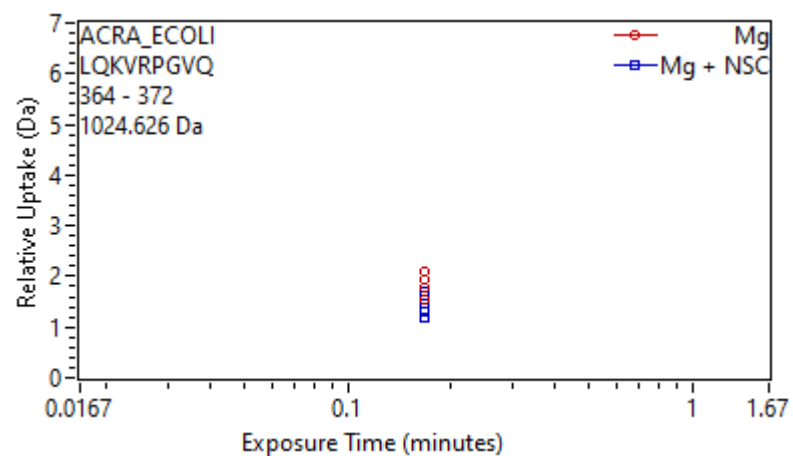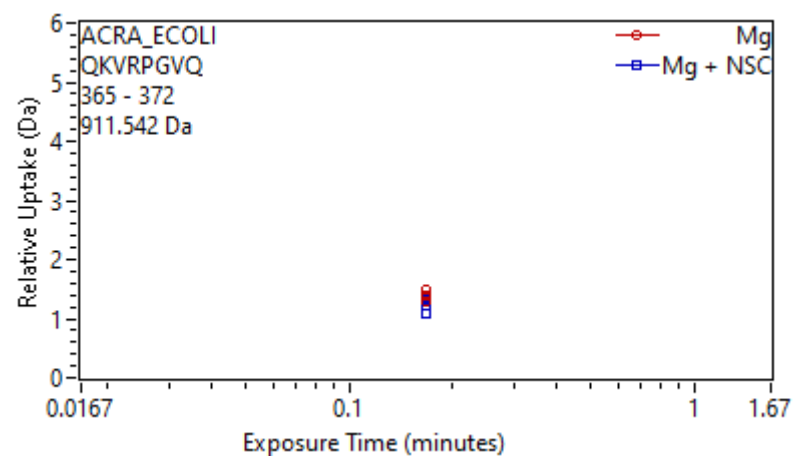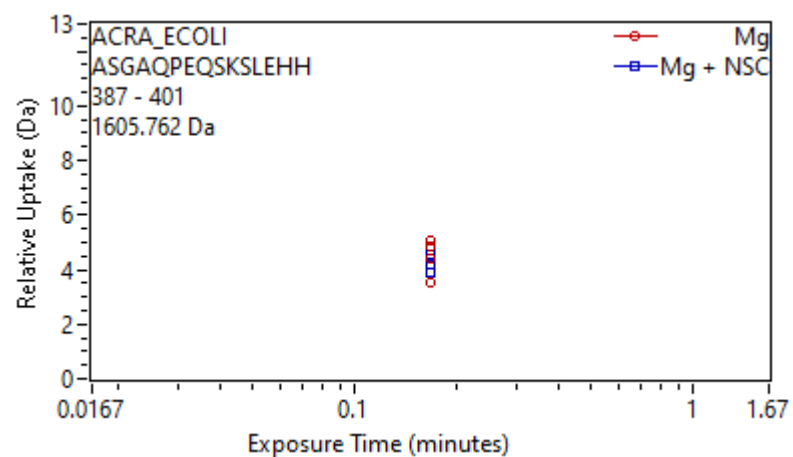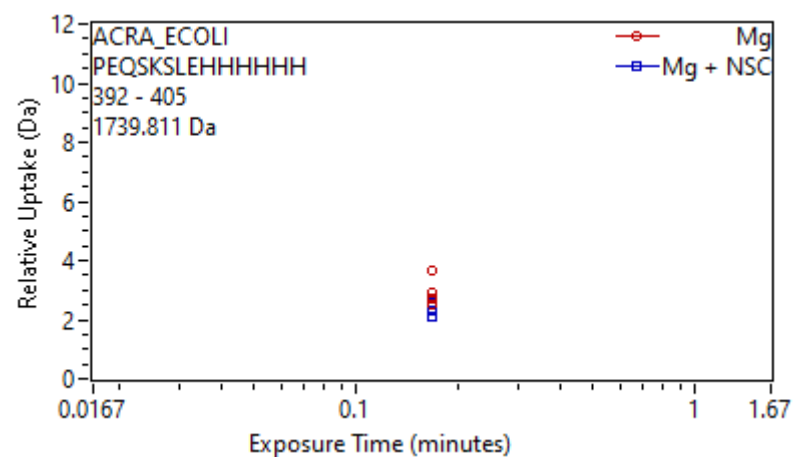

Supplement: Supplement 4 [file media-4.zip › Supplementary Data 3/Uptake plots 5.pdf]
